# Rhodaelectro-catalyzed access to chromones via formyl C–H activation towards peptide electro-labeling

**DOI:** 10.1038/s41467-021-25005-8

**Published:** 2021-08-05

**Authors:** Maximilian Stangier, Antonis M. Messinis, João C. A. Oliveira, Hao Yu, Lutz Ackermann

**Affiliations:** Institute for Organic and Biomolecular Chemistry, Georg-August-Universität Göttingen, Göttingen, Germany

**Keywords:** Electrocatalysis, Reaction mechanisms, Synthetic chemistry methodology

## Abstract

Chromones represent a privileged scaffold in medicinal chemistry and are an omnipresent structural motif in natural products. Chemically encoded non-natural peptidomimetics feature improved stability towards enzymatic degradation, cell permeability and binding affinity, translating into a considerable impact on pharmaceutical industry. Herein, a strategy for the sustainable assembly of chromones via electro-formyl C–H activation is presented. The rational design of the rhodaelectro-catalysis is guided by detailed mechanistic insights and provides versatile access to tyrosine-based fluorogenic peptidomimetics.

## Abstract (web summary)

The chromone scaffold is present in drugs and bioactive natural products, but conventional approaches to access chromones require stoichiometric amounts of oxidants. Here, the authors report rhodaelectro-catalyzed assembly of chromones by electrochemical formyl C–H activations, providing the basis for late-stage peptide diversification.

## Introduction

**Fig. 1:**
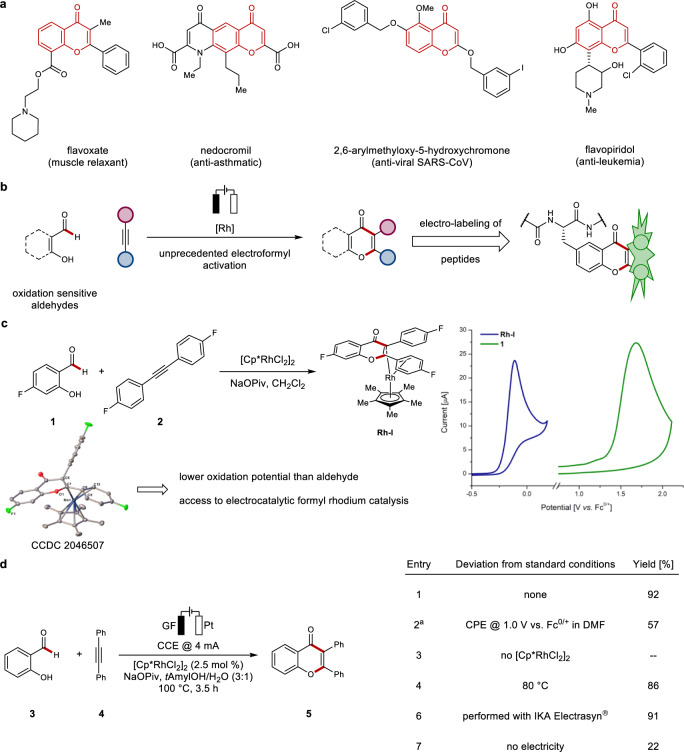
Motivation, rationale, and development of rhodaelectro-catalyzed annulation of benzaldehydes. **a** Chromones as a privileged motive in pharmaceutical and bioactive compounds. **b** Strategy to access chromones via rhodaelectro catalysis and its use for electro-peptide labeling. **c** Synthesis of rhodium complex **Rh-I** and investigations of its redox properties by cyclic voltammetry in CH_2_Cl_2_ with *n*Bu_4_NPF_6_ (0.2 M). **d** Reaction development, 0.25–0.50 mmol scale, 4.0–8.0 mL solvent, isolated yields. ^a^With *n*Bu_4_NPF_6_ (0.1 M) for 4 h. Cp* pentamethylcyclopentadienyl, NaOPiv sodium pivalate, Fc ferrocene, GF graphite felt electrode, CCE constant current electrolysis, CPE constant potential electrolysis, *t*AmylOH 2-methyl-2-butanol.

C–H activation has surfaced as a transformative strategy for molecular synthesis, with remarkable applications to materials science and late-stage diversification^1–6^. Metal-catalyzed hydroacylations have attracted major attention due to their high atom-economy, providing efficient access to substituted ketones^7,8^. In this context, functionalizations of hydroxy-benzaldehydes have proven to be a particularly enabling approach for the assembly of oxygen-containing heterocycles^9–11^, such as *β*-hydroxyketones, aurones, coumarines, and chromones^12–16^. Despite of major advances, this approach was largely limited by the need for stoichiometric amounts of chemical oxidants, compromising the inherent sustainable nature of the formyl C–H activation strategy. In recent years, a renaissance of organic electrosynthesis^17–23^ has provided a major impetus for efficient C–H activations^24–26^. Thereby, stoichiometric amounts of, often toxic, metal-containing oxidants can be avoided. Yet, while major advances in electro-organic synthesis have been noted^27–29^, challenging electro-functionalizations of oxidation-sensitive aldehydes continue to be scarce. Thus, the lability of aldehydes towards decarbonylation, overoxidation, and nucleophilic attack renders an electrochemical approach by anodic electro-oxidation particularly difficult^7,8^. The chromone scaffold is among others present in the commercialized drugs flavoxate and nedocromil^30,31^ as well as in bioactive natural products of relevance to acute myeloid leukemia^32^ and antiviral activity against SARS-associated corona viruses (Fig. 1a)^33,34^.

In this work, we present an electro-formyl C–H activation via rhodaelectro catalysis for the assembly of substituted chromones to provide sustainable access to amino acid chromone hybrids and to label peptides^35,36^ through metallaelectro catalysis.

## Results and discussion

To put our hypothesis into practice, we designed intermediates that feature lower oxidation potentials than the sensitive aldehyde substrates themselves (Fig. 1b). To this end, a stoichiometric transformation of hydroxybenzaldehyde **1**, alkyne **2** and [Cp*RhCl_2_]_2_ in the presence of base delivered rhodium(I) complex **Rh-I** (Fig. 1c), which was unambiguously characterized by X-ray diffraction analysis. With the proposed key intermediates in hand, we probed their redox properties towards an oxidation manifold under electrocatalytic conditions. Studies by cyclic voltammetry revealed that the complex **Rh-I** underwent irreversible oxidation to Rh(III) at *E*_p_ = –0.11 V vs. Fc^0/+^ and therefore exhibited a considerably lower oxidation potential than benzaldehyde **1** (*E*_p_ = 1.68 V vs. Fc^0/+^). With respect to an oxidatively induced reductive elimination from a rhodium(III/IV)-species^37,38^, calculations by means of DFT at the PW6B95-D3(BJ)/def2-QZVP+SMD(acetonitrile)//PBE0-D3(BJ)/def2-SVP level of theory at 298.15 K revealed an oxidation potential of *E*_1/2_ = 0.48 V vs. Fc^0/+^ of the corresponding seven-membered rhoda(III)-cycle, which is in proximity to the experimentally determined, oxidation potentials of related species at *E*_p/2_ = 0.68 V vs. Fc^0/+^^37,38^. Further computational studies of the electro-formyl C–H activation were supportive of a kinetically, favorable oxidatively induced reductive elimination (Supplementary Fig. 21).

**Fig. 2:**
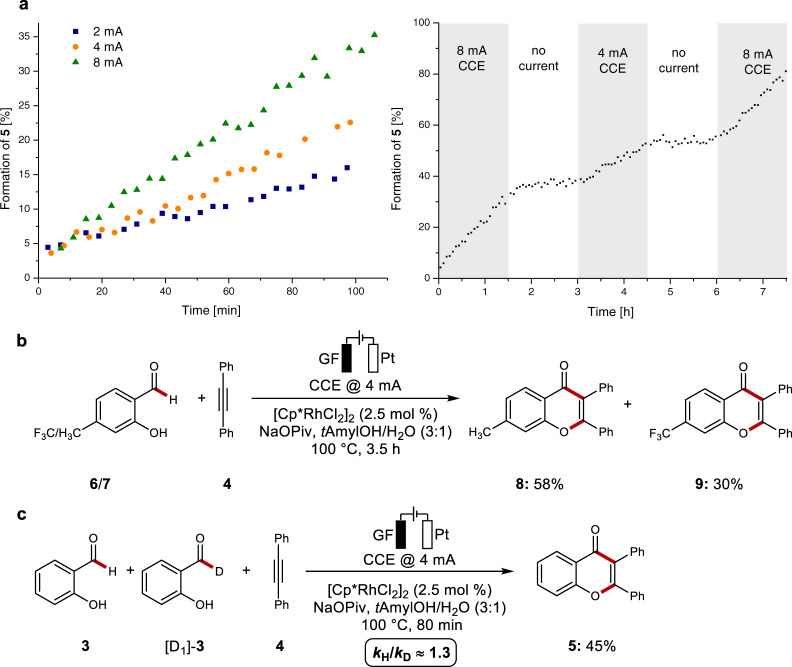
Mechanistic investigations on the rhodaelectro-catalyzed annulation. **a** Influence of the applied current on the reaction rate. **b** Competition experiment. **c** KIE studies.

The isolation and electroanalytical characterization of the key intermediate set the stage for studies on the electrocatalysis, initially with a constant potential of 1.0 V vs. Fc^0/+^, employing [Cp*RhCl_2_]_2_ as the catalyst and NaOPiv as the base to ensure an oxidatively induced reductive elimination. Hence, the desired chromone was obtained in 57% yield (Fig. 1d, entry 2), which is in line with our initial hypothesis for the rhodaelectro-catalyzed formyl activation. Further optimization demonstrated that the reaction furnished chromone **5** likewise under user-friendly galvanostatic conditions, with *t*AmOH/water (3:1) as the reaction medium avoiding additional electrolytes (Fig. 1d, entry 1). Control experiments revealed the crucial role of the rhodium precatalyst (entry 3). Importantly, the reaction was also viable with commercial equipment (Fig. 1d, entry 6). To test the role of electricity in the rhodaelectro-catalyzed formyl C–H activation, we performed an in-operando monitoring of the catalysis at different currents by in situ ^1^H-NMR spectroscopy (Fig. 2a).

Indeed, the reaction rates are strongly dependent on the applied currents, indicating a turnover limiting electron transfer being operative. Additionally, an on/off experiment was conducted, clearly reflecting the key role of electricity for efficient catalyst turnover. To probe the catalysts mode of action, an intermolecular competition experiment between differently substituted salicylic aldehydes **6**/**7**, revealed an inherent higher reactivity of the electron-rich substrate (Fig. 2b), being suggestive of a base-assisted internal electrophilic substitution-type (BIES) manifold^39^. A minor kinetic isotope effect was observed, again being in line with a rate limiting reoxidation (Fig. 2c).

**Fig. 3:**
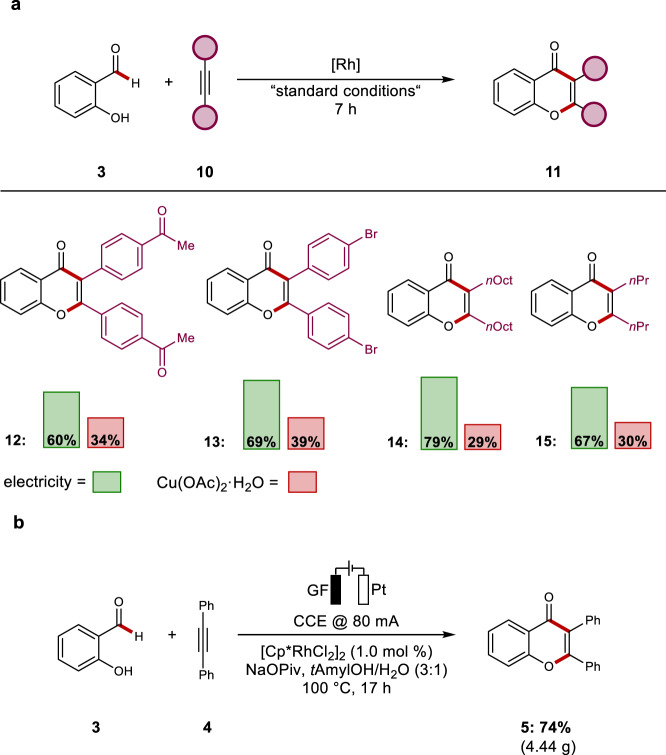
Power of rhodaelectro-catalyzed annulation. **a** Comparison between rhodaelectro catalysis and Cu(OAc)_2_·H_2_O as the chemical oxidant. **b** Rhodaelectro-catalyzed annulation on multigram scale.

To benchmark the presented electro-catalyzed formyl C–H activation, we compared its performance with challenging substrates **10**, such as electron-deficient diphenylacetylenes and alkynes with aliphatic substituents. Thus, the efficacy towards the formation of products **12**–**15** was found to be uniquely effective under the electrocatalytic conditions, as compared to Cu(OAc)_2_ as the oxidant, highlighting the superior performance of the rhodaelectro catalysis (Fig. 3a). Additionally, the scalability of the rhodaelectro-catalyzed transformation was highlighted with a multigram scale synthesis with reduced catalyst loading (Fig. 3b).

**Fig. 4:**
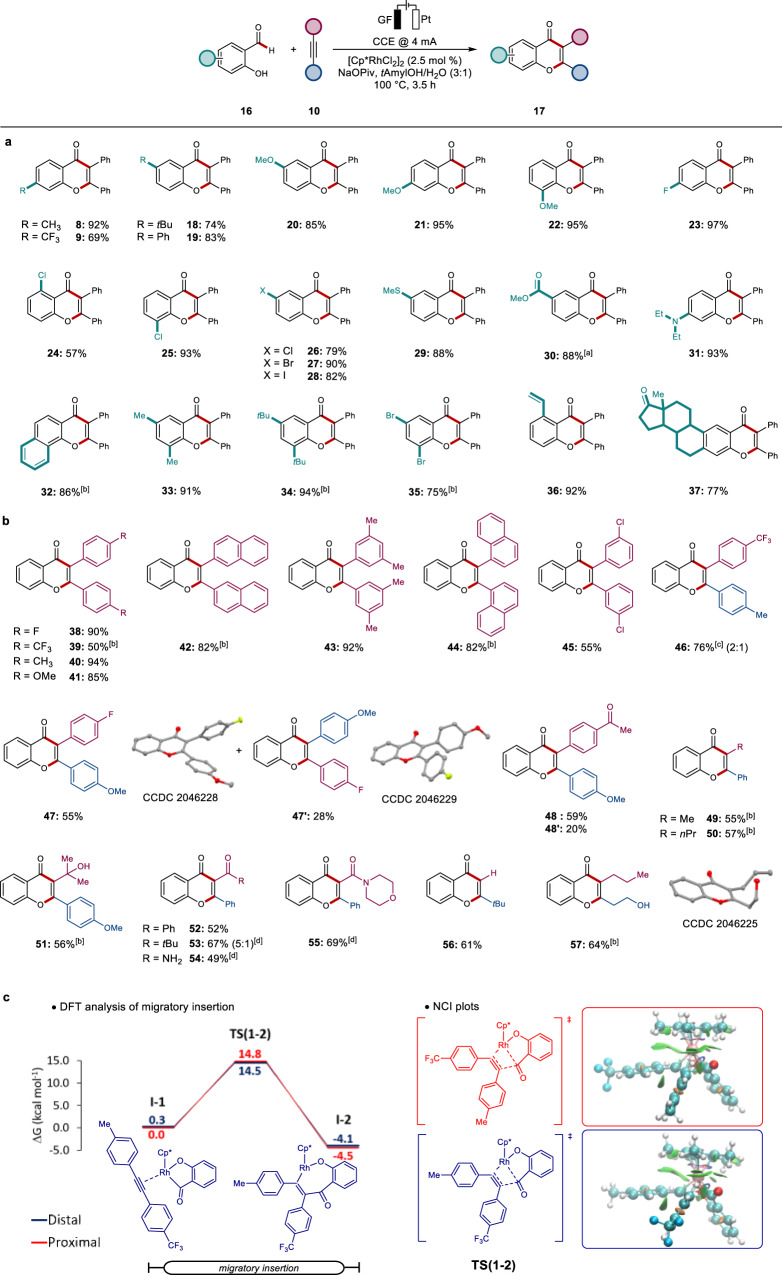
Versatility of the rhodaelectro-catalyzed assembly of chromones and computational studies on the origin of regioselectivity. **a** Aldehyde scope for various substituents. **b** Scope of viable alkynes. **c** Calculated energy profiles for the regioselective determining step for the Me/CF_3_ substituted alkyne at the PW6B95-D3(BJ)/def2-QZVP+SMD(methanol)//PBE0-D3(BJ)/def2-SVP level of theory and spatial localization of noncovalent interactions in the transition states. In the latter, red indicate repulsive interactions, with blue and green being attributed to strong and weak attractive interactions. ^a^With *n*Bu_4_NPF_6_ (0.1 Μ) instead of H_2_O. ^b^7 h. ^c^Ratio determined by ^19^F-NMR. ^d^With 5.0 mol % [Cp*RhCl_2_]_2_.

With the optimized conditions in hand, we explored the scope of salicylic aldehydes **16** (Fig. 4a). Overall, differently substituted aldehydes efficiently furnished the desired products **18**–**36**. Especially redox sensitive groups, such as bromo-, iodo-, and thioether substituents were fully tolerated and delivered the corresponding products **27**–**29**. The electron-deficient substrate with an ester substituent under water-free conditions, as well as salicylic aldehyde with an amine functionality were selectively converted to the corresponding chromones **30** and **31**, respectively. Even bulky disubstituted aldehydes delivered the desired products **34** and **35** in very good yields. To our delight, also the estrone derivative was converted to the product **37**.

Subsequently, we turned our attention to the viable alkyne **10** scope in greater detail (Fig. 4b). Electron-rich alkynes were efficiently converted to the corresponding chromones **40** and **41**. *meta*- and sterically encumbered *ortho-*arylalkynes were also tolerated by the rhodaelectro catalysis regime (**42**–**45**).

Subsequently, a wealth of unsymmetrically substituted alkynes were efficiently converted to the desired products **46–57** (Fig. 4b). Thereby, terminal as well as keto- and amido-substituted alkynes underwent the rhodaelectro catalysis with high efficacy (**52–56**). As to the regioselectivity of unsymmetrical alkynes, the alkyne substrates furnished chromones **49**–**55** with the aryl motif proximal to the oxygen-heteroatom. Under otherwise identical reaction conditions, alkynes with nitrile, ester or free acid groups provided less satisfactory results. It is noteworthy that hydroxyheptyne (**10w**) exclusively yielded the regioisomer **57**. In order to gain insights into the origin of the regioselectivity for the synthesis of chromone **57**, DFT calculations were carried out for the migratory insertion step at the PW6B95-D3(BJ)/def2-QZVP+SMD(methanol)//PBE0-D3(BJ)/def2-SVP level of theory (Supplementary Fig. 22). The transition state leading to regioisomer **57** with the hydroxyl distal to the carbonyl group of the substrate is favored by 2.4 kcal mol^−1^, which can be attributed to favorable hydrogen bonding interactions with the hydroxyl group in the transition state structure (**TS(1-2)**, Supplementary Fig. 22). Likewise, the regioselectivity of the electrocatalytic C–H activation with 4-methyl-4’-trifluoromethyltolane could be rationalized by DFT computation (Fig. 4c). The calculated regioselectivity was in good agreement with the experimental observations. Here, noncovalent interactions were found to be of minor relevance.

**Fig. 5:**
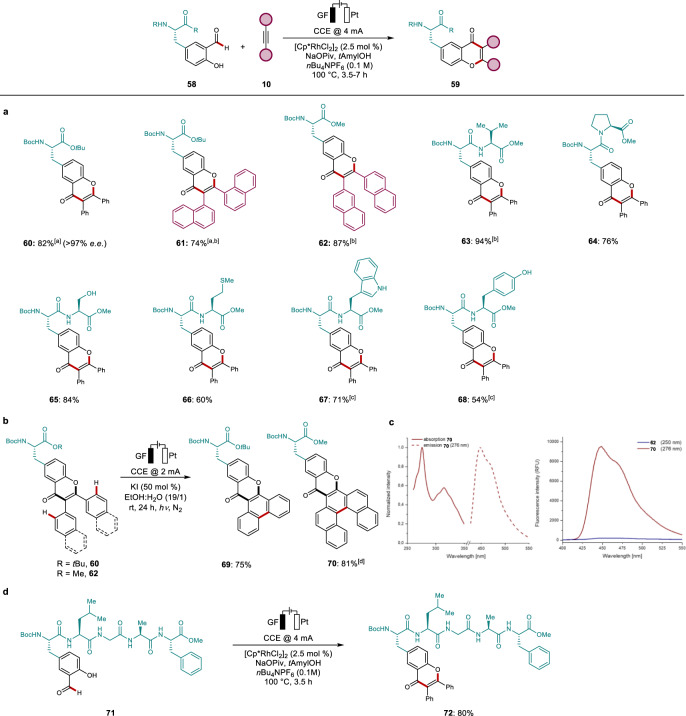
Development of amino acid and peptide labels. **a** Scope of rhodaelectro-catalyzed peptide labeling. **b** Photoelectrochemical annulation towards π-extended peptide labels. **c** Absorption and emission properties of **62** (10 mg/L) and **70** (0.5 mg/L) in CHCl_3_. **d** Late-stage functionalization of pentapeptide **71**. ^a^With *t*AmylOH/H_2_O (3:1). ^b^7 h. ^c^With 5.0 mol % of [Cp*RhCl_2_]_2_. ^d^With MeOH instead of EtOH.

Chemically encoded peptidomimetics reduce enzymatic degradation and feature superior binding affinities, cell permeability, and pharmacokinetics^40,41^. However, the functionalization of structurally complex peptides mostly rely on terminal peptides, azide-based click chemistry or on the innate reactivity of cysteine^42–44^. Given the practical importance of late-stage peptide diversifications, we became attracted to tyrosine modifications through rhodaelectro catalysis to access tyrosine-derived fluorescent amino acids. Indeed, tyrosines were chemo-selectively annulated with tolane and naphthalene derived alkynes furnishing the desired products **60–62**. Next, we probed dipeptides to explore rhodaelectro-catalytic site-specific labeling (Fig. 5a). To our delight, a broad variety of dipeptides was efficiently converted to the corresponding products **63–68**. Notably, even oxidation-sensitive serine (**65**) and methionine (**66**) containing peptides were transformed to the desired products. Furthermore, potentially coordinating dipeptides with unprotected tryptophan, or tyrosine regioselectively provided the desired products **67** and **68**.

We explored an improvement of the photoelectronic properties of the thus-obtained labels by a late-stage annulation. The aryl moieties were selectively transformed into π-extended peptide labels in a photoelectrochemical process to obtain the desired products (Fig. 5b). Labels **69** and **70** demonstrated improved photoelectronic properties in comparison to the corresponding diaryl precursors **60** and **62** (Fig. 5c and Supplementary Table 2). Since compound **70** exhibited intense fluorescence at 458 nm, it bears considerable potential as a fluorogenic probe^45,46^.

Finally, we probed our strategy for the functionalization of structurally complex oligopeptide **71**. Indeed, peptide **72** was obtained in excellent yield, highlighting the unique power of the rhodaelectro-catalytic labeling strategy (Fig. 5d).

In summary, we have reported on a rhodaelectro-catalyzed transformation of hydroxy-benzaldehydes by electrochemical formyl C–H activation, featuring scalability, high functional group tolerance, and improved efficiency in comparison to chemical oxidants. The strategy proved applicable to the functionalization of tyrosine derivatives, enabling difficult to perform, site-selective electro-labeling of amino acids and peptides by formyl C–H activation. A mediated photoelectrochemical oxidation allowed for an enhancement of fluorescence properties of the thus-obtained amino acids. The rhodaelectro catalysis was inspired by in-depth mechanistic insights, through the isolation and electroanalytical characterization of key intermediates, providing strong support for an oxidation-induced reductive elimination.

## Methods

### Rhodaelectro-catalyzed formyl C–H activation

The electrocatalysis was carried out in an undivided cell, with a graphite felt (GF) anode (25 × 10 × 6.0 mm) and a platinum cathode (25 × 10 × 0.125 mm). The 2-hydroxylbenzaldehyde (0.75 mmol), the alkyne (0.25 mmol), NaOPiv (62 mg, 0.50 mmol), [Cp*RhCl_2_]_2_ (3.9 mg, 2.5 mol %), and *t*AmylOH/H_2_O (4.0 mL, 3:1) were placed in a 10 mL cell. Electrocatalysis was performed at 100 °C with a constant current of 4 mA maintained for 3.5–7.0 h. Then, the DC-power supply was stopped and the reaction mixture was diluted with EtOAc (2.0 mL). The electrodes were washed with EtOAc (Pt: 1 × 5.0 mL; C: 3 × 10.0 mL). The solvents were combined with the reaction mixture, silica gel was added and the solvents were removed in vacuo. Subsequent column chromatography on silica gel afforded the corresponding products.

## Supplementary information

Supplementary Information

## Data Availability

The authors declare that the data supporting the findings of this study are available within the paper and its supplementary information files. The X-ray crystallographic coordinates for structures reported in this study have been deposited at the Cambridge Crystallographic Data Centre (CCDC), under deposition numbers 2046225, 2046228-2046229, 2046502-2046508. These data can be obtained free of charge from The Cambridge Crystallographic Data Centre via www.ccdc.cam.ac.uk/data_request/cif. All other requests for materials and information should be addressed to the corresponding authors. Source data are provided with this paper.

## Source data

Source Data
