# Supplementary material for: Rhodaelectro-catalyzed access to chromones via formyl C–H activation towards peptide electro-labeling
Source: Nat Commun. 2021 Aug 5;12:4736. doi: 10.1038/s41467-021-25005-8 (PMC8342597; doi:10.1038/s41467-021-25005-8)
Supplement: Supplementary file 1 — Supplementary Information [file 41467_2021_25005_MOESM1_ESM.pdf]

Supplementary Information

Rhodaelectro-Catalyzed Access to Chromones via Formyl C–H Activation towards Peptide Electro-Labeling

Maximilian Stangier [1], Antonis Messinis[1], João C. A. Oliveira[1], Hao Yu[1] and Lutz Ackermann*[1]

Institute for Organic and Biomolecular Chemistry, Georg-August-Universität Göttingen

Tammannstr. 2, 37077 Göttingen, Germany

*Lutz.Ackermann@chemie.uni-goettingen.de

Table of Contents

1. General Information S3
2. Variations of the standard conditions S4
3. General Procedures..S5
4. Isolation of reactions intermediates S7
5. Cyclic voltammetry S14
6. On-Line NMR Monitoring in Flow S17
7. KIE studies S19
8. Competition experiment S20
9. Multi-gram scale synthesis S22
10. UV/Vis and Fluorescence Measurements S23
11. Proposed Catalytic Cycle S24
12. X-Ray Crystallographic Analyses S25
13. Experimental and Analytical Data for Products S37
14. Studies on Potential Racemization S79
15. Computational Studies..S80
16. NMR Spectra S85
17. Supplementary References S162

1. General Information

Catalytic reactions were carried out in undivided electrochemical cells (10 mL) using pre-dried glassware, if not noted otherwise. 2-Hydroxybenzaldehydes, alkynes and solvents were obtained from commercial sources. Alkynes **10a-10n**[1], **10r-10s**[2], **10t**[3], **10u**[4] and hydroxybenzaldehydes **3D**,[5] **6,**[6] **16b**,[7] **16j**,[8] **16k**,[9] **16r**[10] and tyrosine-derived hydroxybenzaldehyde **58a**[11,12] were synthesized according to previously described procedures. Peptides were synthesized under standard solution phase protocols (EDCI/HOBt). Platinum electrodes (10 mm × 15 mm × 0.25 mm, 99.9%; obtained from ChemPur®, Karlsruhe, Germany) and graphite felt electrodes (10 mm × 15 mm × 6 mm, SIGRACELL® GFA 6 EA, obtained from SGL Carbon, Wiesbaden, Germany) were connected using stainless steel adapters. Electrocatalysis was conducted using an AXIOMET AX-3003P potentiostat or an IKA Electrasyn® 2.0 set-up in constant current mode. Photoelectro-catalyzed annulations[13] were performed under N_2 atmosphere in pre-dried quartz tubes using a Luzchem LZC-ICH2 photoreactor with 254 nm irradiation with the electrolysis equipment mentioned above. Constant Potential electrolysis was conducted using a portable multi potentiostat Metrohm Dropsens μStat 8000P. The ^{1}H-NMR spectroscopy experiments in flow were performed on a Magritek Spinsolve 60ULTRA from Magritek GmbH, Germany. Cyclic Voltammetry studies were performed using a Metrohm Autolab PGSTAT204 workstation and Nova 2.1 software. Yields refer to isolated compounds, estimated to be >95% pure as determined by ^{1}H-NMR. Chromatography was carried out on Merck silica gel 60 (40–63 µm). NMR spectra were recorded on a Varian Mercury VX 300, Inova 500 or Bruker Avance III 300, Avance III 400 and Avance III HD 500 in the solvent indicated; chemical shifts (δ) are given in ppm relative to the residual solvent peak. All IR spectra were recorded on a Bruker FT-IR Alpha-P device. EI-MS was recorded on Jeol AccuTOF at 70eV, ESI-MS on Bruker MicrOTOF and maXis. GC-MS was recorded on Agilent 7890B and Agilent 5977B. M. p.: Stuart melting point apparatus SMP3, Barloworld Scientific, values are uncorrected. X-ray structures were measured on Bruker APEX-II CCD diffractometer. UV-Vis absorption data were recorded on a Jasco® V-770 spectrophotometer. The scan speed was adjusted to 400 nm/min. Fluorescence excitation and emission data in solution were recorded on a Jasco® FP-8500 spectrofluorometer. The scan speed was adjusted to 500 nm/min. The concentration in $CHCl_3$ is given for each sample and the excitation wavelengths were selected at the strongest signal. HPLC chromatograms were recorded on an Agilent 1290 Infinity using CHIRALPAK® IA-3, IB-3 and ID-3 columns (3.0 µm particle size; Ø: 4.6 mm and 250 mm length).

2. Variations of the standard conditions

Supplementary Table 1. Optimization and control experiments for the rhodaelectro-catalyzed annulation of 2-hydroxy benzaldehyde **3** and alkyne **4**.

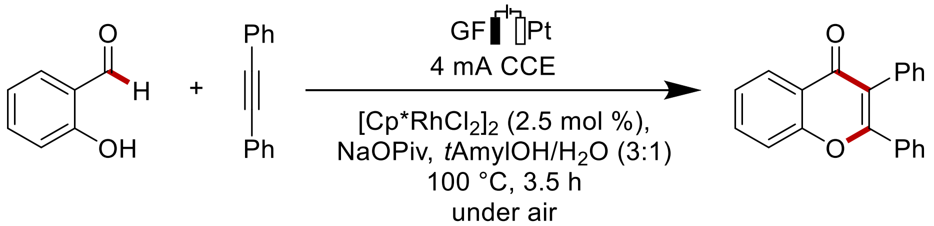

3 **4** **5**

Entry	Deviation from standard conditions	Yield [%][a]
1	none	92
2[b]	CPE @ 1.0 V *vs.* $Fc^{0/+}$ in DMF	57
3	No current	22
4	Under N_2	88
5	80 °C	86
6	40 °C	37
7	$[Cp^{*}RhCl_2]_2$ (1.0 mol %)	85
8[c]	$[Rh(COD)Cl]_2$ (2.5 mol %), $C_5H_2Ph_4$ (10 mol %)	6
9	$[Rh(PPh_3)_3Cl]$ (5 mol %)	-
10	1.5 Eq of **1**	71
11	2.0 Eq. of **1**	86
12	1 h	37
13	2 h	68
14[d]	Performed with IKA Electrasyn®	91

Undivided cell, graphite felt (GF) anode, Pt cathode, constant current = 4.0 mA, **3** (0.75 mmol), **4** (0.25 mmol), catalyst (2.5 mol %), NaOPiv (0.50 mmol), solvent (4.0 mL), under air. [a] isolated Yield. [b] with nBu_4PF_6 (0.1 M) for 4 h. [c] Yield determined by ^{1}H-NMR spectroscopy with CH_2Br_2 as an internal standard. [d] constant current = 8.0 mA, **3** (1.50 mmol), **4** (0.50 mmol), NaOPiv (0.50 mmol), with RVC instead of GF.

3. General Procedures

General Procedure A for the rhodaelectro-catalyzed alkyne annulation

The electrocatalysis was carried out in an undivided cell, with a graphite felt (GF) anode (25 mm × 10 mm × 6.0 mm) and a platinum cathode (25 mm × 10 mm × 0.125 mm). 2-Hydroxybenzaldehyde (0.75 mmol), alkyne (0.25 mmol), NaOPiv (62 mg, 0.50 mmol), $[Cp^*RhCl_2]_2$ (3.9 mg, 2.5 mol %) and *t*AmylOH/H_2O (4 mL, 3:1) were placed in a 10 mL cell. Electrocatalysis was performed at 100 °C with a constant current of 4 mA maintained for 3.5–7 h. Then, the DC-power supply was stopped and the reaction mixture was diluted with EtOAc (2.0 mL). The platinum cathode and the graphite felt anode were washed with EtOAc (Pt: 1 × 5 mL; C: 3 × 10.0 mL). The solvents were combined with the reaction mixture, silica gel was added and the solvents were removed in vacuo. Subsequent column chromatography on silica gel afforded the corresponding products.

General Procedure B for the rhodaelectro-catalyzed alkyne annulation under water free conditions

The electrocatalysis was carried out in an undivided cell, with a graphite felt (GF) anode (25 mm × 10 mm × 6.0 mm) and a platinum cathode (25 mm × 10 mm × 0.125 mm). 2-Hydroxybenzaldehyde (0.75 mmol), alkyne (0.25 mmol), NaOPiv (62 mg, 0.50 mmol), $[Cp^*RhCl_2]_2$ (3.9 mg, 2.5 mol %), $n$$Bu_4NPF_6$ (155 mg, 0.1 M) and *t*AmylOH (4 mL) were placed in a 10 mL cell. Electrocatalysis was performed at 100°C with a constant current of 4 mA maintained for 3.5–7 h. Then, the DC-power supply was stopped and the reaction mixture was diluted with EtOAc (2.0 mL). The platinum cathode and the graphite felt anode were washed with EtOAc (Pt: 1 × 5 mL; C: 3 × 10.0 mL). The solvents were combined with the reaction mixture, silica gel was added, and the solvents were removed in vacuo. Subsequent column chromatography on silica gel afforded the corresponding products.

General Procedure C for the rhodium-catalyzed alkyne annulation using $Cu(OAc)_2$ as the terminal oxidant

2-Hydroxybenzaldehyde **3** (78 μL, 0.75 mmol), alkyne **10** (0.25 mmol), $Cu(OAc)_2 \cdot H_2O$ (99.8 mg, 0.50 mmol), NaOPiv (62 mg, 0.50 mmol), $[Cp^*RhCl_2]_2$ (3.9 mg, 2.5 mol %) and *t*AmylOH/H_2O (4 mL, 3:1) were placed in a 10 mL schlenk tube under nitrogen atmosphere. The reaction mixture was stirred at 100 °C for 7 h. The mixture was diluted with EtOAc (25 mL) and silica gel was added. The solvents were removed in vacuo and subsequent column chromatography on silica gel (*n*hexane/EtOAc) afforded the corresponding products.

4. Isolation of reactions intermediates

Synthesis and characterization of Rh-I

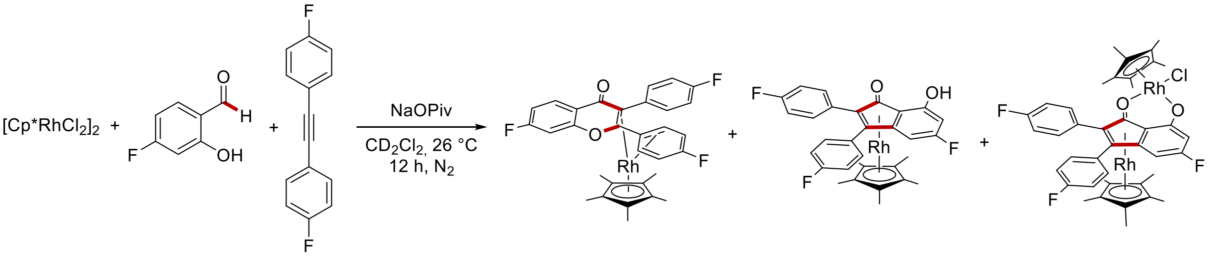

In a nitrogen filled glove box, an airtight NMR tube was loaded with $[Cp^*RhCl_2]_2$ (15.5 mg, 25 µmol), NaOPiv (12.4 mg, 0.100 mmol), and CD_2Cl_2 (500 µL). The mixture was briefly sonicated (1 min) and refluxed (1 min). Subsequently, 1-fluorononane (6.3 µL, 35 µmol) and alkyne **2** (10.7 mg, 50 µmol) were added and the $^{19}F\{H\}$-NMR spectrum of the solution was recorded. The reaction was then initiated by addition of 4-fluoro-2-hydroxybenzaldehyde (5.5 mg, 39 µmol) and monitored by $^{19}F\{H\}$-NMR spectroscopy over a period of 12 hours at 26 °C (Supplementary Supplementary Figure 2). The resulted dark red solution was then transferred to the glove box where it was filtered and dried under reduced pressure. The residue was then dissolved in Et_2O (0.3 mL) and upon standing at room temperature for 2 days dark red crystals formed which were isolated by decanting the solution and washed with pentane to afford pure **Rh-II** (6.9 mg, 53% yield). Subsequently, the filtrate was stored at – 20 °C for two days resulting in the formation of dark rectangular crystals of **Rh-I** which were suitable for an X-ray crystallographic analysis. The crystals were isolated by decanting the supernatant solution and washed with pentane affording pure **Rh-I** (2.1 mg, 16% yield). The filtrate was then analysed by HRMS (Supplementary Figure 3 and Supplementary Figure 4). A peak with a mass of 827.1085 amu was observed being in good agreement with the presence of a cationic bimetallic Rh complex such as **Rh-III** after chloride abstraction. Subsequently, the filtrate was stored at – 20 °C for 15 days during which time a crystal suitable for an X-ray crystallographic analysis was formed and found to be complex **Rh-III**.

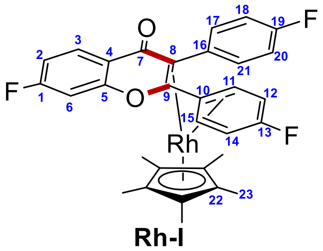

^{1}H-NMR (500 MHz, CD_2Cl_2): δ = 8.21 (dd, *J* = 8.8, 6.7 Hz, H3, 1H), 7.94 (ddd, *J* = 9.9, 5.7, 1.8 Hz, H15, 1H), 7.72 (ddd, *J* = 8.4, 5.7, 2.4 Hz, H17, 1H), 7.18 (ddd, *J* = 10.1, 8.0, 2.6 Hz, H14, 1H), 7.04 (dd, *J* = 9.6, 2.5 Hz, H6, 1H), 7.03 (ddd, *J* = 9.3, 9.3, 2.6 Hz, H18 or H20, 1H), 6.96 (ddd, *J* = 8.5, 8.5, 2.5 Hz, H2, 1H), 6.52 (ddd, *J* = 8.7, 8.7, 2.9 Hz, H20 or H18, 1H), 6.44 (ddd, *J* = 10.1, 7.1, 2.6 Hz, H12, 1H), 6.22 (ddd, *J* = 8.2, 5.5, 2.4 Hz, H21 or H16, 1H), 3.11 (m, H11, 1H), 1.28 (s, H23, 15H). **^{13}C-NMR** (126 MHz, CD_2Cl_2): δ = 181.6 (s, C7), 165.6 (d, *J* = 251.3 Hz, C1), 160.9 (d, *J* = 244.7 Hz, C19), 159.6 (s, C4), 158.7 (d, *J* = 251.5 Hz, C13), 157.0 (d, *J* = 12.6 Hz, C5), 134.4 (d, *J* = 7.4 Hz, C17 or C21), 132.8 (s, C16), 130.8 (d, *J* = 12.3 Hz, C3), 129.5 (d, *J* = 8.3 Hz, C21 or C17), 125.1 (d, *J* = 8.6 Hz, C15), 119.7 (d, *J* = 28.0 Hz, C14), 116.9 (s, C9), 115.4 (d, *J* = 19.0 Hz, C12), 115.3 (d, *J* = 21.9 Hz, C18 or C20), 112.6 (d, *J* = 20.8 Hz, C18 or C20), 110.6 (d, *J* = 22.8 Hz, C2), 103.7 (d, *J* = 25.6 Hz, C6), 95.0 (d, *J* = 6.3 Hz, C22), 90.7 (s, C10), 64.3 (d, *J* = 7.1 Hz, C11), 8.35 (s, C23), C8 not observed. **^{19}F{H}-NMR** (471 MHz, CD_2Cl_2): δ = –106.6, –117.0, –118.1. **HR-MS** (ESI) *m/z* calcd for $C_{31}H_{27}F_3O_2Rh$ $[M+H]^+$: 591.1018, found: 591.1016.

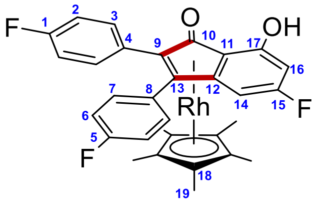

^{1}H-NMR (500 MHz, CD_2Cl_2): δ = 8.27 (s, ArO*H*, 1H), 7.40–7.45 (m, H3, 2H), 7.26–7.21 (m, H7, 2H), 7.13–7.07 (m, H6, 2H), 6.98–6.92 (m, H2, 2H), 6.25 (dd, *J* = 10.7, 1.8 Hz, H16, 1H), 6.13 (dd, *J* = 10.3, 1.8 Hz, H14, 1H), 1.45 (s, H19, 15H). **^{13}C-NMR** (126 MHz, CD_2Cl_2): δ = 165.8 (d, *J* = 246.2 Hz, C15), 162.7 (d, *J* = 247.2 Hz, C5), 161.8 (d, *J* = 246.4 Hz, C1), 160.0 (d, *J* = 16.4 Hz, C17), 150.4 (s, C10), 132.4 (d, *J* = 8.1 Hz, C7), 131.4 (d, *J* = 7.9 Hz, C3), 129.3 (d, *J* = 3.4 Hz, C8), 128.2 (d, *J* = 3.3 Hz, C4), 116.3 (d, *J* = 21.6 Hz, C6), 115.2 (d, *J* = 21.3 Hz, C2), 96.5 (dd, *J* = 14.3, 7.7 Hz, C12), 96.4 (d, *J* = 7.4 Hz, C18), 95.7 (d, *J* = 32.3 Hz, C16), 90.5 (d, *J* = 25.9 Hz, C14), 85.6 (dd, *J* = 9.8, 4.5 Hz, C13), 82.0 (d, *J* = 7.9 Hz, C9), 79.9 (d, *J*

= 7.3 Hz, C11), 8.12 (s, C19). **^{19}F{H}-NMR** (471 MHz, CD_2Cl_2): δ = –105.16, –114.48, –115.37. **HR-MS** (ESI) *m/z* calcd for $C_{31}H_{27}F_3O_2Rh$ $[M+H]^+$: 591.1018, found: 591.1017.

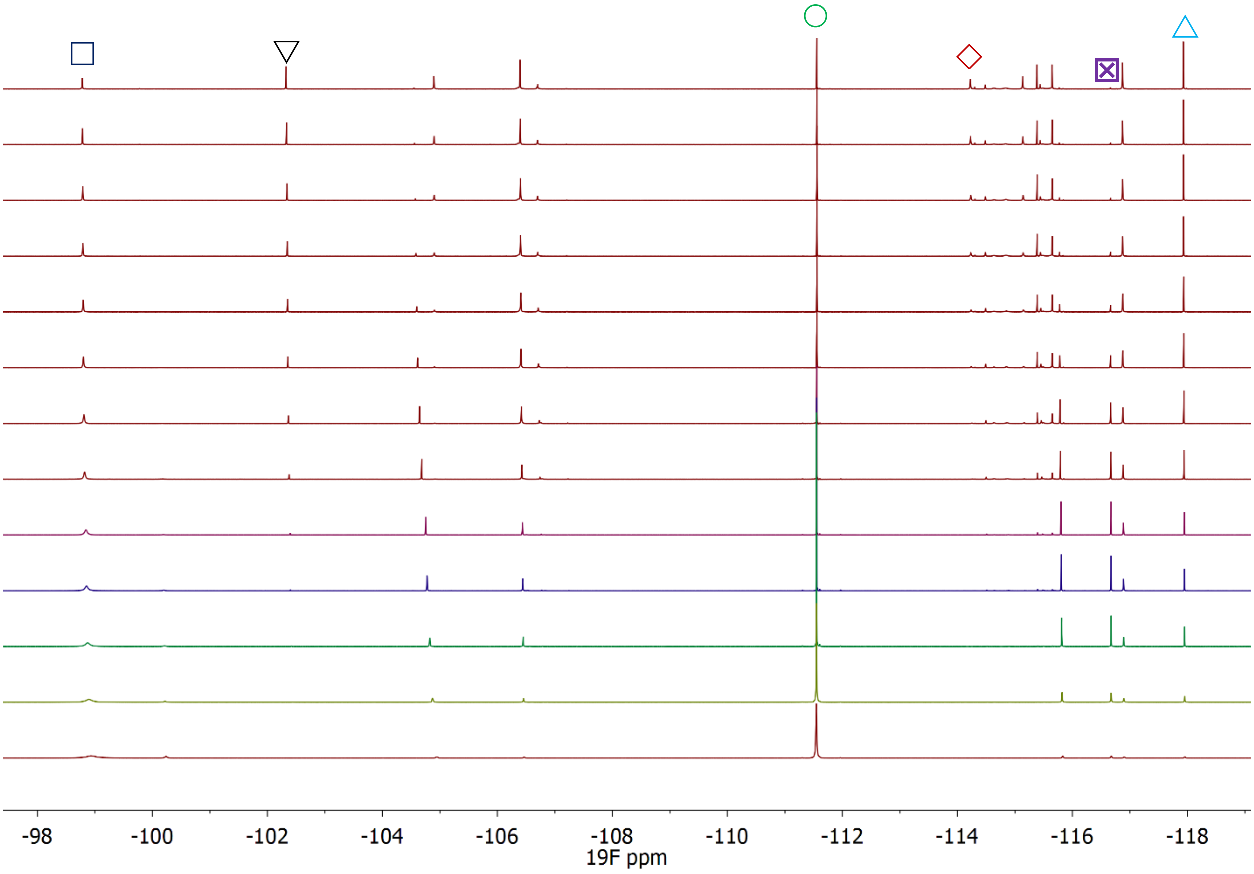

Supplementary Figure 1. ^{19}F{H}-NMR (376 MHz, CD_2Cl_2) spectra collected with a time interval of 3 min (only 13 of the 250 collected spectra are presented here for clarity) in the reaction between $[Cp^*RhCl_2]_2$, aldehyde **1**, and alkyne **2** in CD_2Cl_2 (500 µL) at 26 °C. Compound key: □ = aldehyde **1**, ○ = alkyne **2**, △ = **Rh-I**, ◇ = **Rh-II**, ▽ = **Rh-III** (tentatively assigned according to HESI MS and an X-ray crystallographic study of an isolated crystal), ⊠ = Tentatively assigned to rhodaheptacycle intermediate **C** of Supplementary Figure 12.

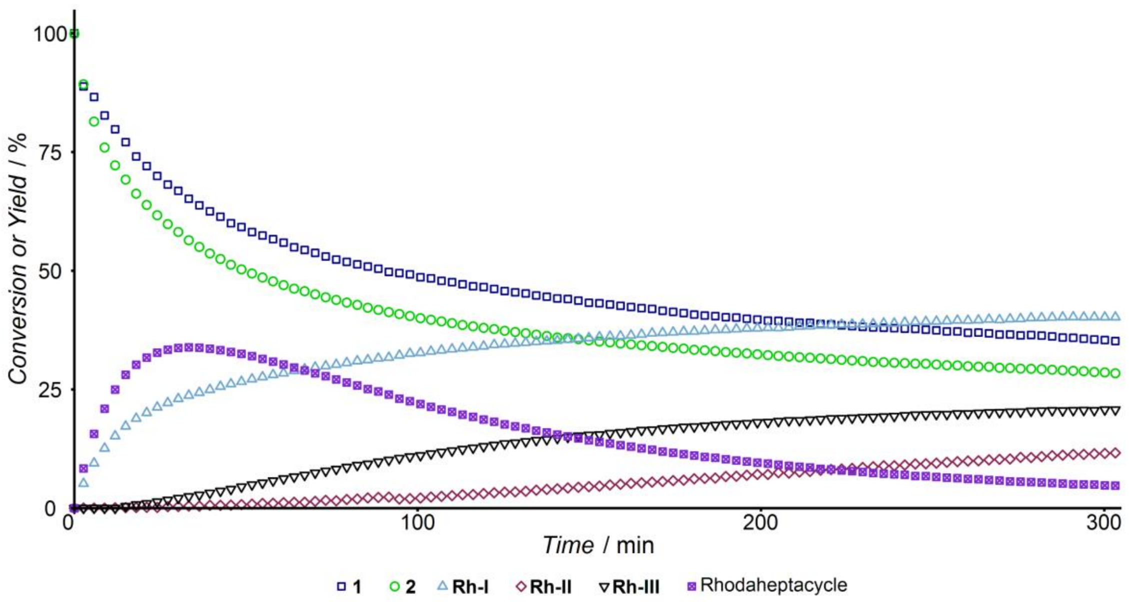

Supplementary Figure 2. ^{19}F{H}-NMR spectroscopic monitoring of the reaction between $[Cp^*RhCl_2]_2$ (25.0 µmol), aldehyde **1** (39.3 µmol), and alkyne **2** (50 µmol) at 25 °C in CD_2Cl_2 (500 µL). Yield was calculated based on the limiting reagent: aldehyde **1**.

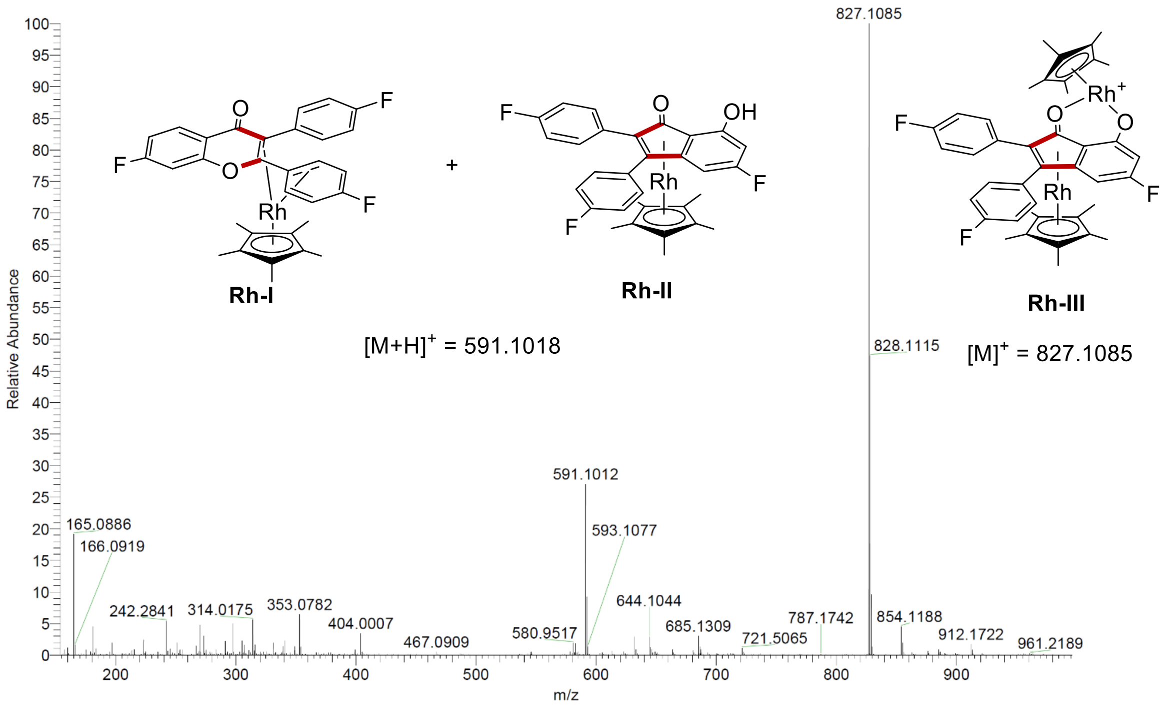

Supplementary Figure 3. High resolution HESI mass spectrum of the final filtrate from the reaction between $[Cp^*RhCl_2]_2$ (25.0 µmol), 4-fluoro-2-hydroxybenzaldehyde **1** (39.3 µmol), and 1,2-bis(4-fluorophenyl)ethyne **2** (50 µmol). Complex **Rh-III** is proposed to correspond to the peak observed at 827.1085 amu.

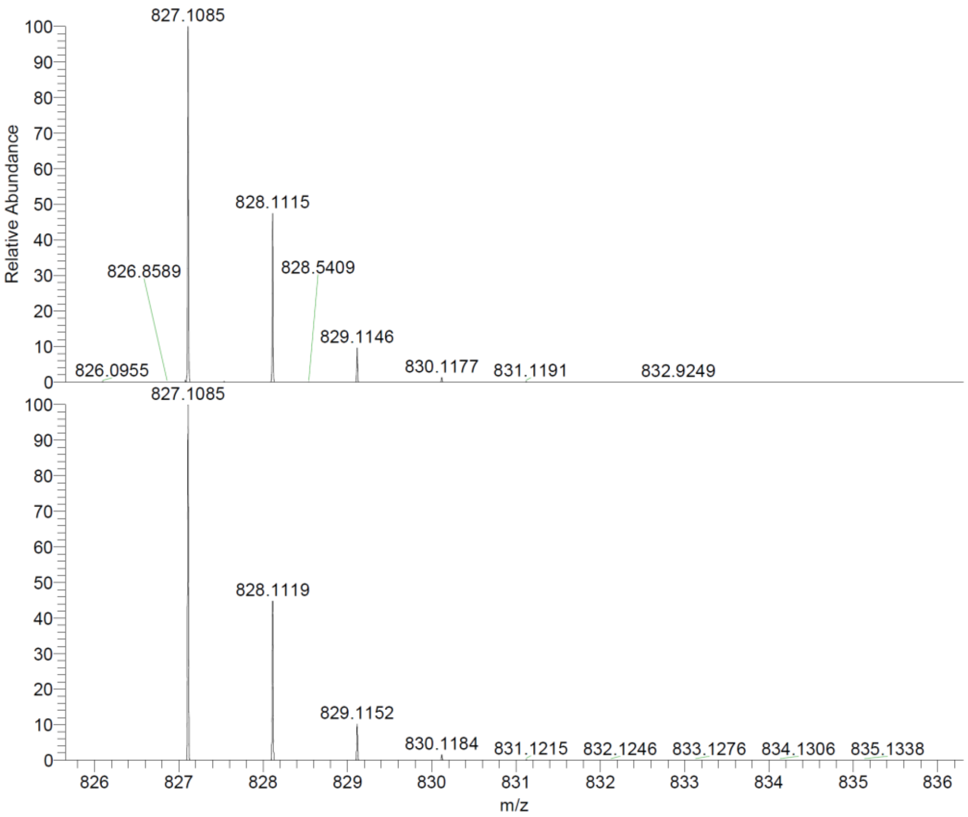

Supplementary Figure 4: Expansion of the peak at 827.1085 amu from Supplementary Figure 3 (top) and comparison with the simulated isotopic pattern resulting from a species with a molecular formula $C_{41}H_{40}F_3O_2Rh_2$ such as **Rh-III** (bottom).

Synthesis and characterization of Rh-IV

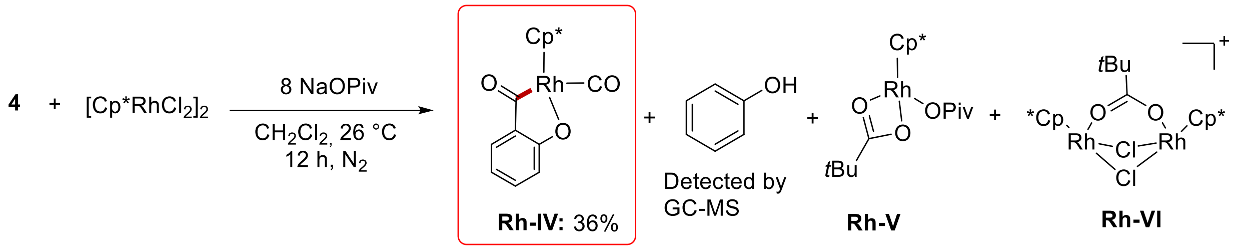

In a nitrogen filled glove box a 1.5 mL vial was loaded with $[Cp^*RhCl_2]_2$ (13.0 mg, 21 μmol) followed by addition of NaOPiv (20.8 mg, 0.168 mmol), and CH_2Cl_2 (0.5 mL). The resulted orange suspension was briefly sonicated (1 min) and refluxed (1 min). Subsequently, 2-hydroxybenzaldehyde (4.4 μL, 42 μmol) was added and the mixture was stirred at room temperature for 12 h. The orange-red solution was then filtered and evaporated leaving behind a viscous oil. Subsequently, the orange oil was dissolved in ether (0.5 mL) and stored at – 20 °C

for 12 hours resulting in orange crystals of **Rh-VI** which, after decanting the solution, were isolated and crystallographically characterized using single crystal X-ray diffraction. The filtrate was then condensed to half its volume and stored at – 20 °C for 12 hours resulting in the formation of **Rh-IV** in the form of red cauliflower like aggregates. The red solid of **Rh-IV** was isolated by decanting the solution and dried under reduced pressure (3.2 mg were collected). The filtrate was then dried resulting in an orange viscous oil which was dissolved in ether (0.25 mL) and layered with pentane (0.25 mL). This layered solution was then stored at – 20 °C for 12 hours resulting in the formation of a second crop of **Rh-IV** which was isolated by decanting the solution and combined with the first crop (combined isolated amount: 5.8 mg, 36% yield). The final filtrate was again dried and the orange oily residue was dissolved in a minimum amount of pentane. Upon standing, this pentane solution yielded orange needle crystals which were found to be **Rh-V** according to the single crystal X-ray crystallographic analysis that followed. Single crystals of **Rh-IV** suitable for a crystallographic analysis were grown out of a cold (– 20 °C) ether solution. **Rh-IV** was also fully spectroscopically characterized: **^{1}H-NMR** (400 MHz, CD_2Cl_2): δ = 7.24 (dd, *J* = 7.9, 1.7 Hz, H6, 1H), 7.12 (ddd, *J* = 8.5, 6.8, 1.7 Hz, C4, 1H), 6.84 (br d, *J* = 8.5 Hz, H3, 1H), 6.41 (ddd, *J* = 7.9, 6.8, 1.0 Hz, H5, 1H), 1.77 (s, H10, 15H). **^{13}C-NMR** (101 MHz, CD_2Cl_2): δ = 225.2 (d, *J* = 23.2 Hz, C8), 188.5 (d, *J* = 81.5 Hz, C1), 180.4 (s, C2), 136.4 (s, C3), 135.1 (s, C4), 124.0 (s, C6), 118.6 (d, *J* = 2.0 Hz, C7), 115.0 (s, C5), 105.3 (d, *J* = 4.5 Hz, C9), 9.42 (s, C10). **HR-MS** (ESI) *m/z* calcd for $C_{18}H_{19}O_3Rh$ $[M+H]^+$: 386.0389, found: 386.0395.

Synthesis and Isolation of Rh-VII

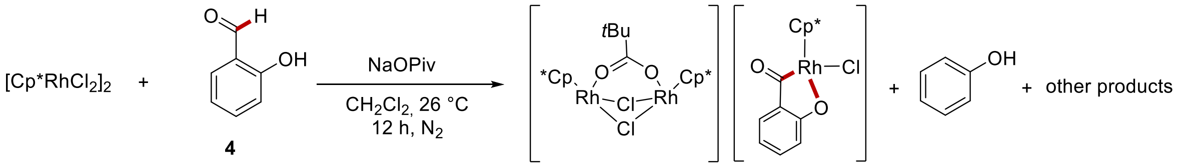

In a nitrogen filled glove box a vial was loaded with $[Cp^*RhCl_2]_2$ (13.0 mg, 21 μmol) followed by addition of NaOPiv (10.4 mg, 84 μmol) and CH_2Cl_2 (2 mL). The suspension was briefly sonicated (1 min) and refluxed (1 min). Subsequently, 2-hydroxybenzaldehyde (8.8 μL, 84 μmol) was added and the mixture was stirred at room temperature for 12 h. The mixture was then filtered, and the solution evaporated leaving behind a viscous oil which was dissolved in ether (0.5 mL). An orange precipitate immediately formed which was removed by filtration. The filtrate was condensed (0.5 mL) and stored at – 20 °C for 12 hours yielding crystals of **Rh-VII** suitable for an X-ray crystallographic analysis.

Synthesis and characterization of Rh-VIII

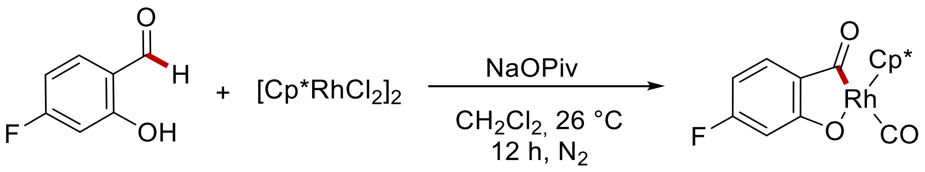

In a nitrogen filled glove box a vial was loaded with $[Cp^*RhCl_2]_2$ (13.0 mg, 21 μmol) followed by addition of NaOPiv (20.8 mg, 0.168 mmol) in CH_2Cl_2 (0.6 mL). The mixture was briefly sonicated (1 min) and heated at reflux (1 min) followed by addition of 4-fluoro-2-hydroxybenzaldehyde (17.7 mg, 0.126 mmol). The resulted mixture was left to react for 12 hours at room temperature filtered and dried under reduced pressure. The residue was dissolved in ether (0.2 mL), filtered and stored at – 20 °C for 24 hours during which time crystals suitable for an X-ray crystallographic analysis of **Rh-VIII** formed.

5. Cyclic voltammetry

Cyclic voltammetry measurements were conducted with a Metrohm Autolab PGSTAT204 potentiostat and Nova 2.1 software. For all experiments a glassy carbon working electrode (disk, diameter: 3 mm), a platinum wire counter electrode, and either a SCE or an Ag/Ag^{+} electrode was used as a quasi-reference electrode with ferrocene as an internal standard. Acetonitrile with 0.1 mol/L $n$$Bu_4NPF_6$ or DCM with 0.2 mol/L $n$$Bu_4NPF_6$ as conducting salt served as electrolyte for the measurements. The voltammograms were recorded at a scan rate of 100 mV/s, if not indicated otherwise.

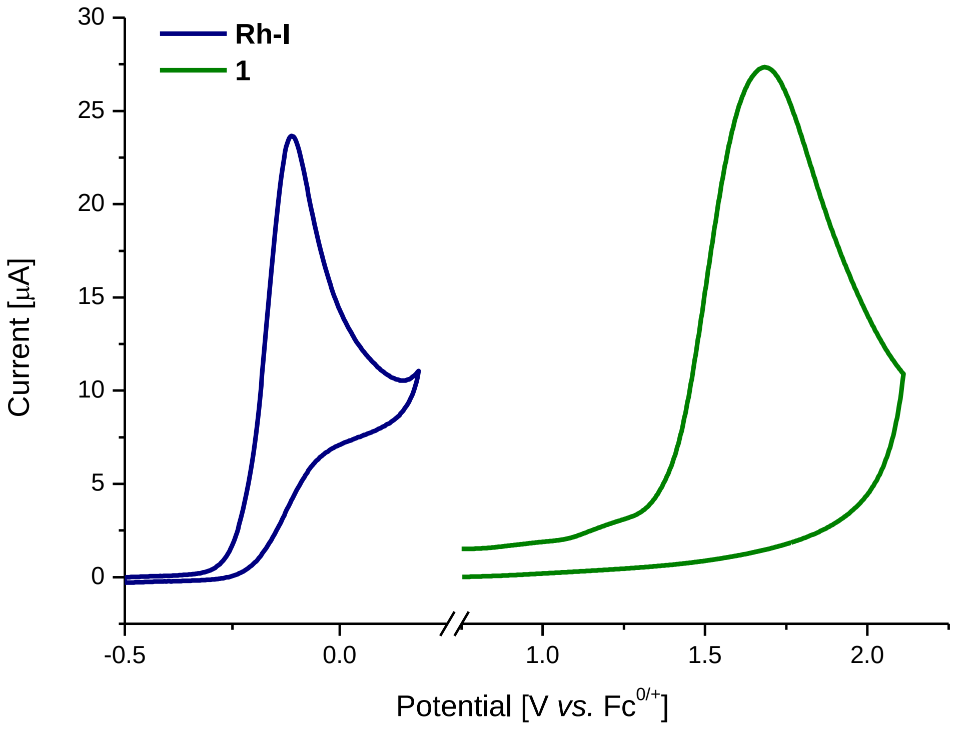

Supplementary Figure 5. Cyclic voltammetry of **Rh-I** (1.8 mM) at 100 mV/s and 4-fluorohydroxybenzaldehyde **1** (1.8 mM) at 5 mV/s in DCM with $n$$Bu_4NPF_6$ (0.2M).

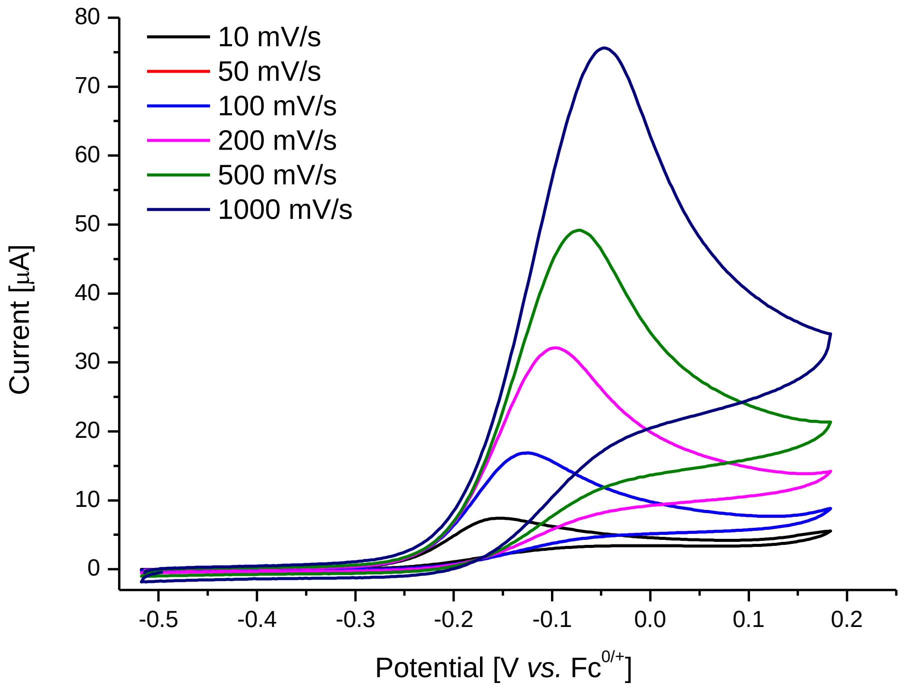

Supplementary Figure 6. Cyclic voltammetry of **Rh-I** (1.8 mM) at different scan rates in DCM with $n\mathrm{Bu_4NPF_6}$ (0.2M).

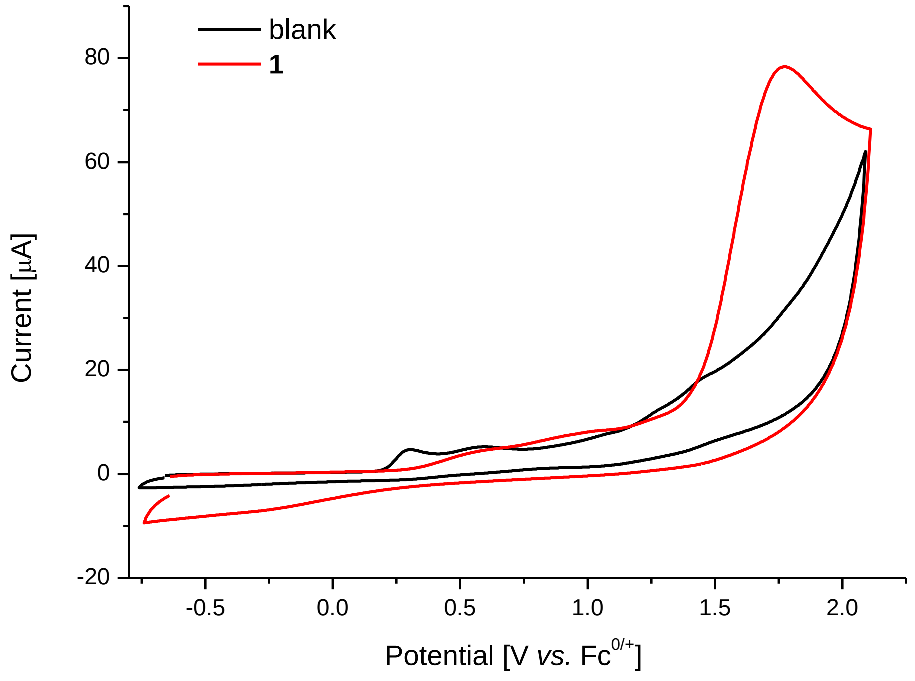

Supplementary Figure 7. Cyclic voltammetry of 4-fluorohydroxybenzalde **1** (1.8 mM) at 100 mV/s in DCM with $n\mathrm{Bu_4NPF_6}$ (0.2M).

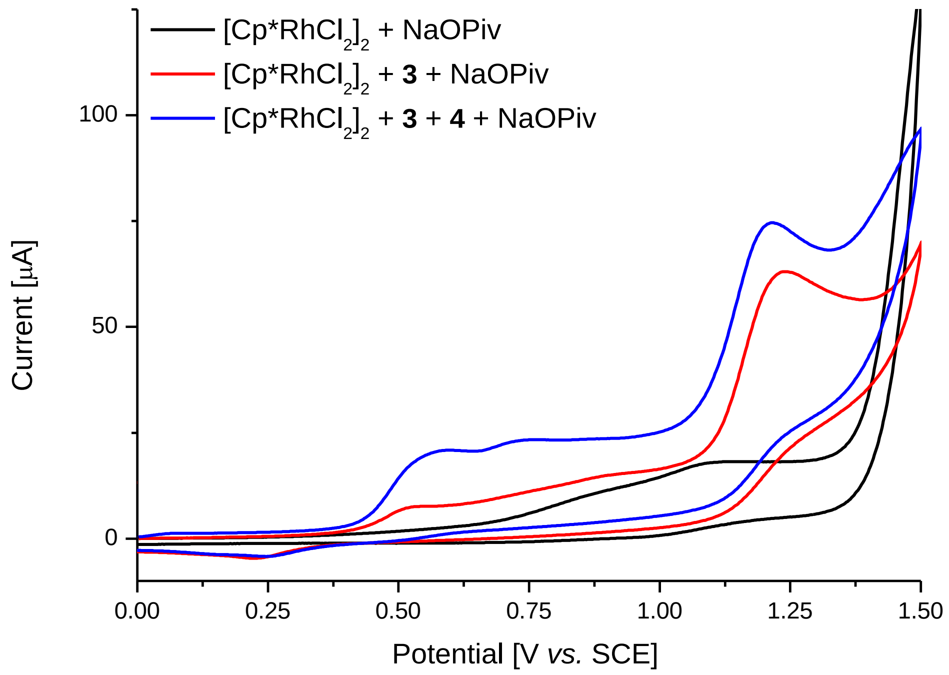

Supplementary Figure 8. Cyclic voltammetry of $[Cp^*RhCl_2]_2$ (5 mM) and NaOPiv (15 mM) with hydroxybenzalde **3** (5 mM), alkyne **4** (5 mM) and NaOPiv (15 mM) in MeCN with $n$$Bu_4NPF_6$ (0.1M) at 100 mV/s.

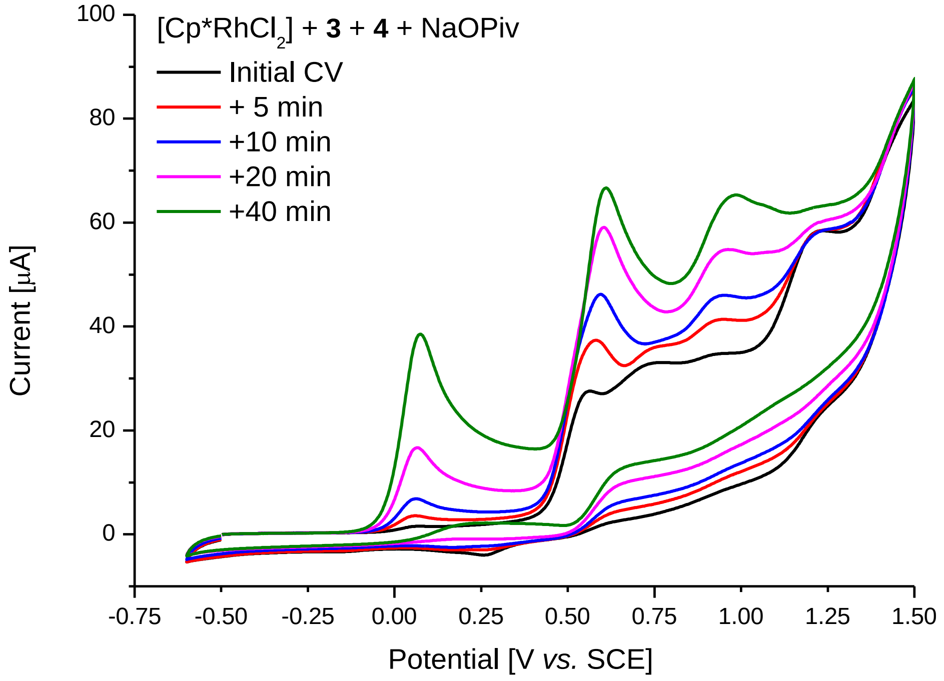

Supplementary Figure 9. Cyclic voltammetry of $[Cp^*RhCl_2]_2$ (5mM) with hydroxybenzalde **3** (5 mM), alkyne **4** (5 mM) NaOPiv (15 mM) in MeCN and NaOPiv (15 mM) in MeCN with $n$$Bu_4NPF_6$ (0.1M) at 100 mV/s.

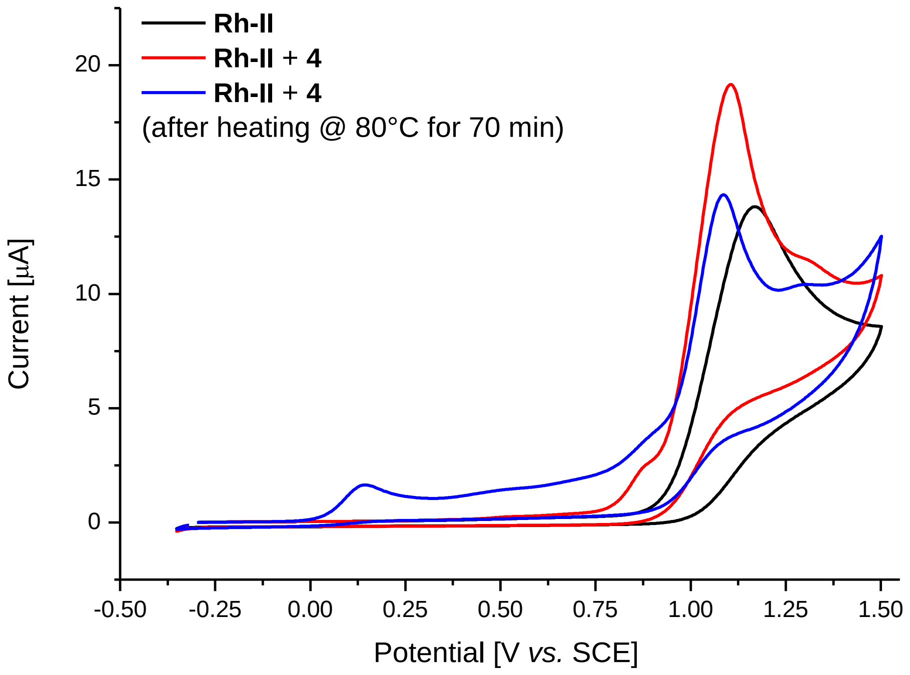

Supplementary Figure 10. Cyclic voltammetry of **Rh-II** (2.6 mM) with alkyne **4** (2.6 mM) in MeCN with $n\text{Bu}_4\text{NPF}_6$ (0.1M) at 100 mV/s.

6. On-Line NMR Monitoring in Flow

^{1}H-NMR spectroscopy experiments in flow were performed on a Magritek Spinsolve 60ULTRA (Magritek GmbH, Germany) with the reaction monitoring kit supplied by the manufacturer. For pumping the solution to the spectrometer, an Ismatec REGLO Digital MS-2/12 (ISM 596) peristaltic pump was employed. The flow rate was set to 0.4 mL/min.

A 10 mL-Schlenk tube was charged with aldehyde **3** (183 mg, 1.50 mmol), alkyne **4** (134 mg, 0.75 mmol), NaOPiv (186 mg, 1.50 mmol), $[\text{Cp*RhCl}_2]_2$ (11.6 mg, 2.5 mol %), trimethoxybenzene (42.0 mg, 0.25 mmol) and *t*AmylOH/H_2O (8 mL, 6.5:1.5). The electrocatalyses were performed with various constant currents. The yields were determined against the aromatic protons of the internal standard 1,3,5-trimethoxybenzene.

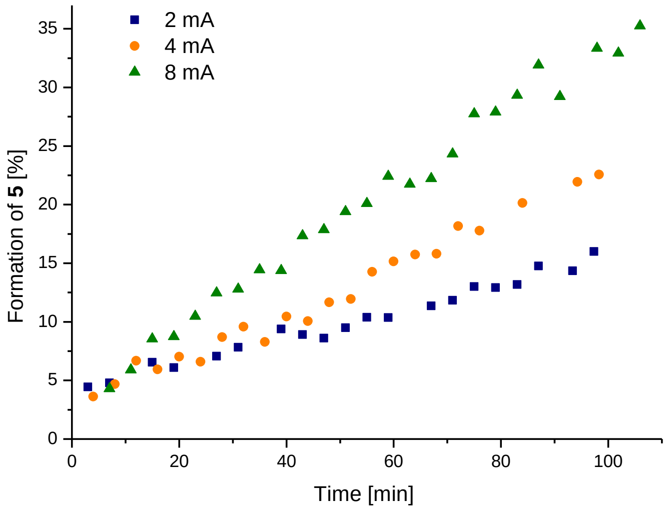

Supplementary Figure 11. On-line reaction monitoring in flow by ^{1}H-NMR spectroscopy at currents of 2 mA, 4 mA and 8 mA.

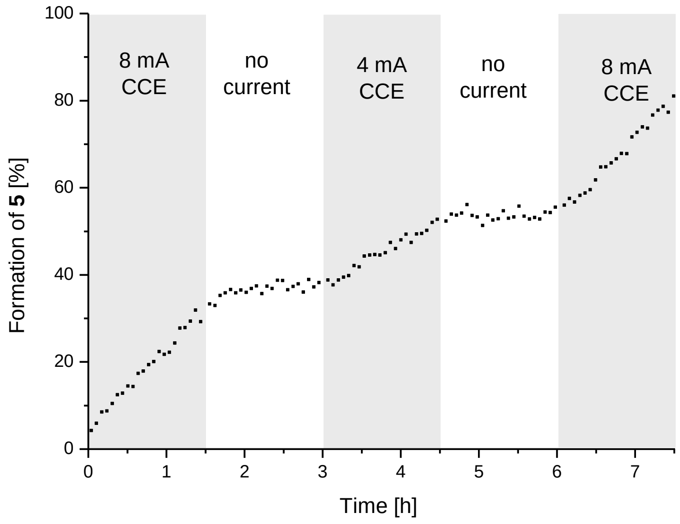

Supplementary Figure 12. On-line reaction monitoring in flow by ^{1}H-NMR spectroscopy with different currents and without applied current.

7. KIE studies

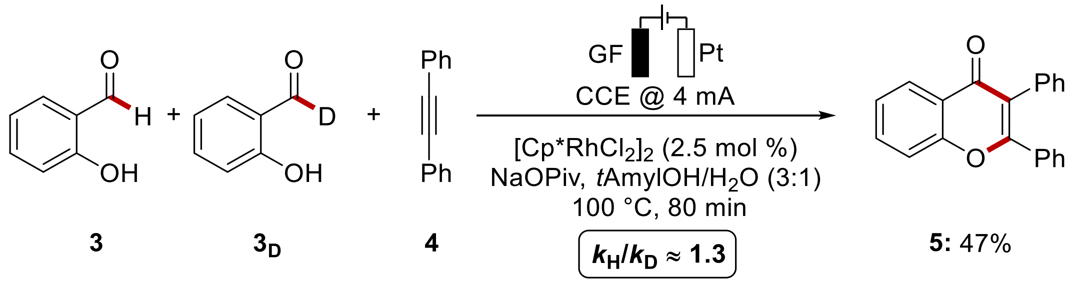

The electrocatalysis was carried out in an undivided cell, with a graphite felt (GF) anode (25 mm × 10 mm × 6.0 mm) and a platinum cathode (25 mm × 10 mm × 0.125 mm). 2-Hydroxybenzaldehyde **3** (45.8 mg, 0.375 mmol) and **3_D** (46.1 mg, 0.375 mmol), alkyne **4** (44.5 mg, 0.25 mmol), NaOPiv (62 mg, 0.50 mmol), $[Cp^*RhCl_2]_2$ (3.9 mg, 2.5 mol %) and *t*AmylOH/H_2O (4 mL, 3:1) were placed in a 10 mL cell. Electrocatalysis was performed at 100 °C with a constant current of 4 mA maintained for 80 min. Then, the DC-power supply was stopped and the reaction mixture was diluted with EtOAc (2.0 mL). The platinum cathode and the graphite felt anode were washed with EtOAc (Pt: 1 × 5 mL; C: 3 × 10.0 mL). The solvents were combined with the reaction mixture, silica gel was added and the solvents were removed in vacuo. Subsequent column chromatography on silica gel (*n*hexane/EtOAc = 25/1 to 10/1) afforded a mixture of the recovered starting materials **3** and **3_D** (34.9 mg) as well as product **5** (34.9 mg, 47%). A KIE value of $k_H/k_D \approx 1.3$ was determined by ^{1}H-NMR spectroscopic analysis of the mixture of **3** and **3_D**.

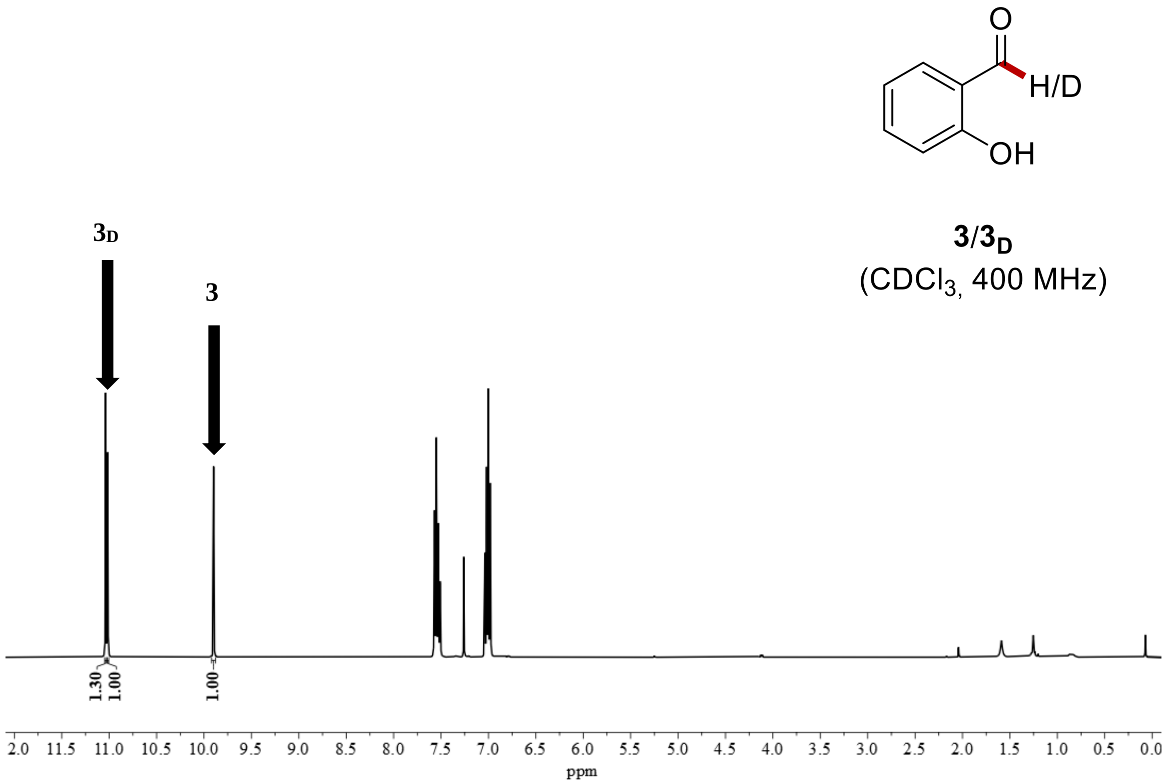

Supplementary Figure 13. ^{1}H-NMR spectrum of the of crude mixture after the competition experiment between starting materials **6** and **7**.

8. Competition experiment

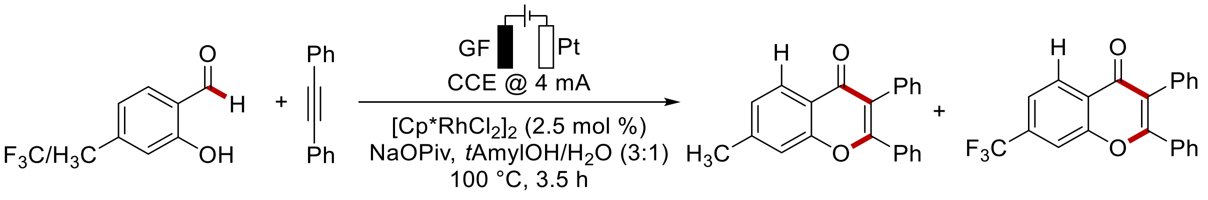

The electrocatalysis was carried out in an undivided cell, with a graphite felt (GF) anode (25 mm × 10 mm × 6.0 mm) and a platinum cathode (25 mm × 10 mm × 0.125 mm). 2-Hydroxybenzaldehydes **6** (71.3 mg, 0.375 mmol) and **7** (51.1 mg, 0.375 mmol), alkyne **4** (44.5 mg, 0.25 mmol), NaOPiv (62 mg, 0.50 mmol), $[Cp^*RhCl_2]_2$ (3.9 mg, 2.5 mol %) and *t*AmylOH/H_2O (4 mL, 3:1) were placed in a 10 mL cell. Electrocatalysis was performed at 100 °C with a constant current of 4 mA maintained for 3.5 h. Then, the DC-power supply was stopped and the reaction mixture was diluted with EtOAc (2.0 mL). The platinum cathode and the graphite felt anode were washed with EtOAc (Pt: 1 × 5 mL; C: 3 × 10.0 mL). The solvents were combined with the reaction mixture, and the solvents were removed in vacuo. $CDCl_3$ (1.2

mL) and internal standard CH_2Br_2 (36 μL, 0.5 mmol) were added and the mixture was filtered through a cotton plug and analyzed by ^1H-NMR spectroscopy ($CDCl_3$, 400 MHz).

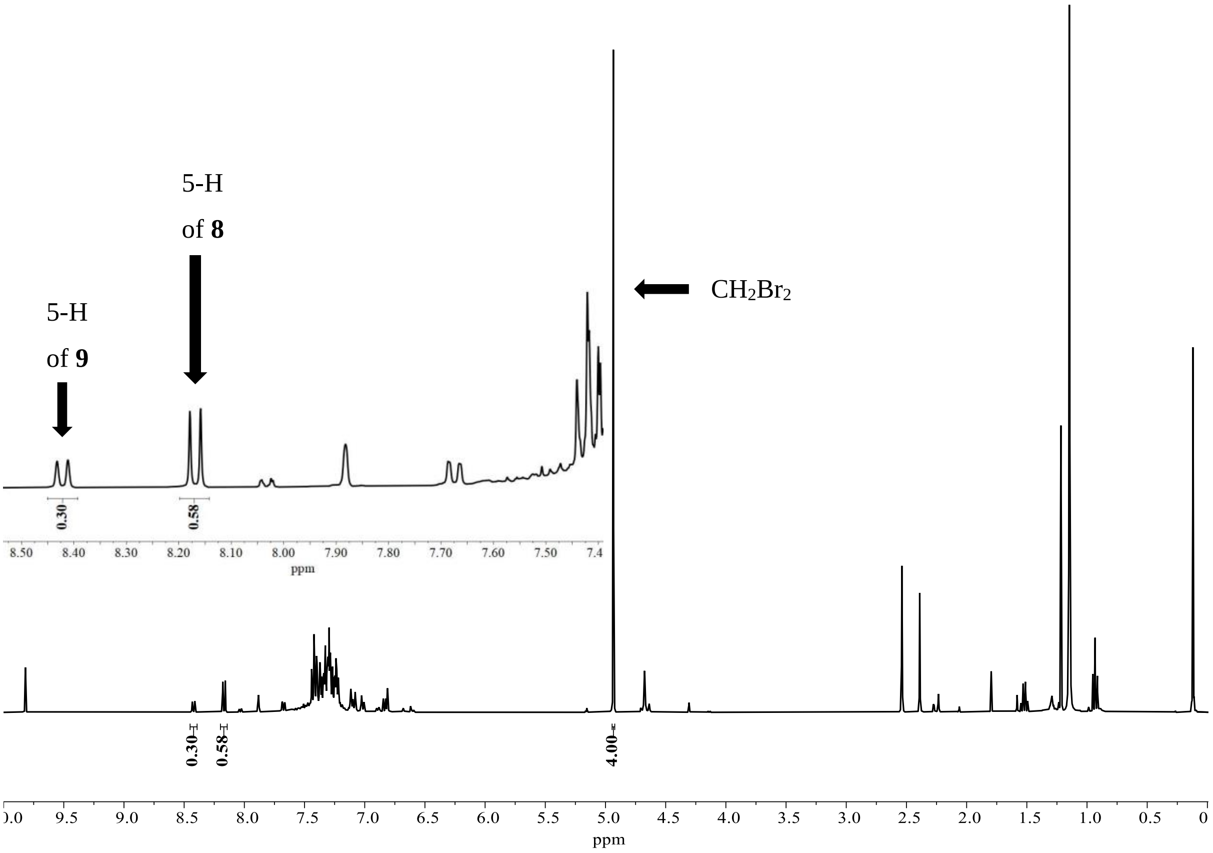

Supplementary Figure 14. ^1H-NMR spectrum of the of the isolated starting materials **3**/**3**$_\mathbf{D}$ after the reaction.

9. Multi-gram scale synthesis

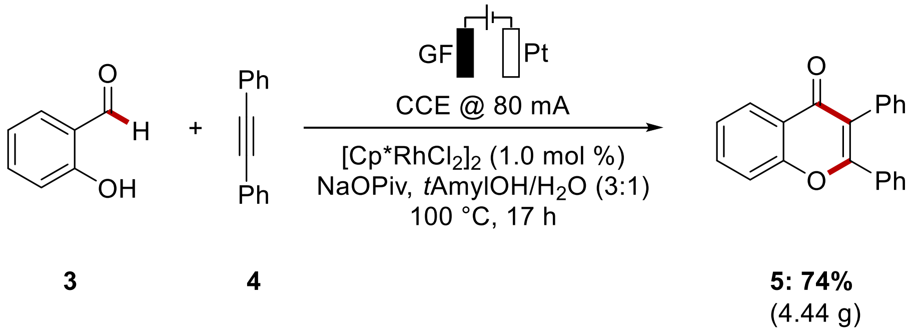

In an undivided cell with graphite felt anode (25 mm x 50 mm x 6 mm) and a platinum cathode (25 mm x 50 mm x 0.25 mm), aldehyde **3** (4.88 g, 40 mmol), alkyne **4** (3.44 g, 20 mmol), NaOPiv (4.96 g, 40 mmol), and $[Cp^*RhCl_2]_2$ (123.6 mg, 0.2 mmol) were dissolved in *t*AmOH/H_2O (60 mL, 3:1). Electrocatalysis was performed at 100 °C with a constant current of 80 mA maintained for 17 h. The graphite felt anode was washed with EtOAc (3 × 30 mL) in an ultrasonic bath. Evaporation of the solvent and subsequent column chromatography (*n*hexane/EtOAc = 10:1) yielded **5** (4.44g, 74%) as a white solid.

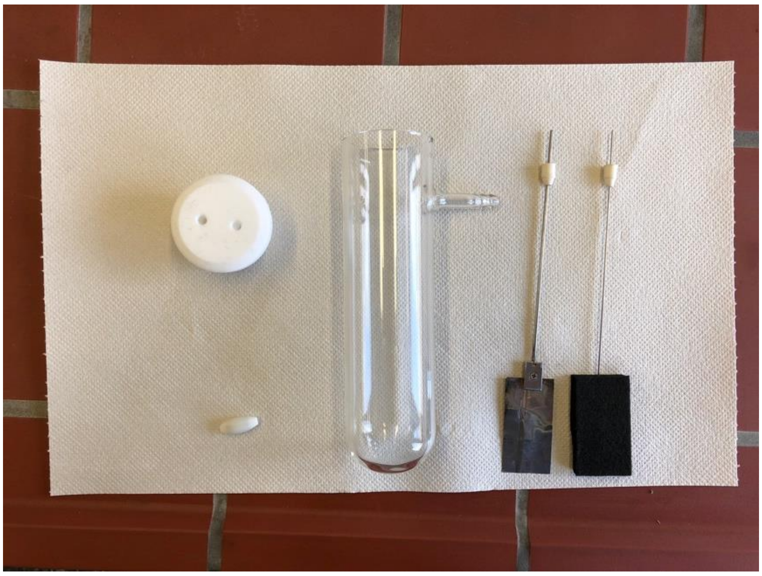

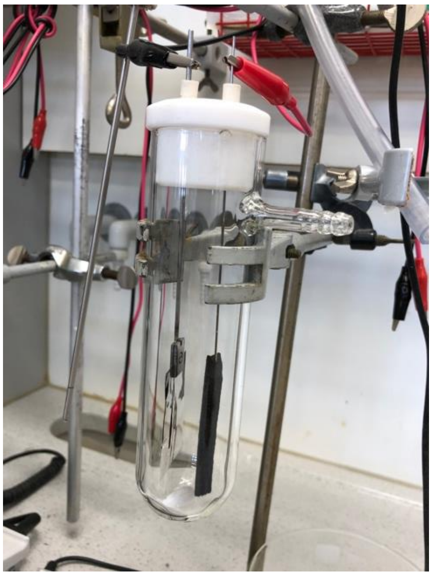

Supplementary Figure 15. Set-up for the multi-gram scale reaction.

10.UV/Vis and Fluorescence Measurements

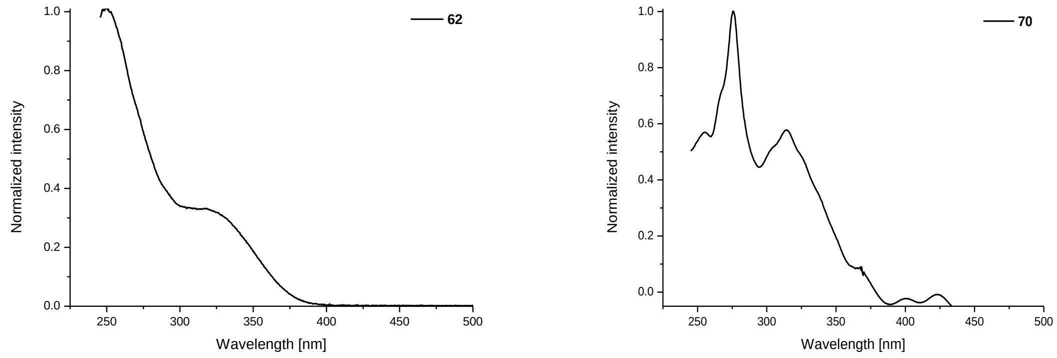

Supplementary Figure 16. Normalized absorption spectra of peptide **62** and **70** $CHCl_3$ (10 mg/L).

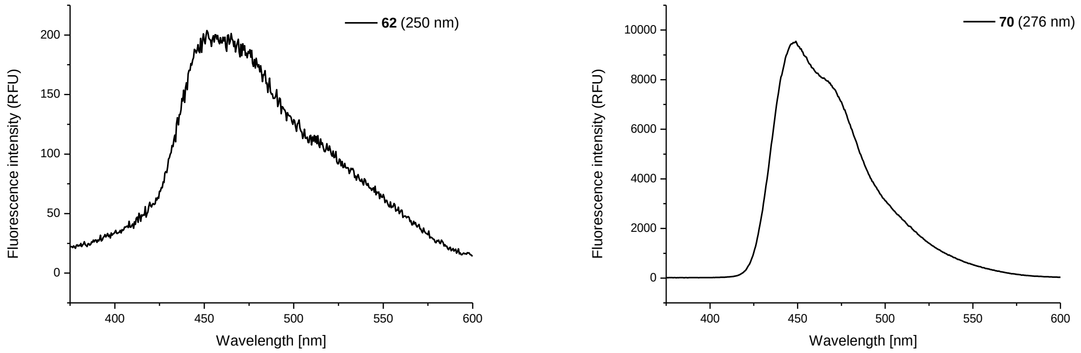

Supplementary Figure 17. Fluorescence spectra of peptide **62** (10 mg/L) and **70** (0.5 mg/L) in $CHCl_3$.

Supplementary Table 2. Absorption and emission properties of selected compounds measured in $CHCl_3$ at 10 mg/L. [a]Concentration 0.5 mg/L.

peptide	Absorption [nm]	Emission [nm]	Stokes shift [nm]
**60**	λ = 250, 351	λ (250) = 373	123
**61**	λ = 240, 294	λ (240) = 460	220
**62**	λ = 250, 318	λ (250) = 454	204
**69**	λ = 270, 280	λ (280) = 396	116
**70**[a]	λ = 276, 314	λ (276) = 448	172

11.Proposed Catalytic Cycle

A plausible mechanistic scenario for the formyl-electro-catalysis involves a facile formyl C–H scission, as suggested by the kinetic isotope effect, to furnish rhodacyle **A**. The structure of **A** is supported by the isolation of **Rh-II** from the reaction between **3** and $[Cp^*RhCl_2]_2$. After coordination of alkyne **4** to form complex **B** and subsequent insertion, formation of a seven-membered rhodacycle **C** is occuring. This rhodium(III) species then undergoes oxidatively-induced reductive elimination[14,15] to furnish the rhodium(II) complex **E**. Anodic oxidation of intermediate **E** completes the catalytic cycle to furnish the desired chromone and regenerate the active rhodium(III) catalyst.

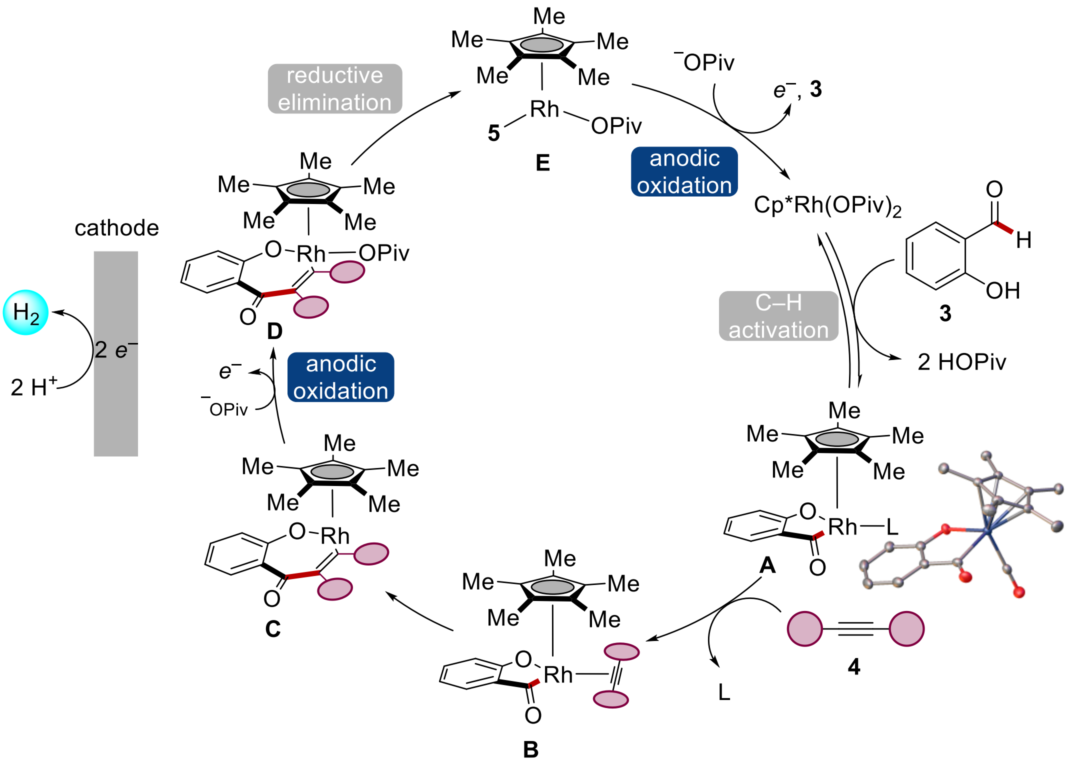

Supplementary Figure 18. Proposed mechanistic cycle of rhodaelectro-catalyzed formyl-C–H activation featuring oxidatively induced reductive elimination *via* Rh(III/IV/II).

12.X-Ray Crystallographic Analyses

X-ray diffraction experiments for all compounds were carried out at 100(2) K on a Bruker D8 Venture four-circle-diffractometer from Bruker AXS GmbH equipped with a Photon II detector purchased from Bruker AXS GmbH and using microfocus IµS Cu/Mo radiation from Incoatec GmbH with HELIOS mirror optics and single-hole collimator from Bruker AXS GmbH. Intensities were integrated[16] and absorption corrections based on equivalent reflections were applied using SADABS.[17] The structures were all solved using SHELXT[18] and refined against all F2 in SHELXL[19] using Olex 2.[20] All of the non-hydrogen atoms were refined anisotropically while the carbon bond hydrogen atoms were located geometrically and refined using a riding model. Molecular structures are presented with hydrogen atoms omitted for clarity and thermal ellipsoids drawn at the 50% probability level. Copies of the data can be obtained free of charge on application to CCDC, 12 Union Road, Cambridge CB2 1EZ, UK (fax +44 1223 336033, e-mail: deposit@ccdc.cam.ac.uk).

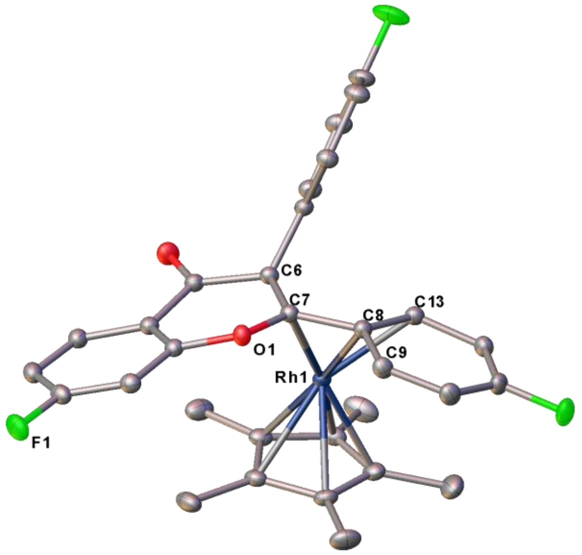

Rh-I

Identification code	CCDC 2046507
Empirical formula	$C_{31}H_{26}F_3O_2Rh$
Formula weight	590.43
Temperature/K	100.0
Crystal system	monoclinic
Space group	$P2_1/n$
a/Å	11.6380(7)
b/Å	13.2870(8)
c/Å	16.2715(10)
α/°	90
β/°	93.412(2)
γ/°	90
Volume/Å^3	2511.7(3)
Z	4
ρ_{calc}g/cm^3	1.561
μ/mm^{-1}	0.730
F(000)	1200.0
Crystal size/mm^3	0.371 × 0.318 × 0.105
Radiation	MoKα (λ = 0.71073)
2Θ range for data collection/°	3.96 to 61.158
Index ranges	$-16 \le h \le 16$, $-18 \le k \le 19$, $-23 \le l \le 23$
Reflections collected	111663
Independent reflections	7687 [R_{int} = 0.0237, R_{sigma} = 0.0110]
Data/restraints/parameters	7687/0/342
Goodness-of-fit on F^2	1.077
Final R indexes [I>=2σ (I)]	R_1 = 0.0199, wR_2 = 0.0527
Final R indexes [all data]	R_1 = 0.0204, wR_2 = 0.0530
Largest diff. peak/hole / e Å^{-3}	0.69/-0.68

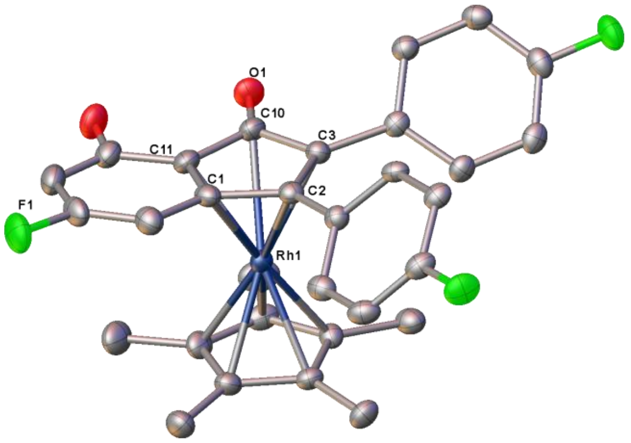

Rh-II

Identification code	CCDC 2046506
Empirical formula	$C_{31}H_{26}F_3O_2Rh$
Formula weight	590.43
Temperature/K	100.0
Crystal system	triclinic
Space group	P-1
a/Å	7.6158(10)
b/Å	16.235(2)
c/Å	19.6335(19)
α/°	95.524(4)
β/°	90.481(3)
γ/°	91.591(4)
Volume/Å^3	2415.1(5)
Z	4
ρ_{calc}g/cm^3	1.624
μ/mm^{-1}	0.759
F(000)	1200.0
Crystal size/mm^3	0.218 × 0.139 × 0.073
Radiation	MoKα (λ = 0.71073)
2Θ range for data collection/°	4.66 to 55.954
Index ranges	-10 ≤ h ≤ 10, -21 ≤ k ≤ 21, -25 ≤ l ≤ 25
Reflections collected	22582
Independent reflections	22582 [R_{int} = ?, R_{sigma} = 0.0362]
Data/restraints/parameters	22582/0/680
Goodness-of-fit on F^2	1.074
Final R indexes [I>=2σ (I)]	R_1 = 0.0413, wR_2 = 0.1008
Final R indexes [all data]	R_1 = 0.0479, wR_2 = 0.1052
Largest diff. peak/hole / e Å^{-3}	2.27/-0.99

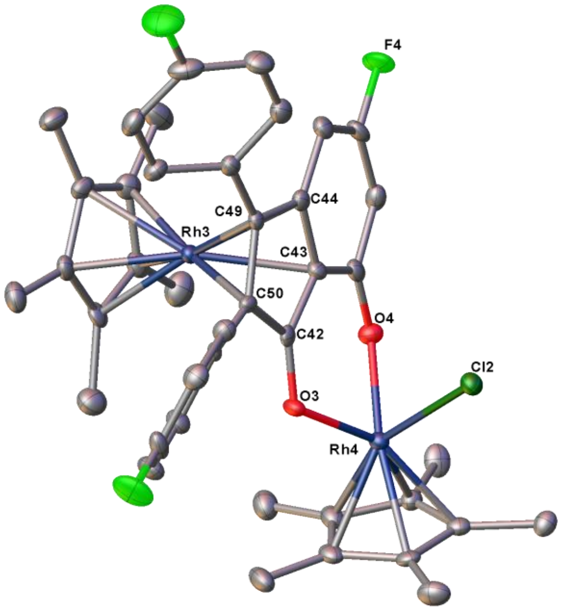

Rh-III

Identification code	CCDC 2046508
Empirical formula	$C_{88.42}Cl_{7.54}F_6H_{94.29}O_4Rh_4$
Formula weight	2013.90
Temperature/K	100.0
Crystal system	triclinic
Space group	P-1
a/Å	10.7660(6)
b/Å	15.0798(7)
c/Å	26.9458(15)
α/°	93.715(2)
β/°	96.373(2)
γ/°	98.979(2)
Volume/Å^3	4279.3(4)
Z	2
ρ_{calc}g/cm^3	1.563
μ/mm^{-1}	1.056
F(000)	2038.0
Crystal size/mm^3	0.453 × 0.24 × 0.11
Radiation	MoKα (λ = 0.71073)
2Θ range for data collection/°	4.272 to 54.424
Index ranges	-13 ≤ h ≤ 13, -19 ≤ k ≤ 19, -34 ≤ l ≤ 34
Reflections collected	224532
Independent reflections	19024 [R_{int} = 0.0505, R_{sigma} = 0.0240]
Data/restraints/parameters	19024/83/1042
Goodness-of-fit on F^2	1.209
Final R indexes [I>=2σ (I)]	R_1 = 0.0658, wR_2 = 0.1408
Final R indexes [all data]	R_1 = 0.0698, wR_2 = 0.1424
Largest diff. peak/hole / e Å^{-3}	2.24/-1.93

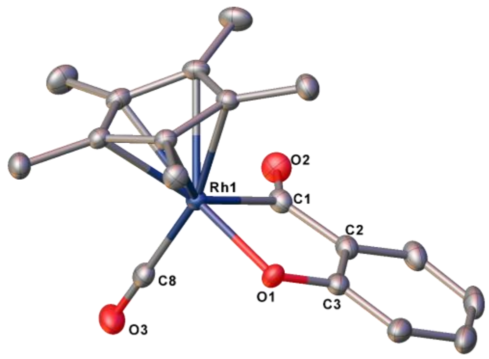

Rh-IV

Identification code	CCDC 2046502
Empirical formula	$C_{18}H_{19}O_3Rh$
Formula weight	386.24
Temperature/K	100.0
Crystal system	monoclinic
Space group	$P2_1/c$
a/Å	12.2323(5)
b/Å	9.6100(3)
c/Å	13.9529(6)
α/°	90
β/°	91.542(2)
γ/°	90
Volume/Å^3	1639.60(11)
Z	4
ρ_{calc}g/cm^3	1.565
μ/mm^{-1}	1.051
F(000)	784.0
Crystal size/mm^3	0.339 × 0.319 × 0.241
Radiation	MoKα (λ = 0.71073)
2Θ range for data collection/°	6.174 to 61.148
Index ranges	$-17 \leq h \leq 17$, $-13 \leq k \leq 12$, $-19 \leq l \leq 19$
Reflections collected	35678
Independent reflections	5014 [R_{int} = 0.0258, R_{sigma} = 0.0170]
Data/restraints/parameters	5014/0/204
Goodness-of-fit on F^2	1.066
Final R indexes [I>=2σ (I)]	R_1 = 0.0186, wR_2 = 0.0428
Final R indexes [all data]	R_1 = 0.0222, wR_2 = 0.0439
Largest diff. peak/hole / e Å^{-3}	0.43/-0.46

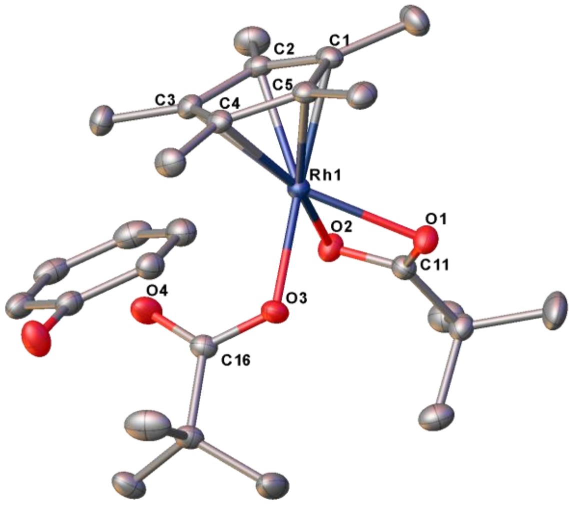

Rh-V

Identification code	CCDC 2046504
Empirical formula	$C_{84}H_{123}O_{16}Rh_3$
Formula weight	1697.55
Temperature/K	100.0
Crystal system	orthorhombic
Space group	$Pca2_1$
a/Å	21.7965(7)
b/Å	9.0386(3)
c/Å	42.3368(14)
α/°	90
β/°	90
γ/°	90
Volume/Å^3	8340.8(5)
Z	4
ρ_{calc}g/cm^3	1.352
μ/mm^{-1}	0.649
F(000)	3560.0
Crystal size/mm^3	0.513 × 0.134 × 0.134
Radiation	MoKα (λ = 0.71073)
2Θ range for data collection/°	4.204 to 59.186
Index ranges	$-30 \leq h \leq 30$, $-12 \leq k \leq 12$, $-58 \leq l \leq 58$
Reflections collected	375255
Independent reflections	23420 [R_{int} = 0.0369, R_{sigma} = 0.0138]
Data/restraints/parameters	23420/58/1022
Goodness-of-fit on F^2	1.047
Final R indexes [I>=2σ (I)]	R_1 = 0.0186, wR_2 = 0.0449
Final R indexes [all data]	R_1 = 0.0204, wR_2 = 0.0460
Largest diff. peak/hole / e Å^{-3}	0.29/-0.41
Flack parameter	0.288(11)

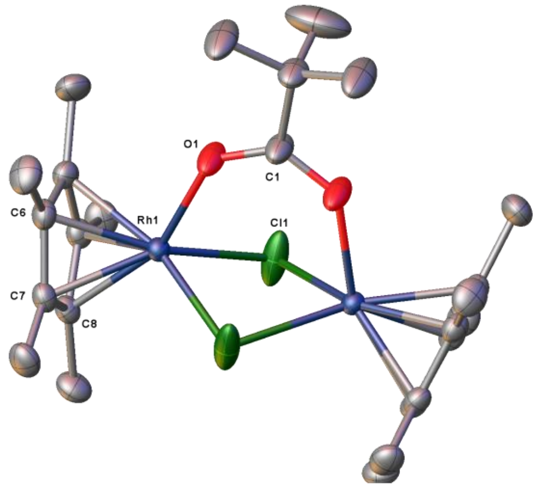

Rh-VI

Identification code	CCDC 2046503
Empirical formula	$C_{12.5}H_{19.5}ClORh$
Formula weight	324.14
Temperature/K	100.0
Crystal system	orthorhombic
Space group	Cmcm
a/Å	13.1757(6)
b/Å	25.8056(13)
c/Å	11.1388(4)
α/°	90
β/°	90
γ/°	90
Volume/Å^3	3787.3(3)
Z	8
ρ_{calc}g/cm^3	1.137
μ/mm^{-1}	1.026
F(000)	1316.0
Crystal size/mm^3	0.365 × 0.286 × 0.216
Radiation	MoKα (λ = 0.71073)
2Θ range for data collection/°	5.042 to 63.046
Index ranges	-19 ≤ h ≤ 19, -37 ≤ k ≤ 37, -16 ≤ l ≤ 16
Reflections collected	71410
Independent reflections	3427 [R_{int} = 0.0184, R_{sigma} = 0.0071]
Data/restraints/parameters	3427/0/92
Goodness-of-fit on F^2	1.083
Final R indexes [I>=2σ (I)]	R_1 = 0.0268, wR_2 = 0.0772
Final R indexes [all data]	R_1 = 0.0272, wR_2 = 0.0776
Largest diff. peak/hole / e Å^{-3}	0.80/-1.85

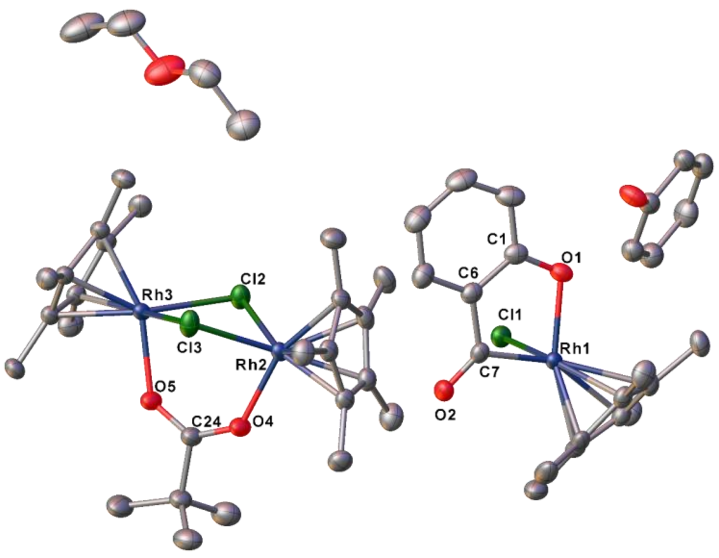

Rh-VII

Identification code	CCDC 2046505
Empirical formula	$C_{52}H_{74}Cl_3O_6Rh_3$
Formula weight	1210.19
Temperature/K	100.0
Crystal system	triclinic
Space group	P-1
a/Å	11.001(3)
b/Å	13.757(3)
c/Å	18.265(5)
α/°	77.975(7)
β/°	81.193(7)
γ/°	89.148(7)
Volume/Å^3	2671.2(12)
Z	2
ρ_{calc}g/cm^3	1.505
μ/mm^{-1}	1.111
F(000)	1240.0
Crystal size/mm^3	0.855 × 0.253 × 0.19
Radiation	MoKα (λ = 0.71073)
2Θ range for data collection/°	4.172 to 56.172
Index ranges	-14 ≤ h ≤ 14, -18 ≤ k ≤ 18, -24 ≤ l ≤ 24
Reflections collected	148778
Independent reflections	12870 [R_{int} = 0.0403, R_{sigma} = 0.0182]
Data/restraints/parameters	12870/0/598
Goodness-of-fit on F^2	1.073
Final R indexes [I>=2σ (I)]	R_1 = 0.0339, wR_2 = 0.0906
Final R indexes [all data]	R_1 = 0.0359, wR_2 = 0.0926
Largest diff. peak/hole / e Å^{-3}	2.67/-1.33

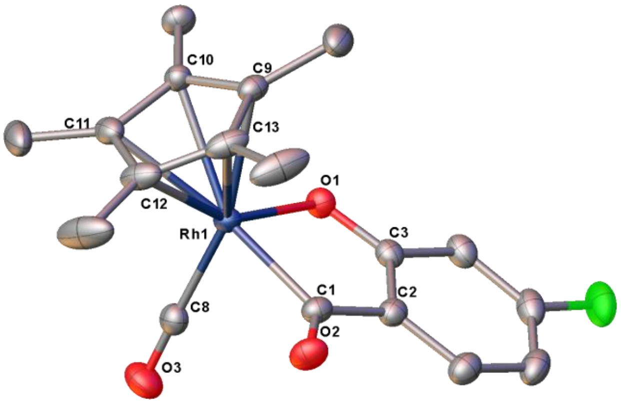

Rh-VIII

Identification code	CCDC 2046506
Empirical formula	$C_{18}H_{18}FO_3Rh$
Formula weight	404.23
Temperature/K	100.0
Crystal system	monoclinic
Space group	$P2_1/c$
a/Å	7.8161(6)
b/Å	13.5730(8)
c/Å	15.7267(11)
α/°	90
β/°	101.269(2)
γ/°	90
Volume/Å^3	1636.2(2)
Z	4
ρ_{calc}g/cm^3	1.641
μ/mm^{-1}	1.065
F(000)	816.0
Crystal size/mm^3	0.258 × 0.154 × 0.064
Radiation	MoKα (λ = 0.71073)
2Θ range for data collection/°	5.282 to 65.256
Index ranges	$-11 \leq h \leq 11$, $-20 \leq k \leq 20$, $-23 \leq l \leq 23$
Reflections collected	111546
Independent reflections	5972 [R_{int} = 0.0321, R_{sigma} = 0.0120]
Data/restraints/parameters	5972/0/214
Goodness-of-fit on F^2	1.073
Final R indexes [I>=2σ (I)]	R_1 = 0.0204, wR_2 = 0.0457
Final R indexes [all data]	R_1 = 0.0245, wR_2 = 0.0477
Largest diff. peak/hole / e Å^{-3}	0.60/-0.63

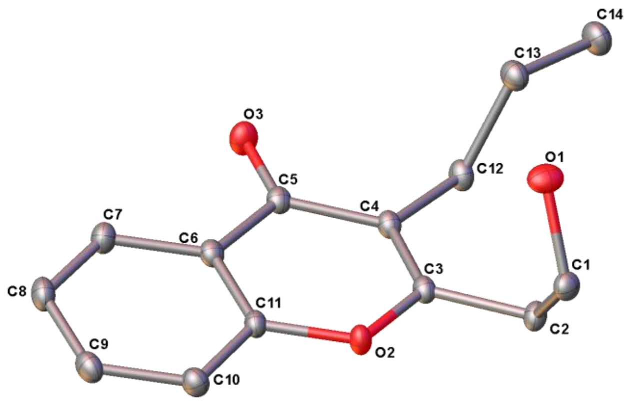

57

Identification code	CCDC 2046225
Empirical formula	$C_{14}H_{16}O_3$
Formula weight	232.27
Temperature/K	100.0
Crystal system	monoclinic
Space group	$P2_1/c$
a/Å	4.8492(3)
b/Å	15.5183(12)
c/Å	15.0910(11)
α/°	90
β/°	93.172(3)
γ/°	90
Volume/Å^3	1133.88(14)
Z	4
ρ_{calc}g/cm^3	1.361
μ/mm^{-1}	0.095
F(000)	496.0
Crystal size/mm^3	0.474 × 0.185 × 0.165
Radiation	MoKα (λ = 0.71073)
2Θ range for data collection/°	5.25 to 59.17
Index ranges	-6 ≤ h ≤ 6, -21 ≤ k ≤ 21, -20 ≤ l ≤ 20
Reflections collected	35861
Independent reflections	3134 [R_{int} = 0.0299, R_{sigma} = 0.0158]
Data/restraints/parameters	3134/0/156
Goodness-of-fit on F^2	1.070
Final R indexes [I>=2σ (I)]	R_1 = 0.0391, wR_2 = 0.1001
Final R indexes [all data]	R_1 = 0.0414, wR_2 = 0.1019
Largest diff. peak/hole / e Å^{-3}	0.43/-0.23

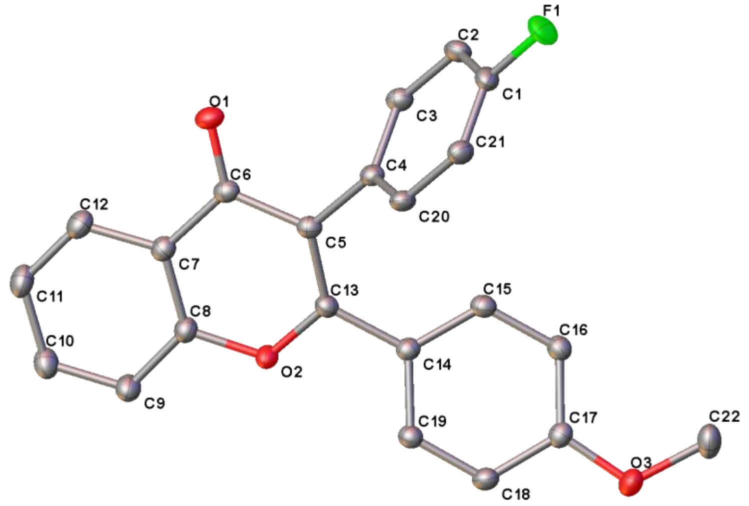

47

Identification code	CCDC 2046228
Empirical formula	$C_{22}H_{15}FO_3$
Formula weight	346.34
Temperature/K	100.0
Crystal system	monoclinic
Space group	$P2_1/c$
a/Å	5.7254(5)
b/Å	15.2781(13)
c/Å	19.087(2)
α/°	90
β/°	98.301(3)
γ/°	90
Volume/Å^3	1652.1(3)
Z	4
ρ_{calc}g/cm^3	1.392
μ/mm^{-1}	0.100
F(000)	720.0
Crystal size/mm^3	0.202 × 0.179 × 0.116
Radiation	MoKα (λ = 0.71073)
2Θ range for data collection/°	5.332 to 59.182
Index ranges	$-7 \leq h \leq 7$, $-21 \leq k \leq 21$, $-25 \leq l \leq 26$
Reflections collected	48386
Independent reflections	4561 [R_{int} = 0.0308, R_{sigma} = 0.0168]
Data/restraints/parameters	4561/0/236
Goodness-of-fit on F^2	1.068
Final R indexes [I>=2σ (I)]	R_1 = 0.0395, wR_2 = 0.0942
Final R indexes [all data]	R_1 = 0.0467, wR_2 = 0.1007
Largest diff. peak/hole / e Å^{-3}	0.36/-0.22

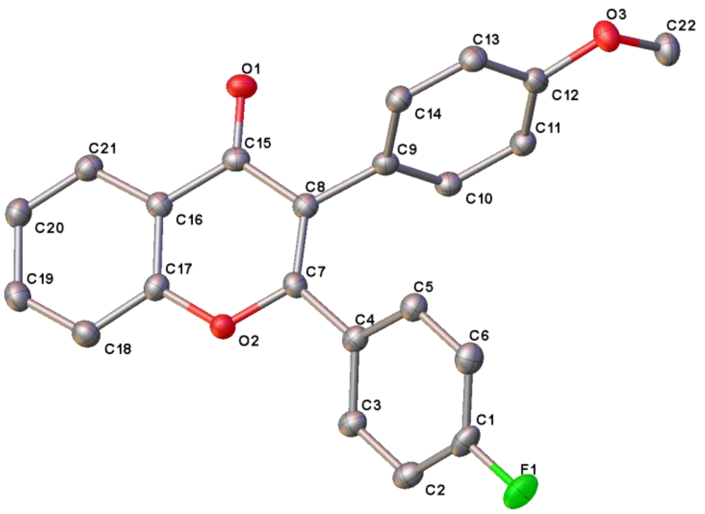

47`

Identification code	CCDC 2046229
Empirical formula	$C_{22}H_{15}FO_3$
Formula weight	346.34
Temperature/K	100.0
Crystal system	monoclinic
Space group	$P2_1/c$
a/Å	5.5391(2)
b/Å	13.8898(6)
c/Å	21.8396(9)
α/°	90
β/°	93.3070(10)
γ/°	90
Volume/Å^3	1677.48(12)
Z	4
ρ_{calc}g/cm^3	1.371
μ/mm^{-1}	0.098
F(000)	720.0
Crystal size/mm^3	0.3 × 0.085 × 0.054
Radiation	MoKα (λ = 0.71073)
2Θ range for data collection/°	4.75 to 57.408
Index ranges	$-7 \le h \le 7$, $-18 \le k \le 18$, $-28 \le l \le 29$
Reflections collected	34891
Independent reflections	4333 [R_{int} = 0.0474, R_{sigma} = 0.0293]
Data/restraints/parameters	4333/0/237
Goodness-of-fit on F^2	1.138
Final R indexes [I>=2σ (I)]	R_1 = 0.0547, wR_2 = 0.1114
Final R indexes [all data]	R_1 = 0.0711, wR_2 = 0.1196
Largest diff. peak/hole / e Å^{-3}	0.26/-0.26

13. Experimental and Analytical Data for Products

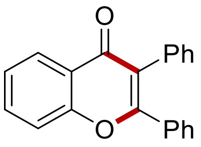

5

2,3-Diphenyl-4*H*-chromen-4-one

The general procedure A was followed using **3** (78 µL, 0.75 mmol) and **4** (44.5 mg, 0.25 mmol) at 100 °C for 3.5 h. Purification by column chromatography on silica gel (*n*hexane/EtOAc = 12/1 to 10/1) yielded **5** (68.2 mg, 92%) as a white solid.

^{1}H-NMR (400 MHz, $CDCl_3$): δ = 8.33 (d, J = 8.8 Hz, 1H), 7.73 (dd, J = 7.5 Hz, 7.5 Hz, 1H), 7.57 (d, J = 8.4 Hz, 1H), 7.49–7.41 (m, 3H), 7.39–7.23 (m, 8H). **^{13}C-NMR** (101 MHz, $CDCl_3$): δ = 177.4 (C_q), 161.6 (C_q), 156.2 (C_q), 133.8 (CH), 133.4 (C_q), 133.0 (C_q), 131.3 (CH), 130.2 (CH), 129.7 (CH), 128.3 (CH), 128.2 (CH), 127.7 (CH), 126.5 (CH), 125.2 (CH), 123.6 (C_q), 123.0 (C_q), 118.1 (CH). **IR** (ATR): $\tilde{\nu}$ = 1633, 1607, 1559, 1373, 1294, 1222, 915, 756, 695 cm^{-1}. **M.p.**: 143–144 °C. **MS** (ESI) *m/z* (relative intensity): 299 (100) $[M+H]^+$, 321 (40) $[M+Na]^+$, 619 (30) $[2M+Na]^+$. **HR-MS** (ESI) *m/z* calcd for $C_{21}H_{15}O_2$ $[M+H]^+$: 299.1067, found: 299.1054.

The spectral data are in accordance with those reported in the literature.[21]

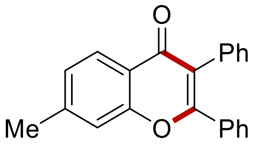

8

7-Methyl-2,3-diphenyl-4*H*-chromen-4-one

The general procedure A was followed using **7** (102 mg, 0.75 mmol) and **4** (44.5 mg, 0.25 mmol) at 100 °C for 3.5 h. Purification by column chromatography on silica gel (*n*hexane/EtOAc = 10/1) yielded **8** (72.1 mg, 92%) as a white solid.

^{1}H-NMR (400 MHz, $CDCl_3$): δ = 8.08 (d, J = 8.1 Hz, 1H), 7.31–7.27 (m, 2H), 7.25–7.19 (m, 3H), 7.19–7.11 (m, 7H), 2.41 (s, 3H). **^{13}C-NMR** (101 MHz, $CDCl_3$): δ = 177.3 (C_q), 161.2 (C_q), 156.3 (C_q), 145.0 (C_q), 133.5 (C_q), 133.1 (C_q), 131.3 (CH), 130.0 (CH), 129.6 (CH), 128.3 (CH), 128.1 (CH), 127.6 (CH), 126.7 (CH), 126.2 (CH), 122.9 (CH), 121.4 (C_q), 117.8 (C_q), 22.0 (CH_3). **IR** (ATR): $\tilde{\nu}$ = 1625, 1555, 1420, 1368, 1222, 1167, 776, 745, 694, 618 cm^{-1}. **M.p.**:

175–176 °C. **MS** (ESI) *m/z* (relative intensity): 313 (30) $[M+H]^+$, 335 (100) $[M+Na]^+$, 647 (60) $[2M+Na]^+$. **HR-MS** (ESI) *m/z* calcd for $C_{22}H_{17}O_2$ $[M+H]^+$: 313.1223, found: 313.1226.

The spectral data are in accordance with those reported in the literature.[22]

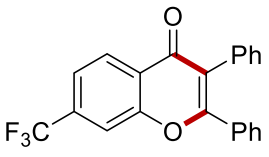

9

2,3-Diphenyl-7-(trifluoromethyl)-4*H*-chromen-4-one

The general procedure A was followed using **6** (143 mg, 0.75 mmol) and **4** (44.5 mg, 0.25 mmol) at 100 °C for 3.5 h. Purification by column chromatography on silica gel (*n*hexane/EtOAc = 15/1) yielded **9** (63.5 mg, 69%) as a white solid.

^{1}H-NMR (400 MHz, $CDCl_3$): δ = 8.33 (d, J = 8.3 Hz, 1H), 7.77 (d, J = 1.5 Hz, 1H), 7.58 (dd, J = 8.3, 1.5 Hz, 1H), 7.35–7.30 (m, 2H), 7.29–7.17 (m, 6H), 7.16–7.12 (m, 2H). **^{13}C-NMR** (101 MHz, $CDCl_3$): δ = 176.6 (C_q), 162.3 (C_q), 155.6 (C_q), 135.4 (q, $^2J_{C–F}$ = 33.3 Hz, C_q), 132.8 (C_q), 132.3 (C_q), 131.2 (CH), 130.6 (CH), 129.7 (CH), 128.5 (CH), 128.3 (CH), 128.1 (CH), 127.9 (CH), 125.8 (C_q), 123.7 (C_q), 123.3 (q, $^1J_{C–F}$ = 273.1 Hz, C_q), 121.5 (q, $^3J_{C–F}$ = 3.4 Hz, CH), 116.1 (q, $^3J_{C–F}$ = 4.1 Hz, CH). **^{19}F-NMR** δ = (377 MHz, $CDCl_3$) δ –63.02. **IR** (ATR): $\tilde{\nu}$ = 1633, 1550, 1434, 1349, 1312, 1114, 1039, 915, 748, 691 cm^{-1}. **M.p.**: 137–139 °C. **MS** (ESI) *m/z* (relative intensity): 367 (40) $[M+H]^+$, 389 (100) $[M+Na]^+$, 405 (20) $[M+K]^+$. **HR-MS** (ESI) *m/z* calcd for $C_{22}H_{14}F_3O_2$ $[M+H]^+$: 367.0940, found: 367.0943.

The spectral data are in accordance with those reported in the literature.[22]

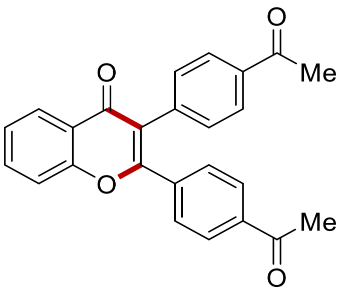

12

2,3-Bis(4-acetophenon)-4*H*-chromen-4-one

The general procedure A was followed using **3** (78 µL, 0.75 mmol) and **10a** (65.5 mg, 0.25 mmol) at 100 °C for 7 h. Purification by column chromatography on silica gel (*n*hexane/EtOAc = 2/1) yielded **12** (57.0 mg, 60%) as a brown solid.

The general procedure C was followed using **3** (78 µL, 0.75 mmol) and **10a** (65.5 mg, 0.25 mmol) at 100 °C for 7 h. Purification by column chromatography on silica gel (*n*hexane/EtOAc = 4/1 to 3/2) yielded **12** (32.6 mg, 34%) as a brown solid among other side products.

^{1}H-NMR (300 MHz, $CDCl_3$): δ = 8.28 (d, J = 7.8 Hz, 1H), 7.89 (d, J = 8.3 Hz, 2H), 7.85 (d, J = 8.4 Hz, 2H), 7.74 (dd, J = 7.8 Hz, 7.8 Hz, 1H), 7.55 (d, J = 8.4 Hz, 1H), 7.51–7.45 (m, 3H), 7.32 (d, J = 8.2 Hz, 2H), 2.58 (s, 3H), 2.57 (s, 3H). **^{13}C-NMR** (75 MHz, $CDCl_3$): δ = 197.9 (C_q), 197.2 (C_q), 176.8 (C_q), 160.6 (C_q), 156.1 (C_q), 138.1 (C_q), 137.8 (C_q), 137.2 (C_q), 136.4 (C_q), 134.3 (CH), 131.6 (CH), 129.9 (CH), 128.4 (CH), 128.2 (CH), 126.5 (CH), 125.7 (CH), 123.4 (C_q), 122.9 (C_q), 118.2 (CH), 26.8 (CH_3), 26.7 (CH_3). **IR** (ATR): $\tilde{\nu}$ = 2923, 2853, 1680, 1633, 1598, 1463, 1375, 1259, 1146, 956 cm^{-1}. **M.p.**: 165–166 °C. **MS** (ESI) *m/z* (relative intensity): 383 (100) $[M+H]^+$, 787 (50) $[2M+Na]^+$. **HR-MS** (ESI) *m/z* calcd for $C_{25}H_{19}O_4$ $[M+H]^+$: 383.1283, found: 383.1278.

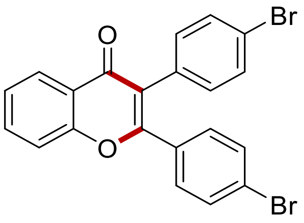

13

2,3-Bis(4-bromophenyl)-4*H*-chromen-4-one

The general procedure A was followed using **3** (78 µL, 0.75 mmol) and **10b** (84.0 mg, 0.25 mmol) at 100 °C for 7 h. Purification by column chromatography on silica gel (*n*hexane/EtOAc = 8/1) yielded **13** (79.6 mg, 69%) as a white solid.

The general procedure C was followed using **3** (78 µL, 0.75 mmol) and **10b** (84.0 mg, 0.25 mmol) at 100 °C for 7 h. Purification by column chromatography on silica gel (*n*hexane/EtOAc = 8/1) yielded **13** (44.5 mg, 39%).

^{1}H-NMR (400 MHz, $CDCl_3$): δ = 8.19 (dd, J = 8.0, 1.7 Hz, 1H), 7.64 (ddd, J = 8.7, 7.1, 1.7 Hz, 1H), 7.44 (dd, J = 8.5, 1.0 Hz, 1H), 7.40–7.34 (m, 5H), 7.18 (m, J = 8.5 Hz, 2H), 7.01 7.18 (m, J = 8.5 Hz, 2H). **^{13}C-NMR** (101 MHz, $CDCl_3$): δ = 176.9 (C_q), 160.5 (C_q), 156.0 (C_q), 134.1 (CH), 132.9 (CH), 131.9 (C_q), 131.8 (CH), 131.7 (CH), 131.6 (C_q), 131.2 (CH), 126.5 (CH), 125.5 (CH), 125.1 (C_q), 123.4 (C_q), 122.3 (C_q), 122.1 (C_q), 118.1 (CH). **IR** (ATR): $\tilde{\nu}$ = 1637, 1608, 1459, 1368, 1216, 1061, 1005, 814, 753, 495 cm^{-1}. **M.p.**: 184–186 °C. **MS** (ESI) *m/z* (relative intensity): 456 (100) $[M+H]^+$ ($^{79}Br^{81}Br$), 478 (40) $[M+Na]^+$ ($^{79}Br^{81}Br$), 934 (60)

[2M+Na]$^+$ (^{79}Br^{81}Br). **HR-MS** (ESI) *m/z* calcd for $C_{21}H_{13}{}^{79}Br^{81}BrO_2$ [M+H]$^+$: 456.9258, found: 456.9252.

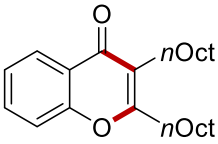

14

2,3-Dioctyl-4*H*-chromen-4-one

The general procedure A was followed using **3** (78 µL, 0.75 mmol) and **10c** (62.6 mg, 0.25 mmol) at 100 °C for 3.5 h. Purification by column chromatography on silica gel (*n*hexane/EtOAc = 40/1 to 20/1) yielded **14** (72.9 mg, 79%) as a colorless oil.

The general procedure C was followed using **3** (78 µL, 0.75 mmol) and **10c** (62.6 mg, 0.25 mmol) at 100 °C for 7 h. Purification by column chromatography on silica gel (*n*hexane/EtOAc = 40/1 to 20/1) yielded **14** (26.8 mg, 29%).

^{1}H-NMR (400 MHz, $CDCl_3$): δ = 8.18 (dd, J = 8.0, 1.7 Hz, 1H), 7.59 (ddd, J = 8.6, 7.0, 1.7 Hz, 1H), 7.39–7.29 (m, 2H), 2.73–2.64 (m, 2H), 2.57–2.47 (m, 2H), 1.74 (m, 2H), 1.53–1.45 (m, 2H), 1.42–1.20 (m, 20H), 0.87 (m, 6H). **^{13}C-NMR** (101 MHz, $CDCl_3$): δ = 178.0 (C_q), 165.7 (C_q), 156.0 (C_q), 132.9 (CH), 126.0 (CH), 124.4 (CH), 123.1 (C_q), 121.5 (C_q), 117.7 (CH), 32.0 (CH_2), 32.0 (CH_2), 32.0 (CH_2), 30.0 (CH_2), 29.6 (CH_2), 29.5 (CH_2), 29.5 (CH_2), 29.5 (CH_2), 29.4 (CH_2), 29.3 (CH_2), 27.7 (CH_2), 24.9 (CH_2), 22.8 (CH_2), 22.8 (CH_2), 14.2 (CH_3), 14.2 (CH_3). **IR** (ATR): $\tilde{\nu}$ = 2922, 2854, 1637, 1573, 1463, 1387, 1227, 1151, 759, 718 cm^{-1}. **MS** (ESI) *m/z* (relative intensity): 371 (100) [M+H]$^+$, 393 (20) [M+Na]$^+$, 741 (40) [2M+H]$^+$. **HR-MS** (ESI) *m/z* calcd for $C_{25}H_{39}O_2$ [M+H]$^+$: 371.2945, found: 371.2947.

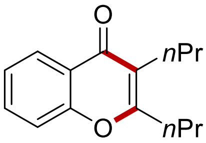

15

2,3-Dipropyl-4*H*-chromen-4-one

The general procedure A was followed using **3** (78 µL, 0.75 mmol) and **10d** (27.5 mg, 0.25 mmol) at 100 °C for 7 h. Purification by column chromatography on silica gel (*n*hexane/EtOAc = 25/1 to 15/1) yielded **15** (39.0 mg, 68%) as a colorless oil.

The general procedure C was followed using **3** (78 µL, 0.75 mmol) and **10d** (27.5 mg, 0.25 mmol) at 100 °C for 7 h. Purification by column chromatography on silica gel

(*n*hexane/EtOAc = 25/1 to 15/1) yielded **15** (17.3 mg, 30%) as a colorless oil among other side products.

^{1}H-NMR (400 MHz, $CDCl_3$): δ = 8.18 (dd, J = 7.9, 1.7 Hz, 1H), 7.59 (ddd, J = 8.7, 7.0, 1.7 Hz, 1H), 7.37 (dd, J = 8.7, 1.1 Hz, 1H), 7.33 (ddd, J = 8.1, 7.0, 1.1 Hz, 1H), 2.72–2.64 (m, 2H), 2.57–2.47 (m, 2H), 1.85–1.74 (m, 2H), 1.60–1.47 (m, 2H), 1.04 (t, J = 7.4 Hz, 3H), 0.98 (t, J = 7.4 Hz, 3H). **^{13}C-NMR** (101 MHz, $CDCl_3$): δ = 178.0 (C_q), 165.5 (C_q), 156.1 (C_q), 133.0 (CH), 126.0 (CH), 124.5 (CH), 123.1 (C_q), 121.4 (C_q), 117.7 (CH), 33.8 (CH_2), 26.8 (CH_2), 22.7 (CH_2), 21.1 (CH_2), 14.3 (CH_3), 14.0 (CH_3). **IR** (ATR): $\tilde{\nu}$ = 2961, 2930, 1632, 1573, 1462, 1385, 1338, 1229, 1160, 760 cm^{-1}. **MS** (ESI) *m/z* (relative intensity): 231 (100) $[M+H]^+$, 253 (40) $[M+Na]^+$, 483 (60) $[2M+Na]^+$. **HR-MS** (ESI) *m/z* calcd for $C_{15}H_{19}O_2$ $[M+H]^+$: 231.1380, found: 231.1380.
The spectral data are in accordance with those reported in the literature.[21]

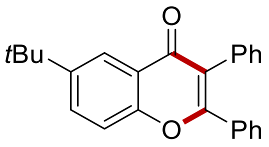

18

6-(*tert*-Butyl)-2,3-diphenyl-4*H*-chromen-4-one
The general procedure A was followed using **16a** (134 mg, 0.75 mmol) and **4** (44.5 mg, 0.25 mmol) at 100 °C for 3.5 h. Purification by column chromatography on silica gel (*n*hexane/EtOAc = 20/1) yielded **18** (65.2 mg, 74%) as a white solid.
^{1}H-NMR (400 MHz, $CDCl_3$): δ = 8.20 (dd, J = 2.5, 0.5 Hz, 1H), 7.67 (dd, J = 8.9, 2.5 Hz, 1H), 7.39 (dd, J = 8.9, 0.5 Hz, 1H), 7.32–7.28 (m, 2H), 7.26–7.11 (m, 8H), 1.31 (s, 9H). **^{13}C-NMR** (101 MHz, $CDCl_3$): δ =177.7 (C_q), 161.4 (C_q), 154.4 (C_q), 148.5 (C_q), 133.5 (C_q), 133.2 (C_q), 131.7 (CH), 131.4 (CH), 130.1 (CH), 129.6 (CH), 128.3 (CH), 128.1 (CH), 127.6 (CH), 122.9 (C_q), 122.9 (C_q), 122.1 (CH), 117.7 (CH), 35.0 (C_q), 31.5 (CH_3). **IR** (ATR): $\tilde{\nu}$ = 1636, 1608, 1486, 1362, 1260, 1219, 924, 828, 765, 697, 516 cm^{-1}. **M.p.**: 152–154 °C. **MS** (ESI) *m/z* (relative intensity): 355 (50) $[M+H]^+$, 377 (100) $[M+Na]^+$, 731 (80) $[2M+Na]^+$. **HR-MS** (ESI) *m/z* calcd for $C_{25}H_{23}O_2$ $[M+H]^+$: 355.1693, found: 355.1694.
The spectral data are in accordance with those reported in the literature.[21]

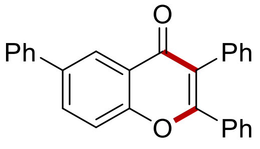

19

2,3,6-Triphenyl-4*H*-chromen-4-one

The general procedure A was followed using **16b** (149 mg, 0.75 mmol) and **4** (44.5 mg, 0.25 mmol) at 100 °C for 3.5 h. Purification by column chromatography on silica gel (*n*hexane/EtOAc = 13/1) yielded **19** (77.6 mg, 83%) as a white solid. **^{1}H-NMR** (500 MHz, $CDCl_3$): δ = 8.41 (d, J = 2.3 Hz, 1H), 7.84 (dd, J = 8.7, 2.3 Hz, 1H), 7.61–7.57 (m, 2H), 7.51 (d, J = 8.7 Hz, 1H), 7.38 (dd, J = 7.7 Hz, 7.7 Hz, 2H), 7.34–7.26 (m, 3H), 7.26–7.14 (m, 8H). **^{13}C-NMR** (126 MHz, $CDCl_3$): δ = 177.4 (C_q), 161.6 (C_q), 155.6 (C_q), 139.6 (C_q), 138.3 (C_q), 133.4 (C_q), 133.0 (C_q), 132.7 (CH), 131.3 (CH), 130.2 (CH), 129.7 (CH), 129.1 (CH), 128.4 (CH), 128.2 (CH), 127.9 (CH), 127.7 (CH), 127.3 (CH), 124.2 (CH), 123.7 (C_q), 123.0 (C_q), 118.6 (CH). **IR** (ATR): $\tilde{\nu}$ = 1642, 1608, 1476, 1361, 1226, 925, 764, 693, 640, 492 cm^{-1}. **M.p.**: 178–180 °C. **MS** (ESI) *m/z* (relative intensity): 375 (100) $[M+H]^+$, 397 (10) $[M+Na]^+$, 771 (20) $[2M+Na]^+$. **HR-MS** (ESI) *m/z* calcd for $C_{27}H_{19}O_2$ $[M+H]^+$: 375.1380, found: 375.1381.

The spectral data are in accordance with those reported in the literature.[21]

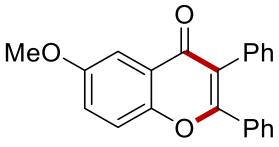

20

6-Methoxy-2,3-diphenyl-4*H*-chromen-4-one

The general procedure A was followed using **16c** (114 mg, 0.75 mmol) and **4** (44.5 mg, 0.25 mmol) at 100 °C for 3.5 h. Purification by column chromatography on silica gel (*n*hexane/EtOAc = 10/1 to 6/1) yielded **20** (69.6 mg, 85%) as a pale-yellow solid.

^{1}H-NMR (400 MHz, $CDCl_3$): δ = 7.56 (d, J = 3.1 Hz, 1H), 7.37 (d, J = 9.1 Hz, 1H), 7.32–7.27 (m, 2H), 7.24–7.12 (m, 9H), 3.93 (s, 3H). **^{13}C-NMR** (101 MHz, $CDCl_3$): δ = 177.1 (C_q), 161.3 (C_q), 157.0 (C_q), 151.0 (C_q), 133.4 (C_q), 133.1 (C_q), 131.3 (CH), 130.0 (CH), 129.6 (CH), 128.3 (CH), 128.1 (CH), 127.6 (CH), 124.1 (C_q), 123.8 (CH), 122.2 (C_q), 119.5 (CH), 105.4 (CH), 56.0 (CH_3). **IR** (ATR): $\tilde{\nu}$ = 1626, 1481, 1435, 1361, 1275, 1022, 830, 783, 743, 570 cm^{-1}. **M.p.**: 179°–181 °C. **MS** (ESI) *m/z* (relative intensity): 329 (100) $[M+H]^+$, 351 (20) $[M+Na]^+$, 679 (40) $[2M+Na]^+$. **HR-MS** (ESI) *m/z* calcd for $C_{22}H_{17}O_3$ $[M+H]^+$: 329.1172, found: 329.1174.

The spectral data are in accordance with those reported in the literature.[21]

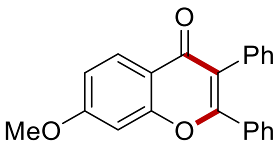

21

7-Methoxy-2,3-diphenyl-4*H*-chromen-4-one

The general procedure A was followed using **16d** (114 mg, 0.75 mmol) and **4** (44.5 mg, 0.25 mmol) at 100 °C for 3.5 h. Purification by column chromatography on silica gel (*n*hexane/EtOAc = 8/1 to 5/1) yielded **21** (74.8 mg, 91%) as a yellowish solid.

^{1}H-NMR (400 MHz, $CDCl_3$): δ = 8.09 (d, J = 8.9 Hz, 1H), 7.31–7.26 (m, 2H), 7.25–7.10 (m, 8H), 6.89 (dd, J = 8.9, 2.4 Hz, 1H), 6.82 (d, J = 2.4 Hz, 1H), 3.81 (s, 3H). **^{13}C-NMR** (101 MHz, $CDCl_3$): δ = 176.8 (C_q), 164.2 (C_q), 161.1 (C_q), 157.8 (C_q), 133.4 (C_q), 133.0 (C_q), 131.3 (CH), 130.0 (CH), 129.6 (CH), 128.3 (CH), 128.1 (CH), 127.8 (CH), 127.6 (CH), 122.8 (C_q), 117.5 (C_q), 114.6 (CH), 100.1 (CH), 55.9 (CH_3). **IR** (ATR): $\tilde{\nu}$ = 1621, 1439, 1379, 1250, 1193, 1016, 829, 746, 690, 629 cm^{-1}. **M.p.**: 216–218 °C. **MS** (ESI) *m/z* (relative intensity): 329 (100) $[M+H]^+$, 351 (20) $[M+Na]^+$, 679 (50) $[2M+Na]^+$. **HR-MS** (ESI) *m/z* calcd for $C_{22}H_{17}O_3$ $[M+H]^+$: 329.1172, found: 329.1176.

The spectral data are in accordance with those reported in the literature.[22]

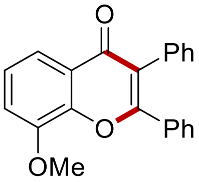

22

8-Methoxy-2,3-diphenyl-4*H*-chromen-4-one

The general procedure A was followed using **16e** (114 mg, 0.75 mmol) and **4** (44.5 mg, 0.25 mmol) at 100 °C for 3.5 h. Purification by column chromatography on silica gel (*n*hexane/EtOAc = 8/1) yielded **22** (78.3 mg, 95%) as a white solid.

^{1}H-NMR (400 MHz, $CDCl_3$): δ = 7.75 (dd, J = 8.1, 1.4 Hz, 1H), 7.37–7.32 (m, 2H), 7.25–7.13 (m, 9H), 7.09 (dd, J = 8.1, 1.4 Hz, 1H), 3.90 (s, 3H). **^{13}C-NMR** (101 MHz, $CDCl_3$): δ = 177.4 (C_q), 161.2 (C_q), 149.0 (C_q), 146.6 (C_q), 133.3 (C_q), 133.0 (C_q), 131.3 (CH), 130.1 (CH), 129.8 (CH), 128.3 (CH), 128.2 (CH), 127.7 (CH), 124.8 (CH), 124.6 (C_q), 122.9 (C_q), 117.1 (CH), 114.3 (CH), 56.4 (CH_3). **IR** (ATR): $\tilde{\nu}$ = 1626, 1558, 1489, 1376, 1266, 1227, 1091, 1069, 757, 695 cm^{-1}. **M.p.**: 187–189 °C. **MS** (ESI) *m/z* (relative intensity): 329 (40) $[M+H]^+$, 451 (100) $[M+Na]^+$, 679 (80) $[2M+Na]^+$. **HR-MS** (ESI) *m/z* calcd for $C_{22}H_{17}O_3$ $[M+H]^+$: 329.1172, found: 329.1173.

The spectral data are in accordance with those reported in the literature.[23]

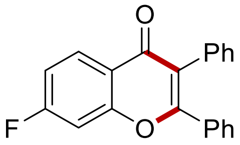

23

7-Fluoro-2,3-diphenyl-4*H*-chromen-4-one

The general procedure A was followed using **1** (105 mg, 0.75 mmol) and **4** (44.5 mg, 0.25 mmol) at 100 °C for 3.5 h. Purification by column chromatography on silica gel (*n*hexane/EtOAc = 15/1) yielded **23** (76.7 mg, 97%) as a white solid.

^{1}H-NMR (400 MHz, $CDCl_3$): δ = 8.21 (dd, J = 8.9, 6.3 Hz, 1H), 7.31–7.26 (m, 2H), 7.26–7.15 (m, 6H), 7.14–7.10 (m, 3H), 7.05 (ddd, J = 8.9, 8.2, 2.4 Hz, 1H). **^{13}C-NMR** (101 MHz, $CDCl_3$): δ = 176.5 (C_q), 165.8 (d, $^{1}J_{C–F}$ = 254.8 Hz, C_q), 161.8 (C_q), 157.1 (d, $^{3}J_{C–F}$ = 13.4 Hz, C_q), 133.0 (C_q), 132.6 (C_q), 131.2 (CH), 130.3 (CH), 129.6 (CH), 129.0 (d, J = 10.7 Hz, CH), 128.4 (CH), 128.2 (CH), 127.8 (CH), 123.1 (C_q), 120.5 (d, $^{4}J_{C–F}$ = 2.3 Hz, C_q), 114.0 (d, $^{2}J_{C–F}$ = 22.8 Hz, CH), 104.6 (d, $^{2}J_{C–F}$ = 25.1 Hz, CH). **^{19}F{^{1}H}-NMR** (282 MHz, $CDCl_3$): δ = –103.1. **IR** (ATR): $\tilde{\nu}$ = 1640, 1611, 1437, 1373, 1242, 1157, 853, 746, 690, 618 cm^{-1}. **M.p.**: 169–170 °C. **MS** (ESI) *m/z* (relative intensity): 317 (30) $[M+H]^+$, 339 (100) $[M+Na]^+$, 655 (40) $[2M+Na]^+$. **HR-MS** (ESI) *m/z* calcd for $C_{21}H_{14}FO_2$ $[M+H]^+$: 317.0972, found: 317.0975.

The spectral data are in accordance with those reported in the literature.[21]

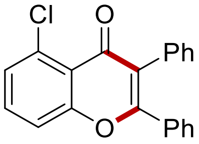

24

5-Chloro-2,3-diphenyl-4*H*-chromen-4-one

The general procedure A was followed using **16f** (118 mg, 0.75 mmol) and **4** (44.5 mg, 0.25 mmol) at 100 °C for 3.5 h. Purification by column chromatography on silica gel (*n*hexane/EtOAc = 15/1) yielded **24** (47.6 mg, 57%) as a white solid.

^{1}H-NMR (500 MHz, $CDCl_3$): δ = 7.45 (dd, J = 8.5, 7.7 Hz, 1H), 7.36 (dd, J = 8.5, 1.2 Hz, 1H), 7.31 (ddd, J = 8.5, 6.8, 1.2 Hz, 3H), 7.27–7.23 (m, 1H), 7.22–7.16 (m, 5H), 7.15–7.13 (m, 2H). **^{13}C-NMR** (126 MHz, $CDCl_3$): δ = 176.4 (C_q), 160.0 (C_q), 157.7 (C_q), 134.1 (C_q), 132.9 (CH), 132.7 (C_q), 132.5 (C_q), 131.3 (CH), 130.3 (CH), 129.6 (CH), 128.3 (CH), 128.2 (CH), 128.1 (CH), 127.8 (CH), 123.9 (C_q), 120.6 (C_q), 117.3 (CH). **IR** (ATR): $\tilde{\nu}$ = 1653, 1618, 1595, 1453,

1373, 949, 785, 752, 699, 648 cm^{-1}. **M.p.**: 165–166 °C. **MS** (ESI) *m/z* (relative intensity): 333 (100) $[M+H]^+$ (^{35}Cl), 355 (10) $[M+Na]^+$ (^{35}Cl), 687 (100) $[2M+Na]^+$ (^{35}Cl). **HR-MS** (ESI) *m/z* calcd for $C_{21}H_{14}{}^{35}ClO_2$ $[M+H]^+$: 333.0677, found: 333.0680.

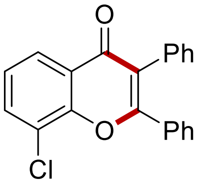

25

8-Chloro-2,3-diphenyl-4*H*-chromen-4-one

The general procedure A was followed using **16g** (118 mg, 0.75 mmol) and **4** (44.5 mg, 0.25 mmol) at 100 °C for 3.5 h. Purification by column chromatography on silica gel (*n*hexane/EtOAc = 12/1 to 7/1) yielded **25** (77.4 mg, 93%) as a white solid.

^{1}H-NMR (400 MHz, $CDCl_3$): δ = 8.11 (dd, *J* = 8.0, 1.5 Hz, 1H), 7.68 (dd, *J* = 7.7, 1.5 Hz, 1H), 7.43–7.36 (m, 2H), 7.30–7.14 (m, 9H). **^{13}C-NMR** (101 MHz, $CDCl_3$): δ = 177.0 (C_q), 161.3 (C_q), 151.8 (C_q), 133.9 (CH), 132.8 (C_q), 132.6 (C_q), 131.2 (CH), 130.5 (CH), 129.9 (CH), 128.6 (CH), 128.3 (CH), 128.0 (CH), 125.2 (CH), 125.1 (CH), 125.0 (C_q), 123.2 (C_q), 123.0 (C_q). **IR** (ATR): $\tilde{\nu}$ = 1642, 1560, 1471, 1440, 1370, 1041, 1024, 758, 689, 652 cm^{-1}. **M.p.**: 154–155 °C. **MS** (ESI) *m/z* (relative intensity): 333 (70) $[M+H]^+$ (^{35}Cl), 355 (100) $[M+Na]^+$ (^{35}Cl), 371 (20) $[M+K]^+$ (^{35}Cl). **HR-MS** (ESI) *m/z* calcd for $C_{21}H_{14}{}^{35}ClO_2$ $[M+H]^+$: 333.0677, found: 333.0681.

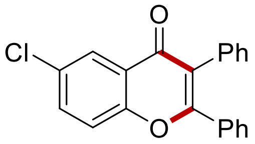

26

6-Chloro-2,3-diphenyl-4*H*-chromen-4-one

The general procedure A was followed using **16h** (117 mg, 0.75 mmol) and **4** (44.5 mg, 0.25 mmol) at 100 °C for 3.5 h. Purification by column chromatography on silica gel (*n*hexane/EtOAc = 12/1) yielded **26** (65.6 mg, 79%) as a white solid.

^{1}H-NMR (400 MHz, $CDCl_3$): δ = 8.15 (d, *J* = 2.5 Hz, 1H), 7.53 (dd, *J* = 8.9, 2.5 Hz, 1H), 7.39 (d, *J* = 8.9 Hz, 1H), 7.31–7.27 (m, 2H), 7.26–7.23 (m, 1H), 7.22–7.19 (m, 3H), 7.19–7.15 (m, 2H), 7.14–7.10 (m, 2H). **^{13}C-NMR** (101 MHz, $CDCl_3$): δ = 176.3 (C_q), 161.8 (C_q), 154.5 (C_q), 134.0 (CH), 133.0 (C_q), 132.5 (C_q), 131.2 (CH), 131.1 (C_q), 130.4 (CH), 129.6 (CH), 128.4 (CH), 128.2 (CH), 127.9 (CH), 125.8 (CH), 124.6 (C_q), 123.0 (C_q), 119.9 (CH). **IR** (ATR): $\tilde{\nu}$

= 1643, 1561, 1432, 1361, 1272, 1221, 1050, 926, 818, 699 cm^{-1}. **M.p.**: 181–182 °C. **MS** (ESI) *m/z* (relative intensity): 333 (100) $[M+H]^+$ (^{35}Cl), 355 (70) $[M+Na]^+$ (^{35}Cl), 687 (90) $[2M+Na]^+$ (^{35}Cl). **HR-MS** (ESI) *m/z* calcd for $C_{21}H_{14}{}^{35}ClO_2$ $[M+H]^+$: 333.0677, found: 333.0679.
The spectral data are in accordance with those reported in the literature.[23]

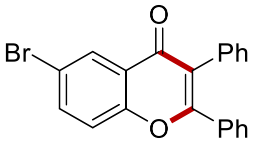

27

6-Bromo-2,3-diphenyl-4*H*-chromen-4-one

The general procedure A was followed using **16i** (168 mg, 0.75 mmol) and **4** (44.5 mg, 0.25 mmol) at 100 °C for 3.5 h. Purification by column chromatography on silica gel (*n*hexane/EtOAc = 15/1) yielded **27** (85.0 mg, 90%) as a white solid.

^{1}H-NMR (400 MHz, $CDCl_3$): δ = 8.31 (d, J = 2.5 Hz, 1H), 7.68 (dd, J = 8.8, 2.5 Hz, 1H), 7.34 (d, J = 8.8 Hz, 1H), 7.29 (m, 2H), 7.27–7.23 (m, 1H), 7.22–7.16 (m, 5H), 7.14–7.10 (m, 2H). **^{13}C-NMR** (101 MHz, $CDCl_3$): δ = 176.1 (C_q), 161.8 (C_q), 154.9 (C_q), 136.7 (CH), 133.0 (C_q), 132.5 (C_q), 131.2 (CH), 130.4 (CH), 129.6 (CH), 129.0 (CH), 128.4 (CH), 128.2 (CH), 127.9 (CH), 124.9 (C_q), 123.1 (C_q), 120.1 (CH), 118.5 (C_q). **IR** (ATR): $\tilde{\nu}$ = 1637, 1603, 1466, 1424, 1356, 1270, 1220, 756, 698, 652 cm^{-1}. **M.p.**: 190–191 °C. **MS** (ESI) *m/z* (relative intensity): 377 (100) $[M+H]^+$ (^{79}Br), 399 (90) $[M+Na]^+$ (^{79}Br), 777 (100) $[2M+Na]^+$ (^{79}Br). **HR-MS** (ESI) *m/z* calcd for $C_{21}H_{14}{}^{79}BrO_2$ $[M+H]^+$: 377.0172, found: 377.0174.

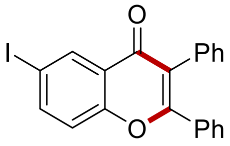

28

6-Iodo-2,3-diphenyl-4*H*-chromen-4-one

The general procedure A was followed using **16j** (186 mg, 0.75 mmol) and **4** (44.5 mg, 0.25 mmol) at 100 °C for 3.5 h. Purification by column chromatography on silica gel (*n*hexane/EtOAc = 20/1) yielded **28** (85.0 mg, 82%) as a white solid.

^{1}H-NMR (300 MHz, $CDCl_3$): δ = 8.50 (d, J = 2.2 Hz, 1H), 7.85 (dd, J = 8.8, 2.2 Hz, 1H), 7.31–7.14 (m, 9H), 7.16–7.06 (m, 2H). **^{13}C-NMR** (101 MHz, $CDCl_3$): δ = 175.9 (C_q), 161.8 (C_q), 155.6 (C_q), 142.3 (CH), 135.3 (CH), 133.0 (C_q), 132.6 (C_q), 131.2 (CH), 130.4 (CH), 129.7

(CH), 128.5 (CH), 128.2 (CH), 127.9 (CH), 125.3 (C_q), 123.3 (C_q), 120.2 (CH), 89.0 (C_q). **IR** (ATR): $\tilde{\nu}$ = 1632, 1597, 1550, 1415, 1359, 1269, 1224, 764, 695, 648 cm^{-1}. **M.p.**: 178–180 °C. **MS** (ESI) *m/z* (relative intensity): 425 (100) $[M+H]^+$, 447 (70) $[M+Na]^+$, 871 (70) $[2M+Na]^+$. **HR-MS** (ESI) *m/z* calcd for $C_{21}H_{14}IO_2$ $[M+H]^+$: 425.0033, found: 425.0037.

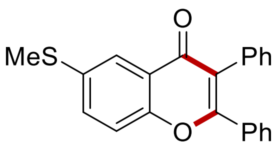

29

6-(Methylthio)-2,3-diphenyl-4*H*-chromen-4-one

The general procedure A was followed using **16k** (168 mg, 0.75 mmol) and **4** (44.5 mg, 0.25 mmol) at 100 °C for 3.5 h. Purification by column chromatography on silica gel (*n*hexane/EtOAc = 15/1) yielded **29** (75.9 mg, 88%) as a yellow solid.

^{1}H-NMR (400 MHz, $CDCl_3$): δ = 7.98 (d, *J* = 2.4 Hz, 1H), 7.49 (dd, *J* = 8.8, 2.4 Hz, 1H), 7.36 (d, *J* = 8.8 Hz, 1H), 7.32–7.28 (m, 2H), 7.27–7.11 (m, 8H), 2.48 (s, 3H). **^{13}C-NMR** (101 MHz, $CDCl_3$): δ = 176.7 (C_q), 161.5 (C_q), 154.1 (C_q), 136.2 (C_q), 133.3 (C_q), 132.9 (C_q), 132.7 (CH), 131.3 (CH), 130.2 (CH), 129.6 (CH), 128.3 (CH), 128.2 (CH), 127.7 (CH), 123.9 (C_q), 123.1 (C_q), 122.1 (CH), 118.6 (CH), 16.2 (CH_3). **IR** (ATR): $\tilde{\nu}$ = 1633, 1599, 1457, 1420, 1357, 1272, 1223, 768, 696 cm^{-1}. **M.p.**: 130–132 °C. **MS** (ESI) *m/z* (relative intensity): 345 (60) $[M+H]^+$, 367 (70) $[M+Na]^+$, 711 (100) $[2M+Na]^+$. **HR-MS** (ESI) *m/z* calcd for $C_{22}H_{17}SO_2$ $[M+H]^+$: 345.0944, found: 345.0947.

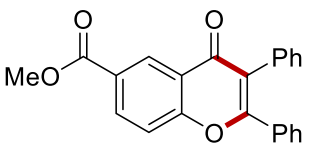

30

Methyl 4-oxo-2,3-diphenyl-4*H*-chromene-6-carboxylate

The general procedure B was followed using **16l** (135 mg, 0.75 mmol) and **4** (44.5 mg, 0.25 mmol) at 100 °C for 3.5 h. Purification by column chromatography on silica gel (*n*hexane/EtOAc = 8/1 to 6/1) yielded **30** (78.2 mg, 88%) as a white solid.

^{1}H-NMR (400 MHz, $CDCl_3$): δ = 8.88 (d, *J* = 2.1 Hz, 1H), 8.27 (dd, *J* = 8.8, 2.1 Hz, 1H), 7.50 (d, *J* = 8.8 Hz, 1H), 7.33–7.29 (m, 2H), 7.29–7.24 (m, 1H), 7.24–7.16 (m, 5H), 7.16–7.12 (m, 2H), 3.88 (s, 3H). **^{13}C-NMR** (101 MHz, $CDCl_3$): δ = 176.8 (C_q), 166.0 (C_q), 161.7 (C_q), 158.6

(C_q), 134.4 (CH), 132.9 (C_q), 132.4 (C_q), 131.2 (CH), 130.4 (CH), 129.7 (CH), 129.0 (CH), 128.4 (CH), 128.2 (CH), 127.9 (CH), 127.2 (C_q), 123.4 (C_q), 123.3 (C_q), 118.6 (CH), 52.5 (CH_3). **IR** (ATR): $\tilde{\nu}$ = 1716, 1642, 1606, 1430, 1291, 1256, 1212, 762, 695, 653 cm^{-1}. **M.p.**: 200–201 °C. **MS** (ESI) *m/z* (relative intensity): 357 (100) $[M+H]^+$, 379 (20) $[M+Na]^+$, 735 (30) $[2M+Na]^+$. **HR-MS** (ESI) *m/z* calcd for $C_{23}H_{17}O_4$ $[M+H]^+$: 357.1121, found: 357.1124.

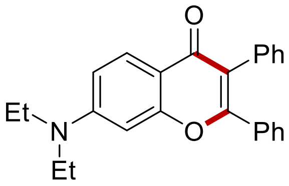

31

7-(Diethylamino)-2,3-diphenyl-4H-chromen-4-one

The general procedure A was followed using **16m** (145 mg, 0.75 mmol) and **4** (44.5 mg, 0.25 mmol) at 100 °C for 3.5 h. Purification by column chromatography on silica gel (*n*hexane/EtOAc = 5/1 to 2/1) yielded **31** (86.1 mg, 93%) as a yellow solid.

^{1}H-NMR (400 MHz, $CDCl_3$): δ = 8.01 (d, J = 9.1 Hz, 1H), 7.33–7.29 (m, 2H), 7.26–7.10 (m, 8H), 6.69 (dd, J = 9.1, 2.5 Hz, 1H), 6.46 (d, J = 2.5 Hz, 1H), 3.39 (q, J = 7.1 Hz, 4H), 1.17 (t, J = 7.0 Hz, 6H). **^{13}C-NMR** (101 MHz, $CDCl_3$): δ = 176.5 (C_q), 160.2 (C_q), 158.6 (C_q), 152.0 (C_q), 134.1 (C_q), 133.6 (C_q), 131.5 (CH), 129.7 (CH), 129.6 (CH), 128.2 (CH), 128.1 (CH), 127.7 (CH), 127.3 (CH), 122.5 (C_q), 112.9 (C_q), 110.7 (CH), 96.3 (CH), 44.9 (CH_2), 12.7 (CH_3). **IR** (ATR): $\tilde{\nu}$ = 1618, 1596, 1442, 1377, 1352, 1267, 1155, 818, 771, 696 cm^{-1}. **M.p.**: 164–165 °C. **MS** (ESI) *m/z* (relative intensity): 370 (100) $[M+H]^+$, 392 (30) $[M+Na]^+$. **HR-MS** (ESI) *m/z* calcd for $C_{25}H_{24}NO_2$ $[M+H]^+$: 370.1802, found: 370.1806.

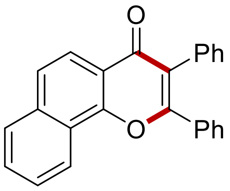

32

2,3-Diphenyl-4*H*-benzo[*h*]chromen-4-one

The general procedure A was followed using **16n** (129 mg, 0.75 mmol) and **4** (44.5 mg, 0.25 mmol) at 100 °C for 7 h. Purification by column chromatography on silica gel (*n*hexane/EtOAc = 12/1) yielded **32** (75.3 mg, 86%) as a light brown solid.

^{1}H-NMR (400 MHz, $CDCl_3$): δ = 8.53 (dd, J = 7.7, 1.6 Hz, 1H), 8.24 (d, J = 8.7 Hz, 1H), 7.94 (dd, J = 7.7, 1.6 Hz, 1H), 7.78 (d, J = 8.7 Hz, 1H), 7.73–7.65 (m, 2H), 7.54–7.49 (m, 2H), 7.42–7.28 (m, 8H). **^{13}C-NMR** (101 MHz, $CDCl_3$): δ =177.2 (C_q), 160.7 (C_q), 153.4 (C_q), 136.0 (C_q), 133.4 (C_q), 132.9 (C_q), 131.3 (CH), 130.2 (CH), 129.7 (CH), 129.3 (CH), 128.4 (CH), 128.3 (CH), 128.3 (CH), 127.8 (CH), 127.2 (CH), 125.3 (CH), 124.1 (C_q), 124.1 (C_q), 122.5 (CH), 121.5 (CH), 119.9 (C_q). **IR** (ATR): $\tilde{\nu}$ = 1627, 1441, 1384, 1239, 1150, 1064, 757, 692, 638, 524 cm^{-1}. **M.p.**: 206–208. **MS** (ESI) *m/z* (relative intensity): 349 (90) $[M+H]^+$, 471 (90) $[M+Na]^+$, 719 (100) $[2M+Na]^+$. **HR-MS** (ESI) *m/z* calcd for $C_{25}H_{17}O_2$ $[M+H]^+$: 349.1223, found: 349.1222.

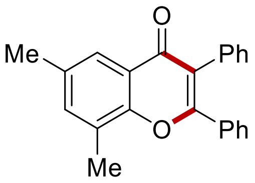

33

6,8-Dimethyl-2,3-diphenyl-4*H*-chromen-4-one

The general procedure A was followed using **16o** (113 mg, 0.75 mmol) and **4** (44.5 mg, 0.25 mmol) at 100 °C for 3.5 h. Purification by column chromatography on silica gel (*n*hexane/EtOAc = 15/1) yielded **33** (72.7 mg, 91%) as a white solid.

^{1}H-NMR (400 MHz, $CDCl_3$): δ = 7.82 (dd, J = 1.5, 1.5 Hz, 1H), 7.34–7.30 (m, 2H), 7.28–7.26 (m, 1H), 7.25–7.21 (m, 2H), 7.21–7.13 (m, 6H), 2.43 (s, 3H), 2.35 (s, 3H). **^{13}C-NMR** (101 MHz, $CDCl_3$): δ = 177.8 (C_q), 160.9 (C_q), 152.9 (C_q), 136.0 (CH), 134.5 (C_q), 133.7 (C_q), 133.3 (C_q), 131.4 (CH), 130.0 (CH), 129.7 (CH), 128.3 (CH), 128.2 (CH), 127.6 (CH), 127.1 (C_q), 123.3 (CH), 123.2 (C_q), 122.6 (C_q), 21.1 (CH_3), 15.7 (CH_3). **IR** (ATR): $\tilde{\nu}$ = 1631, 1603, 1472, 1371, 1267, 918, 802, 728, 696, 570 cm^{-1}. **M.p.**: 198–199 °C. **MS** (ESI) *m/z* (relative intensity): 327 (100) $[M+H]^+$, 349 (30) $[M+Na]^+$, 675 (20) $[2M+Na]^+$. **HR-MS** (ESI) *m/z* calcd for $C_{23}H_{19}O_2$ $[M+H]^+$: 327.1380, found: 327.1382.

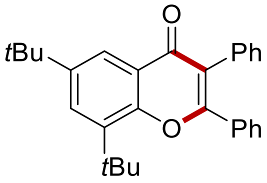

34

6,8-Di-*tert*-butyl-2,3-diphenyl-4*H*-chromen-4-one

The general procedure A was followed using **16p** (176 mg, 0.75 mmol) and **4** (44.5 mg, 0.25 mmol) at 100 °C for 7 h. Purification by column chromatography on silica gel (*n*hexane/EtOAc = 30/1 to 20/1) yielded **34** (96.1 mg, 94%) as a light brown solid.

^{1}H-NMR (400 MHz, $CDCl_3$): δ = 8.12 (d, *J* = 2.5 Hz, 1H), 7.66 (d, *J* = 2.5 Hz, 1H), 7.33 (dd, *J* = 2.5, 2.5 Hz, 2H), 7.27–7.10 (m, 8H), 1.46 (s, 9H), 1.31 (s, 9H). **^{13}C-NMR** (101 MHz, $CDCl_3$): δ = 178.0 (C_q), 161.3 (C_q), 153.2 (C_q), 147.6 (C_q), 138.5 (C_q), 133.7 (C_q), 133.2 (C_q), 131.4 (CH), 129.9 (CH), 129.7 (CH), 128.8 (CH), 128.2 (CH), 128.2 (CH), 127.5 (CH), 123.8 (C_q), 122.6 (C_q), 120.4 (CH), 35.2 (C_q), 35.1 (C_q), 31.5 (CH_3), 30.4 (CH_3). **IR** (ATR): $\tilde{\nu}$ = 1631, 1593, 1467, 1444, 1364, 1234, 1062, 774, 691, 650 cm^{-1}. **M.p.**: 178–181 °C. **MS** (ESI) *m/z* (relative intensity): 411 (100) $[M+H]^+$, 433 (10) $[M+Na]^+$, 821 (10) $[2M+H]^+$. **HR-MS** (ESI) *m/z* calcd for $C_{29}H_{31}O_2$ $[M+H]^+$: 411.2319, found: 411.2321.

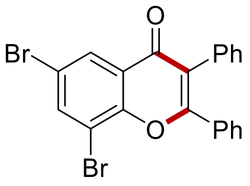

35

6,8-Dibromo-2,3-diphenyl-4*H*-chromen-4-one

The general procedure A was followed using **16q** (223 mg, 0.75 mmol) and **4** (44.5 mg, 0.25 mmol) at 100 °C for 7 h. Purification by column chromatography on silica gel (*n*hexane/EtOAc = 10/1) yielded **35** (85.3 mg, 75%) as a white solid.

^{1}H-NMR (400 MHz, $CDCl_3$): δ = 8.27 (d, *J* = 2.3 Hz, 1H), 7.97 (d, *J* = 2.3 Hz, 1H), 7.41–7.36 (m, 2H), 7.31–7.17 (m, 6H), 7.16–7.11 (m, 2H). **^{13}C-NMR** (101 MHz, $CDCl_3$): δ = 175.8 (C_q), 161.6 (C_q), 151.8 (C_q), 139.3 (CH), 132.5 (C_q), 132.2 (C_q), 131.1 (CH), 130.8 (CH), 129.9 (CH), 128.7 (CH), 128.4 (CH), 128.4 (CH), 128.2 (CH), 125.6 (C_q), 123.0 (C_q), 118.4 (C_q), 113.0 (C_q). **IR** (ATR): $\tilde{\nu}$ = 1637, 1550, 1467, 1351, 1258, 1217, 1049, 924, 769, 697 cm^{-1}. **M.p.**: 219–220 °C. **MS** (ESI) *m/z* (relative intensity): 456 (100) $[M+H]^+$ ($^{79}Br^{81}Br$), 478 (10) $[M+Na]^+$ ($^{79}Br^{81}Br$), 934 (20) $[2M+Na]^+$ ($^{79}Br^{81}Br$). **HR-MS** (ESI) *m/z* calcd for $C_{21}H_{13}{}^{79}Br^{81}BrO_2$ $[M+H]^+$: 456.9258, found: 456.9254.

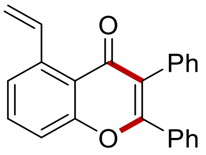

36

2,3-Diphenyl-5-vinyl-4*H*-chromen-4-one

The general procedure A was followed using **16r** (111 mg, 0.75 mmol) and **4** (44.5 mg, 0.25 mmol) at 100 °C for 3.5 h. Purification by column chromatography on silica gel (*n*hexane/EtOAc = 10/1) yielded **36** (72.5 mg, 92%) as a brown solid.

^{1}H-NMR (400 MHz, $CDCl_3$): δ = 8.12 (dd, *J* = 17.3, 10.9 Hz, 1H), 7.65 (dd, *J* = 8.7, 7.2 Hz, 1H), 7.52–7.40 (m, 4H), 7.39–7.21 (m, 8H), 5.64 (dd, *J* = 17.3, 1.6 Hz, 1H), 5.40 (dd, *J* = 10.9, 1.6 Hz, 1H). **^{13}C-NMR** (101 MHz, $CDCl_3$): δ = 179.1 (C_q), 160.0 (C_q), 157.1 (C_q), 141.1 (C_q), 137.4 (CH), 133.1 (CH), 133.0 (C_q), 132.9 (CH), 131.3 (CH), 130.1 (CH), 129.5 (CH), 128.3 (CH), 128.1 (CH), 127.6 (CH), 124.1 (CH), 123.7 (C_q), 120.4 (C_q), 117.6 (CH), 116.6 (CH_2). **IR** (ATR): $\tilde{\nu}$ = 3055, 2926, 1626, 1564, 1470, 1369, 1228, 1058, 976, 819 cm^{-1}. **M.p.**: 154–155. **MS** (ESI) *m/z* (relative intensity): 325 (100) $[M+H]^+$, 671 (70) $[2M+Na]^+$. **HR-MS** (ESI) *m/z* calcd for $C_{23}H_{17}O_2$ $[M+H]^+$: 325.1229, found: 325.1223.

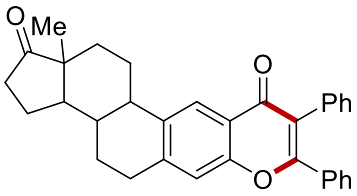

37

13a-Methyl-8,9-diphenyl-2,3,3a,3b,4,5,11b,12,13,13a-decahydrocyclopenta[5,6]naphtho-[1,2-*g*]chromene-1,10-dione

The general procedure A was followed using **16s** (112 mg, 0.375 mmol) and **4** (44.5 mg, 0.25 mmol) at 100 °C for 3.5 h. Purification by column chromatography on silica gel (*n*hexane/EtOAc = 4/1 to 2/1) yielded **37** (91.8 mg, 77%) as a white solid.

^{1}H-NMR (500 MHz, $CDCl_3$): δ =8.09 (d, *J* = 1.3 Hz, 1H), 7.31–7.28 (m, 2H), 7.27–7.21 (m, 2H), 7.21–7.16 (m, 5H), 7.15–7.11 (m, 2H), 3.05–2.93 (m, 2H), 2.55–2.48 (m, 1H), 2.48–2.41 (m, 1H), 2.30 (td, *J* = 11.0, 4.3 Hz, 1H), 2.09 (dt, *J* = 18.8, 8.8 Hz, 1H), 2.01 (ddq, *J* = 12.1, 5.7, 3.5, 2.9 Hz, 2H), 1.94 (dt, *J* = 13.0, 3.5 Hz, 1H), 1.62–1.52 (m, 3H), 1.51–1.41 (m, 3H), 0.85 (s, 3H). **^{13}C-NMR** (126 MHz, $CDCl_3$): δ = 220.6 (C_q), 177.3 (C_q), 161.2 (C_q), 154.4 (C_q), 144.2 (C_q), 137.9 (C_q), 133.6 (C_q), 133.2 (C_q), 131.4 (CH), 130.0 (CH), 129.6 (CH), 128.3 (CH),

128.1 (CH), 127.6 (CH), 122.7 (C_q), 122.6 (CH), 121.4 (C_q), 117.3 (CH), 50.7 (CH), 48.0 (C_q), 44.2 (CH), 38.0 (CH), 35.9 (CH_2), 31.5 (CH_2), 29.8 (CH_2), 26.2 (CH_2), 25.9 (CH_2), 21.7 (CH_2), 13.9 (CH_3). **IR** (ATR): $\tilde{\nu}$ = 1730, 1641, 1616, 1439, 1368, 1242, 1218, 767, 737, 694 cm^{-1}. **M.p.**: 284–286 °C. **MS** (ESI) *m/z* (relative intensity): 475 (100) $[M+H]^+$, 492 (20) $[M+Na]^+$, 971 (10) $[2M+Na]^+$. **HR-MS** (ESI) *m/z* calcd for $C_{33}H_{31}O_3$ $[M+H]^+$: 475.2268, found: 475.2270.

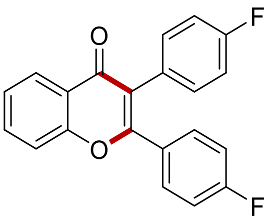

38

2,3-Bis(4-fluorophenyl)-4*H*-chromen-4-one

The general procedure A was followed using **3** (78 µL, 0.75 mmol) and **2** (53.6 mg, 0.25 mmol) at 100 °C for 3.5 h. Purification by column chromatography on silica gel (*n*hexane/EtOAc = 10/1) yielded **38** (75.6 mg, 90%) as a white solid.

^{1}H-NMR (400 MHz, $CDCl_3$): δ = 8.28 (ddd, *J* = 7.9, 1.7, 0.5 Hz, 1H), 7.71 (ddd, *J* = 8.5, 7.1, 1.7 Hz, 1H), 7.53 (ddd, *J* = 8.5, 1.1, 0.5 Hz, 1H), 7.46–7.43 (m, 3H), 7.42–7.37 (m, 2H), 7.22–7.16 (m, 2H), 7.05–6.96 (m, 4H). **^{13}C-NMR** (101 MHz, $CDCl_3$): δ = 177.3 (C_q), 163.6 (d, *J* = 252.4 Hz, C_q), 162.4 (d, *J* = 247.4 Hz, C_q), 160.7 (C_q), 156.0 (C_q), 134.0 (CH), 133.0 (d, *J* = 8.1 Hz, CH), 131.8 (d, *J* = 8.7 Hz, CH), 129.3 (d, *J* = 3.4 Hz, C_q), 128.7 (d, *J* = 3.5 Hz, C_q), 126.5 (CH), 125.4 (CH), 123.4 (C_q), 122.0 (C_q), 118.0 (CH), 115.6 (d, *J* = 21.5 Hz, CH), 115.6 (d, *J* = 21.9 Hz, CH). **^{19}F{^{1}H}-NMR** (376 MHz, $CDCl_3$): δ = –108.8, –113.9. **IR** (ATR): $\tilde{\nu}$ = 1626, 1605, 1503, 1462, 1375, 1225, 1154, 825, 759, 518 cm^{-1}. **M.p.**: 158–159 °C. **MS** (ESI) *m/z* (relative intensity): 335 (100) $[M+H]^+$, 357 (50) $[M+Na]^+$, 691 (70) $[2M+Na]^+$. **HR-MS** (ESI) *m/z* calcd for $C_{21}H_{13}O_2F_2$ $[M+H]^+$: 335.0878, found: 335.0878.

The spectral data are in accordance with those reported in the literature.[21]

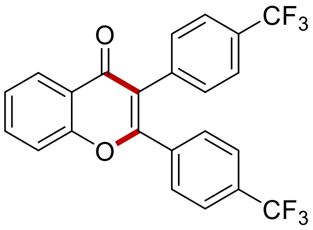

39

2,3-Bis[4-(trifluoromethyl)phenyl]-4*H*-chromen-4-one

The general procedure A was followed using **3** (78 µL, 0.75 mmol) and **10e** (75.5 mg, 0.24 mmol) at 100 °C for 7 h. Purification by column chromatography on silica gel (*n*hexane/EtOAc = 8/1) yielded **39** (52.4 mg, 50%) as a white solid.

^{1}H-NMR (400 MHz, $CDCl_3$): δ = 8.30 (dd, *J* = 8.0, 1.7 Hz, 1H), 7.76 (ddd, *J* = 8.7, 7.2, 1.7 Hz, 1H), 7.61–7.51 (m, 7H), 7.53–7.44 (m, 1H), 7.35 (dt, *J* = 7.8, 0.9 Hz, 2H). **^{13}C-NMR** (101 MHz, $CDCl_3$): δ = 176.8 (C_q), 160.3 (C_q), 156.1 (C_q), 136.4–136.3 (m, C_q), 136.3–136.2 (m, C_q), 134.4 (CH), 132.4 (q, *J* = 32.9 Hz, C_q), 131.7 (CH), 130.2 (q, *J* = 32.6 Hz, C_q), 130.1 (CH),

126.6 (CH), 125.8 (CH), 125.5 (q, J = 3.8 Hz, CH), 125.5 (q, J = 3.8 Hz, CH), 124.2 (q, J = 271.8 Hz, C_q), 123.6 (q, J = 272.6 Hz, C_q), 123.4 (C_q), 122.7 (C_q), 118.2 (CH). **^{19}F-NMR** (376 MHz, $CDCl_3$): δ = –62.7, –63.1. **IR** (ATR): $\tilde{\nu}$ = 1638, 1613, 1463, 1321, 1160, 1104, 1065, 1012, 840, 770 cm^{-1}. **M.p.**: 170–172 °C. **MS** (ESI) *m/z* (relative intensity): 435 (50) $[M+H]^+$, 457 (50) $[M+Na]^+$, 891 (100) $[2M+Na]^+$. **HR-MS** (ESI) *m/z* calcd for $C_{23}H_{13}O_2F_6$ $[M+H]^+$: 435.0814, found: 435.0816.

The spectral data are in accordance with those reported in the literature.[21]

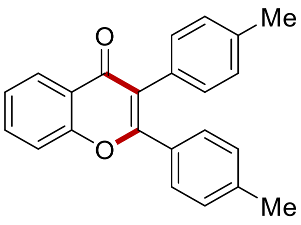

40

2,3-Di-*p*-tolyl-4*H*-chromen-4-one

The general procedure A was followed using **3** (78 µL, 0.75 mmol) and **10f** (51.6 mg, 0.25 mmol) at 100 °C for 3.5 h. Purification by column chromatography on silica gel (*n*hexane/EtOAc = 12/1) yielded **40** (76.9 mg, 94%) as a white solid.

^{1}H-NMR (400 MHz, $CDCl_3$): δ = 8.30 (dd, J = 8.1, 1.7 Hz, 1H), 7.68 (ddd, J = 8.6, 7.1, 1.7 Hz, 1H), 7.52 (dd, J = 8.6, 1.1 Hz, 1H), 7.41 (ddd, J = 8.1, 7.1, 1.1 Hz, 1H), 7.35–7.30 (m, 2H), 7.16–7.12 (m, 4H), 7.09 (d, J = 8.1 Hz, 2H), 2.36 (s, 3H), 2.34 (s, 3H). **^{13}C-NMR** (101 MHz, $CDCl_3$): δ = 177.6 (C_q), 161.5 (C_q), 156.1 (C_q), 140.4 (C_q), 137.2 (C_q), 133.6 (CH), 131.1 (CH), 130.6 (C_q), 130.1 (C_q), 129.6 (CH), 129.1 (CH), 128.9 (CH), 126.4 (CH), 125.0 (CH), 123.6 (C_q), 122.6 (C_q), 118.0 (CH), 21.5 (CH_3), 21.4 (CH_3). **IR** (ATR): $\tilde{\nu}$ = 1637, 1604, 1459, 1371, 1225, 1042, 1013, 814, 751, 500 cm^{-1}. **M.p.**: 139–140 °C. **MS** (ESI) *m/z* (relative intensity): 327 (100) $[M+H]^+$, 349 (20) $[M+Na]^+$, 675 (50) $[2M+Na]^+$. **HR-MS** (ESI) *m/z* calcd for $C_{23}H_{19}O_2$ $[M+H]^+$: 327.1380, found: 327.1381.

The spectral data are in accordance with those reported in the literature.[22]

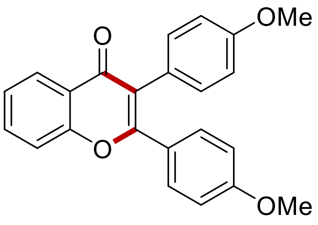

41

2,3-Bis(4-methoxyphenyl)-4*H*-chromen-4-one

The general procedure A was followed using **3** (78 µL, 0.75 mmol) and **10g** (59.6 mg, 0.25 mmol) at 100 °C for 3.5 h. Purification by column chromatography on silica gel (*n*hexane/EtOAc = 5/1 to 3/1) yielded **41** (74.8 mg, 74%) as a white solid.

^{1}H-NMR (400 MHz, $CDCl_3$): δ = 8.27 (dd, *J* = 7.9, 1.6 Hz, 1H), 7.67 (ddd, *J* = 8.7, 7.1, 1.7 Hz, 1H), 7.51 (d, *J* = 8.4 Hz, 1H), 7.42–7.34 (m, 3H), 7.17 (d, *J* = 8.7 Hz, 2H), 6.87 (d, *J* = 8.7 Hz, 2H), 6.79 (d, *J* = 8.9 Hz, 2H), 3.81 (s, 3H), 3.79 (s, 3H). **^{13}C-NMR** (101 MHz, $CDCl_3$): δ = 177.6 (C_q), 161.2 (C_q), 160.9 (C_q), 159.0 (C_q), 156.0 (C_q), 133.5 (CH), 132.4 (CH), 131.3 (CH), 126.4 (CH), 125.7 (C_q), 125.5 (C_q), 124.9 (CH), 123.5 (C_q), 121.6 (C_q), 117.9 (CH), 114.0 (CH), 113.6 (CH), 55.4 (CH_3), 55.3 (CH_3). **IR** (ATR): $\tilde{\nu}$ = 1600, 1504, 1462, 1381, 1239, 1171, 1024, 826, 759, 522 cm^{-1}. **M.p.**: 151–153 °C. **MS** (ESI) *m/z* (relative intensity): 359 (90) $[M+H]^+$, 381 (40) $[M+Na]^+$, 739 (100) $[2M+Na]^+$. **HR-MS** (ESI) *m/z* calcd for $C_{23}H_{19}O_4$ $[M+H]^+$: 359.1278, found: 359.1279.

The spectral data are in accordance with those reported in the literature.[23]

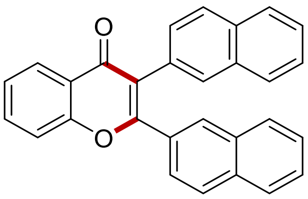

42

2,3-Di(naphthalen-2-yl)-4*H*-chromen-4-one

The general procedure A was followed using **3** (78 µL, 0.75 mmol) and **10h** (69.6 mg, 0.25 mmol) at 100 °C for 7 h. Purification by column chromatography on silica gel (*n*hexane/EtOAc = 10/1 to 5/1) yielded **3da** (81.5 mg, 82%) as a brown solid.

^{1}H-NMR (400 MHz, $CDCl_3$): δ = 8.37 (dd, *J* = 8.0, 1.6 Hz, 1H), 8.14 (s, 1H), 7.86 (s, 1H), 7.80 (d, *J* = 8.0 Hz, 1H), 7.78–7.70 (m, 5H), 7.63 (d, *J* = 8.5 Hz, 1H), 7.57 (d, *J* = 8.5 Hz, 1H), 7.52–7.40 (m, 5H), 7.37–7.30 (m, 2H). **^{13}C-NMR** (101 MHz, $CDCl_3$): δ = 177.6 (C_q), 161.7 (C_q), 156.3 (C_q), 133.9 (CH), 133.8 (C_q), 133.5 (C_q), 132.9 (C_q), 132.7 (C_q), 130.9 (CH), 130.8 (C_q),

130.4 (C_q), 130.1 (CH), 129.1 (CH), 128.9 (CH), 128.3 (CH), 128.0 (CH), 127.7 (CH), 126.7 (CH), 126.5 (CH), 126.3 (CH), 126.2 (CH), 125.9 (CH), 125.3 (CH), 123.7 (C_q), 123.1 (C_q), 118.1 (CH). **IR** (ATR): $\tilde{\nu}$ = 1622, 1464, 1383, 1342, 1218, 904, 858, 741, 660, 473 cm^{-1}. **M.p.**: 110–111 °C. **MS** (ESI) *m/z* (relative intensity): 399 (100) $[M+H]^+$, 421 (10) $[M+Na]^+$, 819 (20) $[2M+Na]^+$. **HR-MS** (ESI) *m/z* calcd for $C_{29}H_{19}O_2$ $[M+H]^+$: 399.1380, found: 399.1381.

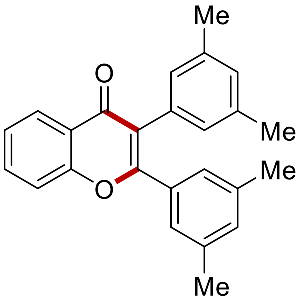

43

2,3-Bis(3,5-dimethylphenyl)-4*H*-chromen-4-one

The general procedure A was followed using **3** (78 µL, 0.75 mmol) and **10i** (58.5 mg, 0.25 mmol) at 100 °C for 3.5 h. Purification by column chromatography on silica gel (*n*hexane/EtOAc = 20/1) yielded **43** (81.3 mg, 92%) as a brown solid.

^{1}H-NMR (400 MHz, $CDCl_3$): δ = 8.32 (dd, *J* = 8.1, 1.7 Hz, 1H), 7.71 (ddd, *J* = 8.6, 7.1, 1.7 Hz, 1H), 7.57 (dd, *J* = 8.6, 1.1 Hz, 1H), 7.43 (ddd, *J* = 8.1, 7.1, 1.1 Hz, 1H), 7.04 (s, 2H), 6.97 (s, 1H), 6.93 (s, 1H), 6.85 (s, 2H), 2.26 (s, 6H), 2.22 (s, 6H). **^{13}C-NMR** (101 MHz, $CDCl_3$): δ = 177.7 (C_q), 161.6 (C_q), 156.1 (C_q), 137.6 (C_q), 137.5 (C_q), 133.6 (CH), 133.2 (C_q), 132.9 (C_q), 131.7 (CH), 129.3 (CH), 128.8 (CH), 127.4 (CH), 126.4 (CH), 125.0 (CH), 123.6 (C_q), 123.2 (C_q), 118.0 (CH), 21.4 (CH_3), 21.3 (CH_3). **IR** (ATR): $\tilde{\nu}$ = 2914, 1633, 1605, 1463, 1369, 1223, 1181, 1084, 847, 766 cm^{-1}. **M.p.**: 153–155 °C. **MS** (ESI) *m/z* (relative intensity): 355 (100) $[M+H]^+$, 731 (60) $[2M+Na]^+$. **HR-MS** (ESI) *m/z* calcd for $C_{25}H_{23}O_2$ $[M+H]^+$: 355.1698, found: 355.1694.

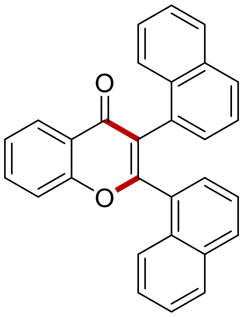

44

2,3-Di(naphthalen-1-yl)-4*H*-chromen-4-one

The general procedure A was followed using **3** (78 µL, 0.75 mmol) and **10j** (69.6 mg, 0.25 mmol) at 100 °C for 7 h. Purification by column chromatography on silica gel (*n*hexane/EtOAc = 12/1 to 8/1) yielded **44** (83.7 mg, 82%) as a yellow solid.

^{1}H-NMR (400 MHz, $CDCl_3$): δ = 8.44 (dd, J = 8.0, 1.6 Hz, 1H), 8.02 (d, J = 7.3 Hz, 1H), 7.93–7.88 (m, 1H), 7.80 (dd, J = 7.3, 2.0 Hz, 1H), 7.79–7.74 (m, 2H), 7.72 (d, J = 8.2 Hz, 1H), 7.67–7.63 (m, 1H), 7.57–7.48 (m, 4H), 7.47–7.41 (m, 2H), 7.22 (d, J = 7.1 Hz, 1H), 7.15–7.11 (m, 2H), 7.08 (t, J = 7.7 Hz, 1H). **^{13}C-NMR** (101 MHz, $CDCl_3$): δ = 177.4 (C_q), 163.8 (C_q), 156.6 (C_q), 134.0 (CH), 133.5 (C_q), 133.3 (C_q), 132.6 (C_q), 131.0 (C_q), 131.0 (C_q), 130.7 (C_q), 130.4 (CH), 128.6 (CH), 128.5 (CH), 128.5 (CH), 128.3 (CH), 127.7 (CH), 127.1 (CH), 126.7 (CH), 126.3 (CH), 126.3 (CH), 125.7 (CH), 125.7 (CH), 125.5 (CH), 125.3 (CH), 125.2 (CH), 124.9 (C_q), 124.7 (CH), 123.8 (C_q), 118.3 (CH). **IR** (ATR): $\tilde{\nu}$ = 1642, 1616, 1566, 1461, 1363, 1212, 1112, 891, 762, 674 cm^{-1}. **M.p.**: 224–226 °C. **MS** (ESI) *m/z* (relative intensity): 399 (100) $[M+H]^+$, 421 (30) $[M+Na]^+$, 819 (50) $[2M+Na]^+$. **HR-MS** (ESI) *m/z* calcd for $C_{29}H_{19}O_2$ $[M+H]^+$: 399.1380, found: 399.1382.

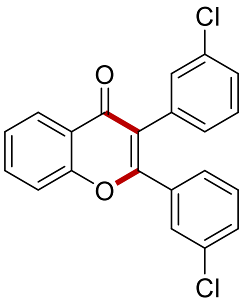

45

2,3-Bis(3-chlorophenyl)-4*H*-chromen-4-one

The general procedure A was followed using **3** (78 µL, 0.75 mmol) and **10k** (61.8 mg, 0.25 mmol) at 100 °C for 3.5 h. Purification by column chromatography on silica gel (*n*hexane/EtOAc = 8/1) yielded **45** (52.0 mg, 55%) as a white solid.

^{1}**H-NMR** (400 MHz, $CDCl_3$): δ = 8.20 (dd, *J* = 8.2, 1.7 Hz, 1H), 7.69–7.63 (m, 1H), 7.48 (d, *J* = 8.6, 1H), 7.46–7.41 (m, 1H), 7.41–7.35 (m, 1H), 7.30–7.25 (m, 1H), 7.24–7.16 (m, 3H), 7.16–7.06 (m, 2H), 7.01–6.97 (m, 1H). ^{13}**C-NMR** (101 MHz, $CDCl_3$): δ = 176.9 (C_q), 160.2 (C_q), 156.1 (C_q), 134.6 (C_q), 134.5 (C_q), 134.4 (C_q), 134.2 (CH), 131.3 (CH), 130.6 (CH), 129.8 (CH), 129.6 (CH), 129.5 (CH), 129.4 (CH), 128.3 (CH), 128.1 (CH), 126.5 (CH), 125.6 (CH), 123.4 (C_q), 122.3 (C_q), 118.1 (CH). **IR** (ATR): $\tilde{\nu}$ = 1640, 1564, 1466, 1372, 1225, 1085, 1052, 787, 755, 697 cm^{-1}. **M.p.**: 148–150 °C. **MS** (ESI) *m/z* (relative intensity): 367.0 (100) $[M+H]^+$ (^{35}Cl), 389.0 (30) $[M+Na]^+$ (^{35}Cl), 757 (80) $[2M+Na]^+$ (^{35}Cl). **HR-MS** (ESI) *m/z* calcd for $C_{21}H_{13}O_2{}^{35}Cl_2$ $[M+H]^+$: 367.0287, found: 367.0289.

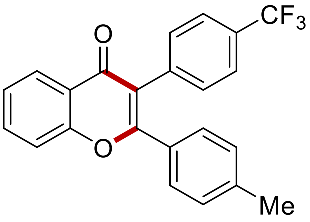

46 (2:1)

2-(*p*-Tolyl)-3-[4-(trifluoromethyl)phenyl]-4*H*-chromen-4-one (mixture of isomers)

The general procedure A was followed using **3** (78 µL, 0.75 mmol) and **10l** (65.1 mg, 0.25 mmol) at 100 °C for 3.5 h. Purification by column chromatography on silica gel (*n*hexane/EtOAc = 10/1) yielded **46** as a 2:1 mixture of isomers (72.7 mg, 76%) appearing as a white solid.

^{1}**H-NMR** (300 MHz, $CDCl_3$): δ = 8.25–8.16 (m, 1H), 7.63–7.59 (m, 1H), 7.50–7.42 (m, 4H), 7.38–7.32 (m, 1H), 7.28 (d, *J* = 8.2 Hz, 1H), 7.18 (d, *J* = 8.2 Hz, 1H), 7.07–6.98 (m, 3H), 2.29–2.23 (m, 3H). ^{19}**F{^{1}H}-NMR** (282 MHz, $CDCl_3$): δ = –62.5, –62.9. **IR** (ATR): $\tilde{\nu}$ = 1641, 1614, 1465, 1377, 1322, 1165, 1119, 1068, 760 cm^{-1}. **M.p.**: 143–144 °C. **MS** (ESI) *m/z* (relative intensity): 381 (100) $[M+H]^+$, 403 (40) $[M+Na]^+$, 783 (60) $[2M+Na]^+$. **HR-MS** (ESI) *m/z* calcd for $C_{23}H_{16}F_3O_2$ $[M+H]^+$: 381.1097, found: 381.1098.

Major isomer:

^{13}**C{^{1}H,^{19}F}-NMR** (151 MHz, $CDCl_3$): δ = 177.0 (C_q), 162.4 (C_q), 156.1 (C_q), 141.1 (C_q), 137.2 (C_q), 134.0 (CH), 131.8 (CH), 129.9 (C_q), 129.6 (C_q), 129.6 (CH), 129.2 (CH), 126.4 (CH), 125.4 (CH), 125.3 (CH), 124.3 (C_q), 123.5 (C_q), 121.5 (C_q), 118.1 (CH), 21.6 (CH_3).

Minor isomer:

^{13}C{^{1}H,^{19}F}-NMR (151 MHz, $CDCl_3$): δ = 177.5 (C_q), 159.5 (C_q), 156.1 (C_q), 138.0 (C_q), 137.0 (C_q), 134.0 (CH), 131.7 (C_q), 131.0 (CH), 130.1 (CH), 129.4 (CH), 129.2 (C_q), 126.6 (CH), 125.4 (CH), 125.2 (CH), 123.8 (C_q), 123.8 (C_q), 123.6 (C_q), 118.1 (CH), 21.4 (CH_3).

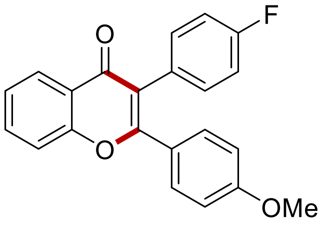

47

3-(4-Fluorophenyl)-2-(4-methoxyphenyl)-4*H*-chromen-4-one

The general procedure A was followed using **3** (78 µL, 0.75 mmol) and **10m** (56.6 mg, 0.25 mmol) at 100 °C for 3.5 h. Purification by column chromatography on silica gel (*n*hexane/EtOAc = 8/1 to 5/1) yielded **47** (47.2 mg, 55%) as a white solid and **47`** (24.3 mg, 28%) as white solid.

^{1}H-NMR (400 MHz, $CDCl_3$): δ = 8.27 (dd, J = 8.1, 1.7 Hz, 1H), 7.69 (ddd, J = 8.6, 7.2, 1.7 Hz, 1H), 7.53 (dd, J = 8.6, 1.1 Hz, 1H), 7.42 (ddd, J = 8.1, 7.2, 1.1 Hz, 1H), 7.37–7.32 (m, 2H), 7.25–7.19 (m, 2H), 7.06–6.99 (m, 2H), 6.83–6.78 (m, 2H), 3.80 (s, 3H). **^{13}C-NMR** (101 MHz, $CDCl_3$): δ = 177.4 (C_q), 162.3 (d, J = 246.8 Hz, C_q), 161.6 (C_q), 161.1 (C_q), 156.1 (C_q), 133.7 (CH), 133.0 (d, J = 8.2 Hz, CH), 131.3 (CH), 129.3 (d, J = 3.6 Hz, C_q), 126.4 (CH), 125.3 (C_q), 125.2 (CH), 123.5 (C_q), 121.1 (C_q), 118.0 (CH), 115.5 (d, J = 21.5 Hz, CH), 113.7 (CH), 55.4 (CH_3). **^{19}F-NMR** (377 MHz, $CDCl_3$): δ = –114.5 (m, 1F). **M.p.**: 201–202 °C. **IR** (ATR): $\tilde{\nu}$ = 1598, 1561, 1462, 1375, 1251, 1226, 1021, 829, 763, 518 cm^{-1}. **MS** (ESI) *m/z* (relative intensity): 347 (100) $[M+H]^+$, 369 (20) $[M+Na]^+$, 715 (60) $[2M+Na]^+$. **HR-MS** (ESI) *m/z* calcd for $C_{22}H_{16}FO_3$ $[M+H]^+$: 347.1078, found: 347.1079.

The spectral data are in accordance with those reported in the literature.[5]

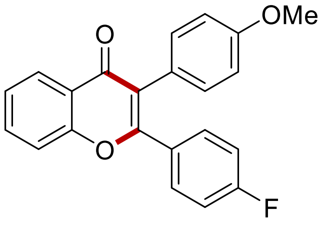

47'

2-(4-Fluorophenyl)-3-(4-methoxyphenyl)-4*H*-chromen-4-one

^{1}H-NMR (400 MHz, $CDCl_3$): δ = 8.29 (ddd, J = 7.9, 1.8, 0.5 Hz, 1H), 7.70 (ddd, J = 8.7, 7.1, 1.7 Hz, 1H), 7.52 (ddd, J = 8.5, 1.1, 0.5 Hz, 1H), 7.47–7.37 (m, 3H), 7.18–7.09 (m, 2H), 7.05–6.93 (m, 2H), 6.90–6.82 (m, 2H), 3.81 (s, 3H). **^{13}C-NMR** (101 MHz, $CDCl_3$): δ = 177.6 (C_q), 163.5 (d, J = 251.8 Hz, C_q), 160.3 (C_q), 159.3 (C_q), 156.1 (C_q), 133.8 (CH), 132.4 (CH), 131.8 (d, J = 8.6 Hz, CH), 129.7 (d, J = 3.3 Hz, C_q), 126.6 (CH), 125.2 (CH), 124.9 (C_q), 123.6 (C_q), 122.6 (C_q), 118.0 (CH), 115.5 (d, J = 21.8 Hz, CH), 114.1 (CH), 55.4 (CH_3). **^{19}F-NMR** (377 MHz, $CDCl_3$): δ = –109.4 (m, 1F). **IR** (ATR): $\tilde{\nu}$ = 1633, 1603, 1505, 1462, 1376, 1227, 1020, 825, 763, 515 cm^{-1}. **M.p.**: 145–146 °C. **MS** (ESI) *m/z* (relative intensity): 347 (100) $[M+H]^+$, 369 (50) $[M+Na]^+$, 715 (60) $[2M+Na]^+$. **HR-MS** (ESI) *m/z* calcd for $C_{22}H_{16}FO_3$ $[M+H]^+$: 347.1078, found: 347.1077.

The spectral data are in accordance with those reported in the literature.[5]

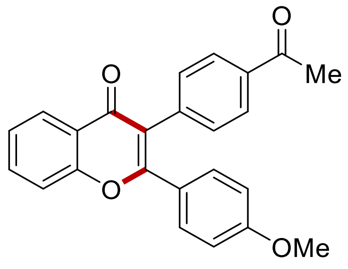

48

3-(4-Acetylphenyl)-2-(4-methoxyphenyl)-4*H*-chromen-4-one

The general procedure A was followed using **3** (78 µL, 0.75 mmol) and **10n** (62.5 mg, 0.25 mmol) at 100 °C for 3.5 h. Purification by column chromatography on silica gel (*n*hexane/EtOAc = 6/1 to 4/1) yielded isomer **48** (54.2 mg, 59%) as a white solid and its isomer **48'** (18.1 mg, 20%) as a white solid.

^{1}H-NMR (400 MHz, $CDCl_3$): δ = 8.26 (dd, J = 8.0, 1.7 Hz, 1H), 7.92–7.89 (m, 2H), 7.70 (ddd, J = 8.6, 7.1, 1.7 Hz, 1H), 7.53 (dd, J = 8.6, 1.1 Hz, 1H), 7.42 (ddd, J = 8.0, 7.1, 1.1 Hz, 1H), 7.37–7.34 (m, 2H), 7.34–7.31 (m, 2H), 6.79–6.76 (m, 2H), 3.78 (s, 3H), 2.59 (s, 3H). **^{13}C-NMR** (101 MHz, $CDCl_3$): δ = 198.1 (C_q), 176.9 (C_q), 161.9 (C_q), 161.3 (C_q), 156.0 (C_q), 138.9 (C_q), 136.0 (C_q), 133.9 (CH, C), 131.7 (CH), 131.4 (CH), 128.4 (CH), 126.4 (CH), 125.3 (CH), 125.0 (C_q), 123.4 (C_q), 121.1 (C_q), 118.0 (CH), 113.8 (CH), 55.4 (CH_3), 26.8 (CH_3). **IR** (ATR): $\tilde{\nu}$ = 1679, 1629, 1576, 1501, 1467, 1379, 1297, 1261, 1181, 1105 cm^{-1}. **M.p.**: 198–200 °C. **MS** (ESI) *m/z* (relative intensity): 371 (100) $[M+H]^+$, 763 (15) $[2M+Na]^+$. **HR-MS** (ESI) *m/z* calcd for $C_{24}H_{19}O_4$ $[M+H]^+$: 371.1283, found: 371.1278.

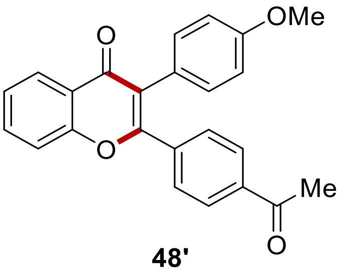

48'

2-(4-Acetylphenyl)-3-(4-methoxyphenyl)-4*H*-chromen-4-one

^{1}H-NMR (400 MHz, $CDCl_3$): δ = 8.29 (dd, J = 8.1, 1.7 Hz, 1H), 7.88–7.85 (m, 2H), 7.72 (ddd, J = 8.6, 7.1, 1.7 Hz, 1H), 7.54 (d, J = 8.6 Hz, 1H), 7.53–7.51 (m, 2H), 7.44 (ddd, J = 8.1, 7.1, 1.1 Hz, 1H), 7.14–7.11 (m, 2H), 6.87–6.83 (m, 2H), 3.81 (s, 3H), 2.59 (s, 3H). **^{13}C-NMR** (101 MHz, $CDCl_3$): δ = 197.5 (C_q), 177.6 (C_q), 159.9 (C_q), 159.4 (C_q), 156.1 (C_q), 138.0 (C_q), 137.7 (C_q), 134.0 (CH), 132.4 (CH), 130.0 (CH), 128.1 (CH), 126.6 (CH), 125.4 (CH), 124.5 (C_q), 123.6 (C_q), 123.4 (C_q), 118.1 (CH), 114.1 (CH), 55.4 (CH_3), 26.8 (CH_3). **IR** (ATR): $\tilde{\nu}$ = 1683, 1637, 1572, 1512, 1463, 1377, 1295, 1264, 1175, 1105 cm^{-1}. **M.p.**: 196–198 °C. **MS** (ESI) *m/z* (relative intensity): 371 (100) $[M+H]^+$, 763 (15) $[2M+Na]^+$. **HR-MS** (ESI) *m/z* calcd for $C_{24}H_{19}O_4$ $[M+H]^+$: 371.1283, found: 371.1279.

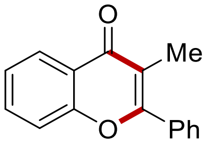

49

3-Methyl-2-phenyl-4*H*-chromen-4-one

The general procedure A was followed using **3** (78 µL, 0.75 mmol) and **10o** (29.1 mg, 0.25 mmol) at 100 °C for 7 h. Purification by column chromatography on silica gel (*n*hexane/EtOAc = 15/1) yielded **49** (32.8 mg, 55%) as a colorless oil. **^{1}H-NMR** (300 MHz, $CDCl_3$): δ = 8.26 (dd, J = 8.0, 1.7 Hz, 1H), 7.68–7.61 (m, 3H), 7.55–7.49 (m, 3H), 7.45 (dd, J = 8.5, 1.1 Hz, 1H), 7.39 (ddd, J = 8.0, 7.1, 1.1 Hz, 1H), 2.17 (s, 3H). **^{13}C-NMR** (101 MHz, $CDCl_3$): δ = 179.0 (C_q), 161.1 (C_q), 156.3 (C_q), 133.6 (C_q), 133.5 (CH), 130.3 (CH), 129.1 (CH), 128.6 (CH), 126.0 (CH), 124.8 (CH), 122.6 (C_q), 118.0 (CH), 117.7 (C_q), 11.9 (CH_3). **IR** (ATR): $\tilde{\nu}$ = 1631, 1571, 1467, 1380, 1230, 1131, 1015, 761, 697 cm^{-1}. **MS** (ESI) *m/z* (relative intensity): 237 (100) $[M+H]^+$, 259 (20) $[M+Na]^+$, 495 (40) $[2M+Na]^+$. **HR-MS** (ESI) *m/z* calcd for $C_{16}H_{13}O_2$ $[M+H]^+$: 237.0910, found: 237.0914.

The spectral data are in accordance with those reported in the literature.[5]

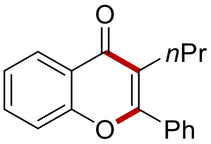

50

3-Propyl-2-phenyl-4*H*-chromen-4-one

The general procedure A was followed using **3** (78 µL, 0.75 mmol) and **10p** (36.1 mg, 0.25 mmol) at 100 °C for 3.5 h. Purification by column chromatography on silica gel (*n*hexane/EtOAc = 35/1 to 25/1) yielded **50** (37.4 mg, 57%) as a colorless oil.

^{1}H-NMR (400 MHz, $CDCl_3$): δ = 8.25 (ddd, J = 8.0, 1.7, 0.5 Hz, 1H), 7.64 (ddd, J = 8.7, 7.1, 1.7 Hz, 1H), 7.61–7.58 (m, 2H), 7.54–7.50 (m, 3H), 7.44–7.41 (m, 1H), 7.39 (ddd, J = 8.1, 7.1, 1.1 Hz, 1H), 2.56–2.50 (m, 2H), 1.65–1.53 (m, 2H), 0.90 (t, J = 7.4 Hz, 3H). **^{13}C-NMR** (101 MHz, $CDCl_3$): δ = 178.6 (C_q), 161.9 (C_q), 156.3 (C_q), 133.8 (C_q), 133.4 (CH), 130.2 (CH), 128.7 (CH), 128.6 (CH), 126.0 (CH), 124.8 (CH), 123.1 (C_q), 122.3 (C_q), 118.0 (CH), 27.9 (CH_2), 22.6 (CH_2), 14.3 (CH_3). **IR** (ATR): $\tilde{\nu}$ = 2960, 1629, 1569, 1464, 1379, 1224, 1129, 1111, 760, 698 cm^{-1}. **MS** (ESI) *m/z* (relative intensity): 265 (100) $[M+H]^+$, 287 (30) $[M+Na]^+$, 551 (70) $[2M+Na]^+$. **HR-MS** (ESI) *m/z* calcd for $C_{18}H_{17}O_2$ $[M+H]^+$: 265.1223, found: 265.1223.

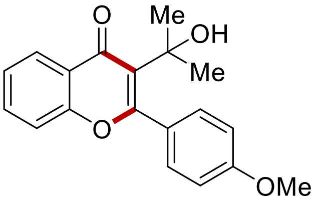

51

3-(2-Hydroxypropan-2-yl)-2-(4-methoxyphenyl)-4*H*-chromen-4-one

The general procedure A was followed using **3** (78 µL, 0.75 mmol) and **10q** (47.5 mg, 0.25 mmol) at 100 °C for 7 h. Purification by column chromatography on silica gel (*n*hexane/EtOAc = 6/1 to 5/1) yielded **51** (43.4 mg, 56%) as a yellow solid.

^{1}H-NMR (400 MHz, $CDCl_3$): δ = 8.24 (dd, J = 7.9, 1.7 Hz, 1H), 7.67 (ddd, J = 8.7, 7.1, 1.8 Hz, 1H), 7.43–7.40 (m, 1H), 7.39–7.36 (m, 3H), 7.04–6.97 (m, 2H), 6.44 (s, 1H), 3.88 (s, 3H), 1.32 (s, 6H). **^{13}C-NMR** (101 MHz, $CDCl_3$): δ = 181.3 (C_q), 162.4 (C_q), 161.1 (C_q), 155.5 (C_q), 134.2 (CH), 130.2 (CH), 127.8 (C_q), 127.5 (C_q), 126.1 (CH), 125.2 (CH), 123.1 (C_q), 117.8 (CH), 113.9 (CH), 72.6 (C_q), 55.5 (CH_3), 31.1 (CH_3). **IR** (ATR): $\tilde{\nu}$ = 1612, 1582, 1461, 1364, 1248, 1172, 1064, 1025, 763, 620 cm^{-1}. **M.p.**: 147–149 °C. **MS** (ESI) *m/z* (relative intensity): 311 (10) $[M+H]^+$, 333 (90) $[M+Na]^+$, 643 (100) $[2M+Na]^+$. **HR-MS** (ESI) *m/z* calcd for $C_{19}H_{19}O_4$ $[M+H]^+$: 311.1278, found: 311.1274.

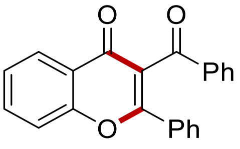

52

3-Acetyl-2-phenyl-4*H*-chromen-4-one

The general procedure A was followed using **3** (78 µL, 0.75 mmol) and **10r** (51.6 mg, 0.25 mmol) at 100 °C for 3.5 h. Purification by column chromatography on silica gel (*n*hexane/EtOAc = 8/1 to 4/1) yielded **52** as (42.7 mg, 52%) as a yellowish oil.

^{1}H-NMR (400 MHz, $CDCl_3$): δ = 8.24 (dd, J = 8.0, 1.7 Hz, 1H), 7.92 (d, J = 7.9 Hz, 2H), 7.75 (dd, J = 6.9, 1.7 Hz, 1H), 7.66 (d, J = 7.2 Hz, 2H), 7.59 (d, J = 8.4 Hz, 1H), 7.52 (dd, J = 7.4, 7.4 Hz, 1H), 7.46 (dd, J = 7.7, 7.7 Hz, 1H), 7.44–7.32 (m, 5H).**^{13}C-NMR** (101 MHz, $CDCl_3$): δ = 193.6 (C_q), 176.6 (C_q), 162.6 (C_q), 156.2 (C_q), 137.1 (C_q), 134.5 (CH), 133.9 (CH), 131.9 (C_q), 131.6 (CH), 129.5 (CH), 128.9 (CH), 128.8 (CH), 128.6 (CH), 126.2 (CH), 125.8 (CH), 123.4 (C_q), 122.7 (C_q), 118.3 (CH). **IR** (ATR): $\tilde{\nu}$ = 1674, 1632, 1564, 1464, 1375, 1225, 1117, 859, 762, 694 cm^{-1}. **MS** (ESI) *m/z* (relative intensity): 327 (100) $[M+H]^+$, 349 (10) $[M+Na]^+$, 670 (30) $[2M+NH_4]^+$. **HR-MS** (ESI) *m/z* calcd for $C_{22}H_{14}O_3$ $[M+H]^+$: 327.1016, found: 327.1016.

The spectral data are in accordance with those reported in the literature.[24]

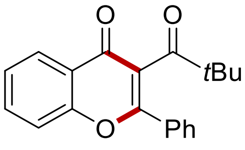

53

2-Phenyl-3-pivaloyl-4*H*-chromen-4-one

The general procedure A was followed using **3** (78 µL, 0.75 mmol), **10s** (46.6 mg, 0.25 mmol) and $[Cp^*RhCl_2]_2$ (7.7 mg, 5.0 mol %) at 80 °C for 3.5 h. Purification by column chromatography on silica gel (*n*hexane/EtOAc = 15/1 to 10/1) yielded **53** as a 5:1 mixture of isomers (51.0 mg, 67%) appearing as a brownish oil.

^{1}H-NMR (300 MHz, $CDCl_3$): δ = 8.27 (dd, J = 7.9, 1.7 Hz, 0.16H), 8.21 (dd, J = 8.0, 1.7 Hz, 0.84H), 7.74–7.65 (m, 3H), 7.56–7.42 (m, 4H), 7.41–7.29 (m, 1H), 1.07 (s, 1.42H), 0.96 (s, 7.59H). **IR** (ATR): $\tilde{\nu}$ = 1698, 1636, 1566, 1466, 1370, 1224, 914, 875, 761, 699 cm^{-1}. **MS** (ESI) *m/z* (relative intensity): 307 (100) $[M+H]^+$, 329 (10) $[M+Na]^+$, 630 (25) $[2M+NH_4]^+$. **HR-MS** (ESI) *m/z* calcd for $C_{20}H_{18}O_3$ $[M+H]^+$: 307.1329, found: 307.1330.

Major isomer:

^{13}C-NMR (126 MHz, $CDCl_3$): δ = 210.5 (C_q), 176.6 (C_q), 159.8 (C_q), 156.2 (C_q), 134.3 (CH), 132.9 (C_q), 131.5 (CH), 129.2 (CH), 128.9 (CH), 126.0 (CH), 125.5 (CH), 124.7 (C_q), 123.1 (C_q), 118.2 (CH), 46.0 (C_q), 27.0 (CH_3).

Minor isomer:
^{13}C-NMR (126 MHz, $CDCl_3$): δ = 204.3 (C_q), 176.6 (C_q), 158.6 (C_q), 155.4 (C_q), 134.3 (CH), 131.0 (CH), 130.3 (C_q), 128.6 (CH), 128.4 (CH), 126.5 (CH), 125.8 (CH), 124.0 (C_q), 121.9 (C_q), 118.1 (CH), 44.4 (C_q), 26.6 (CH_3).

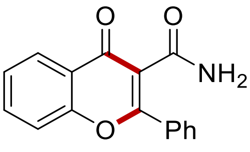

54

4-Oxo-2-phenyl-4*H*-chromene-3-carboxamide

The general procedure A was followed using **3** (78 µL, 0.75 mmol), **10t** (36.3 mg, 0.25 mmol) and $[Cp^*RhCl_2]_2$ (7.7 mg, 5.0 mol %) at 100 °C for 3.5 h. Purification by column chromatography on silica gel (*n*hexane/EtOAc = 1/1 to 1/3) yielded **54** (32.8 mg, 49%) as a white solid.

^{1}H-NMR (400 MHz, $CDCl_3$): δ = 8.26 (d, J = 7.9 Hz, 1H), 8.03 (brs, 1H), 7.77–7.70 (m, 3H), 7.59–7.43 (m, 5H), 5.84 (brs, 1H). **^{13}C-NMR** (101 MHz, $CDCl_3$): δ = 177.2 (C_q), 169.2 (C_q), 165.7 (C_q), 155.6 (C_q), 134.7 (CH), 133.4 (C_q), 131.4 (CH), 128.8 (CH), 128.5 (CH), 126.3 (CH), 126.1 (CH), 123.4 (C_q), 118.2 (CH), 116.2 (C_q). **IR** (ATR): $\tilde{\nu}$ = 3321, 1671, 1624, 1564, 1466, 1394, 1224, 1103, 760, 697 cm^{-1}. **M.p.**: 220–222 °C. **MS** (ESI) *m/z* (relative intensity): 266 (100) $[M+H]^+$, 288 (30) $[M+Na]^+$, 553 (30) $[2M+Na]^+$. **HR-MS** (ESI) *m/z* calcd for $C_{16}H_{11}NO_3$ $[M+H]^+$: 266.0812, found: 266.0815.

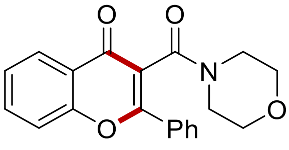

55

3-(Morpholine-4-carbonyl)-2-phenyl-4*H*-chromen-4-one

The general procedure A was followed using **3** (78 µL, 0.75 mmol), **10u** (53.8 mg, 0.25 mmol) and $[Cp^*RhCl_2]_2$ (7.7 mg, 5.0 mol %) at 80 °C for 3.5 h. Purification by column

chromatography on silica gel (*n*hexane/EtOAc = 1/1 to 1/3) yielded **55** (57.8 mg, 69%) as a brownish solid.

^{1}H-NMR (400 MHz, $CDCl_3$): δ = 8.21 (d, *J* = 7.9 Hz, 1H), 7.83 (d, *J* = 7.5 Hz, 2H), 7.71 (dd, *J* = 7.8 Hz, 7.8 Hz,1H), 7.55–7.47 (m, 4H), 7.42 (dd, *J* = 7.6 Hz, 7.6 Hz 1H), 3.79–3.66 (m, 3H), 3.54 (ddd, *J* = 11.2, 6.3, 3.0 Hz, 1H), 3.51–3.44 (m, 1H), 3.33 (ddd, *J* = 13.4, 6.9, 3.0 Hz, 1H), 3.13 (ddd, *J* = 13.4, 6.3, 3.1 Hz, 1H), 2.99 (ddd, *J* = 10.5, 6.9, 3.0 Hz, 1H).

^{13}C-NMR (101 MHz, $CDCl_3$): δ = 175.4 (C_q), 163.9 (C_q), 161.0 (C_q), 156.1 (C_q), 134.4 (CH), 131.8 (CH), 131.8 (C_q), 129.0 (CH), 128.2 (CH), 126.1 (CH), 125.7 (CH), 123.1 (C_q), 118.8 (C_q), 118.1 (CH), 66.5 (CH_2), 66.4 (CH_2), 47.1 (CH_2), 42.1 (CH_2). **IR** (ATR): $\tilde{\nu}$ = 1629, 1617, 1565, 1462, 1436, 1375, 1111, 1018, 758, 696 cm^{-1}. **M.p.**: 178–180 °C. **MS** (ESI) *m/z* (relative intensity): 336 (100) $[M+H]^+$, 358 (5) $[M+Na]^+$. **HR-MS** (ESI) *m/z* calcd for $C_{20}H_{17}NO_4$ $[M+H]^+$: 336.1230, found: 336.1233.

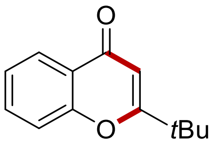

56

2-(*tert*-Butyl)-4*H*-chromen-4-one

The general procedure A was followed using **3** (156 µL, 1.50 mmol), **10v** (41.1 mg, 0.50 mmol), $[Cp^*RhCl_2]_2$ (7.7 mg, 2.5 mol %) and NaOPiv (124 mg, 1.00 mmol) at rt for 7 h. Purification by column chromatography on silica gel (*n*hexane/EtOAc = 10/1 to 4/1) yielded **56** (61.5 mg, 61%) as a brown oil.

^{1}H-NMR (400 MHz, $CDCl_3$): δ = 8.15 (dd, *J* = 8.0, 1.8 Hz, 1H), 7.66–7.57 (m, 1H), 7.42 (d, *J* = 8.4 Hz, 1H), 7.34 (dd, *J* = 7.5 Hz, 1H), 6.26 (s, 1H), 1.33 (s, 9H). **^{13}C-NMR** (101 MHz, $CDCl_3$): δ = 179.0 (C_q), 176.2 (C_q), 156.5 (C_q), 133.6 (CH), 125.6 (CH), 124.9 (CH), 123.5 (C_q), 117.9 (CH), 106.7 (CH), 36.6 (C_q), 27.9 (CH_3). **IR** (ATR): $\tilde{\nu}$ = 1647, 1462, 1355, 1229, 1133, 1077, 937, 852, 779, 757 cm^{-1}. **MS** (ESI) *m/z* (relative intensity): 203 (100) $[M+H]^+$, 225 (15) $[M+Na]^+$, 427 (20) $[2M+Na]^+$. **HR-MS** (ESI) *m/z* calcd for $C_{13}H_{14}O_2$ $[M+H]^+$: 203.1067, found: 203.1056.

The spectral data are in accordance with those reported in the literature.[25]

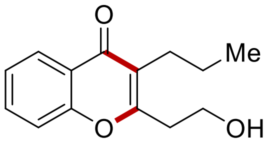

57

2-(2-Hydroxyethyl)-3-propyl-4*H*-chromen-4-one

The general procedure A was followed using **3** (78 µL, 0.75 mmol) and **10w** (30.6 mg, 0.25 mmol) at 100 °C for 7 h. Purification by column chromatography on silica gel (*n*hexane/EtOAc = 6/1) yielded **49** (37.4 mg, 64%) as a white solid.

^{1}H-NMR (400 MHz, $CDCl_3$): δ = 8.10 (dd, *J* = 8.1, 1.7, 1H), 7.56 (ddd, *J* = 8.6, 7.1, 1.7 Hz, 1H), 7.33 (ddd, *J* = 8.6, 1.1, 0.5 Hz, 1H), 7.29 (ddd, *J* = 8.1, 7.1, 1.1 Hz, 1H), 4.06 (t, *J* = 6.5 Hz, 2H), 2.99 (t, *J* = 6.4 Hz, 2H), 2.55–2.48 (m, 2H), 1.57–1.44 (m, 2H), 0.96 (t, *J* = 7.3 Hz, 3H). **^{13}C-NMR** (101 MHz, $CDCl_3$): δ = 178.0 (C_q), 162.7 (C_q), 156.0 (C_q), 133.2 (CH), 126.0 (CH), 124.7 (CH), 122.9 (C_q), 122.8 (C_q), 117.7 (CH), 60.2 (CH_2), 35.4 (CH_2), 26.7 (CH_2), 22.6 (CH_2), 14.3 (CH_3). **IR** (ATR): $\tilde{\nu}$ = 3370, 2918, 1611, 1564, 1464, 1394, 1159, 1059, 757, 656 cm^{-1}. **M.p.**: 104–105 °C. **MS** (ESI) *m/z* (relative intensity): 233 (80) $[M+H]^+$, 255 (50) $[M+Na]^+$, 487 (100) $[2M+Na]^+$. **HR-MS** (ESI) *m/z* calcd for $C_{14}H_{17}O_3$ $[M+H]^+$: 233.1172, found: 233.1174.

The spectral data are in accordance with those reported in the literature.[5]

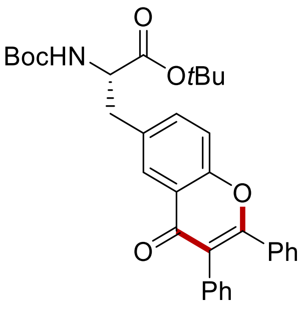

60

***tert*-Butyl (*S*)-2-[(*tert*-butoxycarbonyl)amino]-3-(4-oxo-2,3-diphenyl-4*H*-chromen-6-yl)propanoate**

The general procedure A was followed using **58a** (183 mg, 0.50 mmol) and **4** (44.5 mg, 0.25 mmol) at 100 °C for 3.5 h. Purification by column chromatography on silica gel (*n*hexane/EtOAc = 6/1 to 4/1) yielded **60** (112 mg, 82%) as a white solid.

^{1}H-NMR (600 MHz, $CDCl_3$): δ = 7.98 (d, *J* = 2.2 Hz, 1H), 7.49–7.45 (m, 1H), 7.39 (d, *J* = 8.5 Hz, 1H), 7.31–7.27 (m, 2H), 7.26–7.21 (m, 2H), 7.20–7.16 (m, 4H), 7.13–7.11 (m, 2H), 5.03 (d, *J* = 7.8 Hz, 1H), 4.41 (ddd, *J* = 7.8, 5.8, 5.7 Hz, 1H), 3.18 (dd, *J* = 13.9, 5.7 Hz, 1H), 3.08 (dd, *J* = 13.9, 5.8 Hz, 1H), 1.38 (s, 9H), 1.35 (s, 9H). **^{13}C-NMR** (151 MHz, $CDCl_3$): δ = 177.1 (C_q), 170.6 (C_q), 161.5 (C_q), 155.2 (C_q), 155.2 (C_q), 135.2 (CH), 133.8 (C_q), 133.4 (C_q), 133.0 (C_q), 131.3 (CH), 130.1 (CH), 129.6 (CH), 128.3 (CH), 128.1 (CH), 127.7 (CH), 126.8 (CH), 123.3 (C_q), 123.0 (C_q), 118.1 (CH), 82.7 (C_q), 79.9 (C_q), 54.9 (CH), 38.0 (CH_2), 28.4 (CH_3), 28.1 (CH_3). **IR** (ATR): $\tilde{v}$ = 1689, 1636, 1614, 1444, 1364, 1239, 1152, 1052, 698 cm^{-1}. **M.p.**: 145–147 °C. **MS** (ESI) *m/z* (relative intensity): 542 (70) $[M+H]^+$, 564 (100) $[M+Na]^+$, 1105 (80) $[2M+Na]^+$. **HR-MS** (ESI) *m/z* calcd for $C_{33}H_{36}NO_6$ $[M+H]^+$: 542.2537, found: 542.2521.

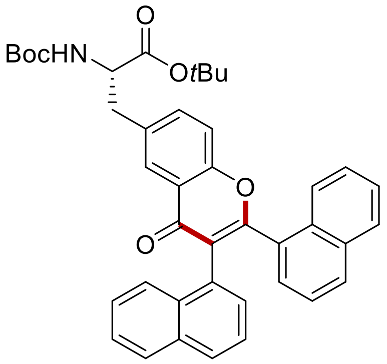

61

***tert*-Butyl (*S*)-2-[(*tert*-butoxycarbonyl)amino]-3-(2,3-di(naphthalen-1-yl)-4-oxo-4*H*-chrom-en-6-yl)propanoate**

The general procedure A was followed using **58a** (183 mg, 0.50 mmol) and **10j** (69.6 mg, 0.25 mmol) at 100 °C for 7 h. Purification by column chromatography on silica gel (CH_2Cl_2/EtOAc = 50/1 to 20/1) yielded **61** (119 mg, 74%) as a white solid.

^{1}H-NMR (400 MHz, $CDCl_3$): δ = 8.08 (dd, J = 4.2, 2.2 Hz, 1H), 7.88 (dd, J = 8.4, 4.2, 1H), 7.77–7.70 (m, 1H), 7.68–7.61 (m, 2H), 7.57 (d, J = 8.0, 1H), 7.53–7.46 (m, 2H), 7.44–7.33 (m, 3H), 7.30 (dd, J = 6.8, 3.4 Hz, 2H), 7.05 (dd, J = 6.1, 6.1 Hz, 2H), 7.02–6.91 (m, 3H), 5.06 (d, J = 5.7 Hz, 1H), 4.45 (ddd, J = 6.4, 5.7 Hz), 3.28–3.17 (m, 1H), 3.16–3.06 (m, 1H), 1.40 (s, 9H), 1.36 (m, 9H). **^{13}C-NMR** (101 MHz, $CDCl_3$): δ = 177.1 (C_q), 170.6 (C_q), 163.6 (C_q), 155.6 (C_q), 155.2 (C_q), 135.5 (CH), 134.1 (C_q), 133.5 (C_q), 133.3 (C_q), 132.6 (C_q), 131.0 (C_q), 130.9 (C_q), 130.7 (C_q), 130.3 (CH), 128.5 (CH), 128.5 (CH), 128.4 (CH), 128.2 (CH), 127.6 (CH), 127.1 (CH), 127.0 (CH), 126.3 (CH), 126.1 (CH), 125.7 (CH), 125.6 (CH), 125.2 (CH), 125.1 (CH), 124.8 (C_q), 124.6 (CH), 123.4 (C_q), 118.3 (CH), 82.8 (C_q), 79.9 (C_q), 55.0 (CH), 38.1 (CH_2), 28.4 (CH_3), 28.2 (CH_3). **IR** (ATR): $\tilde{\nu}$ = 1708, 1643, 1616, 1485, 1360, 1231, 1148, 800, 772, 750 cm^{-1}. **M.p.**: 122–124 °C. **MS** (ESI) *m/z* (relative intensity): 642 (20) $[M+H]^+$, 664 (100) $[M+Na]^+$, 1305 (70) $[2M+Na]^+$. **HR-MS** (ESI) *m/z* calcd for $C_{41}H_{40}NO_6$ $[M+H]^+$: 642.2850, found: 642.2879.

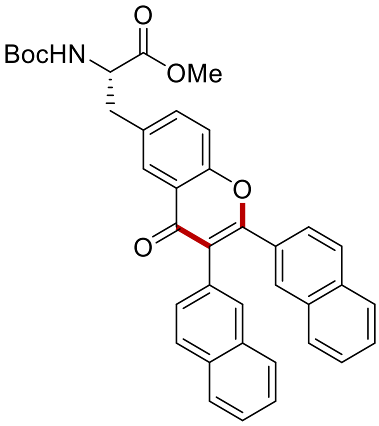

62

Methyl (*S*)-2-[(*tert*-butoxycarbonyl)amino]-3-(2,3-di(naphthalen-2-yl)-4-oxo-4*H*-chromen-6-yl)propanoate

The general procedure B was followed using **58b** (169 mg, 0.50 mmol) and **10i** (72.4 mg, 0.25 mmol) at 100 °C for 3.5 h. Purification by column chromatography on silica gel (*n*hexane/EtOAc = 4/1 to 2/1) yielded **62** (135 mg, 87%) as a white solid.

^{1}H-NMR (400 MHz, $CDCl_3$): δ = 8.00–7.95 (m, 1H), 7.72–7.68 (m, 1H), 7.66 (d, *J* = 7.9 Hz, 1H), 7.62 (d, *J* = 8.5 Hz, 1H), 7.60–7.55 (m, 3H), 7.44–7.39 (m, 3H), 7.37–7.25 (m, 4H), 7.20 (dd, *J* = 8.5, 1.8 Hz, 1H), 7.16 (dd, *J* = 8.7, 1.8 Hz, 1H), 5.08 (d, *J* = 8.0 Hz, 1H), 4.55 (ddd, *J* = 8.0, 6.3, 5.3 Hz, 1H), 3.65 (s, 3H), 3.19 (dd, *J* = 13.9, 5.3 Hz, 1H), 3.08 (dd, *J* = 13.9, 6.3 Hz, 1H), 1.33 (s, 9H). **^{13}C-NMR** (101 MHz, $CDCl_3$): δ = 177.3 (C_q), 172.0 (C_q), 161.6 (C_q), 155.3 (C_q), 155.2 (C_q), 135.0 (CH), 133.6 (C_q), 133.6 (C_q), 133.4 (C_q), 132.8 (C_q), 132.5 (C_q), 130.7 (CH), 130.6 (C_q), 130.3 (C_q), 130.0 (CH), 129.0 (CH), 128.8 (CH), 128.2 (CH), 127.9 (CH), 127.7 (CH). (One aromatic CH is missing due to overlap, the overlap was verified by HSQC, showing the peak at 127.7 corresponds to two carbons). 127.7 (CH). (One aromatic CH is missing due to overlap, the overlap was verified by HSQC, showing the peak at 127.7 corresponds to two carbons). 126.6 (CH), 126.6 (CH), 126.2 (CH), 126.1 (CH), 125.9 (CH), 123.4 (C_q), 123.0 (C_q), 118.4 (CH), 80.1 (C_q), 54.6 (CH), 52.5 (CH_3), 37.9 (CH_2), 28.3 (CH_3). **IR** (ATR): $\tilde{\nu}$ = 1706, 1617, 1487,1442, 1364, 1214, 1162, 821, 746, 474 cm^{-1}. **M.p.**: 137–139 °C. **MS** (ESI) *m/z* (relative intensity): 600 (40) $[M+H]^+$, 622 (40) $[M+Na]^+$, 1221 (100) $[2M+Na]^+$. **HR-MS** (ESI) *m/z* calcd for $C_{38}H_{33}NO_6Na$ $[M+Na]^+$: 622.2206, found: 622.2172.

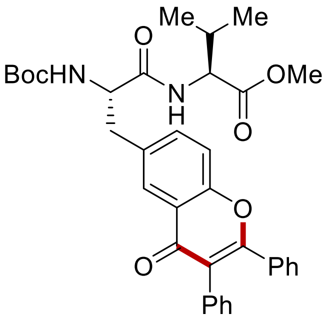

63

Methyl {(*S*)-2-[(*tert*-butoxycarbonyl)amino]-3-(4-oxo-2,3-diphenyl-4*H*-chromen-6-yl)propanoyl}-*L*-valinate

The general procedure B was followed using **58c** (211 mg, 0.50 mmol) and **4** (44.5 mg, 0.25 mmol) at 100 °C for 7 h. Purification by column chromatography on silica gel (*n*hexane/EtOAc = 4/1 to 1/1) yielded **63** (141 mg, 94%) as a white solid.

^{1}H-NMR (400 MHz, $CDCl_3$): δ = 8.00 (d, J = 2.3 Hz, 1H), 7.50 (d, J = 2.3 Hz, 1H), 7.37 (dd, J = 8.5, 2.3 Hz, 1H), 7.30–7.26 (m, 2H), 7.25–7.15 (m, 6H), 7.14–7.10 (m, 2H), 6.66 (d, J = 8.5 Hz, 1H), 5.23 (d, J = 8.0 Hz, 1H), 4.44 (ddd, J = 8.6, 5.1 Hz, 1H), 4.39–4.31 (ddd, J = 8.0, 7.8, 5.9 Hz, 1H), 3.62 (s, 3H), 3.20 (dd, J = 14.0, 5.9 Hz, 1H), 3.04 (dd, J = 14.0, 7.8 Hz, 1H), 2.14–1.99 (m, 1H), 1.32 (s, 9H), 0.83 (d, J = 6.8 Hz, 2H), 0.80 (d, J = 7.7 Hz, 3H). **^{13}C-NMR** (101 MHz, $CDCl_3$): δ = 177.2 (C_q), 172.0 (C_q), 171.1 (C_q), 161.5 (C_q), 155.6 (C_q), 155.2 (C_q), 135.0 (CH), 134.2 (C_q), 133.3 (C_q), 133.0 (C_q), 131.3 (CH), 130.1 (CH), 129.6 (CH), 128.3 (CH), 128.1 (CH), 127.7 (CH), 126.7 (CH), 123.4 (C_q), 123.0 (C_q), 118.4 (CH), 77.4 (C_q), 57.3 (CH), 55.8 (CH), 52.2 (CH_3), 37.5 (CH_2), 31.3 (CH), 28.3 (CH_3), 19.0 (CH_3), 17.9 (CH_3). **IR** (ATR): $\tilde{\nu}$ = 3307, 1646, 1617, 1520, 1487, 1443, 1368, 1228, 1163, 694 cm^{-1}. **M.p.**: 110–111 °C. **MS** (ESI) *m/z* (relative intensity): 599 (50) $[M+H]^+$, 621 (100) $[M+Na]^+$, 1219 (40) $[2M+Na]^+$. **HR-MS** (ESI) *m/z* calcd for $C_{35}H_{39}N_2O_7$ $[M+H]^+$: 599.2752, found: 599.2745.

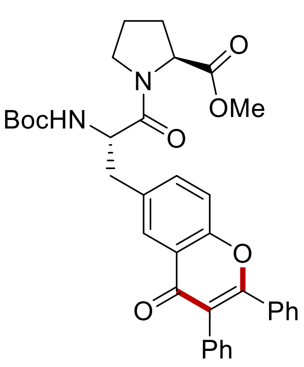

64

Methyl {(*S*)-2-[(*tert*-butoxycarbonyl)amino]-3-(4-oxo-2,3-diphenyl-4*H*-chromen-6-yl)propanoyl}-*L*-prolinate

The general procedure B was followed using **58d** (168 mg, 0.40 mmol) and **4** (35.6 mg, 0.20 mmol) at 100 °C for 3.5 h. Purification by column chromatography on silica gel (CH_2Cl_2/MeOH = 98/2) yielded **64** (92.5 mg, 76%) as a white solid.

^{1}H-NMR (400 MHz, $CDCl_3$): δ = 8.05 (d, J = 2.3 Hz, 1H), 7.57 (dd, J = 8.5, 2.2 Hz, 1H), 7.40 (d, J = 8.7 Hz, 1H), 7.32–7.28 (m, 2H), 7.26–7.22 (m, 2H), 7.20–7.16 (m, 4H), 7.15–7.11 (m, 2H), 5.24 (d, J = 8.8 Hz, 1H), 4.64 (ddd, J = 8.8, 7.1, 5.5 Hz, 1H), 4.60–4.53 (m, 1H), 3.68 (s, 3H), 3.51–3.42 (m, 1H), 3.27 (dd, J = 13.9, 5.5 Hz, 1H), 3.02 (dd, J = 13.9, 7.1 Hz, 1H), 2.27 –2.17 (m, 1H), 2.10–1.97 (m, 2H), 1.92–1.80 (m, 1H), 1.29 (s, 9H). **^{13}C-NMR** (101 MHz, $CDCl_3$): δ = 177.2 (C_q), 172.5 (C_q), 170.4 (C_q), 161.4 (C_q), 155.3 (C_q), 155.2 (C_q), 135.6 (CH), 133.8 (C_q), 133.4 (C_q), 133.0 (C_q), 131.3 (CH), 130.1 (CH), 129.6 (CH), 128.3 (CH), 128.1 (CH), 127.6 (CH), 126.9 (CH), 123.3 (C_q), 123.0 (C_q), 118.1 (CH), 79.9 (C_q), 59.0 (CH_3), 53.1 (CH), 52.4 (CH), 47.1 (CH_2), 38.3 (CH_2), 29.1 (CH_2), 28.3 (CH_3), 25.0 (CH_2). **IR** (ATR): $\tilde{\nu}$ = 1705, 1641, 1488, 1439, 1368, 1228, 1167, 753, 697 cm^{-1}. **M.p.**: 109–111 °C. **MS** (ESI) *m/z* (relative intensity): 597 (30) $[M+H]^+$, 619 (100) $[M+Na]^+$, 1215 (50) $[2M+Na]^+$. **HR-MS** (ESI) *m/z* calcd for $C_{35}H_{37}N_2O_7$ $[M+H]^+$: 597.2595, found: 597.2692.

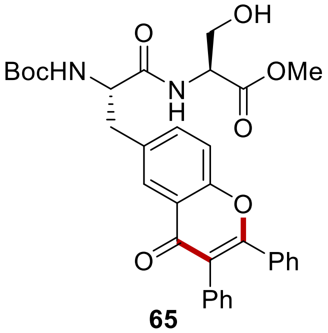

65

Methyl {(*S*)-2-[(*tert*-butoxycarbonyl)amino]-3-(4-oxo-2,3-diphenyl-4*H*-chromen-6-yl)propanoyl}-*L*-serinate

The general procedure B was followed using **58e** (80.0 mg, 0.20 mmol) and **4** (17.5 mg, 0.10 mmol) at 100 °C for 3.5 h. Purification by column chromatography on silica gel (CH_2Cl_2/MeOH = 98/2 to 95/5) yielded **65** (48.4 mg, 84%) as a white solid.

^{1}H-NMR (600 MHz, $CDCl_3$): δ = 8.00 (d, J = 2.3 Hz, 1H), 7.51 (dd, J = 8.6, 2.3 Hz, 1H), 7.40 (d, J = 8.6 Hz, 1H), 7.32 (d, J = 7.4 Hz, 1H), 7.28–7.26 (m, 2H), 7.26–7.23 (m, 1H), 7.23–7.19 (m, 3H), 7.18–7.15 (m, 2H), 7.11–7.09 (m, 2H), 5.30 (d, J = 7.9 Hz, 1H), 4.56 (ddd, J = 7.4, 7.2, 7.2 Hz, 1H), 4.46 (ddd, J = 7.9, 6.4, 6.4 Hz, 1H), 3.93–3.84 (m, 2H), 3.84–3.79 (m, 1H), 3.67 (s, 3H), 3.18 (d, J = 6.4 Hz, 2H), 1.30 (s, 9H). **^{13}C-NMR** (151 MHz, $CDCl_3$): δ = 177.8 (C_q), 171.5 (C_q), 170.9 (C_q), 161.9 (C_q), 155.6 (C_q), 155.3 (C_q), 135.6 (CH), 134.1 (C_q), 133.2 (C_q), 132.8 (C_q), 131.3 (CH), 130.2 (CH), 129.6 (CH), 128.4 (CH), 128.2 (CH), 127.8 (CH), 126.4 (CH), 123.3 (C_q), 123.0 (C_q), 118.5 (CH), 80.5 (C_q), 62.5 (CH_2), 55.6 (CH_3), 55.1 (CH), 52.8 (CH), 37.7 (CH_2), 28.3 (CH_3). **IR** (ATR): $\tilde{\nu}$ = 3305, 1642, 1619, 1488, 1444, 1370, 1223, 1165, 750, 695 cm^{-1}. **M.p.**: 120–122 °C. **MS** (ESI) *m/z* (relative intensity): 587 (50) $[M+H]^+$, 609 (100) $[M+Na]^+$, 1195 (60) $[2M+Na]^+$. **HR-MS** (ESI) *m/z* calcd for $C_{33}H_{35}N_2O_8$ $[M+H]^+$: 587.2388; found: 587.2586.

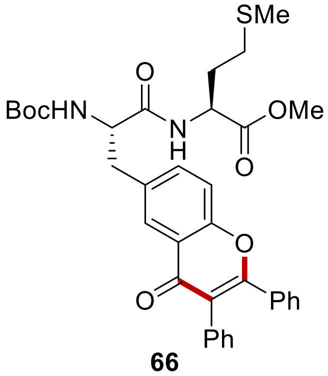

66

Methyl {(*S*)-2-[(*tert*-butoxycarbonyl)amino]-3-(4-oxo-2,3-diphenyl-4*H*-chromen-6-yl)propanoyl}-*L*-methioninate

The general procedure B was followed using **58f** (112 mg, 0.25 mmol) and **4** (22.6 mg, 0.13 mmol) at 100 °C for 3.5 h. Purification by column chromatography on silica gel (*n*hexane/EtOAc = 4/2 to 4/3) yielded **66** (48.8 mg, 60%) as a white solid.

^{1}H-NMR (400 MHz, $CDCl_3$): δ = 8.01 (d, *J* = 2.2 Hz, 1H), 7.51 (dd, *J* = 8.6, 2.2 Hz, 1H), 7.39 (d, *J* = 8.6 Hz, 1H), 7.31–7.26 (m, 2H), 7.26–7.15 (m, 6H), 7.14–7.10 (m, 2H), 6.85 (d, *J* = 7.8 Hz, 1H), 5.21 (d, *J* = 8.1 Hz, 1H), 4.60 (td, *J* = 7.8, 5.0, 5.0 Hz, 1H), 4.38 (ddd, *J* = 8.1, 7.5, 6.0 Hz, 1H), 3.65 (s, 3H), 3.19 (dd, *J* = 14.0, 6.0 Hz, 1H), 3.06 (dd, *J* = 14.0, 7.5 Hz, 1H), 2.37 (dd, *J* = 7.5, 7.5 Hz, 2H), 2.10–2.00 (m, 1H), 1.97 (s, 3H), 1.92–1.80 (m, 1H), 1.33 (s, 9H). **^{13}C-NMR** (101 MHz, $CDCl_3$): δ = 177.2 (C_q), 172.0 (C_q), 171.1 (C_q), 161.6 (C_q), 155.5 (C_q), 155.2 (C_q), 135.1 (CH), 134.1 (C_q), 133.3 (C_q), 133.0 (C_q), 131.3 (CH), 130.2 (CH), 129.6 (CH), 128.4 (CH), 128.2 (CH), 127.7 (CH), 126.7 (CH), 123.3 (C_q), 123.0 (C_q), 118.4 (CH), 80.5 (C_q), 55.7 (CH), 52.7 (CH_3), 51.7 (CH), 37.7 (CH_2), 31.6 (CH_2), 29.9 (CH_2), 28.3 (CH_3), 15.5 (CH_3). **IR** (ATR): $\tilde{\nu}$ = 3307, 1646, 1618, 1488, 1442, 1369, 1225, 1164, 751, 696 cm^{-1}. **M.p.**: 89–91 °C. **MS** (ESI) *m/z* (relative intensity): 631 (80) $[M+H]^+$, 653 (90) $[M+Na]^+$, 1283 (100) $[2M+Na]^+$. **HR-MS** (ESI) *m/z* calcd for $C_{35}H_{39}N_2O_7S$ $[M+H]^+$: 631.2472, found: 631.2451.

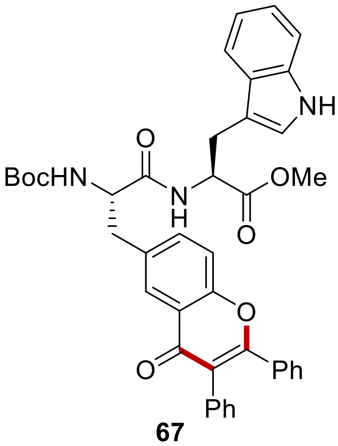

67

Methyl {(*S*)-2-[(*tert*-butoxycarbonyl)amino]-3-(4-oxo-2,3-diphenyl-4*H*-chromen-6-yl)propanoyl}-*L*-tryptophanate

The general procedure B was followed using **58g** (90.2 mg, 0.18 mmol), **4** (15.7 mg, 90 µmol) and $[Cp^*RhCl_2]_2$ (2.8 mg, 5.0 mol %) at 100 °C for 3.5 h. Purification by column chromatography on silica gel (*n*hexane/EtOAc = 2/1 to 1/1) yielded **67** (58.1 mg, 71%) as a white solid.

^{1}H-NMR (400 MHz, $CDCl_3$): δ = 8.65–8.34 (m, 1H), 7.99–7.84 (m, 1H), 7.48–7.39 (m, 1H), 7.36–7.31 (m, 1H), 7.30–7.15 (m, 10H), 7.15–7.03 (m, 3H), 6.99 (ddd, *J* = 7.2, 6.7, 6.7 Hz, 1H), 6.92–6.85 (m, 1H), 6.70 (d, *J* = 7.8 Hz, 1H), 5.14 (dd, *J* = 16.0, 8.0 Hz, 1H), 4.81 (ddd, *J* = 8.0, 5.5, 5.5 Hz, 1H), 4.46–4.29 (m, 1H), 3.53 (s, 3H), 3.23–3.14 (m, 2H), 3.14–3.02 (m, 1H), 2.95–2.75 (m, 1H), 1.26 (s, 9H). **^{13}C-NMR** (101 MHz, $CDCl_3$): δ = 177.4 (C_q), 172.2 (C_q), 170.9 (C_q), 161.7 (C_q), 155.5 (C_q), 155.2 (C_q), 136.2 (C_q), 135.2 (CH), 134.3 (C_q), 133.3 (C_q), 133.0 (C_q), 131.3 (CH), 130.2 (CH), 129.7 (CH), 128.4 (CH), 128.2 (CH), 127.8 (CH), 127.6 (C_q), 126.7 (CH), 123.4 (CH), 123.3 (C_q), 123.0 (C_q), 122.2 (CH), 119.6 (CH), 118.4 (CH), 111.6 (CH), 109.5 (C_q), 80.3 (C_q), 55.7 (CH), 53.0 (CH_3), 52.5 (CH), 38.1 (CH_2), 28.3 (CH_3), 27.7 (CH_2). **IR** (ATR): $\tilde{\nu}$ = 3316, 1664, 1618, 1489, 1444, 1370, 1226, 1166, 747, 696 cm^{-1}. **M.p.**: 147–149 °C. **MS** (ESI) *m/z* (relative intensity): 686 (100) $[M+H]^+$, 708 (100) $[M+Na]^+$, 1388 (30) $[2M+Na]^+$. **HR-MS** (ESI) *m/z* calcd for $C_{41}H_{40}N_3O_7$ $[M+H]^+$: 686.2861, found: 686.2867.

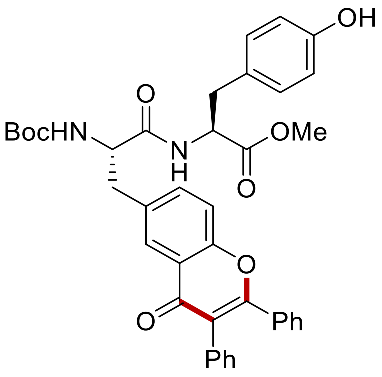

68

Methyl {(*S*)-2-[(*tert*-butoxycarbonyl)amino]-3-(4-oxo-2,3-diphenyl-4*H*-chromen-6-yl)propanoyl}-*L*-tyrosinate

The general procedure B was followed using **58h** (60.4 mg, 0.12 mmol), **4** (11.0 mg, 60 µmol) and $[Cp^*RhCl_2]_2$ (1.9 mg, 5.0 mol %) at 100 °C for 3.5 h. Purification by column chromatography on silica gel (*n*hexane/EtOAc = 3/2 to 1:1) yielded **68** (21.5 mg, 54%) as a white solid.

^{1}H-NMR (400 MHz, $CDCl_3$): δ = 7.96 (s, 1H), 7.49 (d, *J* = 8.6 Hz, 1H), 7.38 (d, *J* = 8.6 Hz, 1H), 7.31–7.28 (m, 2H), 7.27–7.16 (m, 7H), 7.15–7.12 (m, 2H), 6.86 (d, *J* = 8.0 Hz, 1H), 6.79 (d, *J* = 8.5 Hz, 2H), 6.61 (d, *J* = 8.5 Hz, 1H), 5.26 (d, *J* = 5.4 Hz, 1H), 4.72 (ddd, *J* = 7.9, 6.5, 5.4 Hz, 1H), 4.43–4.33 (m, 1H), 3.58 (s, 3H), 3.13 (dd, *J* = 14.0, 5.6 Hz, 1H), 3.00 (dd, *J* = 14.0, 5.3 Hz, 1H), 2.87 (d, *J* = 6.5 Hz, 1H), 2.83 (d, *J* = 6.5 Hz, 1H), 1.25 (s, 9H). **^{13}C-NMR** (101 MHz, $CDCl_3$): δ = 177.8 (C_q), 172.0 (C_q), 171.2 (C_q), 162.0 (C_q), 155.7 (C_q), 155.3 (C_q), 135.0 (CH), 134.4 (C_q), 133.2 (C_q), 133.0 (C_q), 131.3 (CH), 130.5 (CH), 130.3 (CH), 129.7 (CH), 128.5 (CH), 128.2 (CH), 127.9 (CH), 127.0 (C_q), 126.9 (CH), 123.2 (C_q), 123.1 (C_q), 118.5 (CH), 115.8 (CH), 80.4 (C_q), 55.8 (CH), 53.6 (CH), 52.6 (CH_3), 38.1 (CH_2), 37.1 (CH_2), 28.3 (CH_3). **IR** (ATR): $\tilde{\nu}$ = 3312, 2925, 1654, 1616, 1512, 1445, 1371, 1225, 1167, 755 cm^{-1}. **M.p.**: 141–143 °C. **MS** (ESI) *m/z* (relative intensity): 663 (100) $[M+H]^+$, 685 (80) $[M+Na]^+$, 1347 (30) $[2M+Na]^+$. **HR-MS** (ESI) *m/z* calcd for $C_{39}H_{39}N_2O_8$ $[M+H]^+$: 663.2701, found: 663.2708.

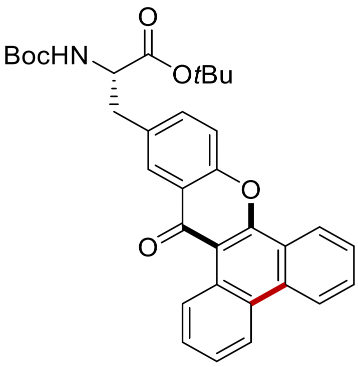

69

***tert*-Butyl (*S*)-2-[(*tert*-butoxycarbonyl)amino]-3-(14-oxo-14*H*-dibenzo[*a*,*c*]xanthen-12-yl)propanoate**

Amino acid **60** (65.8 mg, 0.12 mmol) and KI (10.1 mg, 60 μmol) were added to a quarz tube. Afterwards the tube was closed with a stopper equipped with the electrodes, the solvents were added (EtOH/H_2O, 19/1, 4 mL) and the mixture was degassed by purging the solution with nitrogen for 10 min. The mixture was then electrolyzed (2 mA) in a UV-reactor at 254 nm for 24 h. The electrodes were washed with EtOAc (3x5 mL), the combined solvents were removed in vacuo and the residue was purified by column chromatography on silica gel (*n*hexane/EtOAc = 6/1 to 4/1) to furnish **69** (48.3 mg, 75%) as a white solid.

^{1}H-NMR (400 MHz, $CDCl_3$): δ = 10.09 (dd, *J* = 8.4, 1.4 Hz, 1H), 8.59 (d, *J* = 8.4 Hz, 1H), 8.56 (d, *J* = 8.4 Hz, 2H), 8.21–8.15 (m, 1H), 7.78–7.68 (m, 2H), 7.67–7.60 (m, 2H), 7.59–7.51 (m, 2H), 5.15 (d, *J* = 7.9 Hz, 1H), 4.53 (ddd, *J* = 7.9, 6.1, 5.8 Hz, 1H), 3.27 (dd, *J* = 14.0, 5.8 Hz, 1H), 3.17 (dd, *J* = 14.0, 6.1 Hz, 1H), 1.47 (s, 9H), 1.44 (s, 9H). **^{13}C-NMR** (101 MHz, $CDCl_3$): δ = 178.0 (C_q), 170.8 (C_q), 155.3 (C_q), 155.0 (C_q), 153.0 (C_q), 135.2 (CH), 133.8 (C_q), 133.4 (C_q), 130.6 (CH), 129.0 (C_q), 128.6 (CH), 127.8 (CH), 127.5 (C_q), 127.3 (CH), 127.2 (CH), 126.7 (CH), 124.0 (CH), 123.9 (C_q), 123.7 (C_q), 122.8 (CH), 122.3 (CH), 117.6 (CH), 112.6 (C_q), 82.7 (C_q), 80.0 (C_q), 55.1 (CH), 39.6 (CH_2), 28.5 (CH_3), 28.2 (CH_3). **IR** (ATR): $\tilde{\nu}$ = 1706, 1641, 1488, 1446, 1364, 1244, 1151, 755, 728 cm^{-1}. **M.p.**: 197–199 °C. **MS** (ESI) *m/z* (relative intensity): 540 (60) $[M+H]^+$, 562 (70) $[M+Na]^+$, 1101 (100) $[2M+Na]^+$. **HR-MS** (ESI) *m/z* calcd for $C_{33}H_{34}NO_6$ $[M+H]^+$: 540.2381, found: 540.2380.

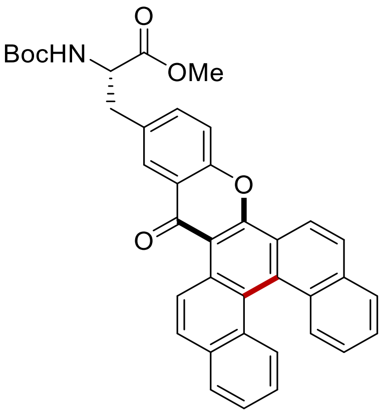

70

Methyl (*S*)-2-[(*tert*-butoxycarbonyl)amino]-3-(16-oxo-16*H*-dinaphtho[2,1-*a*:1',2'-*c*]xanthen-14-yl)propanoate

Amino acid **62** (49.5 mg, 0.08 mmol) and KI (6.9 mg, 40 μmol) were added to a quarz tube. Afterwards the tube was closed with a stopper equipped with the electrodes, the solvents were added (MeOH/H_2O, 19/1, 4 mL) and the mixture was degassed by purging the solution with nitrogen for 10 min. The mixture was then electrolyzed (2 mA) in a UV-reactor at 254 nm for 24 h. The electrodes were washed with EtOAc (3x5 mL), the combined solvents were removed in vacuo and the residue was purified by column chromatography on silica gel (*n*hexane/EtOAc = 5/1 to 3/1) to furnish **70** (39.9 mg, 81%) as a yellow solid.

^{1}H-NMR (400 MHz, $CDCl_3$): δ = 10.10 (dd, *J* = 9.2, 2.8 Hz, 1H), 8.58 (dd, *J* = 8.8, 5.1 Hz, 1H), 8.24 (dd, *J* = 8.5, 2.8 Hz, 1H), 8.21–8.18 (m, 1H), 8.15 (d, *J* = 9.2 Hz, 1H), 8.11 (dd, *J* = 9.2, 2.4 Hz, 1H), 8.02–7.93 (m, 3H), 7.63–7.55 (m, 3H), 7.52 (dd, *J* = 7.5, 7.5 Hz, 1H), 7.31–7.26 (m, 1H), 7.26–7.19 (m, 1H), 5.18 (d, *J* = 8.2 Hz, 1H), 4.71 (ddd, *J* = 8.2, 7.2, 7.2 Hz, 1H), 3.82 (s, 3H), 3.34 (d, *J* = 15.2 Hz, 1H), 3.22 (d, *J* = 15.2 Hz, 1H), 1.45 (s, 9H). **^{13}C-NMR** (101 MHz, $CDCl_3$): δ = 178.2 (C_q), 172.2 (C_q), 155.3 (C_q), 154.5 (C_q), 153.7 (C_q), 135.1 (CH), 133.9 (C_q), 133.0 (C_q), 132.0 (C_q), 131.6 (C_q), 130.3 (C_q), 130.3 (C_q), 130.0 (CH), 129.4 (C_q), 129.3 (CH), 129.3 (CH), 128.4 (CH), 128.0 (CH), 127.9 (CH), 127.6 (CH), 127.1 (CH), 126.3 (CH), 125.3 (CH), 124.6 (CH), 123.8 (C_q), 123.7 (CH), 123.6 (C_q), 123.1 (C_q), 119.3 (CH), 117.9 (CH), 113.3 (C_q), 80.3 (C_q), 54.7 (CH), 52.6 (CH_3), 38.2 (CH_2), 28.4 (CH_3). **IR** (ATR): $\tilde{\nu}$ = 2925, 1743, 1709, 1641, 1485, 1441, 1247, 1165, 823, 753 cm^{-1}. **M.p.**: 142–144 °C. **MS** (ESI) *m/z* (relative intensity): 598 (80) $[M+H]^+$, 620 (100) $[M+Na]^+$, 1217 (90) $[2M+Na]^+$. **HR-MS** (ESI) *m/z* calcd for $C_{38}H_{32}NO_6$ $[M+H]^+$: 598.2224, found: 598.2228.

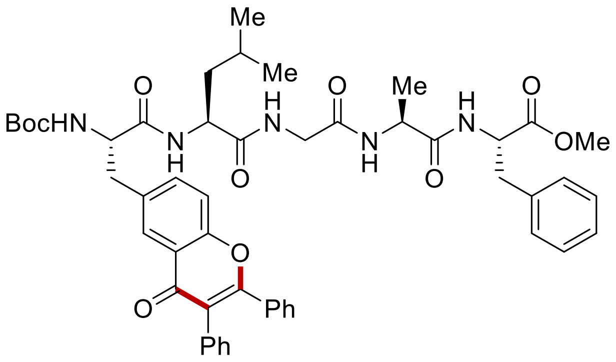

72

Methyl {(*S*)-2-[(*tert*-butoxycarbonyl)amino]-3-(4-oxo-2,3-diphenyl-4*H*-chromen-6-yl)propanoyl}-*L*-leucylglycyl-*L*-alanyl-*L*-phenylalaninate

The general procedure B was followed using **71** (192 mg, 0.27 mmol) and **4** (23.1 mg, 0.13 mmol) at 100 °C for 3.5 h. Purification by column chromatography on silica gel (CH_2Cl_2/MeOH = 95/5) yielded **72** (92.3 mg, 80%) as a white solid.

^{1}H-NMR (400 MHz, DMSO-d$_6$): δ = 8.32 (d, *J* = 7.6 Hz, 1H), 8.20–8.12 (m, 2H), 8.08 (d, *J* = 2.1 Hz, 1H), 7.89 (d, *J* = 7.6 Hz, 1H), 7.84–7.74 (m, 1H), 7.63 (d, *J* = 8.5 Hz, 1H), 7.40–7.35 (m, 3H), 7.34–7.26 (m, 5H), 7.24 (d, *J* = 6.8 Hz, 2H), 7.21–7.19 (m, 2H), 7.19–7.17 (m, 1H), 7.15 (dd, *J* = 7.6, 1.9 Hz, 2H), 7.06 (d, *J* = 8.5 Hz, 1H), 4.49–4.39 (m, 1H), 4.38–4.29 (m, 2H), 4.29–4.21 (m, 1H), 3.70 (d, *J* = 5.9 Hz, 1H), 3.56 (s, 3H), 3.15 (dd, *J* = 13.7, 3.7 Hz, 1H), 3.01 (dd, *J* = 13.8, 5.9 Hz, 1H), 2.93 (dd, *J* = 12.1, 6.9 Hz, 1H), 2.88 (dd, *J* = 12.1, 9.0 Hz, 1H), 1.73–1.59 (m, 1H), 1.57–1.45 (m, 2H), 1.28 (s, 9H), 1.17 (d, *J* = 7.1 Hz, 3H), 0.89 (d, *J* = 6.4 Hz, 3H), 0.84 (d, *J* = 6.4 Hz, 3H). **^{13}C-NMR** (101 MHz, DMSO-d$_6$): δ =176.3 (C$_q$), 172.4 (C$_q$), 172.3 (C$_q$), 171.8 (C$_q$), 171.6 (C$_q$), 168.2 (C$_q$), 161.2 (C$_q$), 155.4 (C$_q$), 154.5 (C$_q$), 137.1 (C$_q$), 136.0 (C$_q$), 135.8 (CH), 133.2 (C$_q$), 133.0 (C$_q$), 131.2 (CH), 130.2 (CH), 129.4 (CH), 129.1 (CH), 128.3 (CH), 128.2 (CH), 128.1 (CH), 127.5 (CH), 126.6 (CH), 125.7 (CH), 122.5 (C$_q$), 122.4 (C$_q$), 118.0 (CH), 78.2 (C$_q$), 56.0 (CH), 53.7 (CH), 51.9 (CH_3), 51.3 (CH), 47.8 (CH), 42.0 (CH_2), 41.0 (CH_2), 36.7 (CH_2), 36.6 (CH_2), 28.1 (CH_3), 24.1 (CH), 23.1 (CH_3), 21.6 (CH_3), 18.3 (CH_3). **IR** (ATR): $\tilde{\nu}$ = 3291, 1642, 1521, 1370, 1221, 1167, 1050, 1026, 747, 697 cm^{-1}. **M.p.**: 66–68 °C. **MS** (ESI) *m/z* (relative intensity): 888 (100) [M+H]$^+$, 910 (70) [M+Na]$^+$. **HR-MS** (ESI) *m/z* calcd for $C_{50}H_{58}N_5O_{10}$ [M+H]$^+$: 888.4178, found: 888.4165.

14. Studies on Potential Racemization

A racemic tyrosine derivative *rac*-**58a** was employed in the rhodaelectro-catalyzed C−H activation and analyzed by HPLC, showing that no racemization occurs during the catalysis. The *ee* % of **60** was determined >97%.

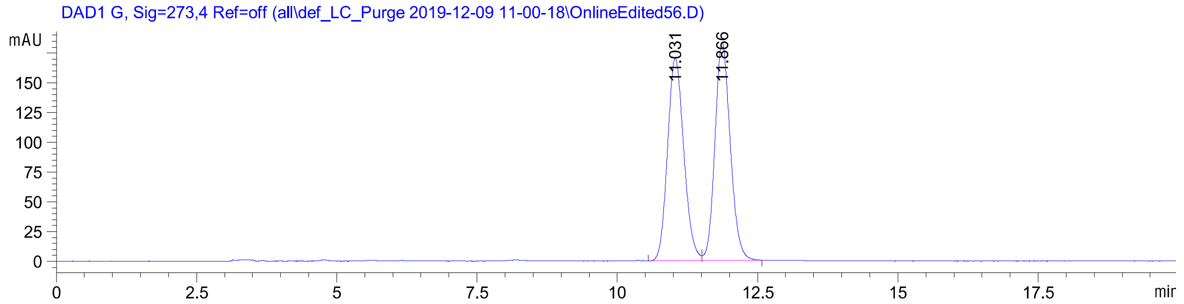

Signal 2: DAD1 G, Sig=273,4 Ref=off

Peak #	RetTime [min]	Type	Width [min]	Area [mAU*s]	Height [mAU]	Area %
1	11.031	VV R	0.3046	3434.80225	170.10265	49.6033
2	11.866	VB	0.2919	3489.74536	183.43951	50.3967
Totals :				6924.54761	353.54216	

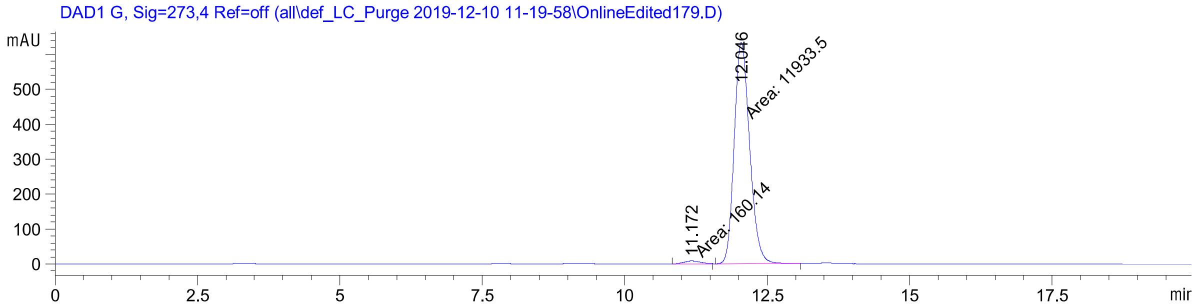

Signal 2: DAD1 G, Sig=273,4 Ref=off

Peak #	RetTime [min]	Type	Width [min]	Area [mAU*s]	Height [mAU]	Area %
1	11.172	MM	0.3282	160.13980	8.13293	1.3242
2	12.046	MM	0.3138	1.19335e4	633.90845	98.6758
Totals :				1.20937e4	642.04137	

Supplementary Figure 19. HPLC chromatograms of *rac*-**60** and **60**. HPLC chromatograms were recorded on an Agilent 1290 Infinity using the column CHIRALPAK® IA and *n*hexane/*i*PrOH (70:30, 1 mL/min, detection at 250 nm).

15. Computational Studies

All calculations were performed using the Gaussian 16, Revision A.03 package.[26] All structures were optimized at the PBE0[27,28] level of theory in combination with D3 dispersion corrections with the Becke-Johnson damping scheme (D3BJ).[29,30] Analytical frequency calculations were carried out at the same level of theory in order to identify each stationary point as either an intermediate (no imaginary frequencies) or a transition state (only one imaginary frequency) and to provided thermal and non-thermal corrections to the Gibbs free energy at 373.15 K and 1 atm. All atoms were described with the def2-SVP basis set,[31-34] while rhodium was in addtion described with a SSD pseudopotential.[35,36] The electronic energy was then refined through PW6B95[37] single-point calculations on the optimized geometries with a def2-QZVP basis set[31-34] combined with a SSD pseudopotential for rhodium.[35,36] Solvent effects were included implicitly through the use of the SMD model[38] for methanol and acetonitrile. Energies reported herein are based on gas-phase Gibbs free energies with a def2-SVP basis set, for which the electronic energies were corrected to PW6B95-D3(BJ) with a def2-QZVP basis set and solvent effects. Non-covalent interactions where localized and visualized through the use of NCIPLOT program.[39,40]

Oxidation potentials were calculated as follows:[41]

$$E_{1/2}^{\mathrm{o,calc}} = -\frac{\Delta G_{1/2}^{\mathrm{o}}}{n_e F} - E_{1/2}^{\mathrm{o,SHE}} - E^{\mathrm{o,SCE}} \quad (1)$$

With $\Delta G_{1/2}^{\mathrm{o}}$ defined as:

$$\Delta G_{1/2}^{\mathrm{o}} = G_{298.15}(reduced) - G_{298.15}(oxidized) \quad (2)$$

In the above equation, n_e is the number of electrons transferred during the oxidation or reduction process, F is the Faraday constant with a value of 23.061 kcal $mol^{-1}V^{-1}$, $E_{1/2}^{\mathrm{o,SHE}}$ is the

absolute value of the standard hydrogen electrode (4.281 V), and $E^{o,SCE}$ is the saturated calomel electrode relative to SHE in sat. KCl (0.244 V). The calculated oxidation potential was then corrected referenced to oxidation potential of ferrocene ($Fc^{0/+}$) at the respective electrolyte concentration.[42]

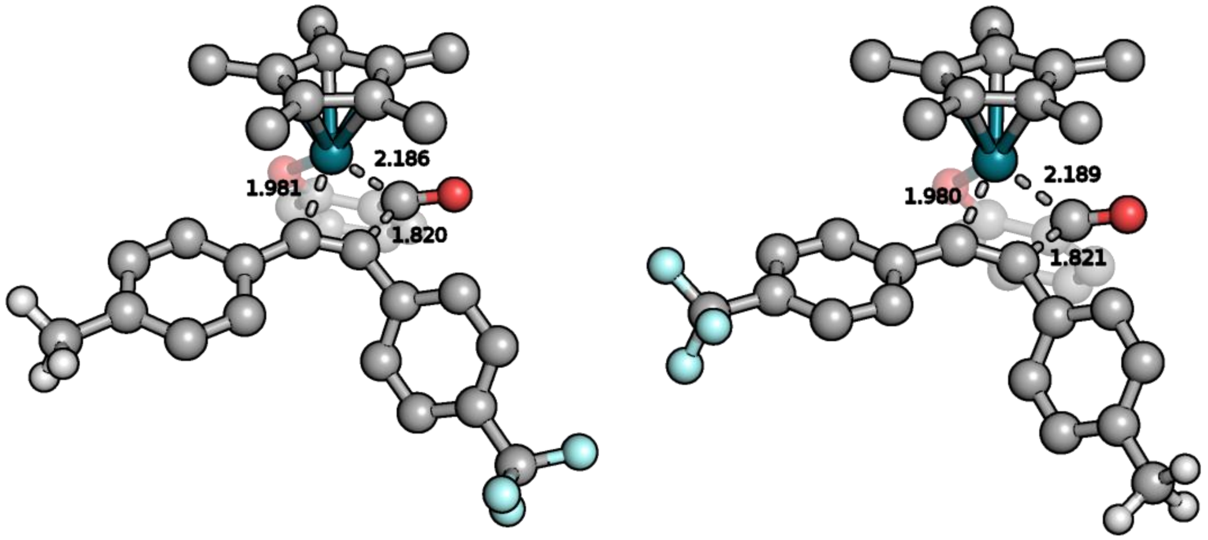

TS(1-2)*^d^* **TS(1-2)*^p^***

Supplementary Figure 20. Computed transition state structures for the migratory insertion elementary step for the regioisomer where the methyl-substituted phenyl moiety is in a distal [**TS(1-2)*^d^***] and in a proximal [**TS(1-2)*^p^***] position with respect to the carbonyl group of the substrate with alkyne **10l**. Key distances are provided in Å and non-participating hydrogen atoms are omitted for clarity.

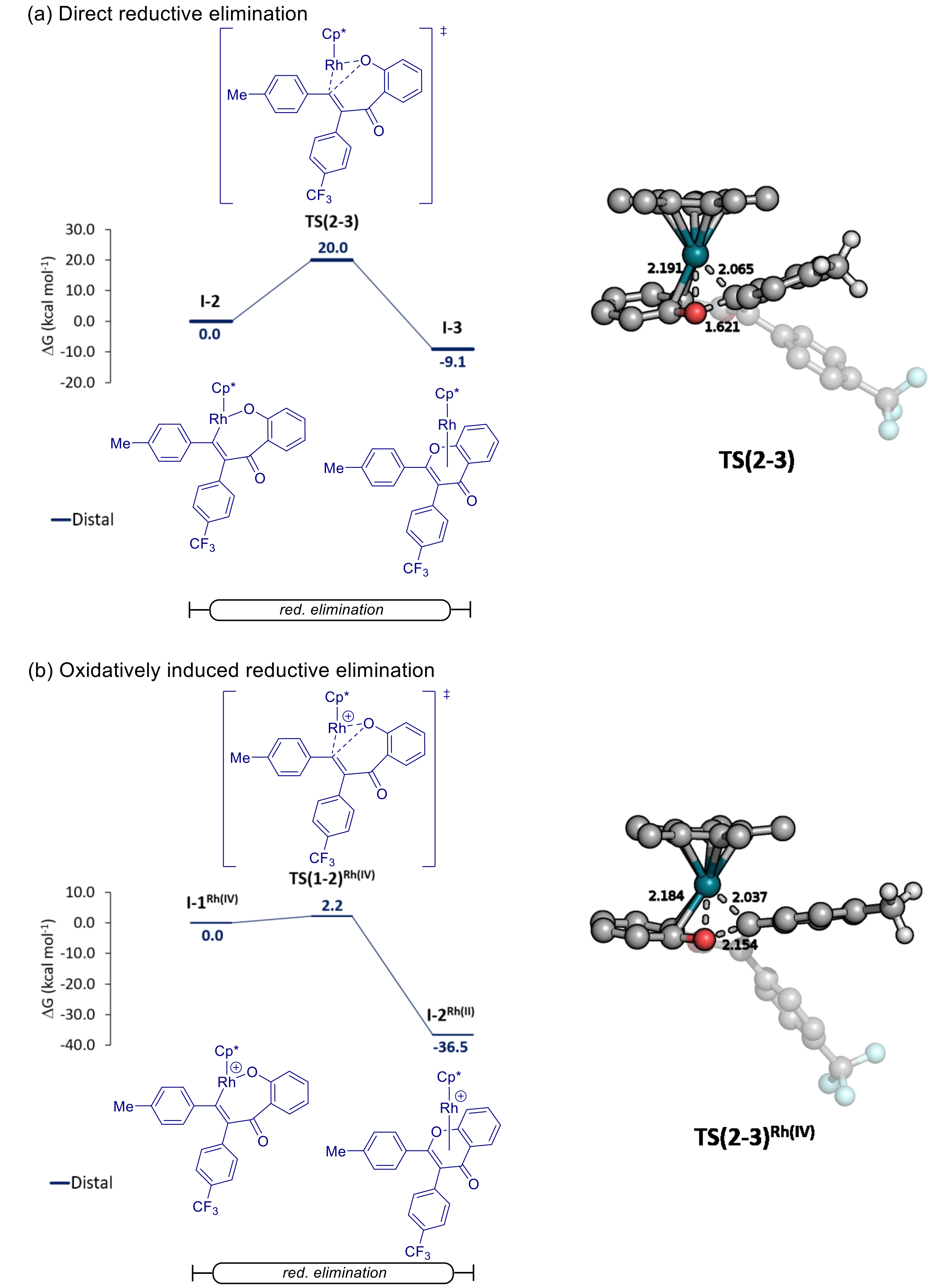

Supplementary Figure 21. Gibbs free energy profile in kcal mol^{-1} for the direct reductive elimination and oxidatively induced reductive elimination steps for alkyne **10l** at the PW6B95-D3(BJ)/def2-QZVP+SMD(methanol) //PBE0-D3(BJ)/def2-SVP level of theory. Nonparticipating hydrogen atoms are omitted for clarity. Relevant bond distances are given in Å.

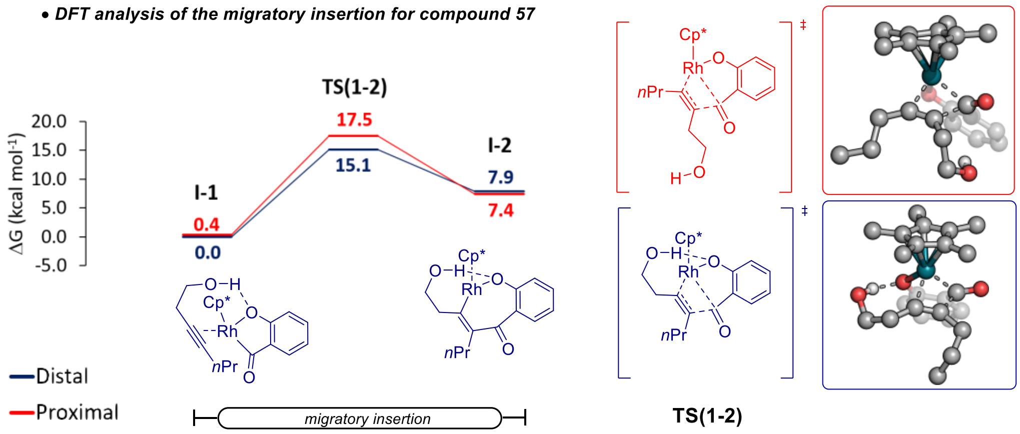

Supplementary Figure 22. Computed Gibbs free energy profiles in kcal mol^{-1} for the regioselectivity of the migratory insertion step of alkyne **10w** calculated at the PW6B95-D3(BJ)/def2-QZVP+SMD(methanol)//PBE0-D3(BJ)/def2-SVP level of theory. In the transition state structures non-relevant hydrogens were omitted for clarity.

Supplementary Table 3. Calculated electronic energies at the PW6B95-D3(BJ)/def2-QZVP+SMD(methanol or acetonitrile) level of theory and Gibbs free Energies with dispersion corrections for all structures for the system with alkyne **10l** (all in Hartree).[a]

Structure	Solvent	Electronic Energy	Total Gibbs Free Energy
**I-1**^{d}	MeOH	-1839.245747	-1838.807016
**TS(1-2)**^{d}	MeOH	-1839.224050	-1838.784260
**I-2**^{d}	MeOH	-1839.258587	-1838.813882
**I-2**^{d}	MeCN[b]	-1839.258004	-1838.783401[b]
**I-1**^{p}	MeOH	-1839.245746	-1838.807420
**TS(1-2)**^{p}	MeOH	-1839.222440	-1838.783769
**I-2**^{p}	MeOH	-1839.258379	-1838.814543
**TS(2-3)**^{d}	MeOH	-1839.227813	-1838.782003
**I-3**^{d}	MeOH	-1839.276886	-1838.828332
**I-2**$^{d,\mathrm{Rh(IV)}}$	MeOH	-1839.053486	-1838.611035
**I-2**$^{d,\mathrm{Rh(IV)}}$	MeCN[b]	-1839.055309	-1838.582554[b]
**TS(2-3)**$^{d,\mathrm{Rh(IV)}}$	MeOH	-1839.051177	-1838.607571
**I-3**$^{d,\mathrm{Rh(II)}}$	MeOH	-1839.114657	-1838.669254

[a] Superscripts *d* and *p* correspond to the regioisomer structures where the methyl-substituted phenyl moiety is in a distal and in a proximal position with respect to the carbonyl group from the substrate, respectively. [b] indicates that the total Gibbs free energy was calculated at 298.15 K.

Supplementary Table 4. Calculated electronic energies at the PW6B95-D3(BJ)/def2-QZVP+SMD(methanol) level of theory and Gibbs free Energies with dispersion corrections for all structures for the system with alkyne **10w** (all in Hartree).[a]

Structure	Solvent	Electronic Energy	Total Gibbs Free Energy
**I-1**^{d}	MeOH	-1271.604438	-1271.194065
**TS(1-2)**^{d}	MeOH	-1271.582437	-1271.169931
**I-2**^{d}	MeOH	-1271.593163	-1271.181422
**I-1**^{p}	MeOH	-1271.601381	-1271.193477
**TS(1-2)**^{p}	MeOH	-1271.575224	-1271.166122
**I-2**^{p}	MeOH	-1271.589724	-1271.182267

[a] Superscripts *d* and *p* correspond to the regioisomer structures where the alcohol moiety is in a distal and in a proximal position with respect to the carbonyl group from the substrate.

16. NMR Spectra

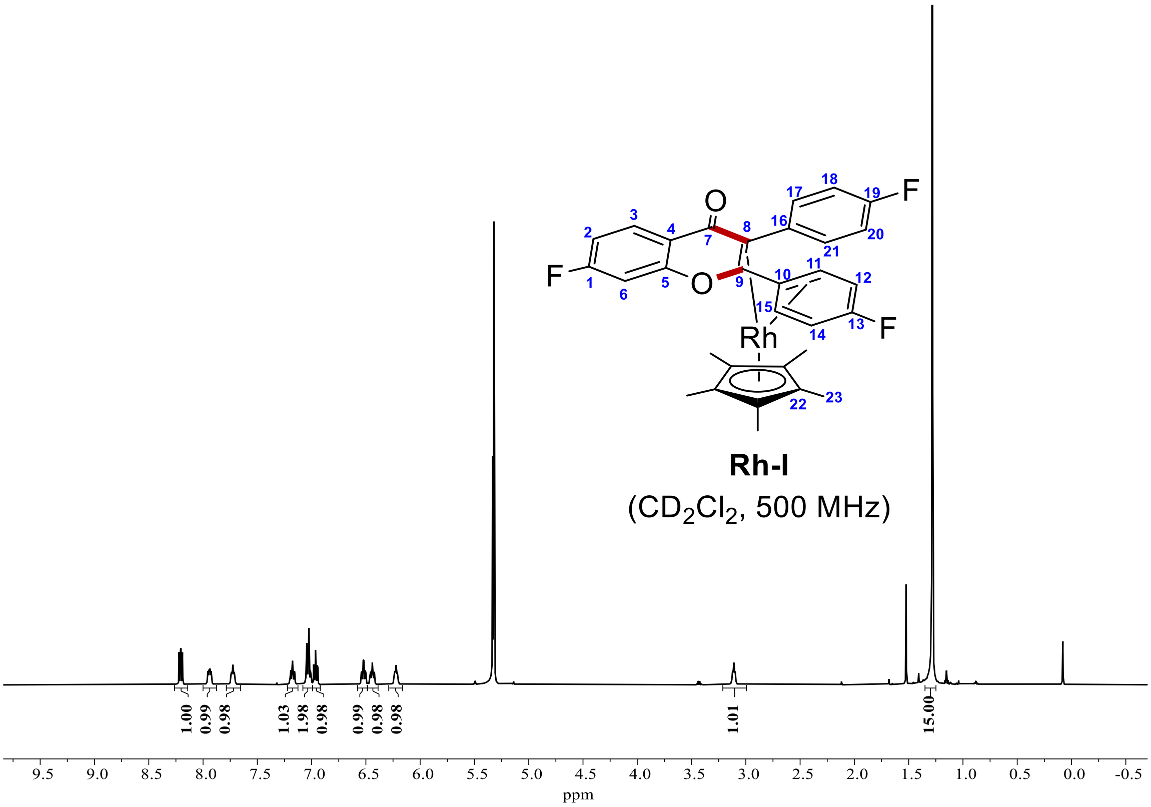

Supplementary Figure 23. ^{1}H-NMR spectrum of **Rh-I**.

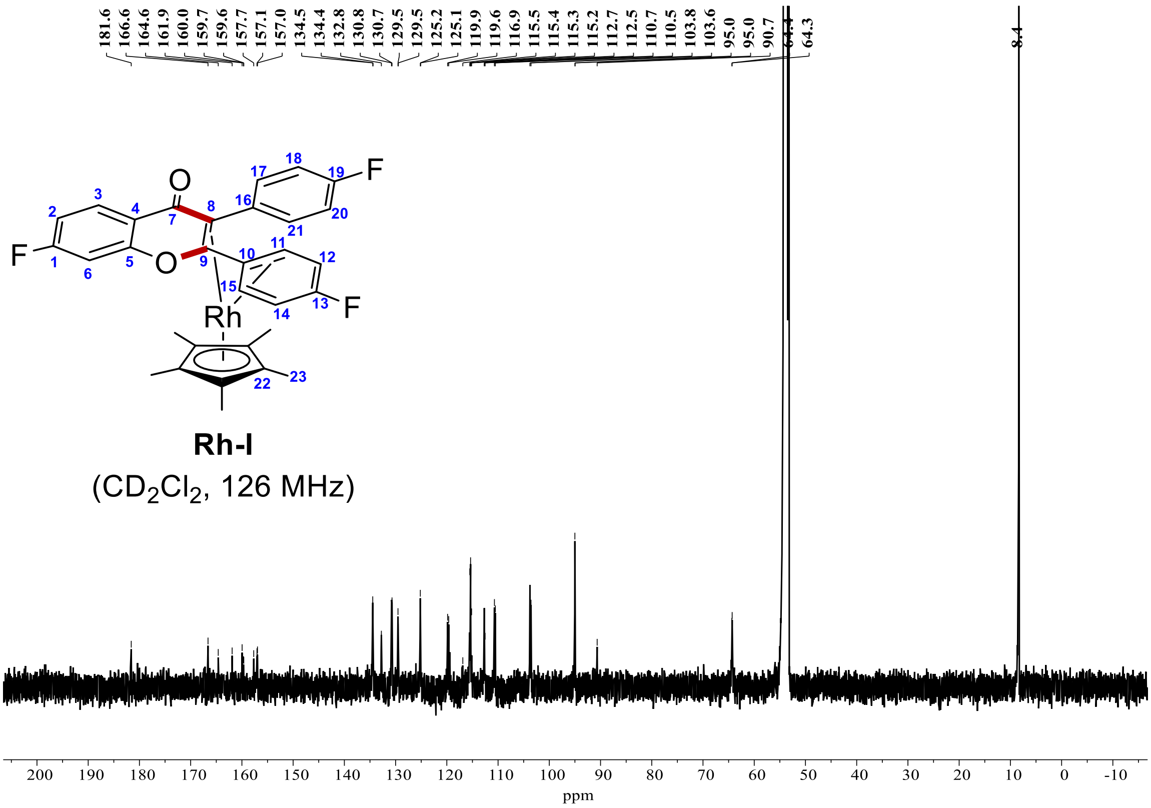

Supplementary Figure 24. ^{13}C-NMR spectrum of **Rh-I**.

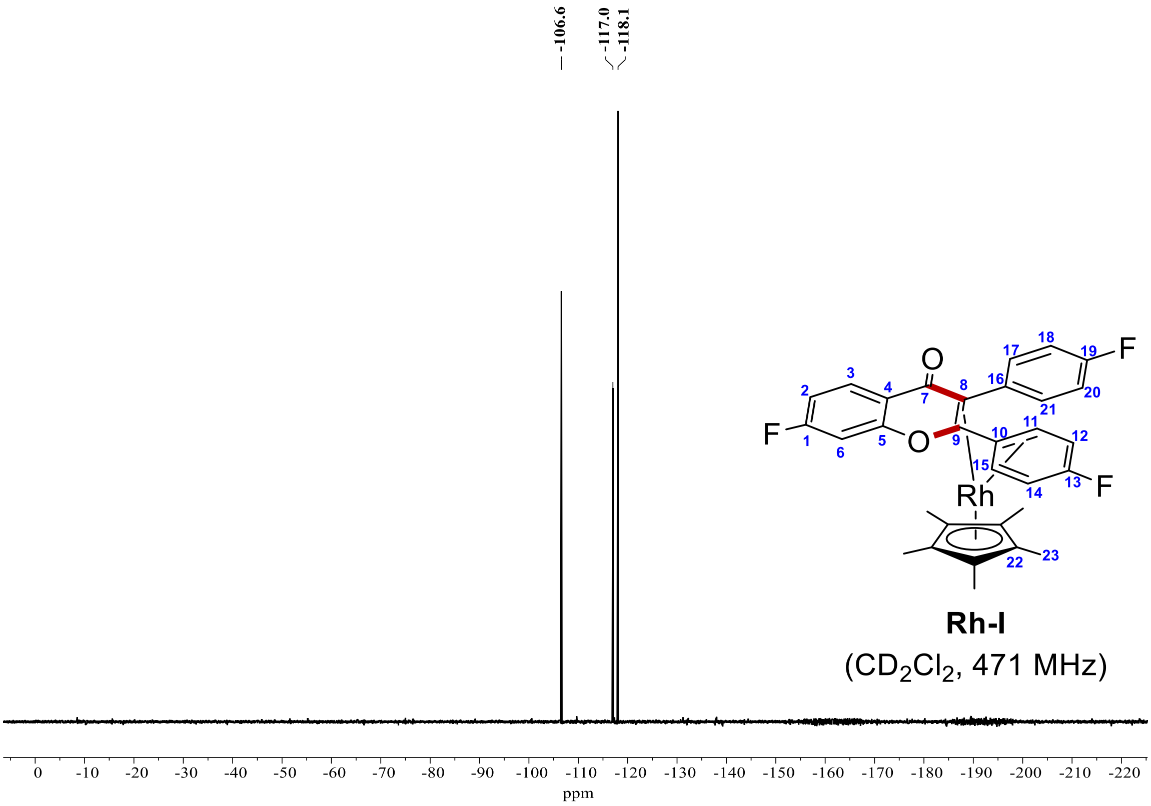

Supplementary Figure 25. ^{19}F-NMR spectrum of **Rh-I**.

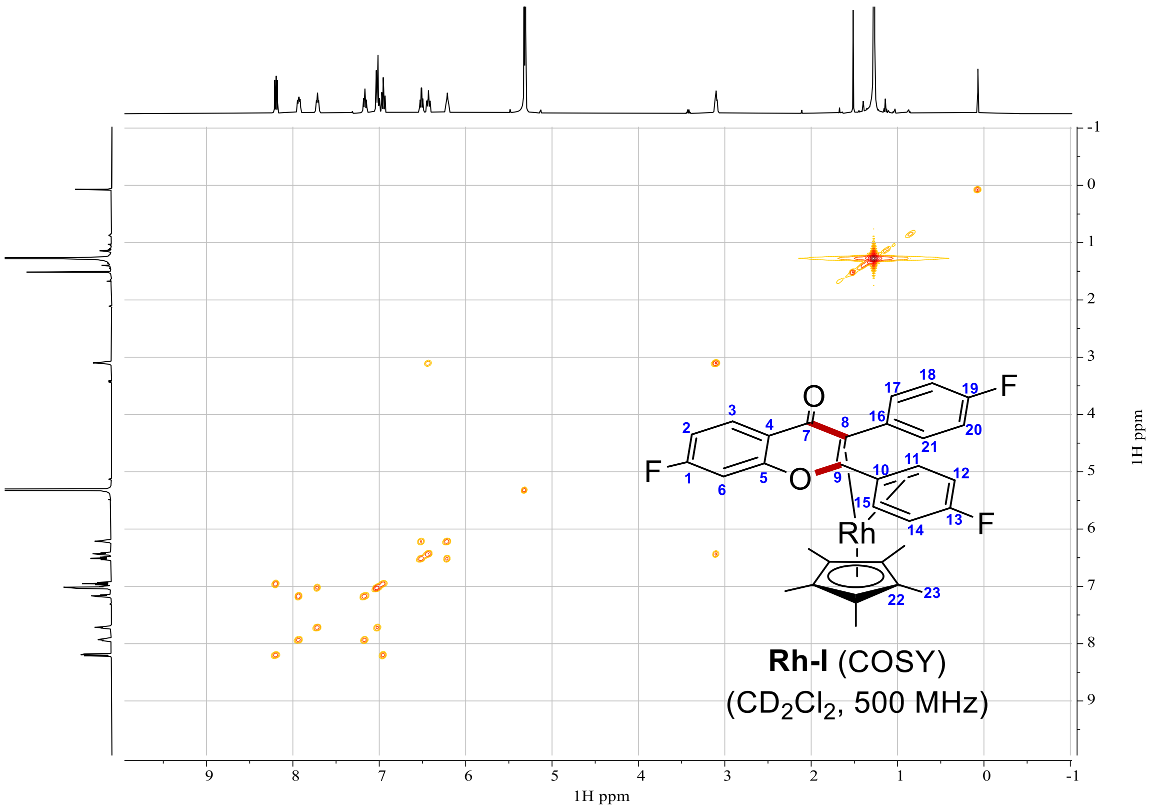

Supplementary Figure 26. 2D-NMR (COSY) spectrum of **Rh-I**.

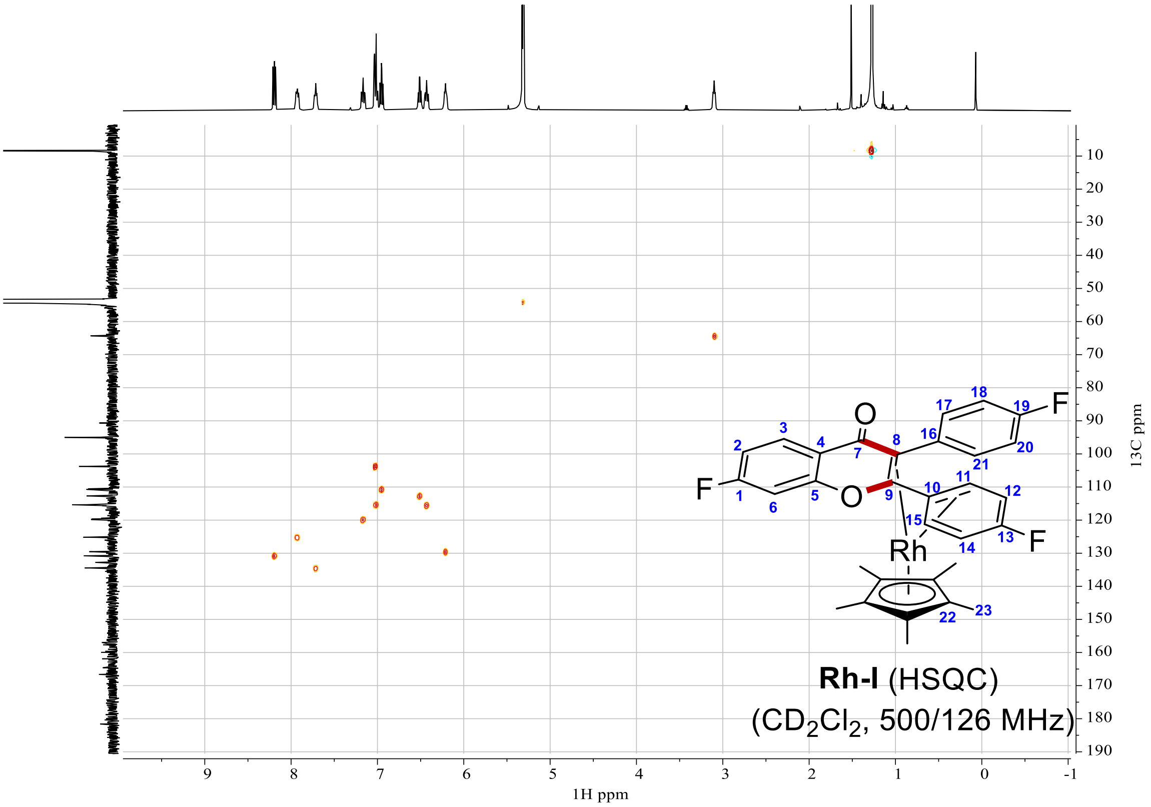

Supplementary Figure 27. 2D-NMR (HSQC) spectrum of **Rh-I**.

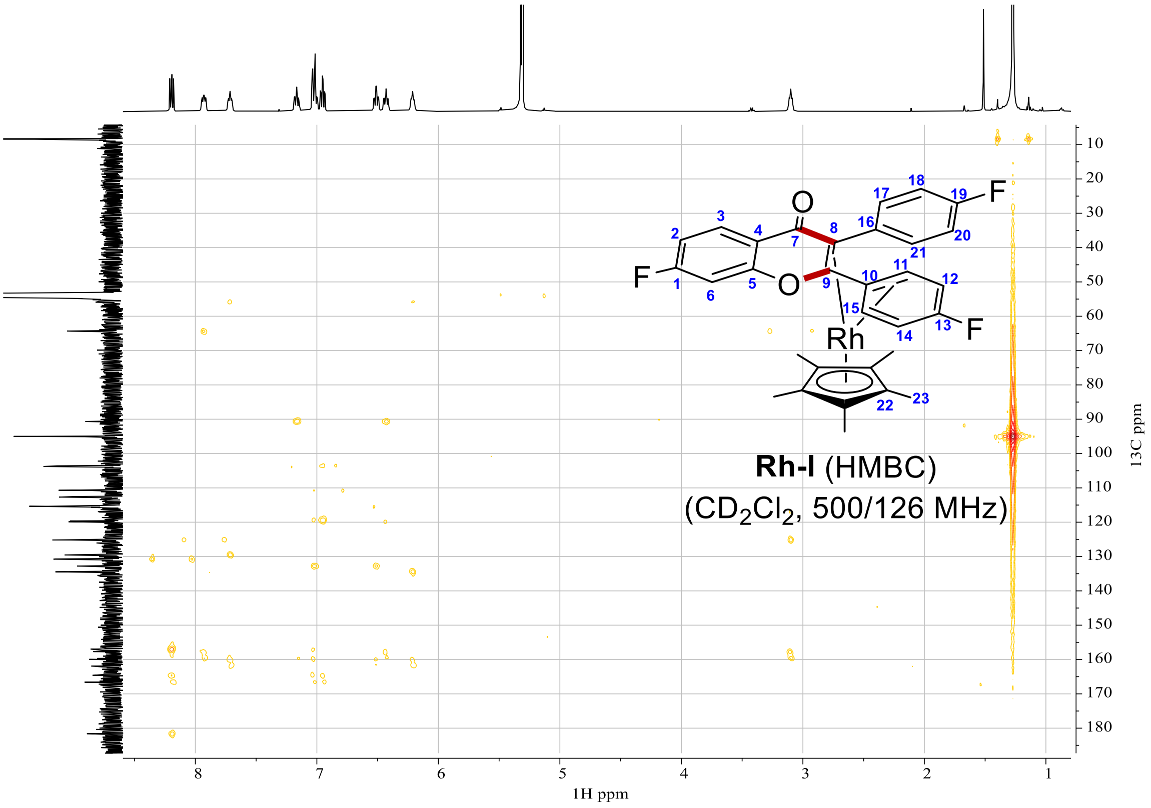

Supplementary Figure 28. 2D-NMR (HMBC) spectrum of **Rh-I**.

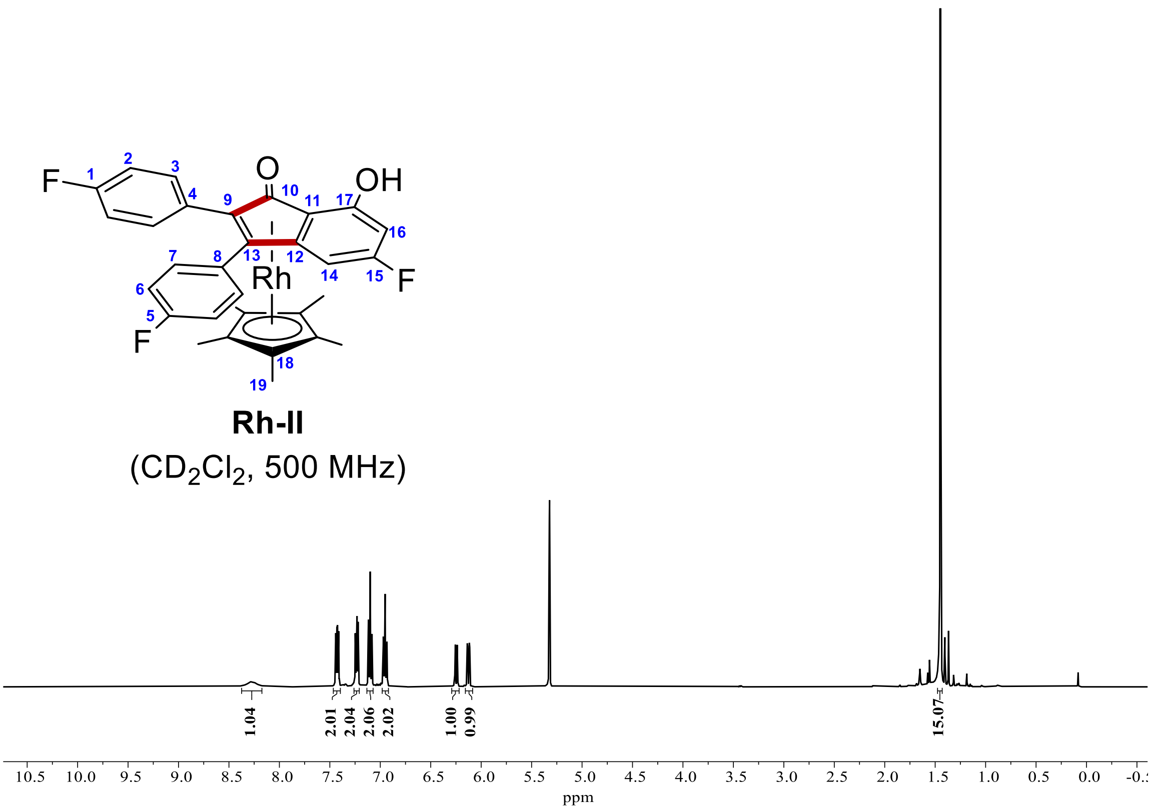

Supplementary Figure 29. ^{1}H-NMR spectrum of **Rh-II.**

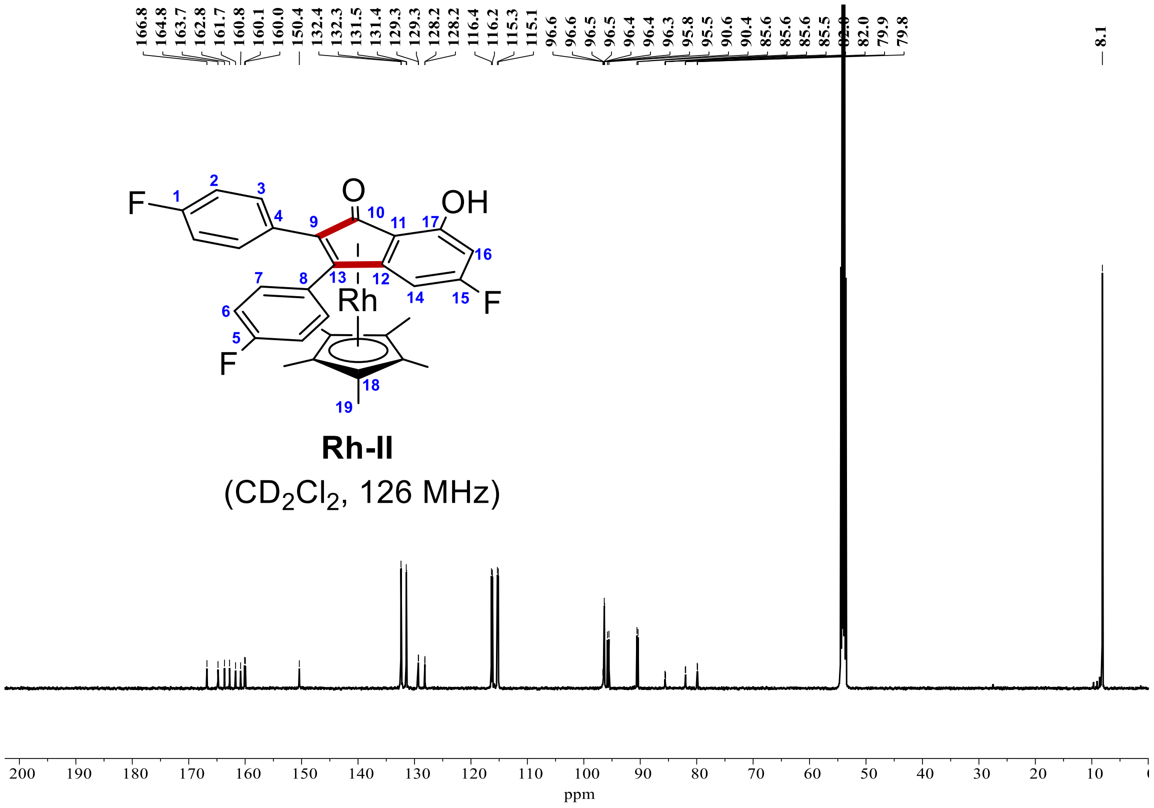

Supplementary Figure 30. ^{13}C-NMR spectrum of **Rh-II.**

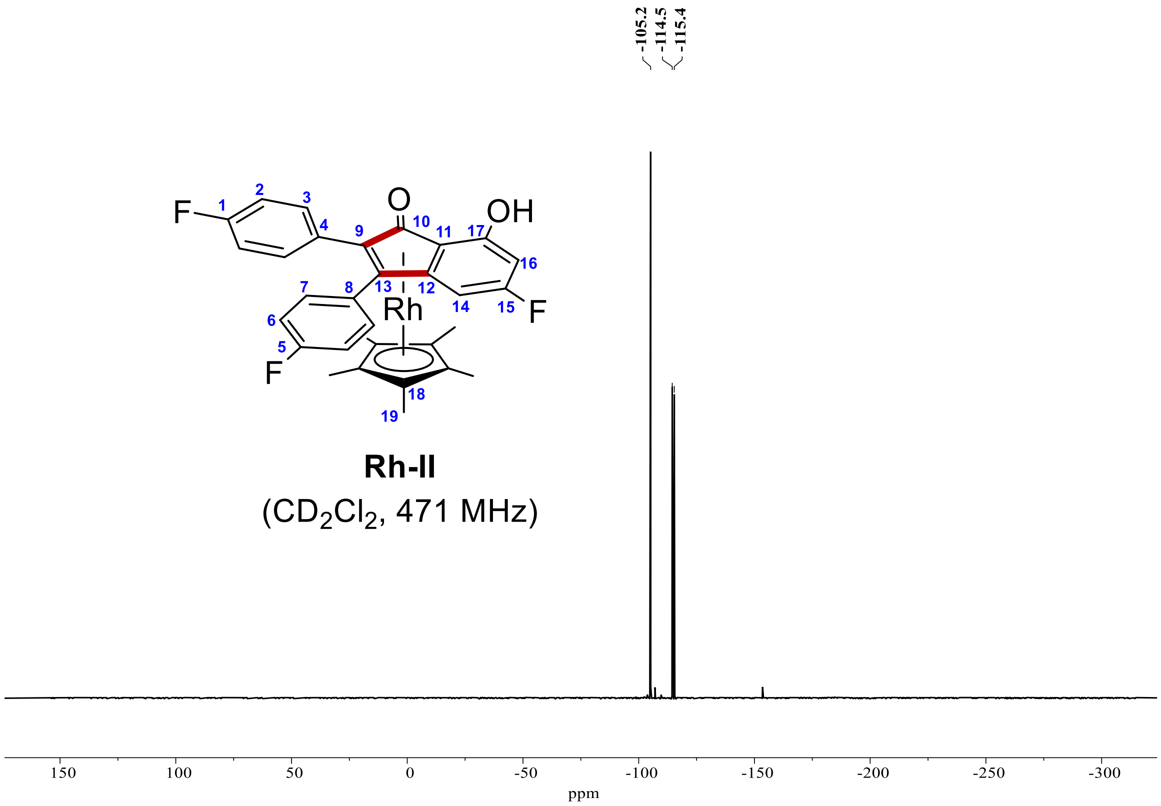

Supplementary Figure 31. ^{19}F-NMR spectrum of **Rh-II.**

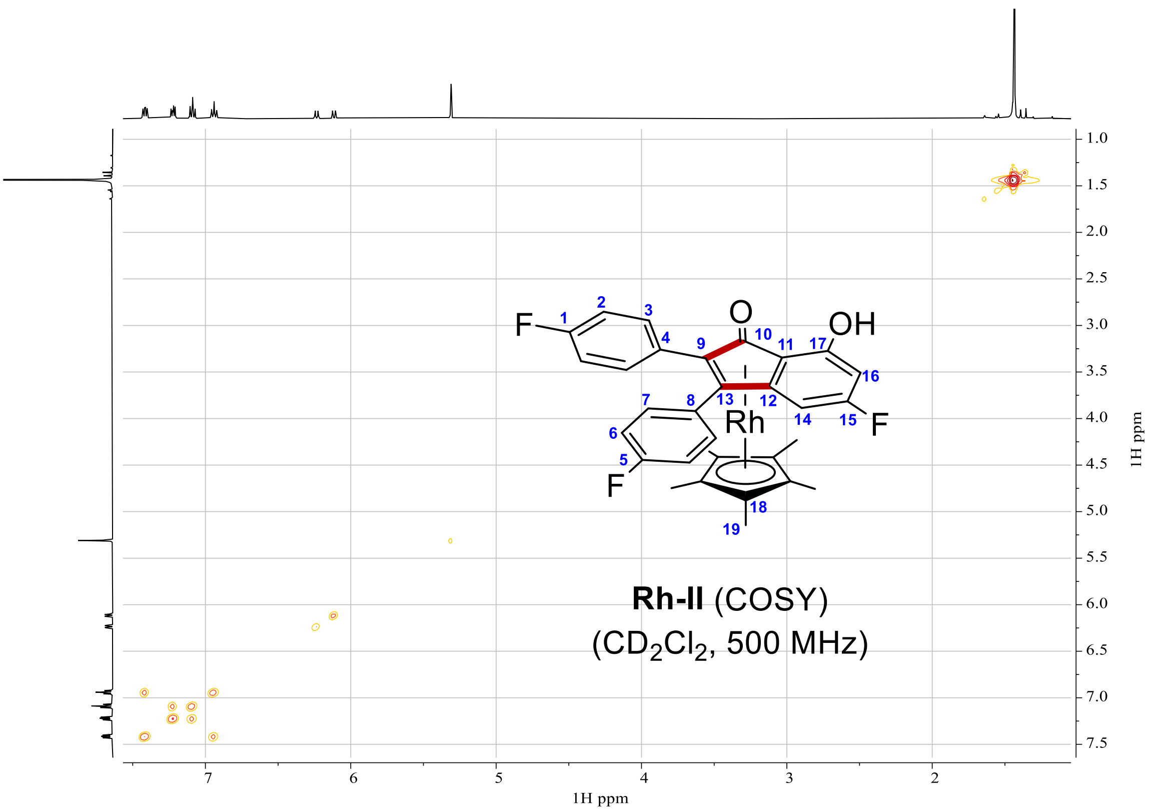

Supplementary Figure 32. 2D-NMR (COSY) spectrum of **Rh-II**.

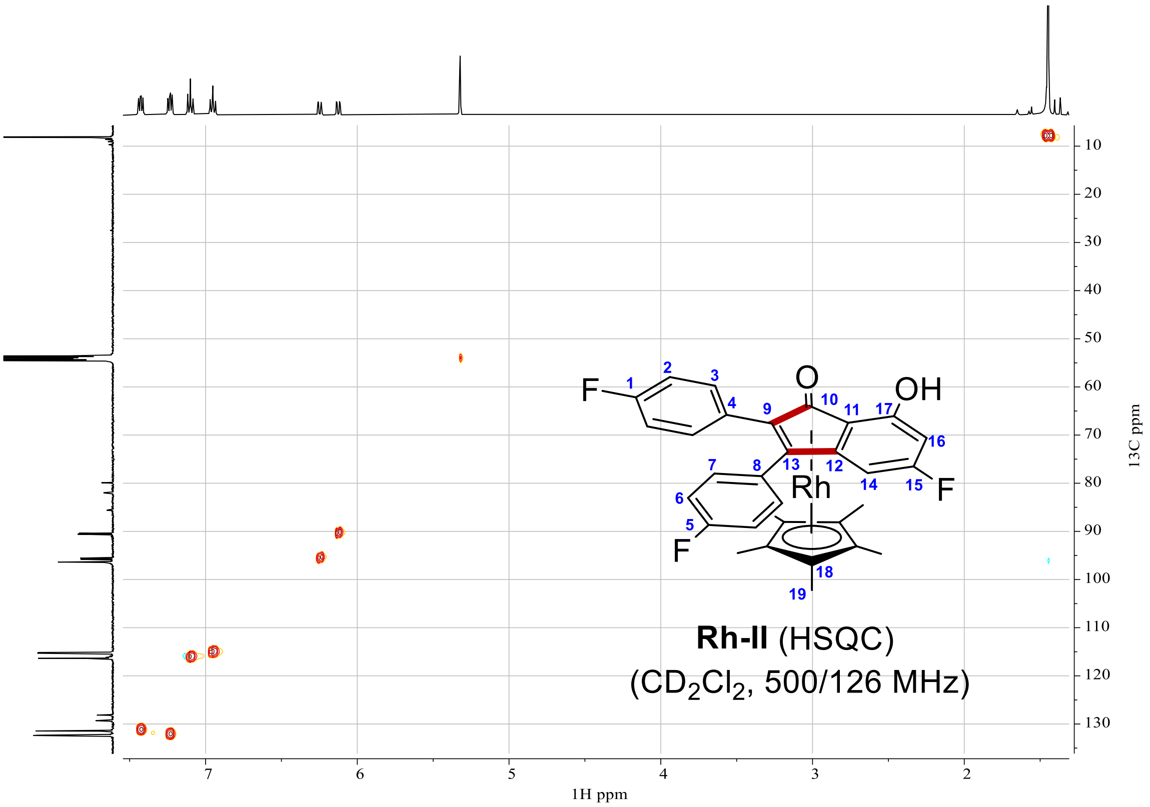

Supplementary Figure 33. 2D-NMR (HSQC) spectrum of **Rh-II**.

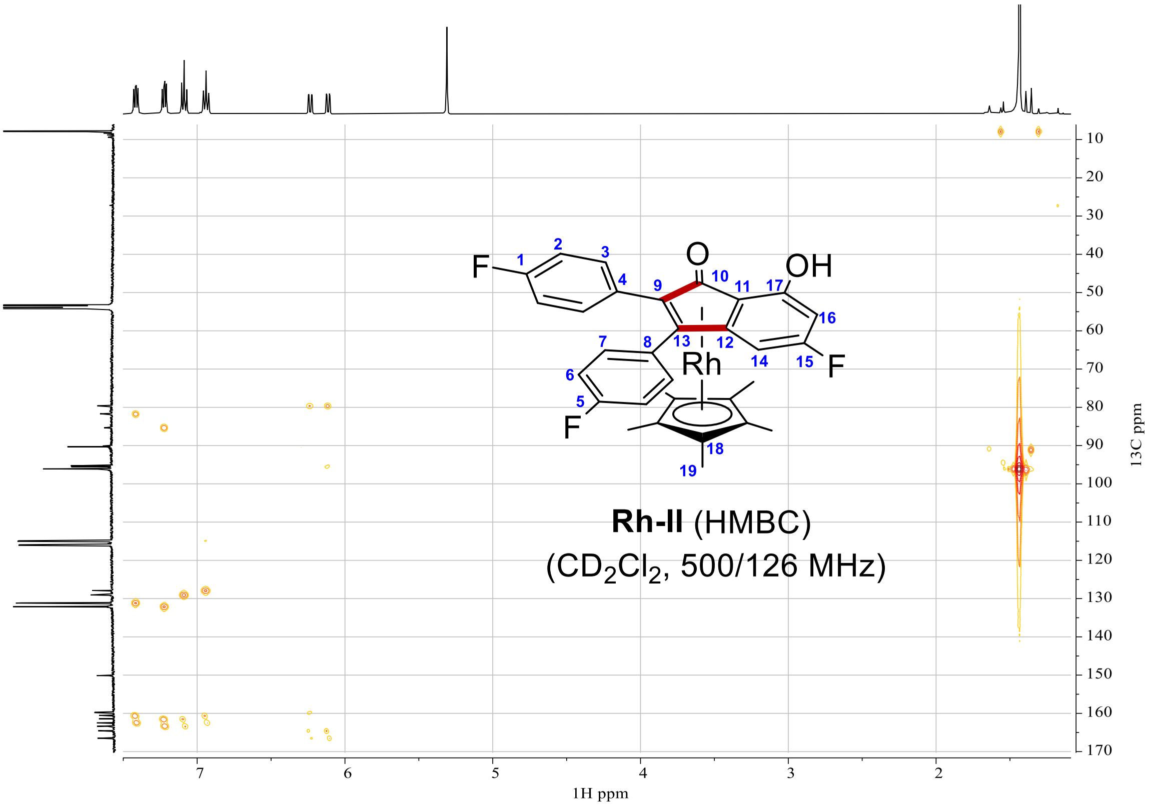

Supplementary Figure 34. 2D-NMR (HMBC) spectrum of **Rh-II**.

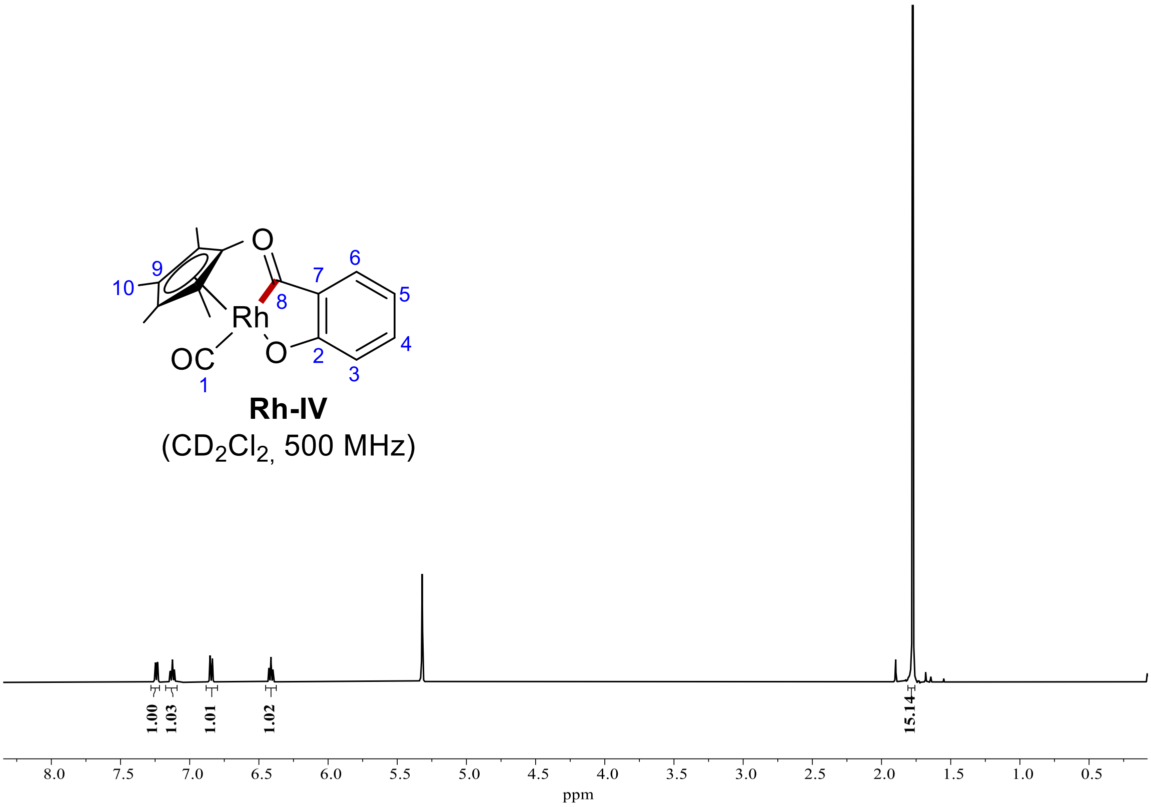

Supplementary Figure 35. ^{1}H-NMR spectrum of **Rh-IV**.

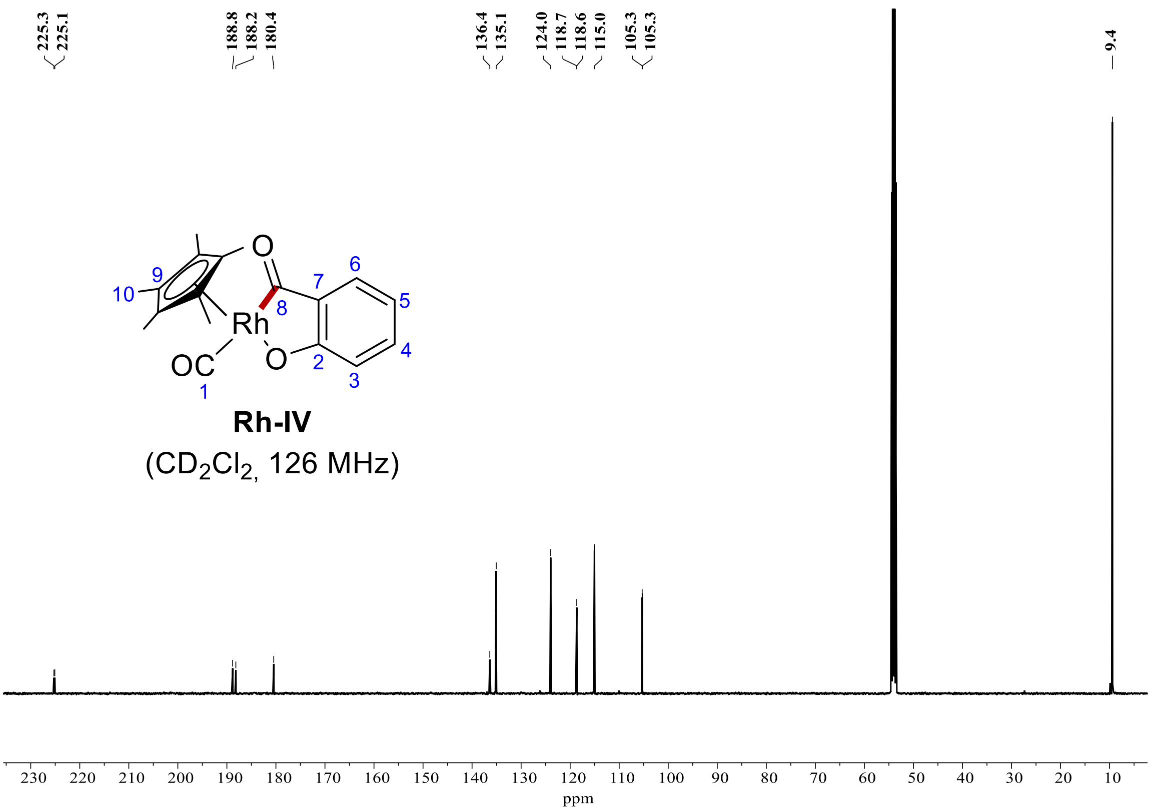

Supplementary Figure 36. ^{13}C-NMR spectrum of **Rh-IV**.

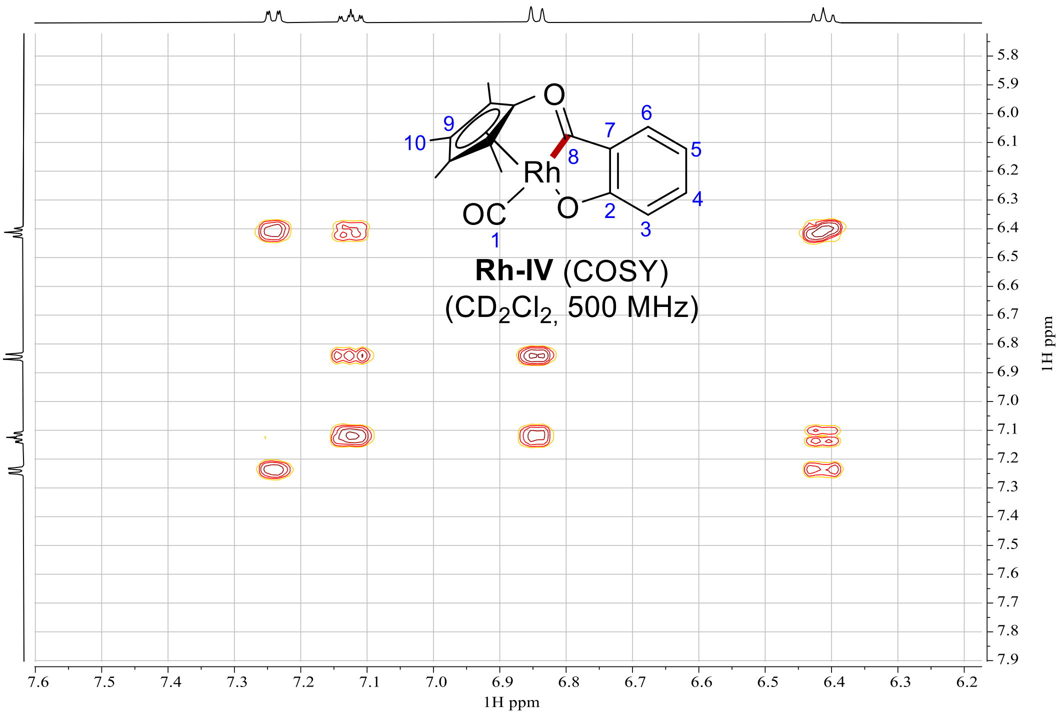

Supplementary Figure 37. 2D-NMR (COSY) spectrum of **Rh-IV**.

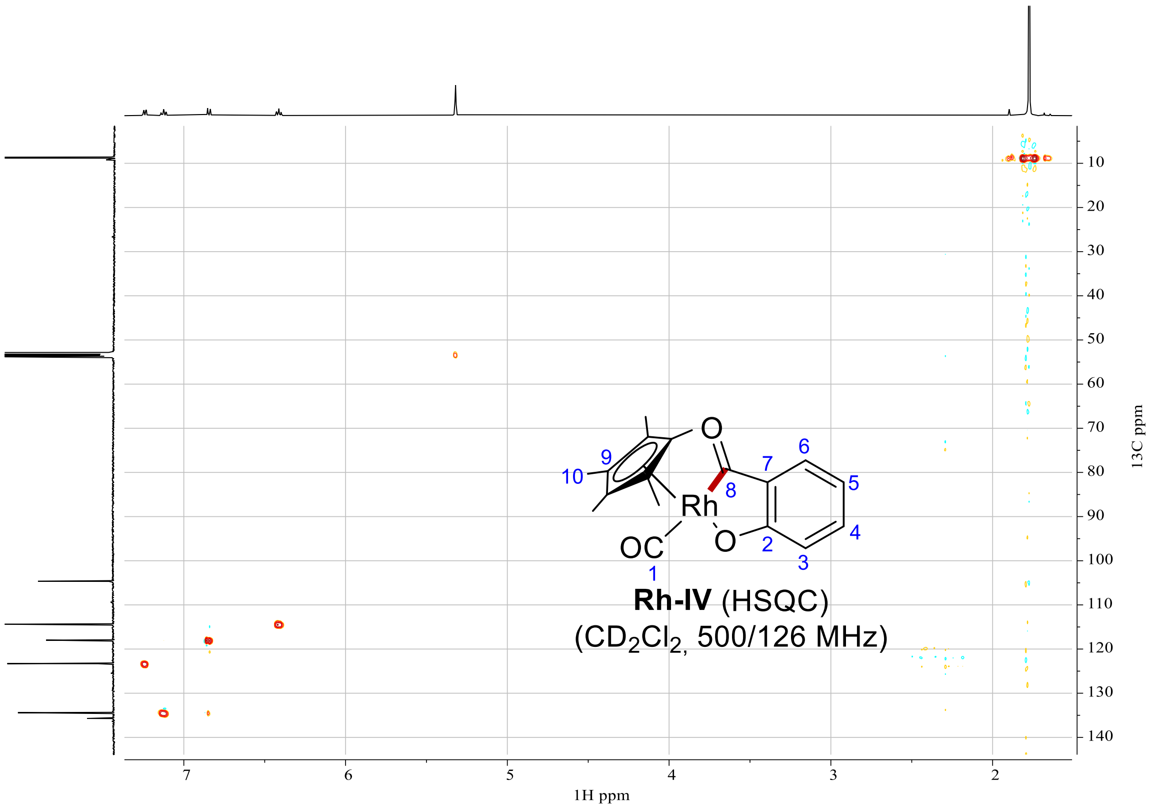

Supplementary Figure 38. 2D-NMR (HSQC) spectrum of **Rh-IV**.

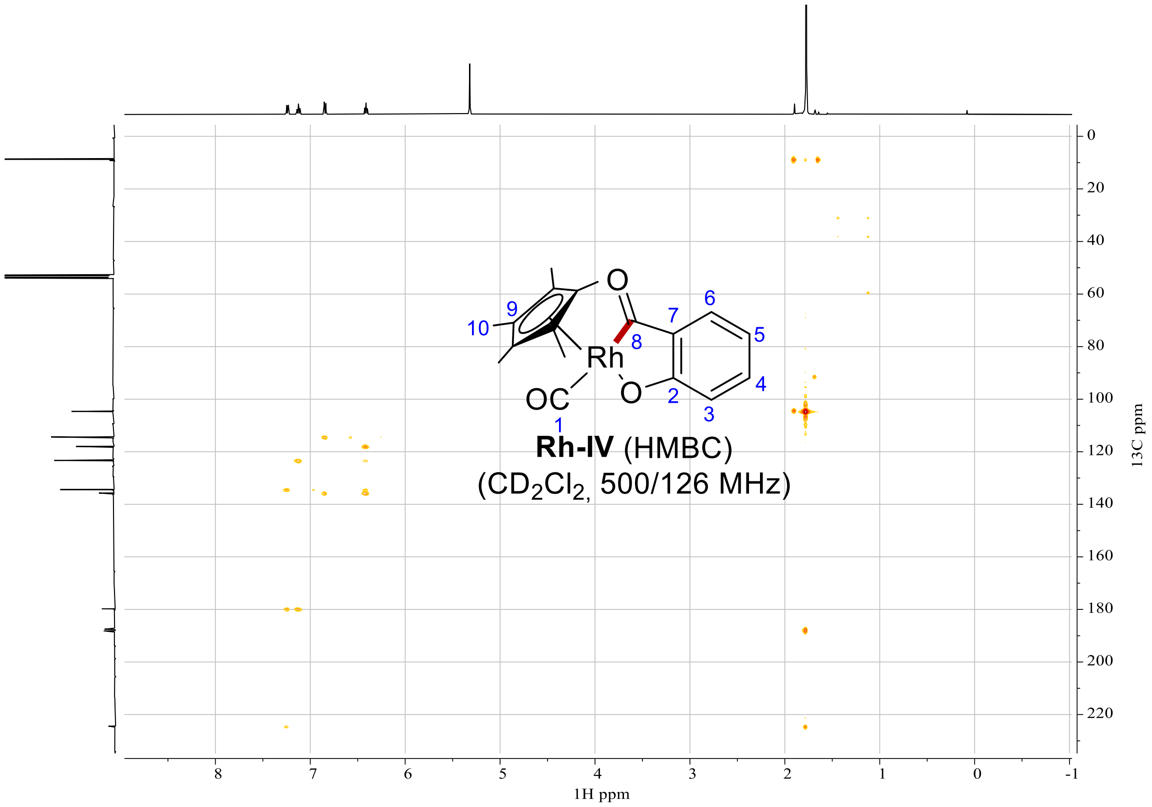

Supplementary Figure 39. 2D-NMR (HMBC) spectrum of **Rh-IV**.

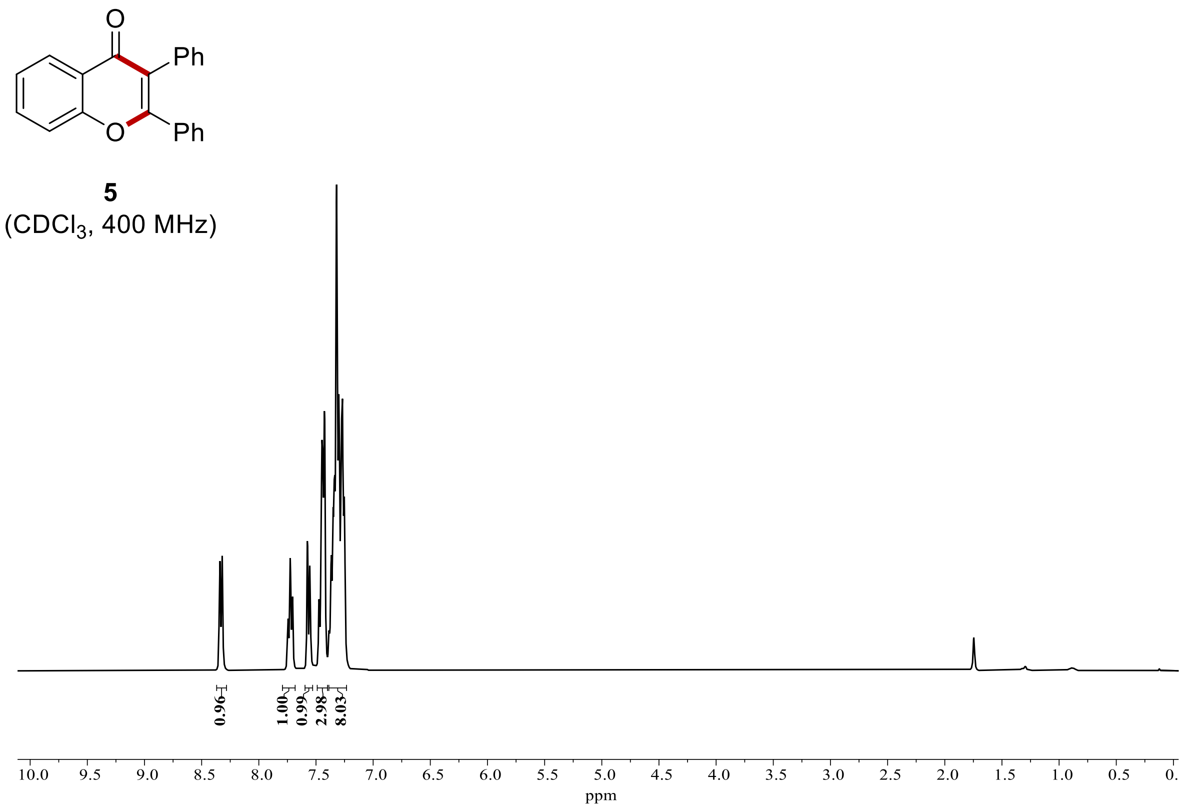

Supplementary Figure 40. ^{1}H-NMR spectrum of **5**.

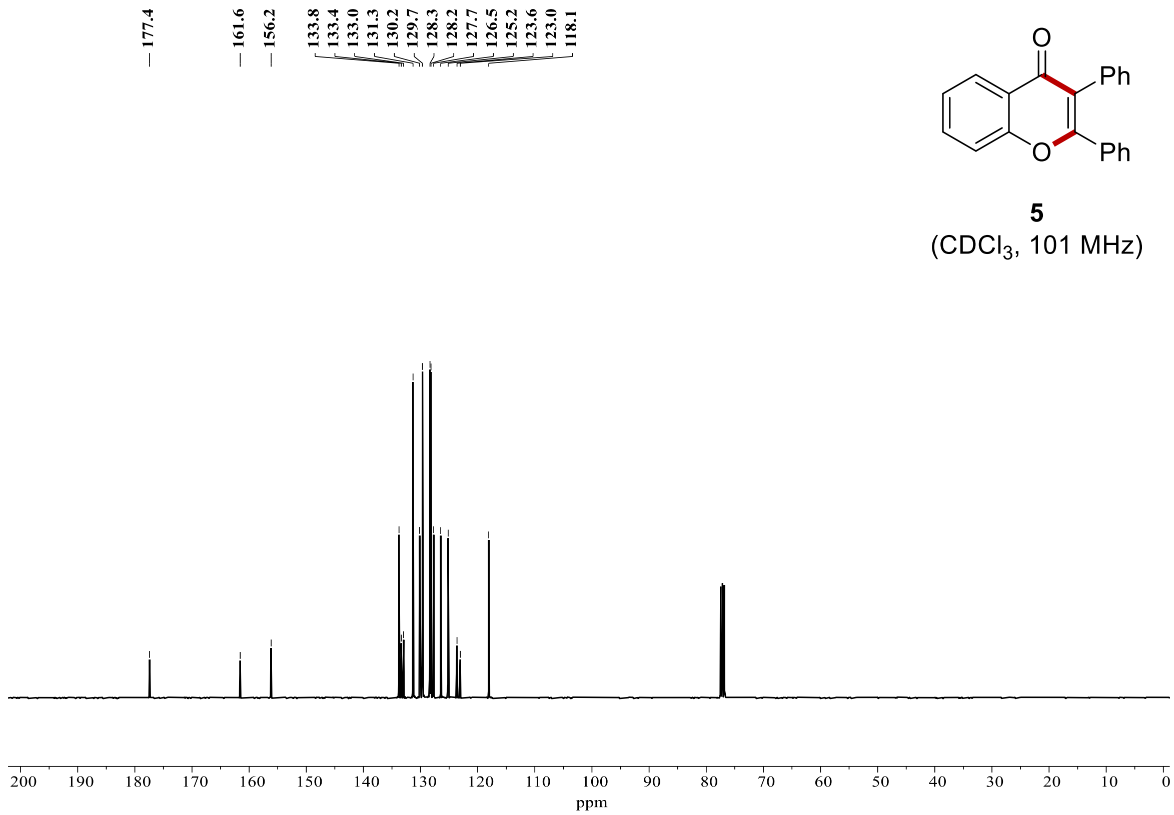

Supplementary Figure 41. ^{13}C-NMR spectrum of **5**.

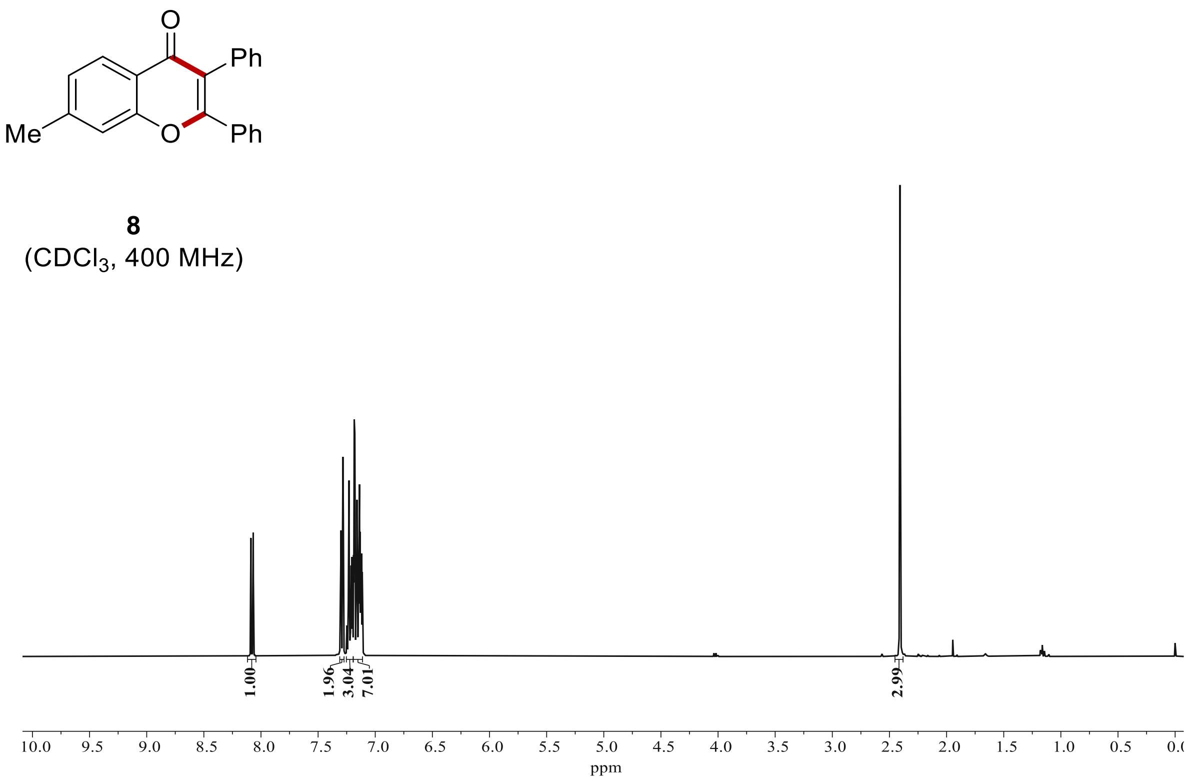

Supplementary Figure 42. ^{1}H-NMR spectrum of **8**.

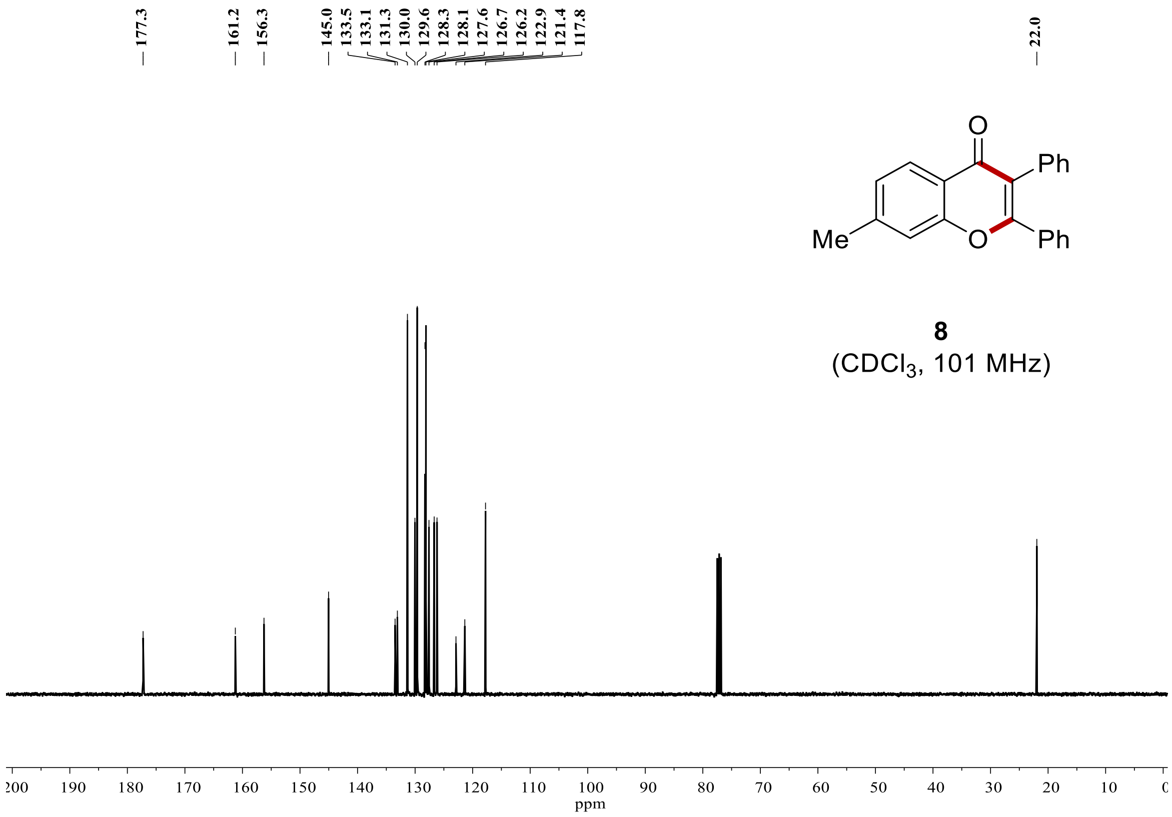

Supplementary Figure 43. ^{13}C-NMR spectrum of **8**.

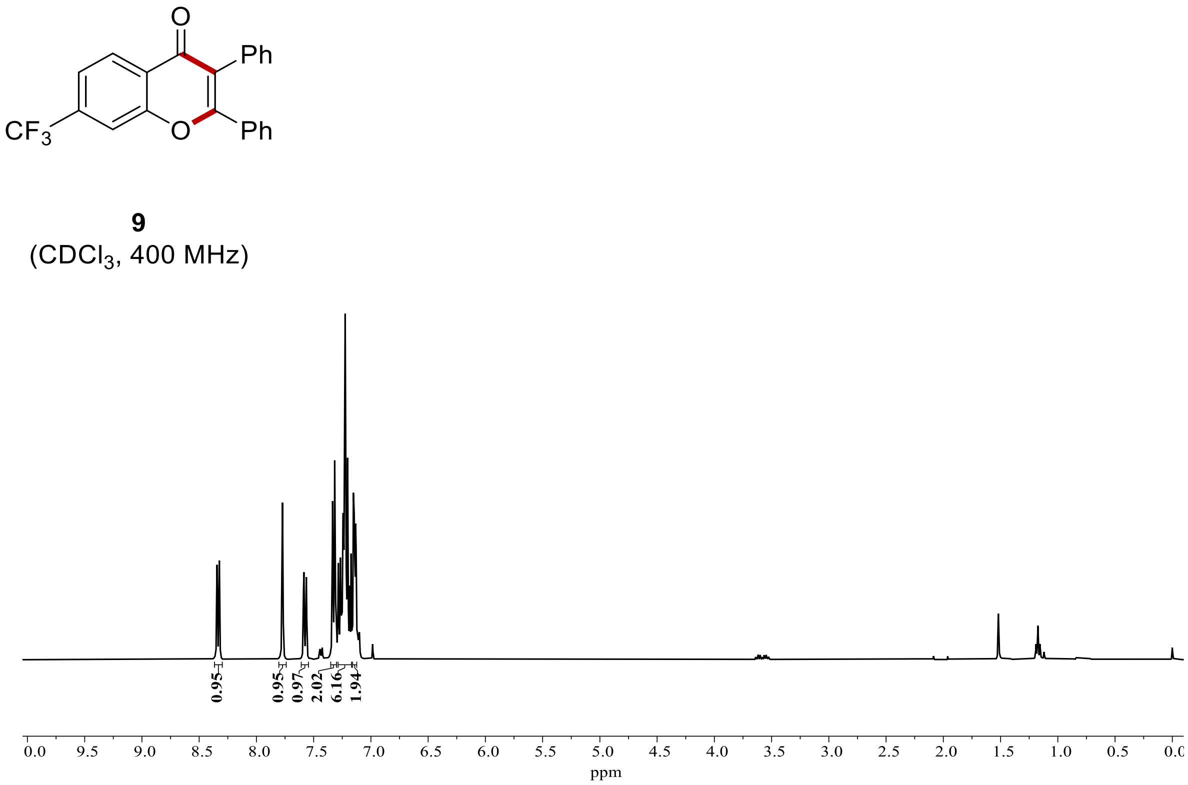

Supplementary Figure 44. ^{1}H-NMR spectrum of **9**.

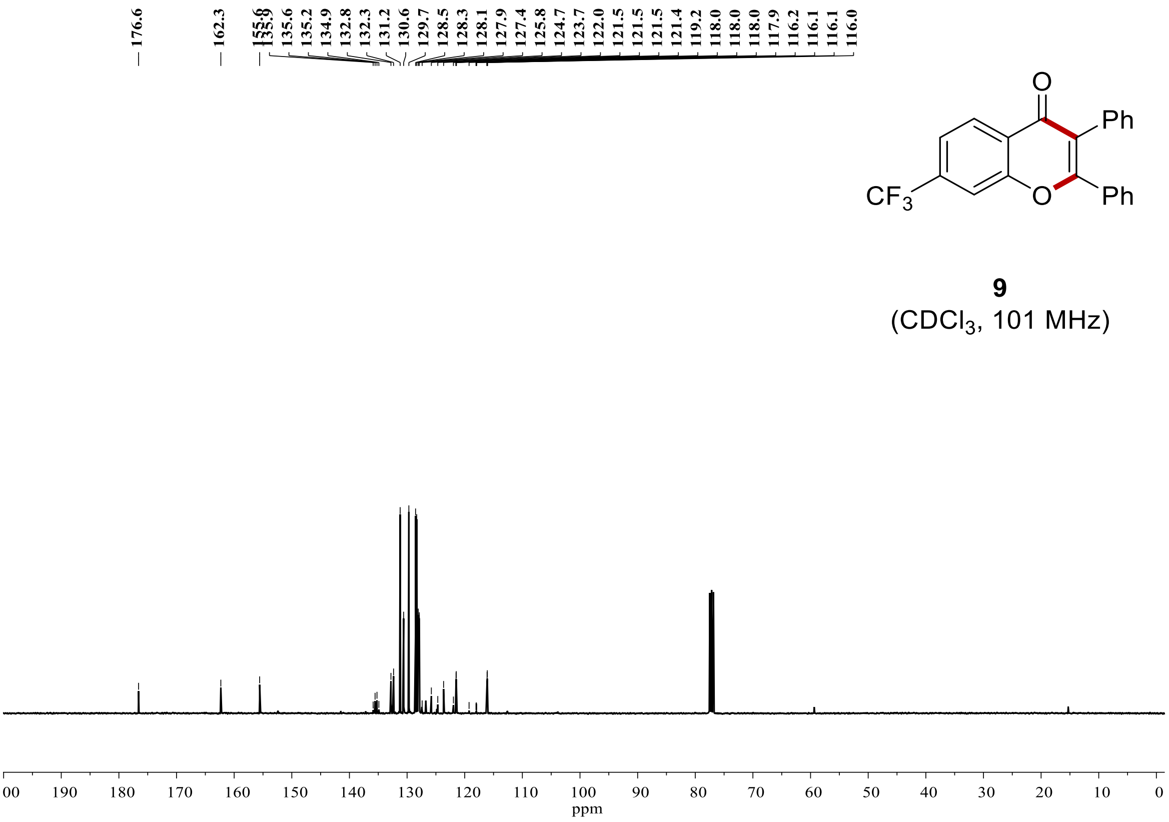

Supplementary Figure 45. ^{13}C-NMR spectrum of **9**.

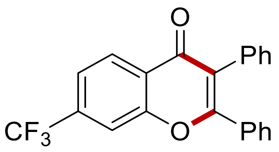

9
($CDCl_3$, 377 MHz)

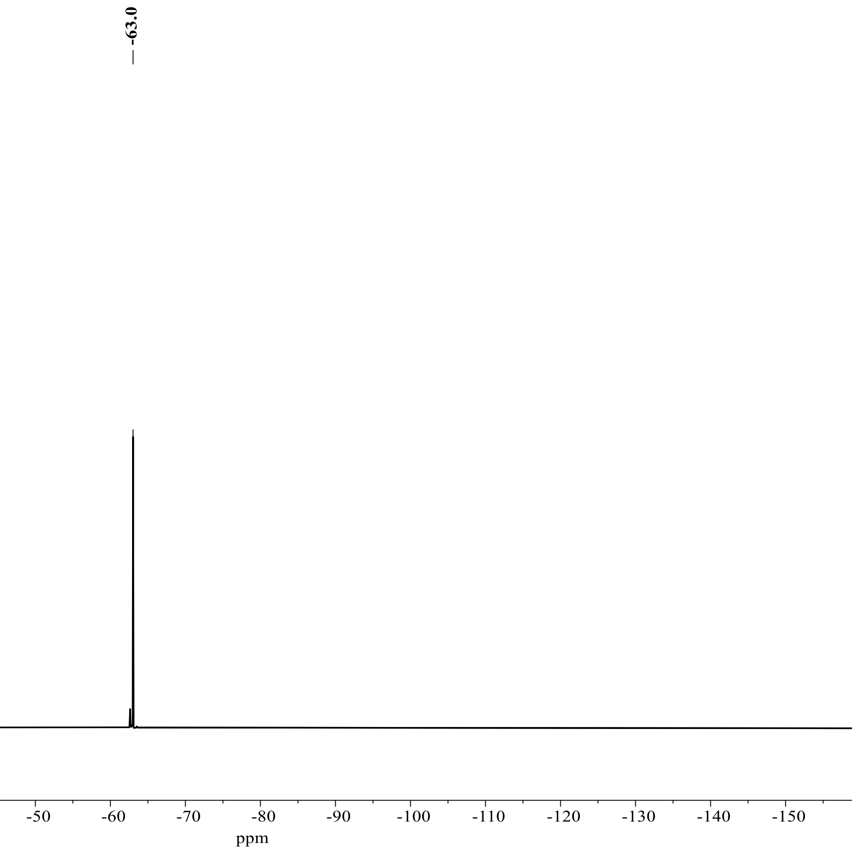

Supplementary Figure 46. ^{19}F-NMR spectrum of **9**.

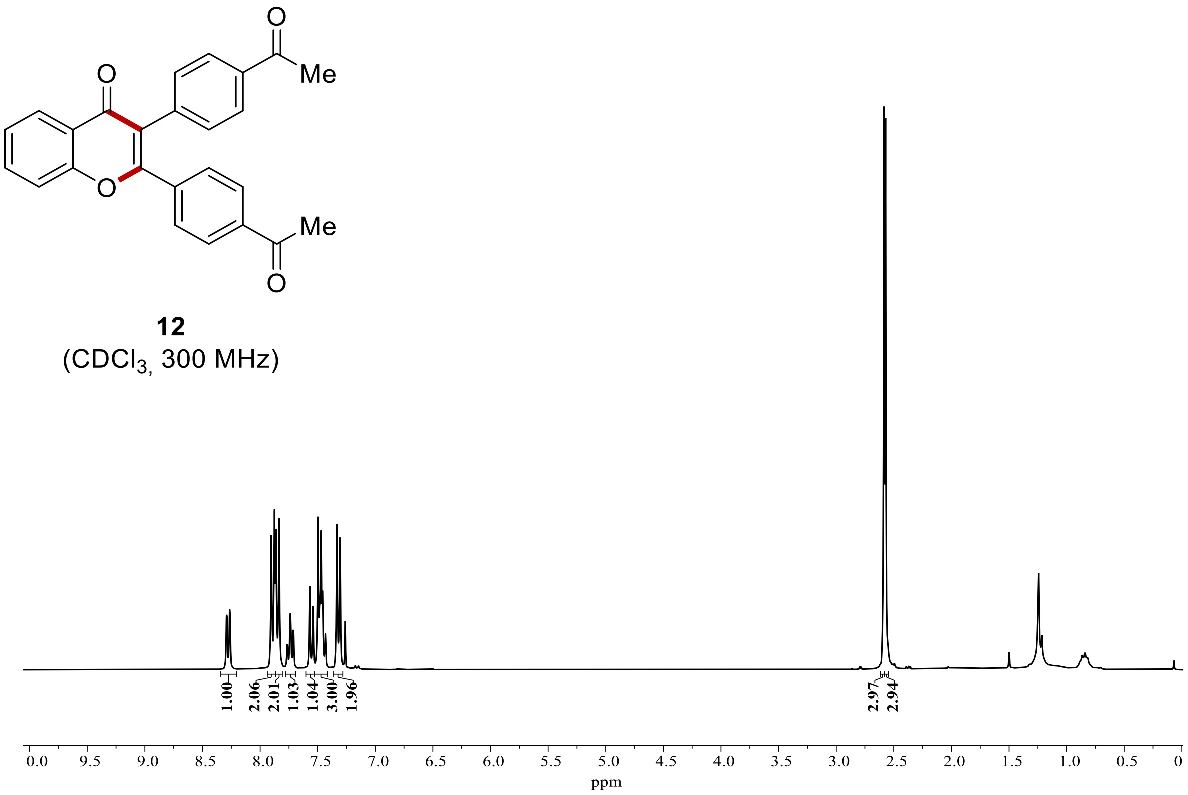

Supplementary Figure 47. ^{1}H-NMR spectrum of **12**.

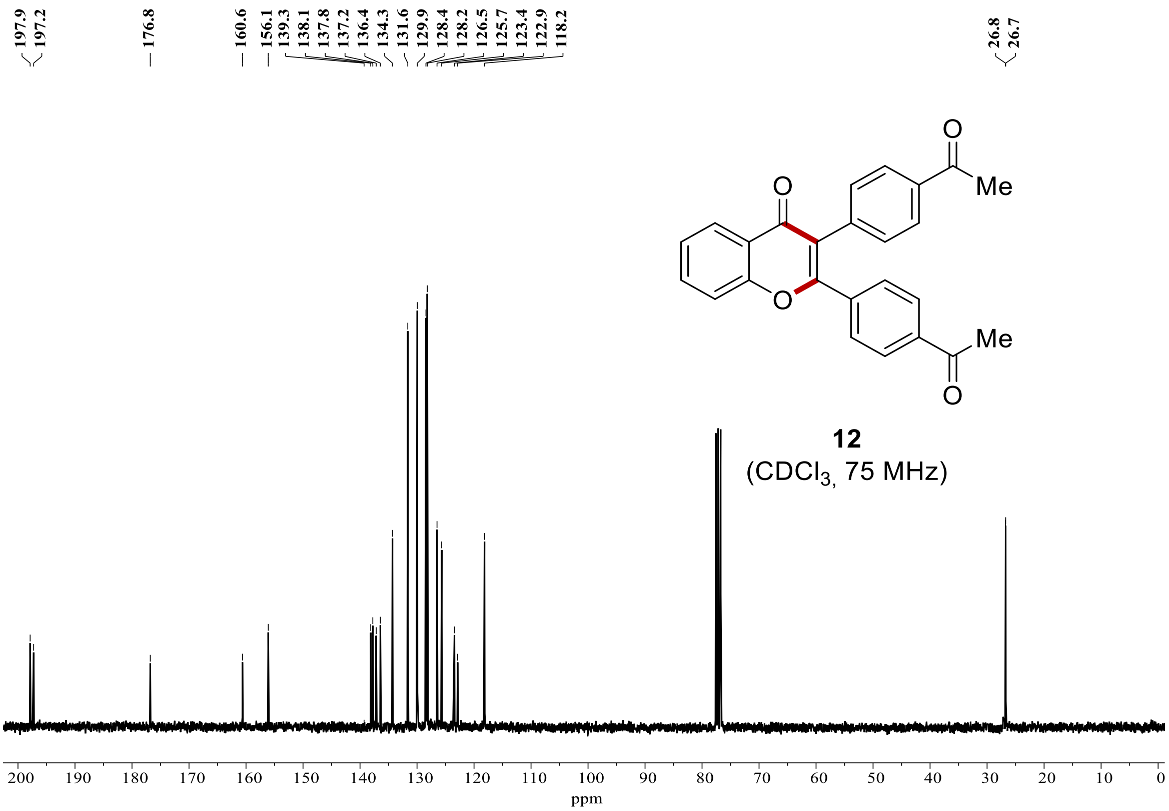

Supplementary Figure 48. ^{13}C-NMR spectrum of **12**.

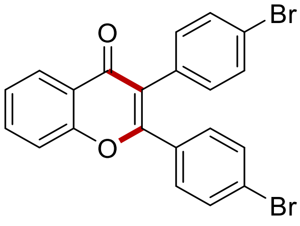

13
($CDCl_3$, 400 MHz)

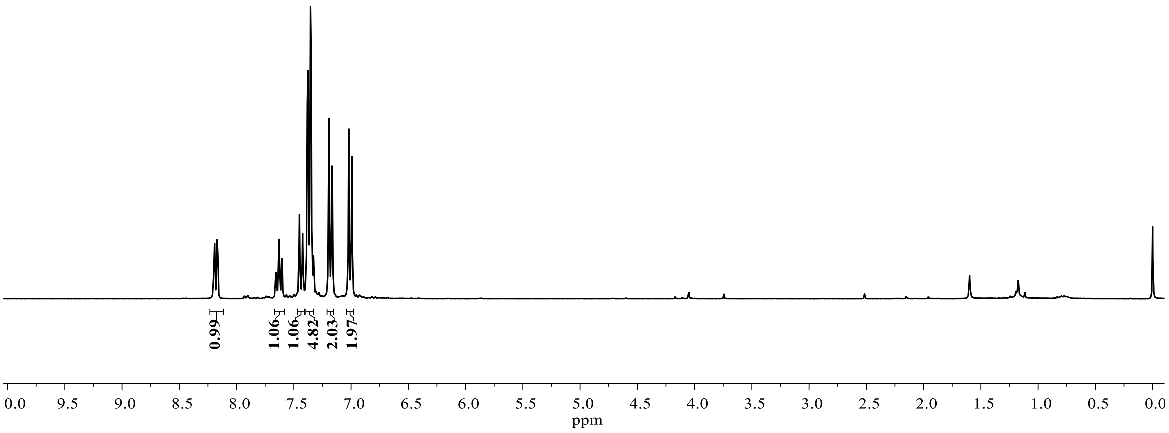

Supplementary Figure 49. ^{1}H-NMR spectrum of **13**.

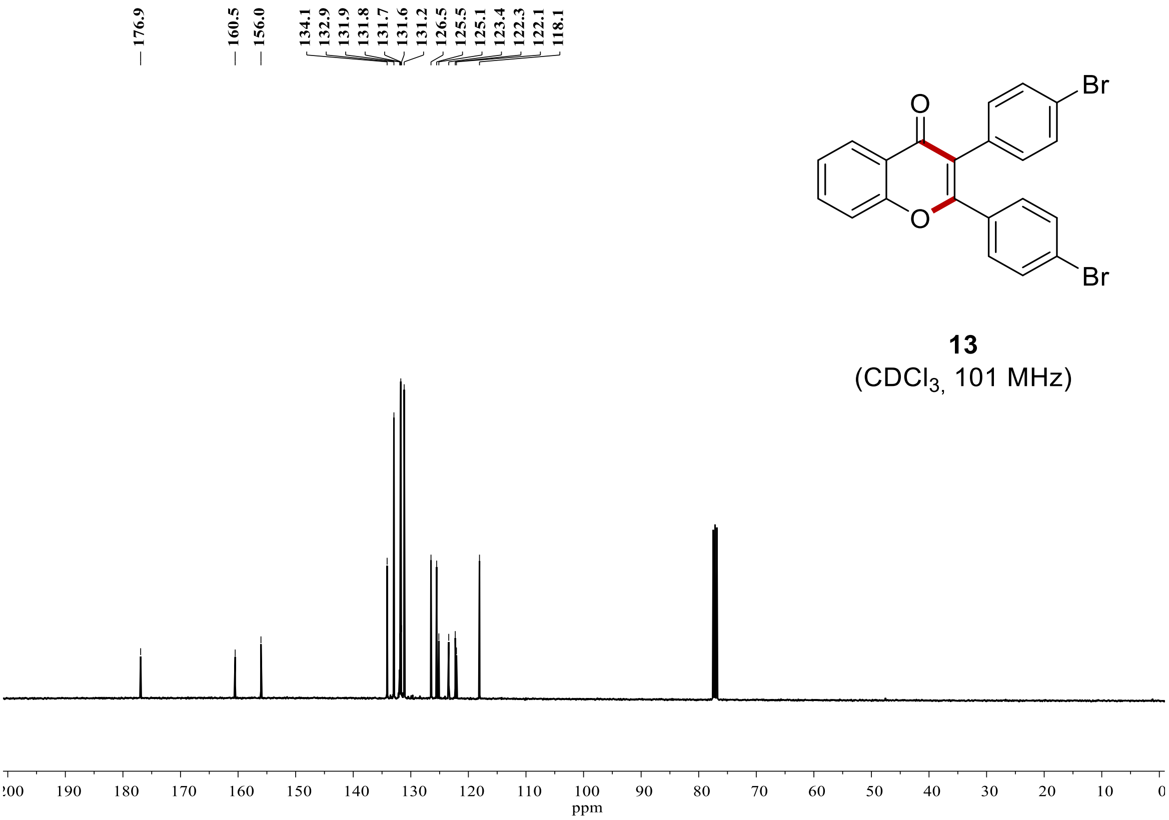

13
($CDCl_3$, 101 MHz)

Supplementary Figure 50. ^{13}C-NMR spectrum of **13**.

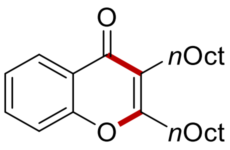

14
($CDCl_3$, 400 MHz)

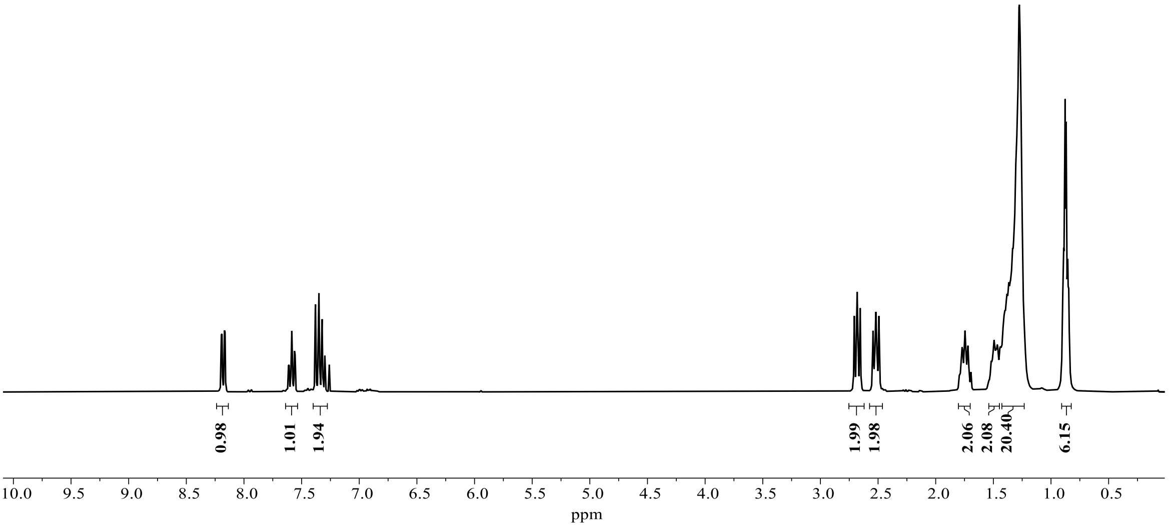

Supplementary Figure 51. ^{1}H-NMR spectrum of **14**.

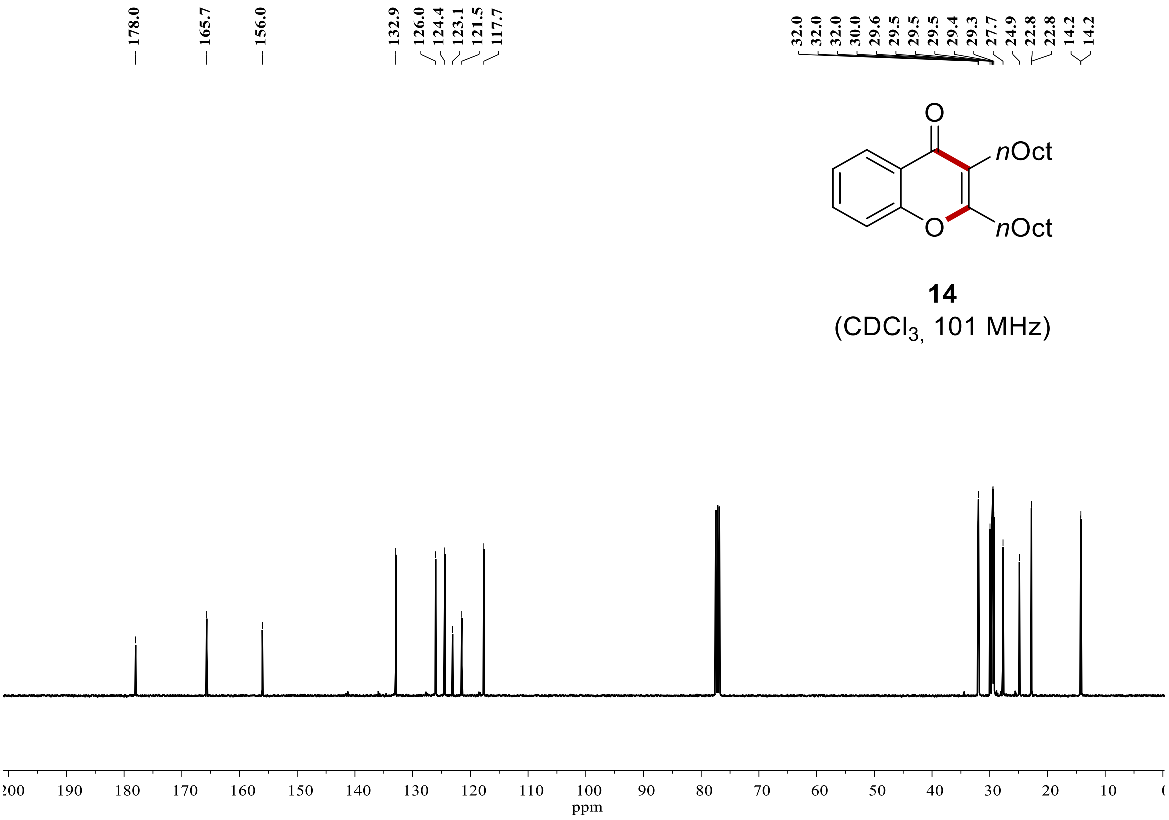

Supplementary Figure 52. ^{13}C-NMR spectrum of **14**.

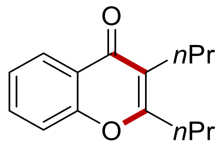

15
($CDCl_3$, 400 MHz)

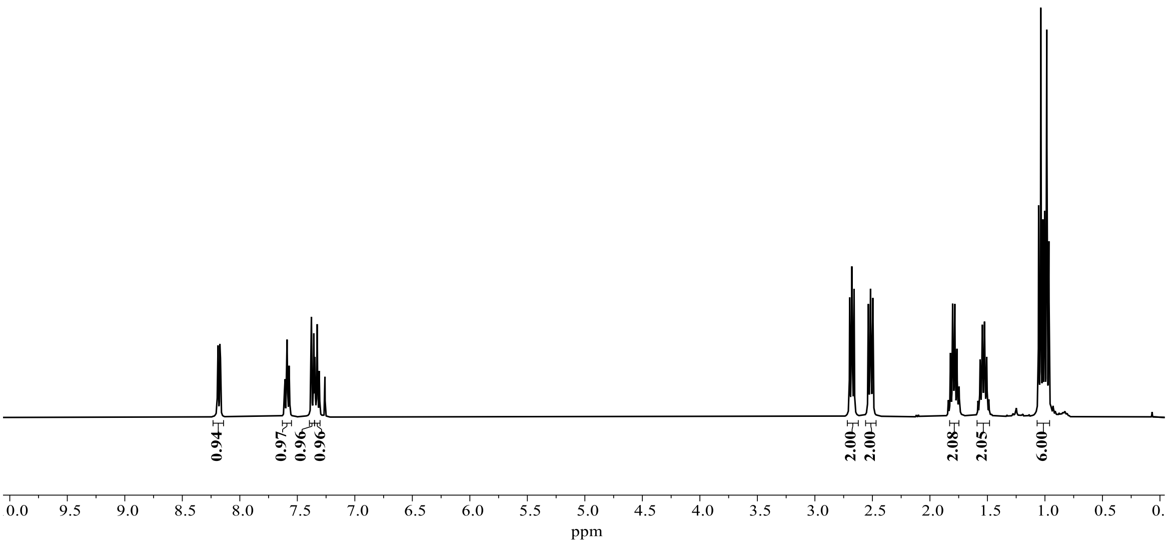

Supplementary Figure 53. ^{1}H-NMR spectrum of **15**.

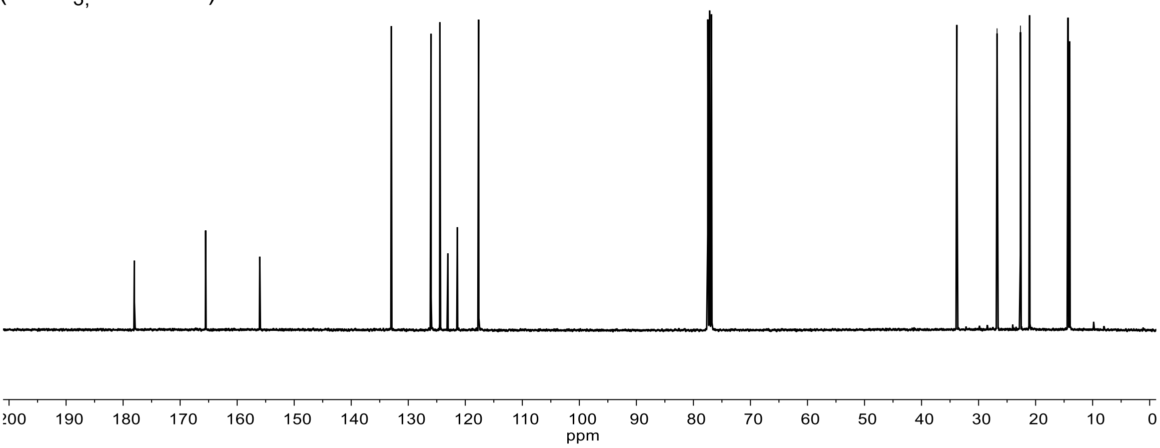

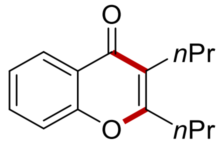

15
($CDCl_3$, 101 MHz)

Supplementary Figure 54. ^{13}C-NMR spectrum of **15**.

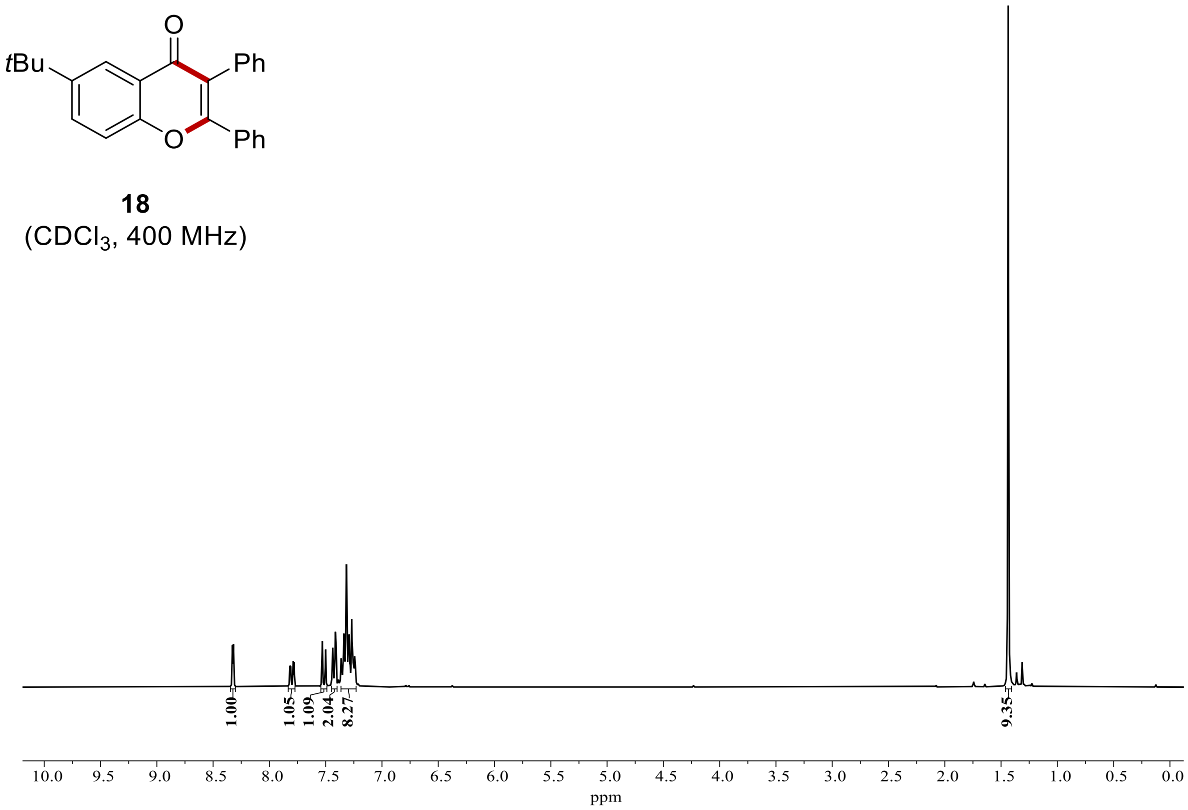

Supplementary Figure 55. ^{1}H-NMR spectrum of **18**.

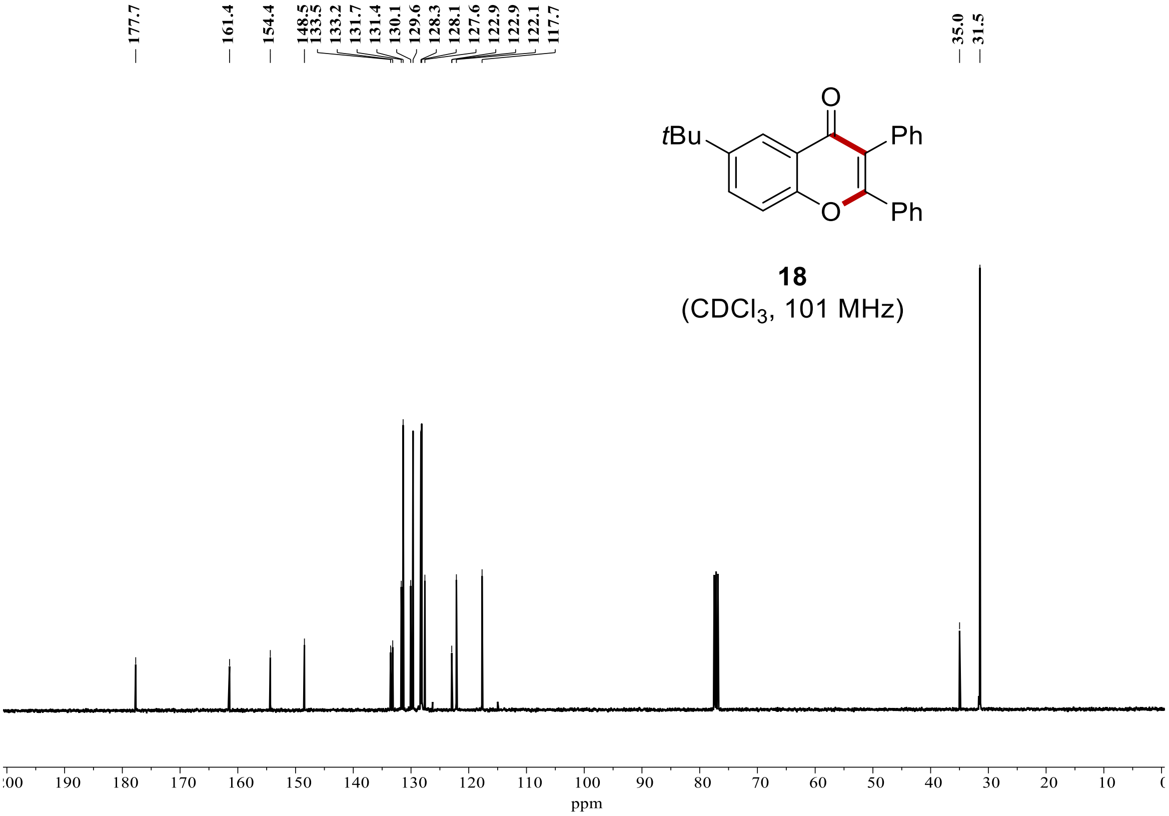

Supplementary Figure 56. ^{13}C-NMR spectrum of **18**.

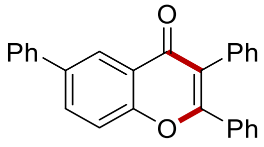

19
($CDCl_3$, 500 MHz)

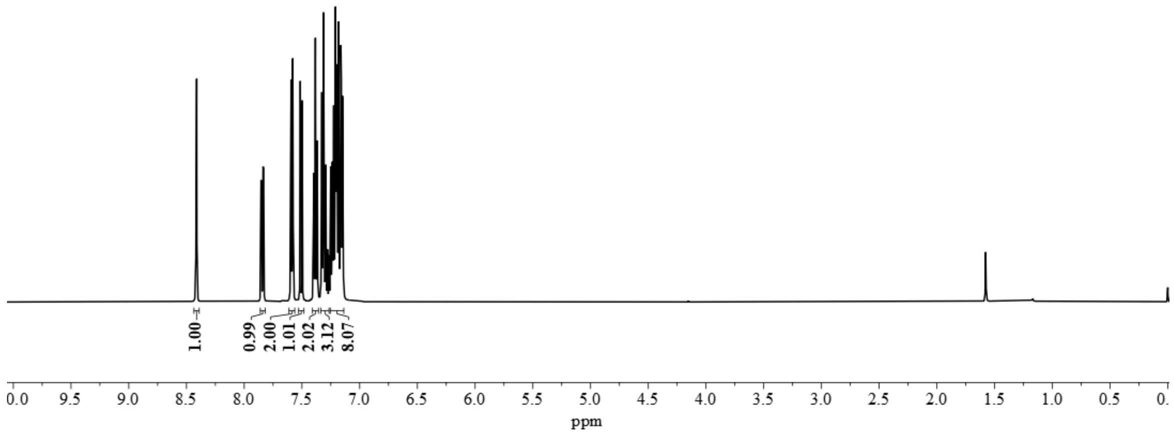

Supplementary Figure 57. ^{1}H-NMR spectrum of **19**.

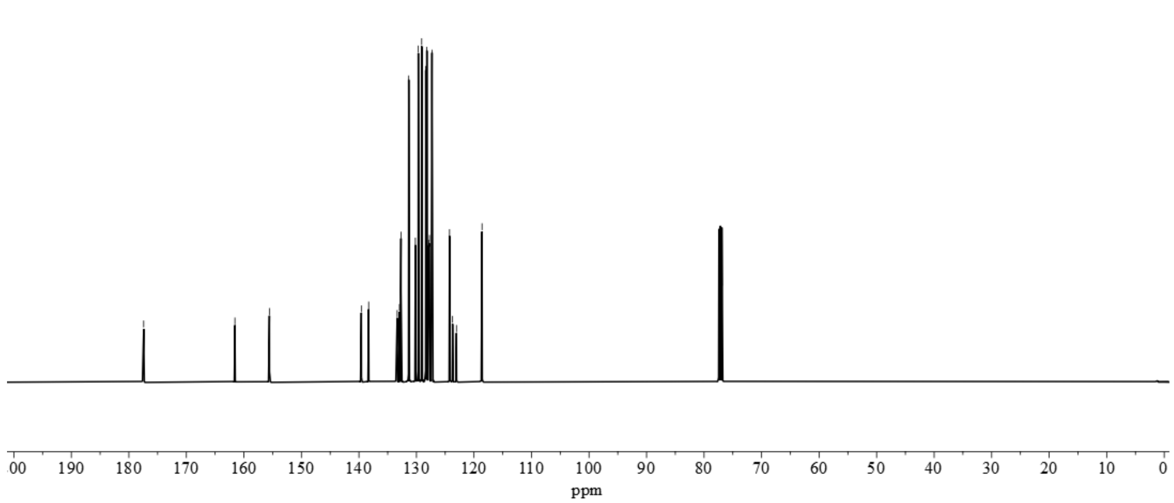

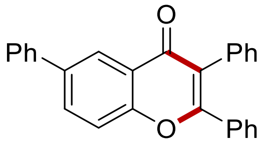

19
($CDCl_3$, 126 MHz)

Supplementary Figure 58. ^{13}C-NMR spectrum of **19**.

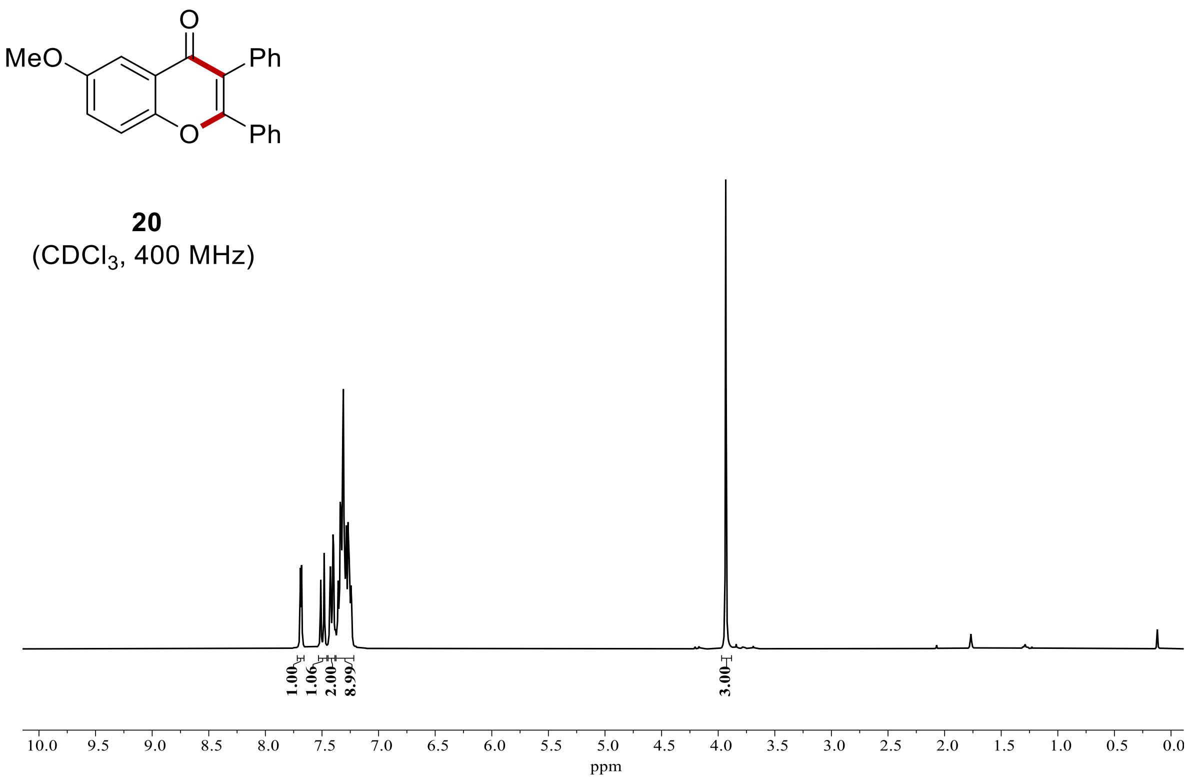

Supplementary Figure 59. ^{1}H-NMR spectrum of **20**.

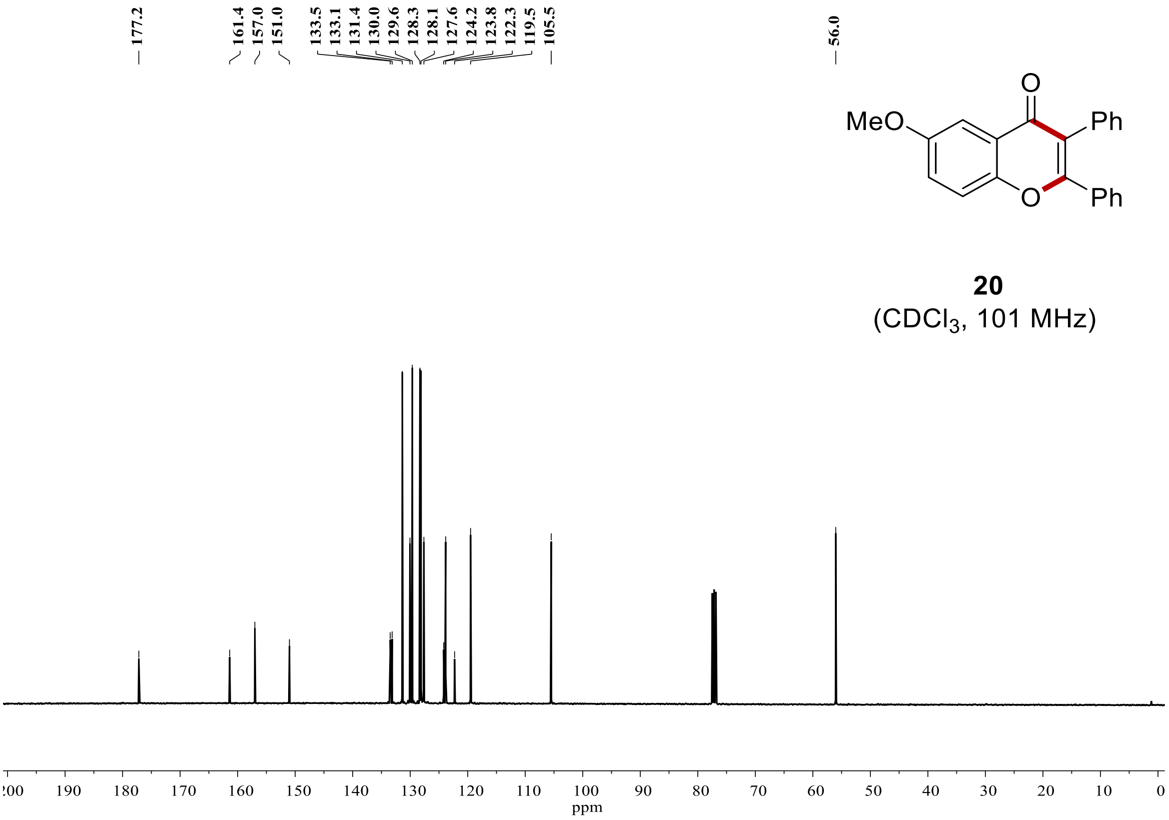

Supplementary Figure 60. ^{13}C-NMR spectrum of **20**.

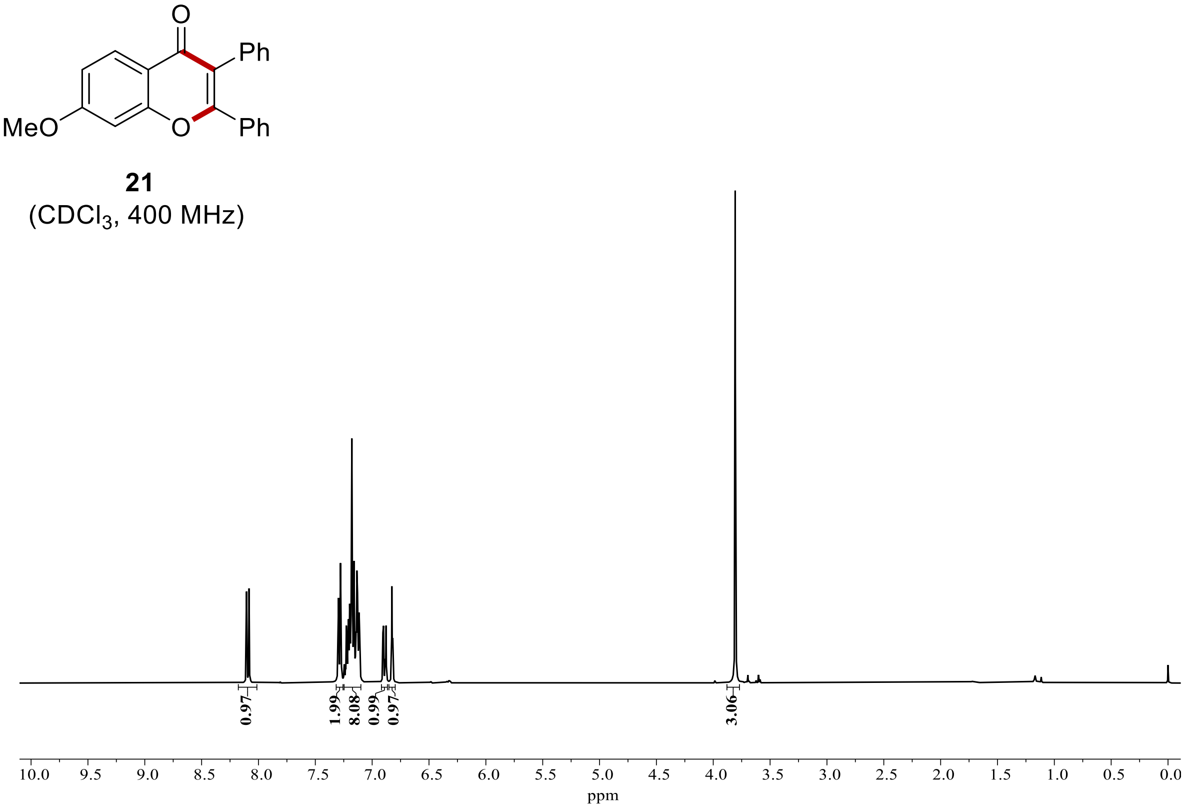

Supplementary Figure 61. ^{1}H-NMR spectrum of **21**.

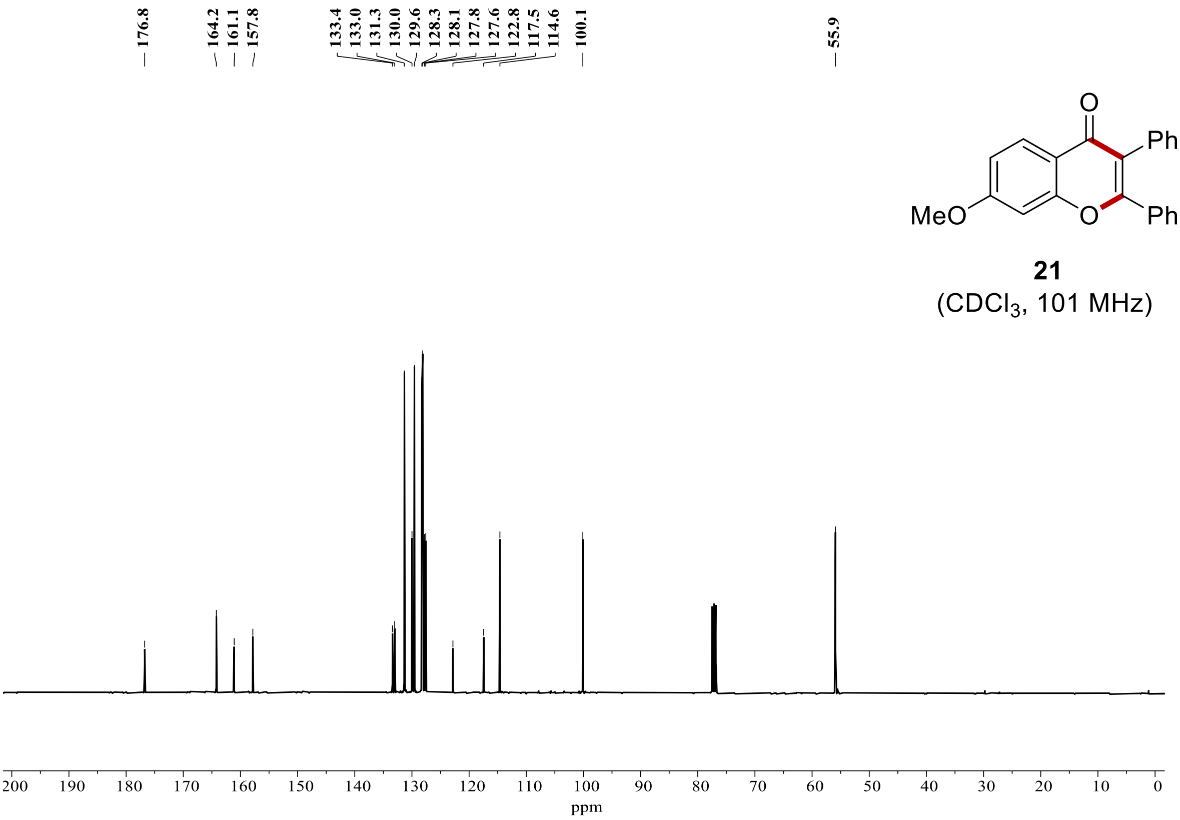

Supplementary Figure 62. ^{13}C-NMR spectrum of **21**.

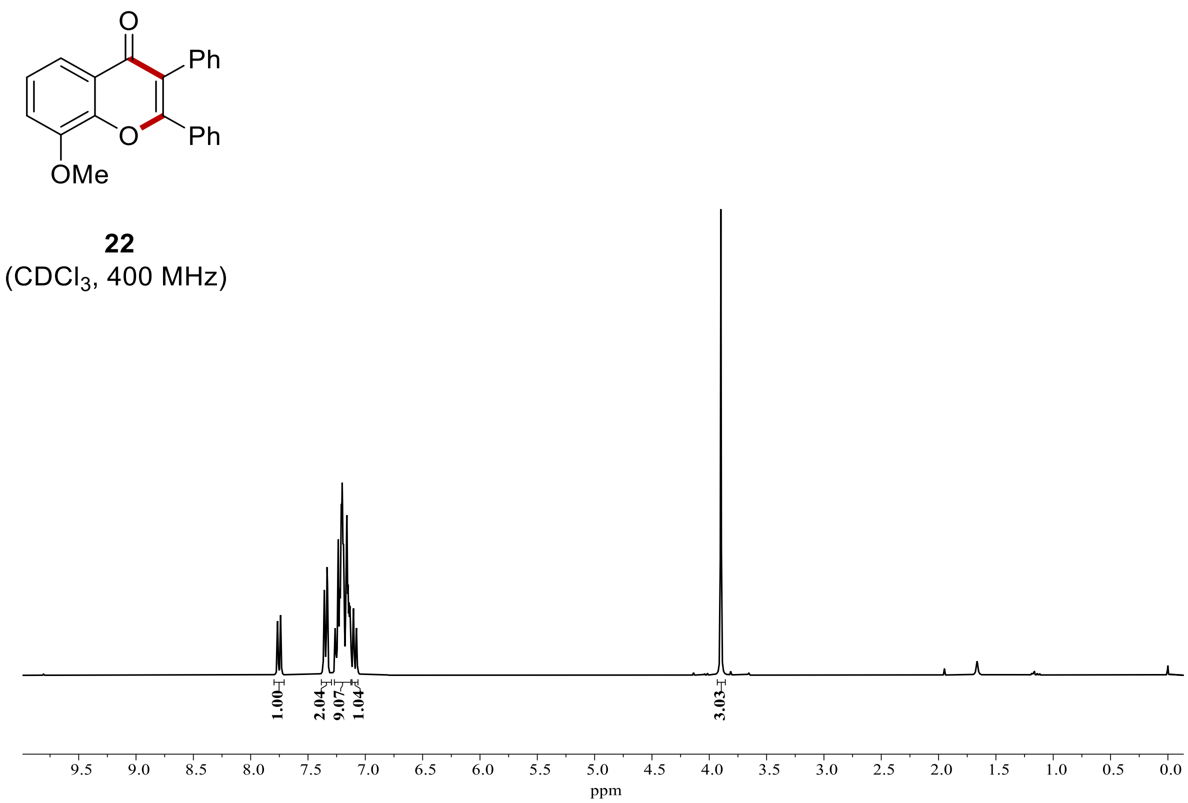

Supplementary Figure 63. ^{1}H-NMR spectrum of **22**.

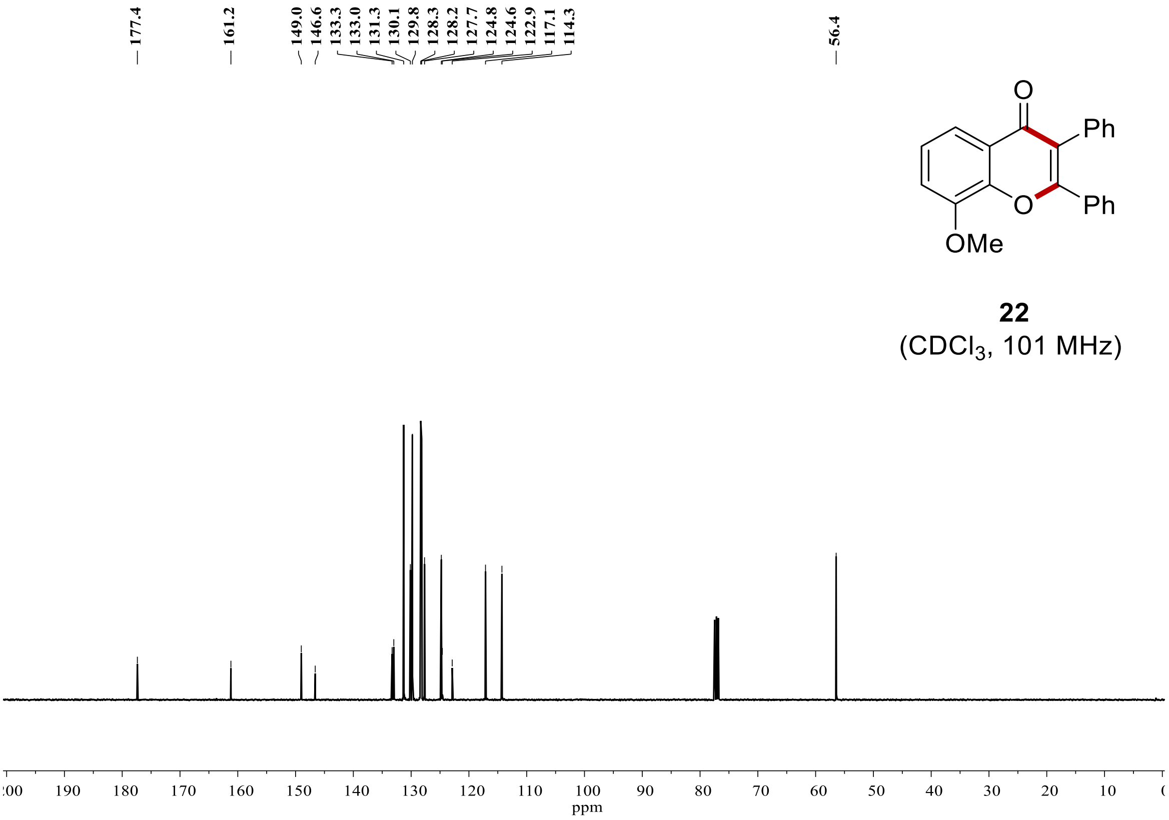

Supplementary Figure 64. ^{13}C-NMR spectrum of **22**.

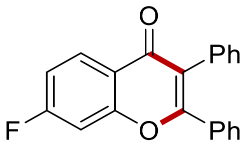

23
($CDCl_3$, 400 MHz)

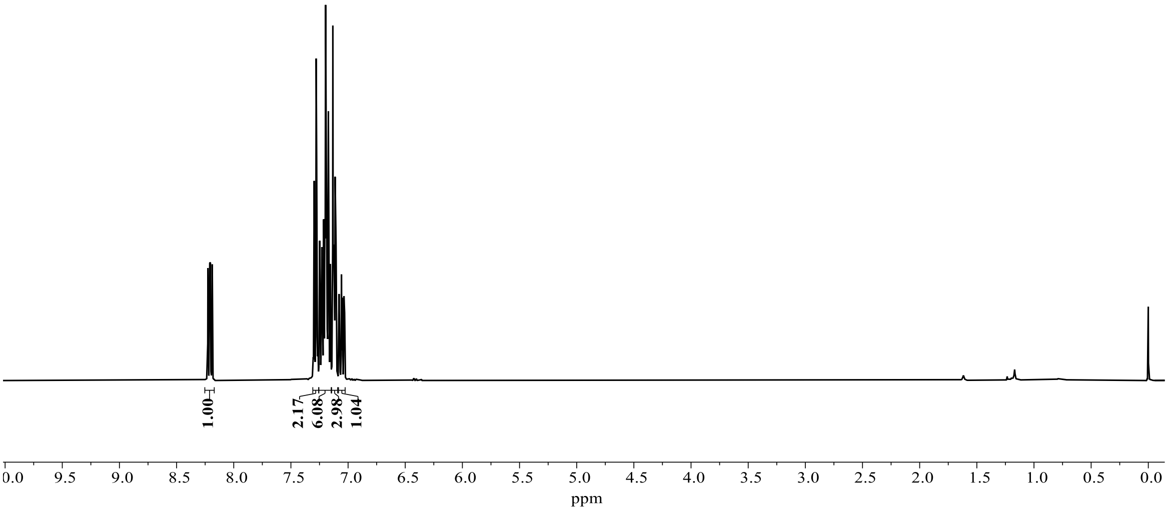

Supplementary Figure 65. ^{1}H-NMR spectrum of **23**.

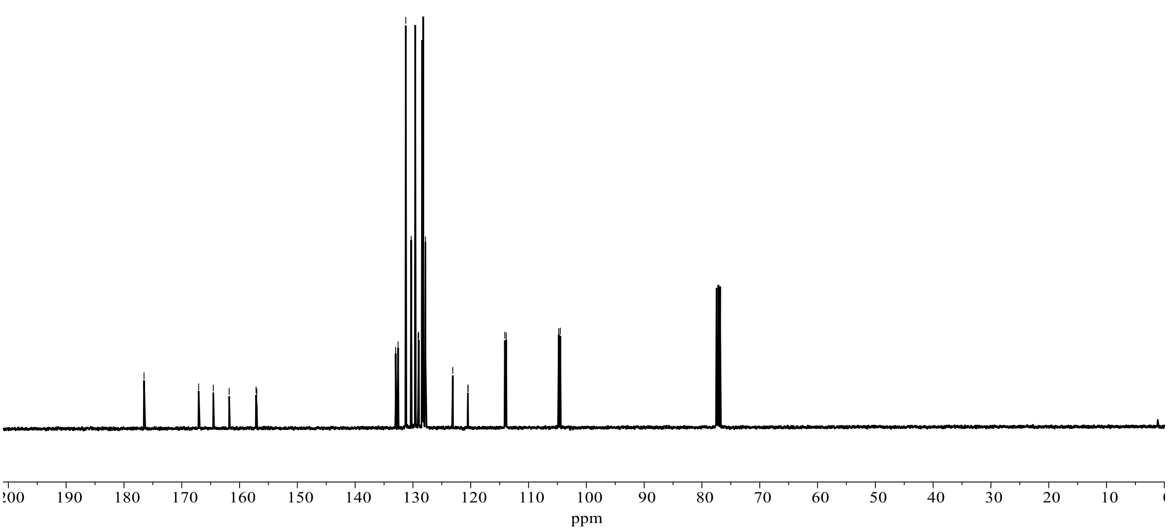

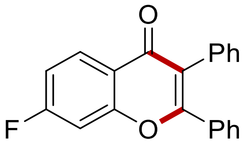

23
($CDCl_3$, 101 MHz)

Supplementary Figure 66. ^{13}C-NMR spectrum of **23**.

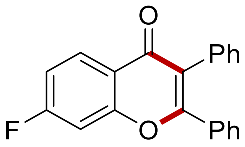

23
($CDCl_3$, 282 MHz)

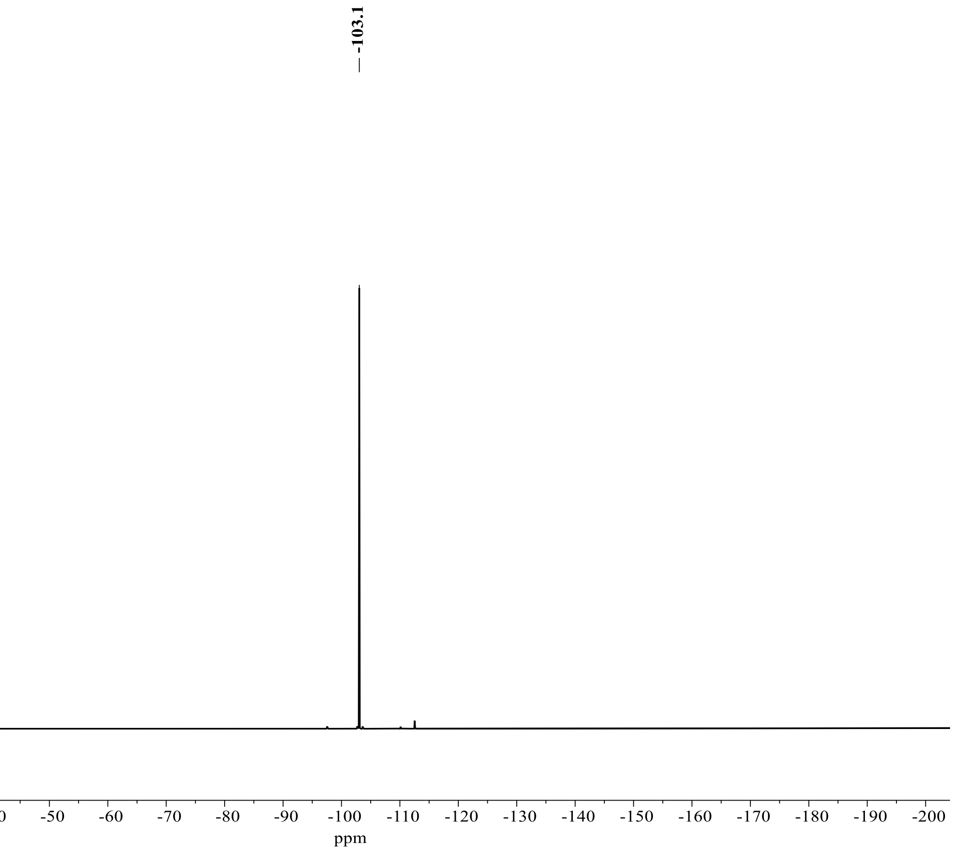

Supplementary Figure 67. ^{19}F-NMR spectrum of **23**.

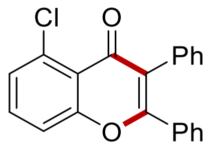

24
($CDCl_3$, 500 MHz)

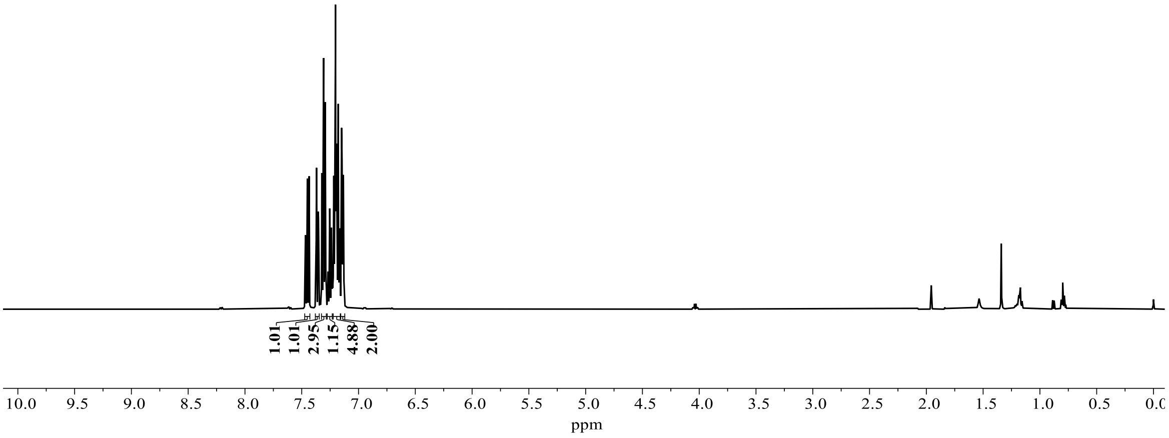

Supplementary Figure 68. ^{1}H-NMR spectrum of **24**.

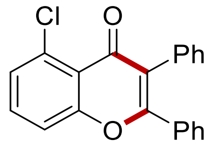

24
($CDCl_3$, 126 MHz)

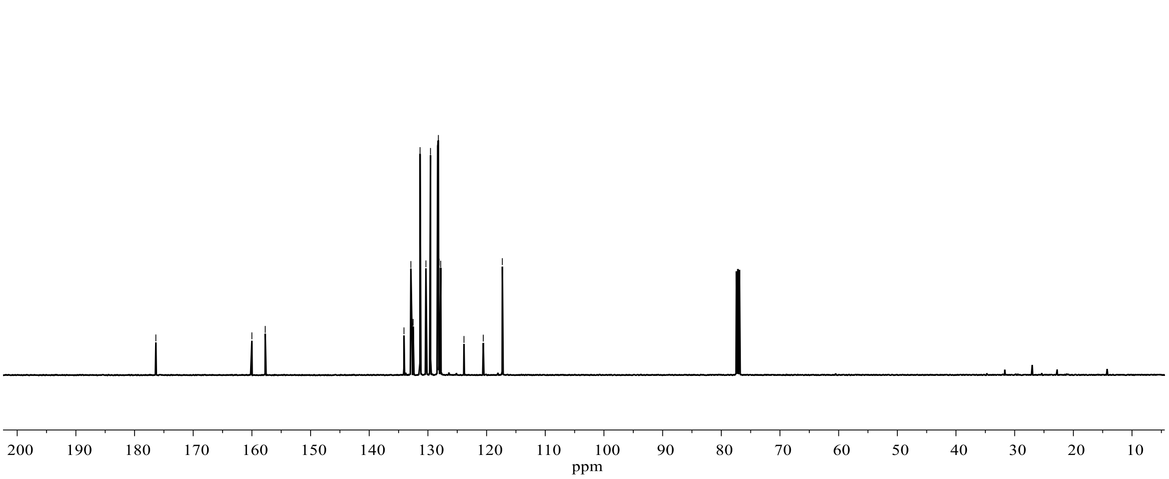

Supplementary Figure 69. ^{13}C-NMR spectrum of **24**.

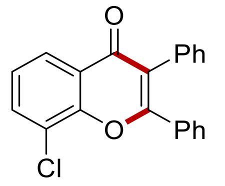

25
($CDCl_3$, 400 MHz)

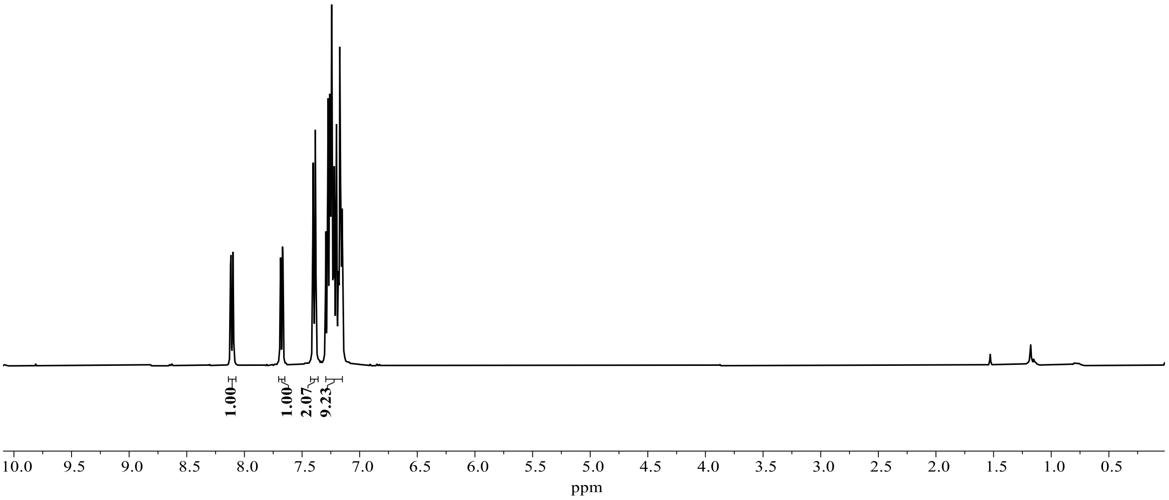

Supplementary Figure 70. ^{1}H-NMR spectrum of **25**.

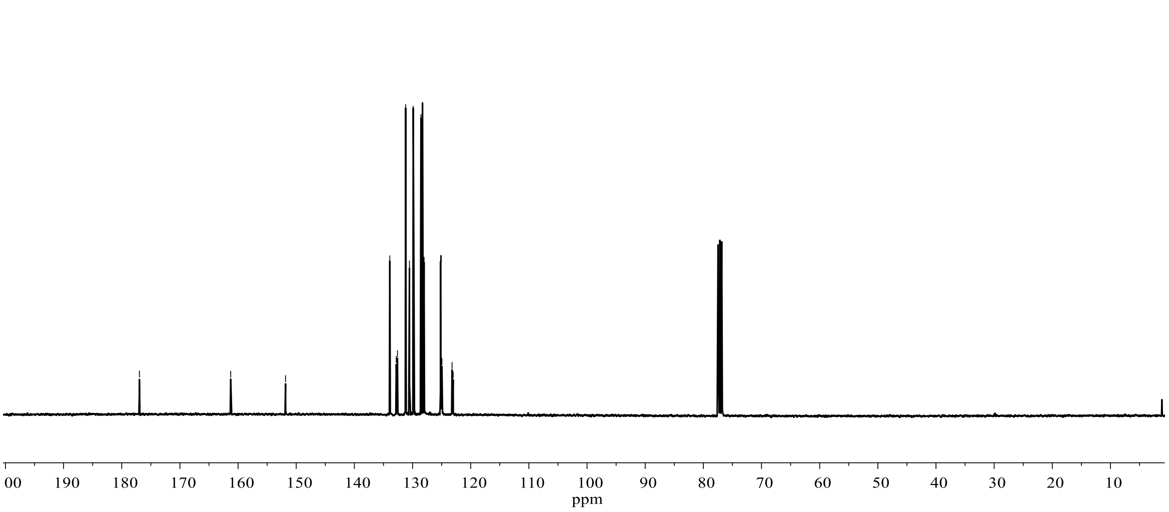

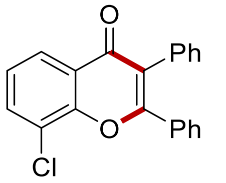

25
($CDCl_3$, 101 MHz)

Supplementary Figure 71. ^{13}C-NMR spectrum of **25**.

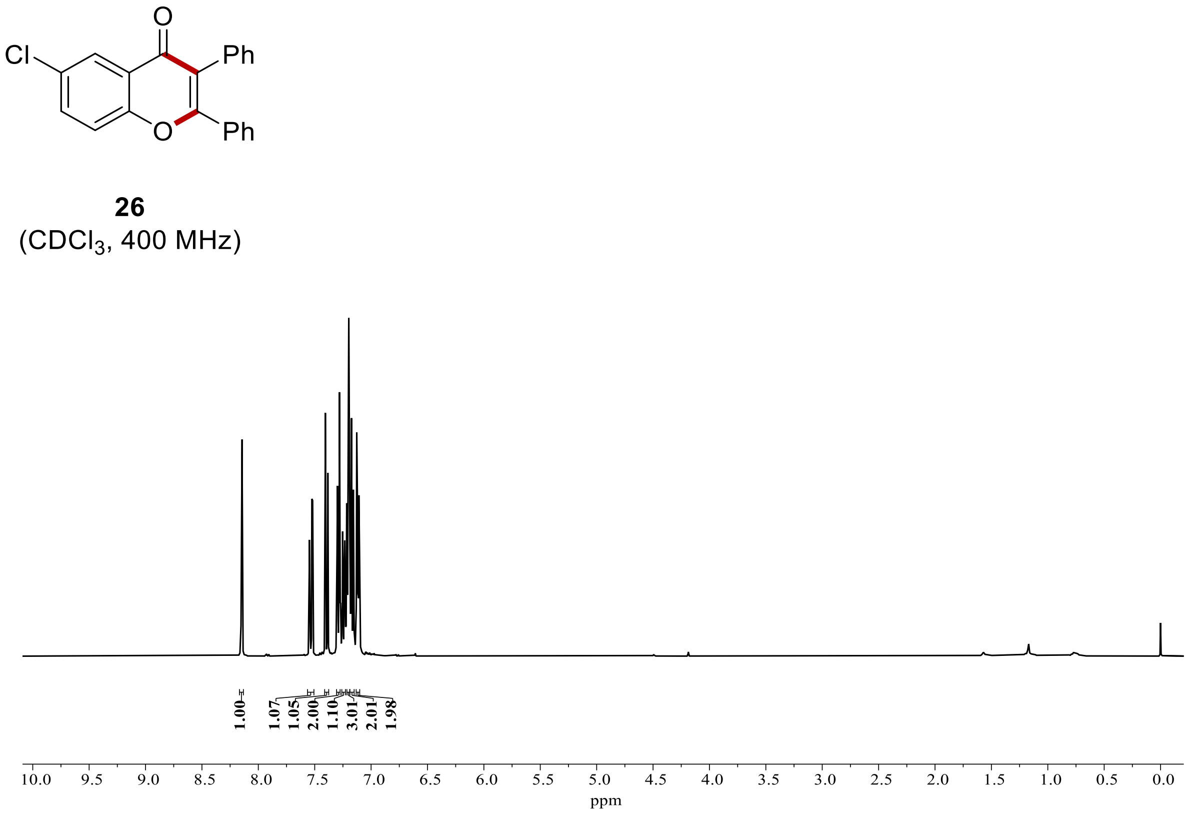

Supplementary Figure 72. ^{1}H-NMR spectrum of **26**.

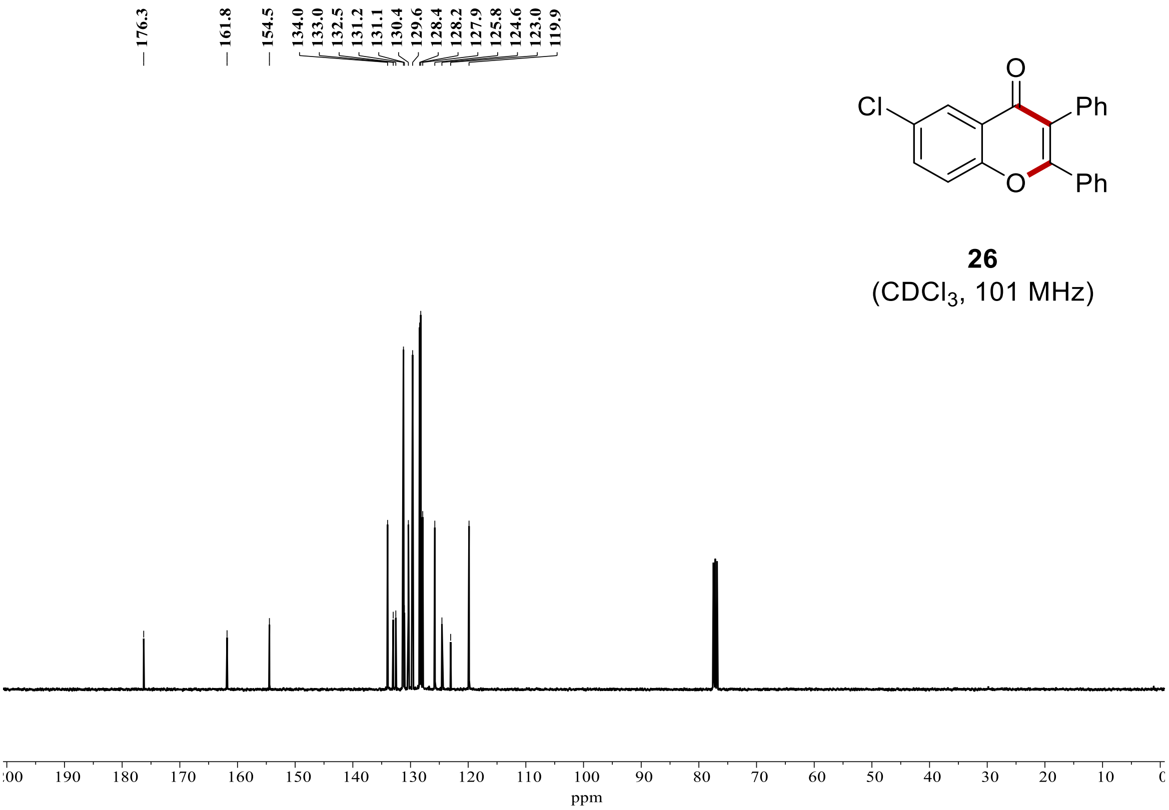

Supplementary Figure 73. ^{13}C-NMR spectrum of **26**.

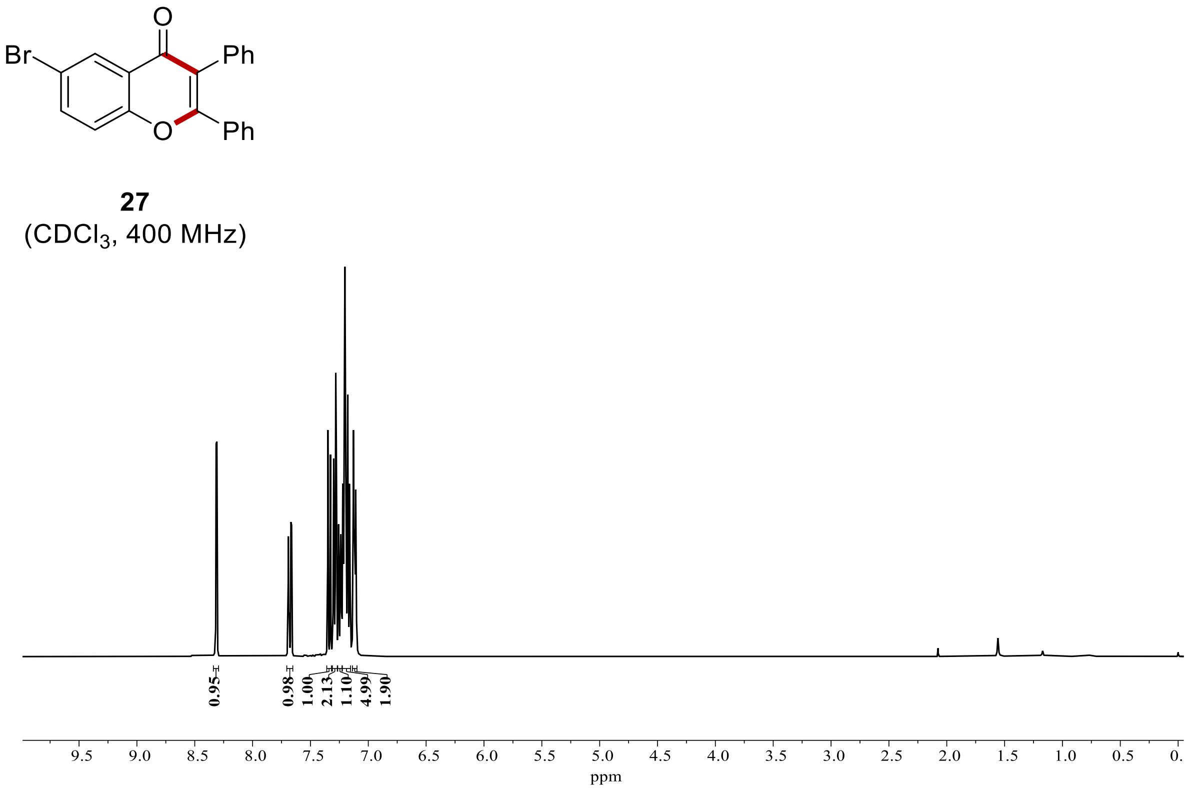

Supplementary Figure 74. ^{1}H-NMR spectrum of **27**.

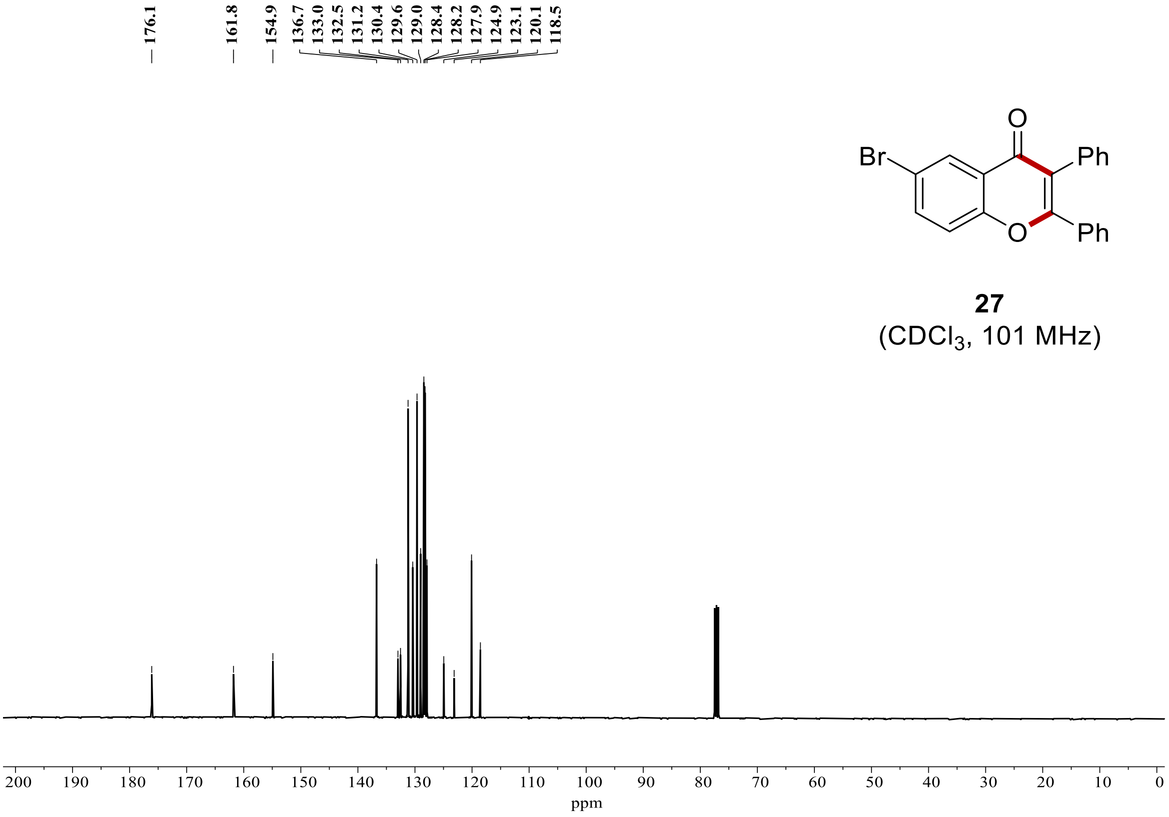

Supplementary Figure 75. ^{13}C-NMR spectrum of **27**.

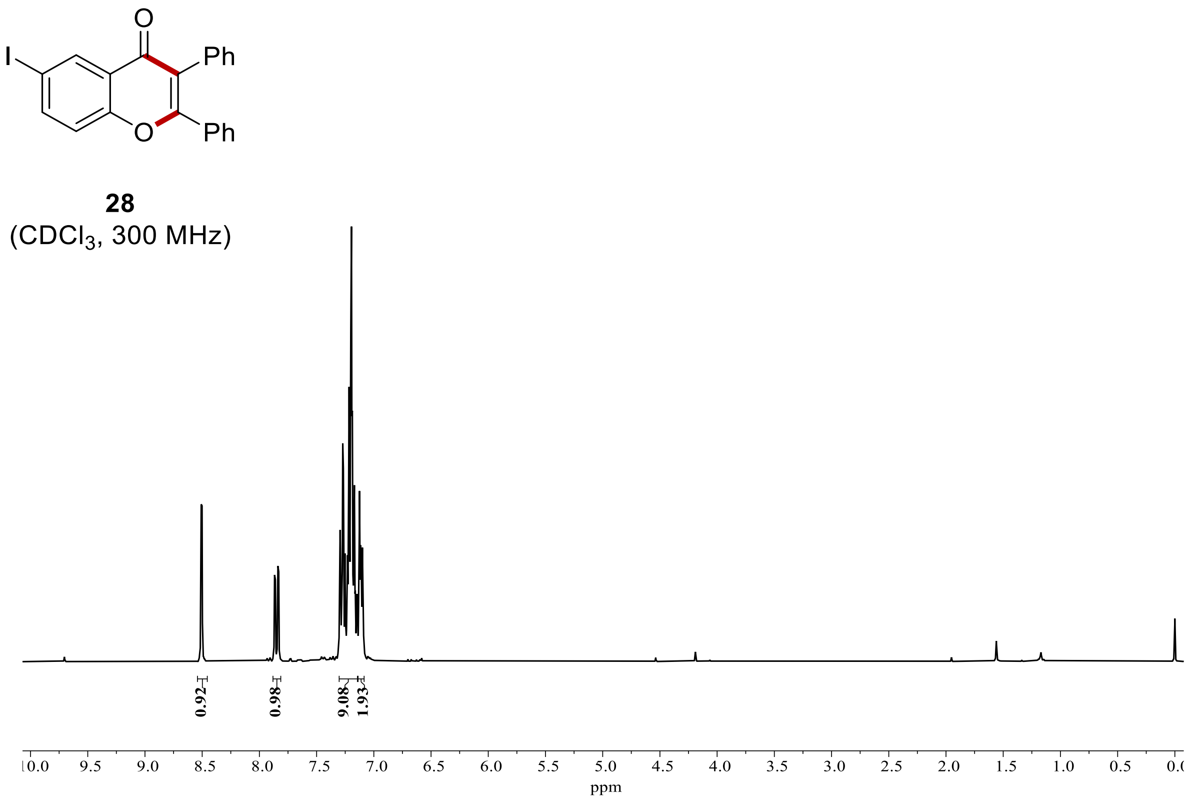

Supplementary Figure 76. ^{1}H-NMR spectrum of **28**.

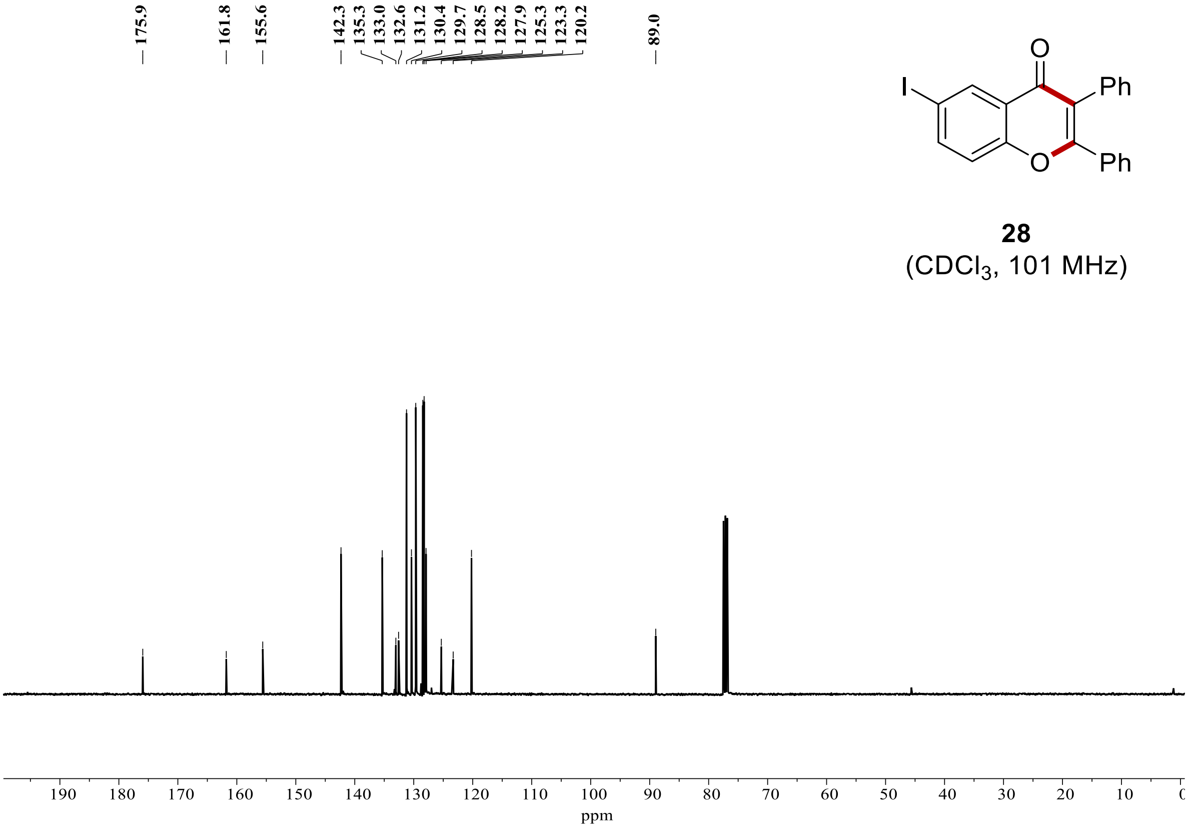

Supplementary Figure 77. ^{13}C-NMR spectrum of **28**.

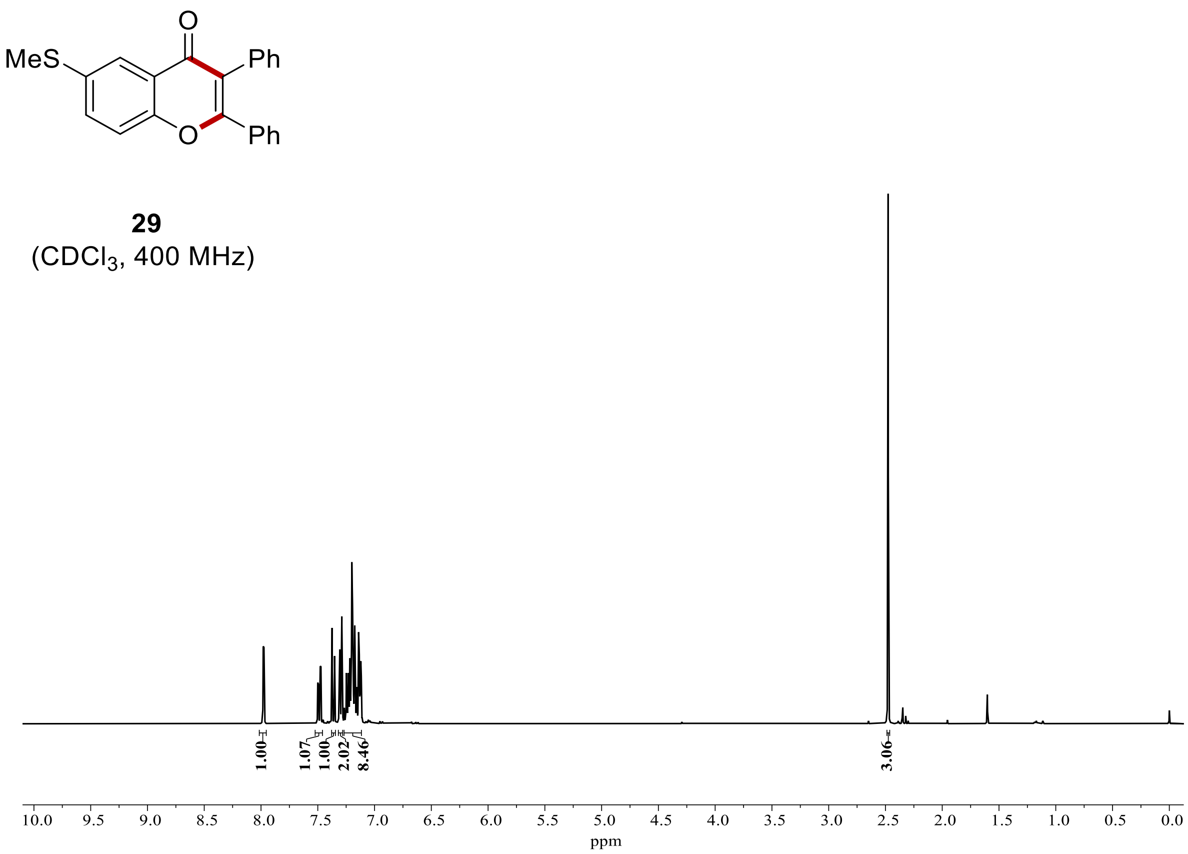

Supplementary Figure 78. ^{1}H-NMR spectrum of **29**.

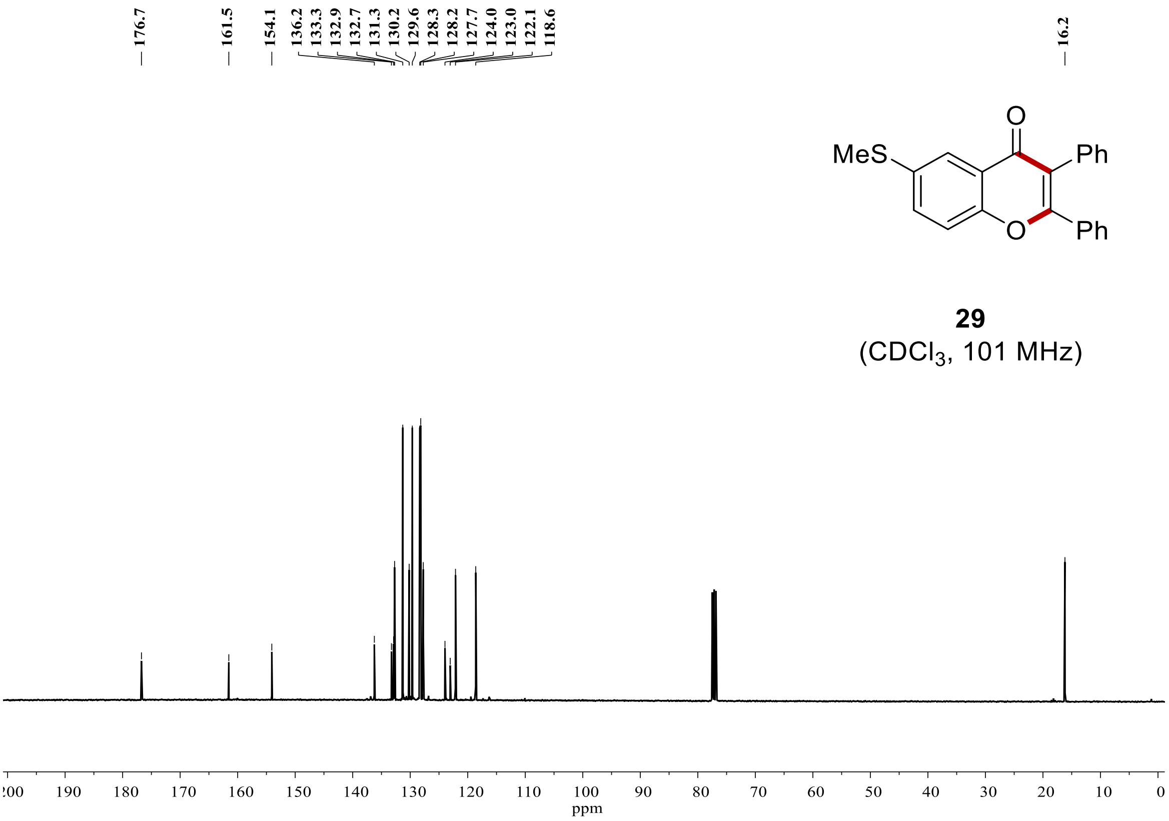

Supplementary Figure 79. ^{13}C-NMR spectrum of **29**.

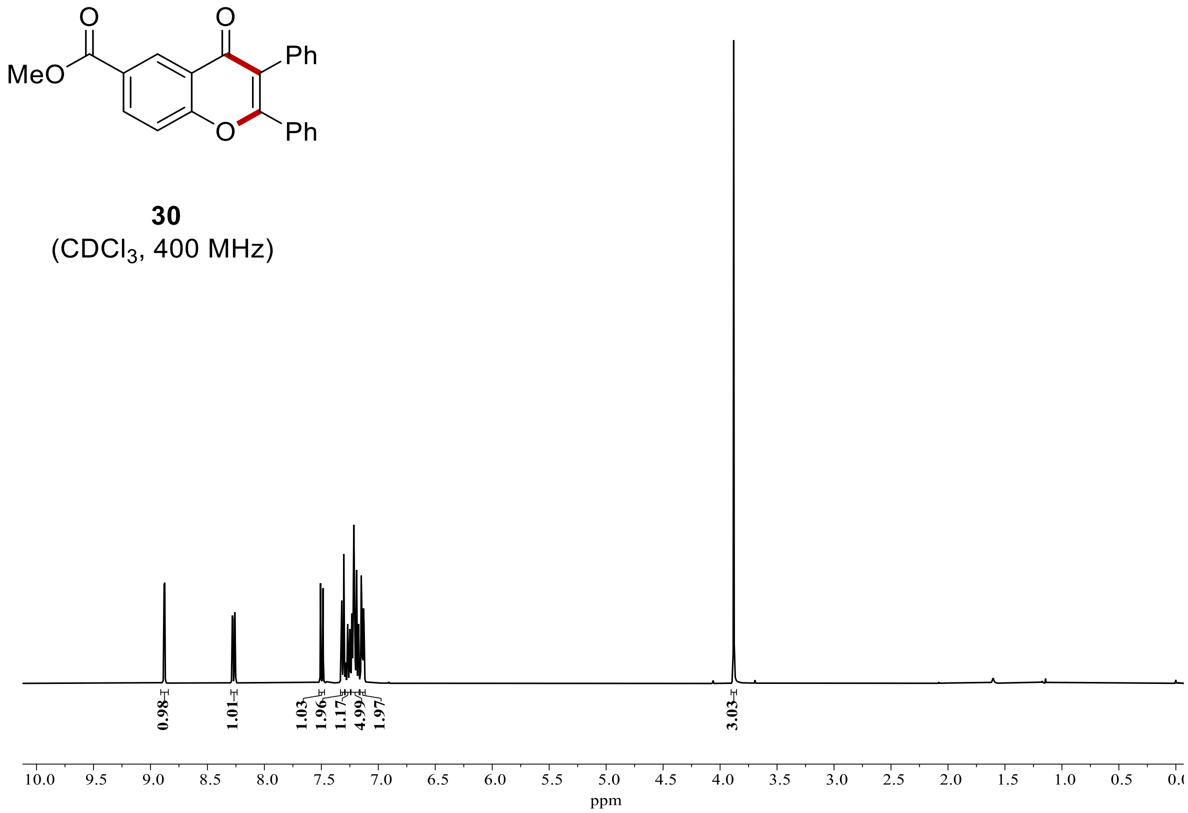

Supplementary Figure 80. ^{1}H-NMR spectrum of **30**.

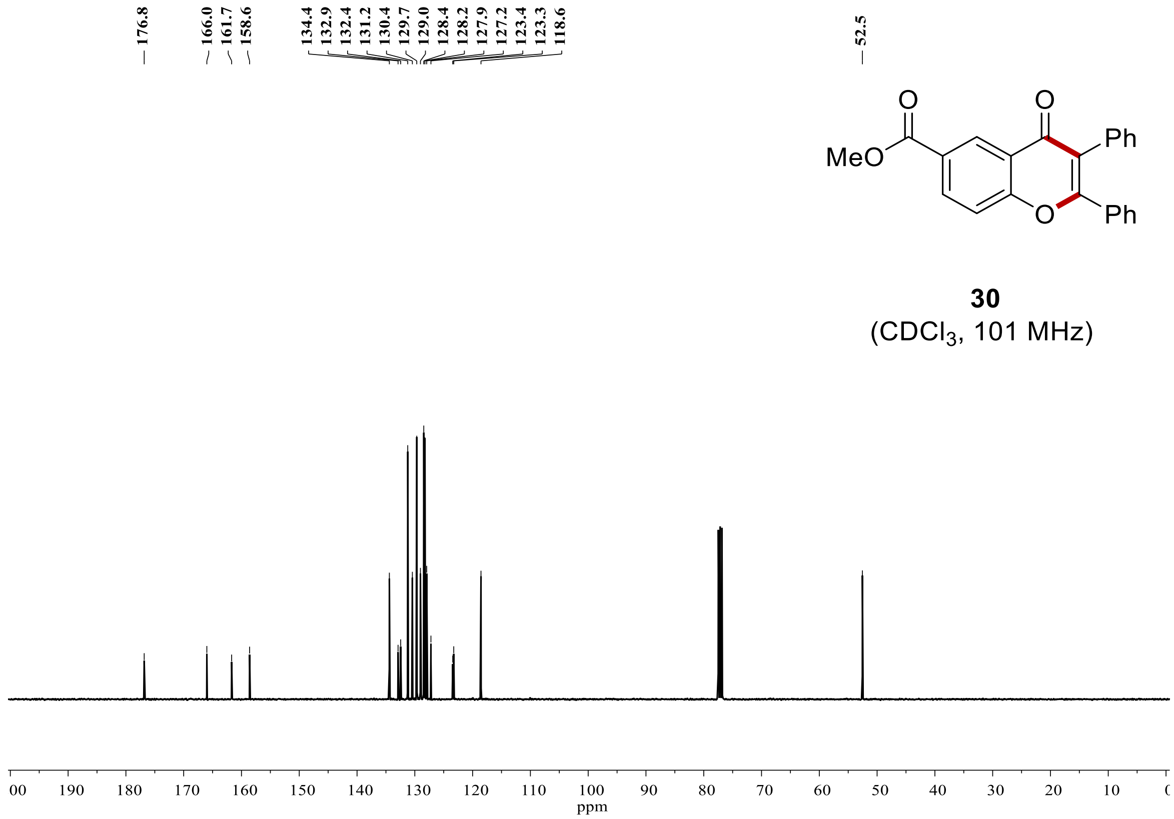

Supplementary Figure 81. ^{13}C-NMR spectrum of **30**.

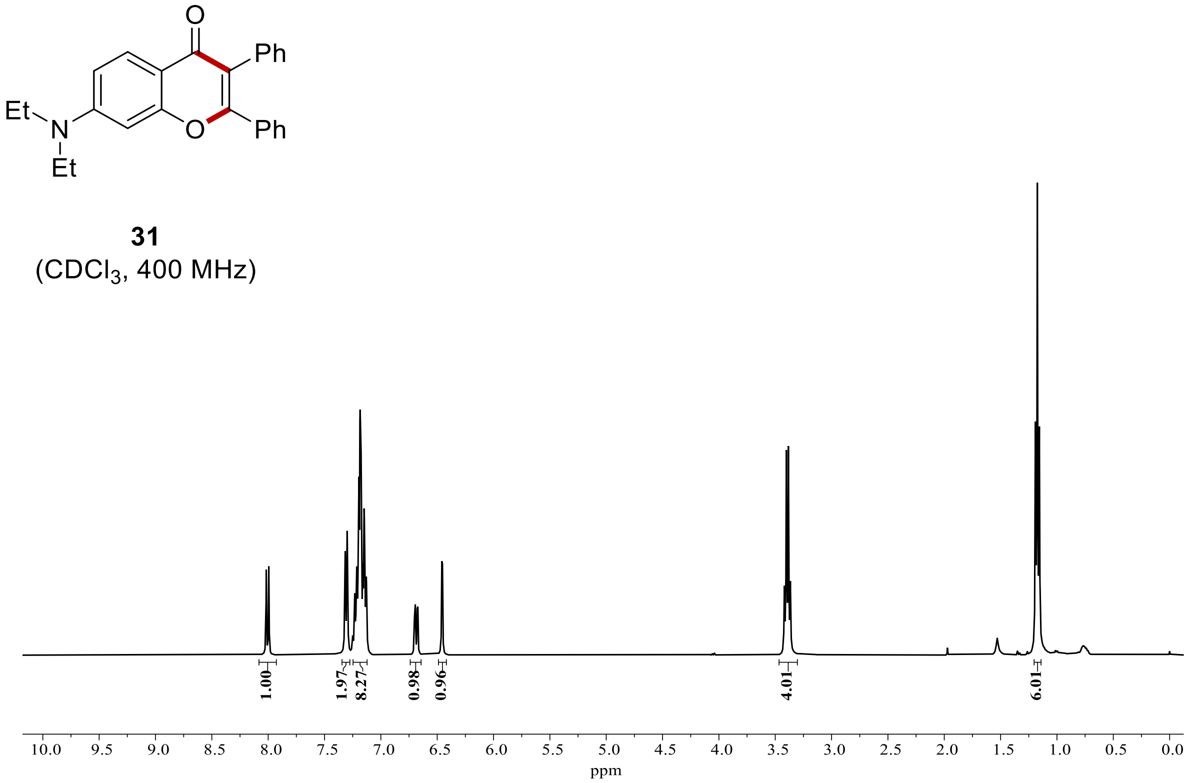

Supplementary Figure 82. ^{1}H-NMR spectrum of **31**.

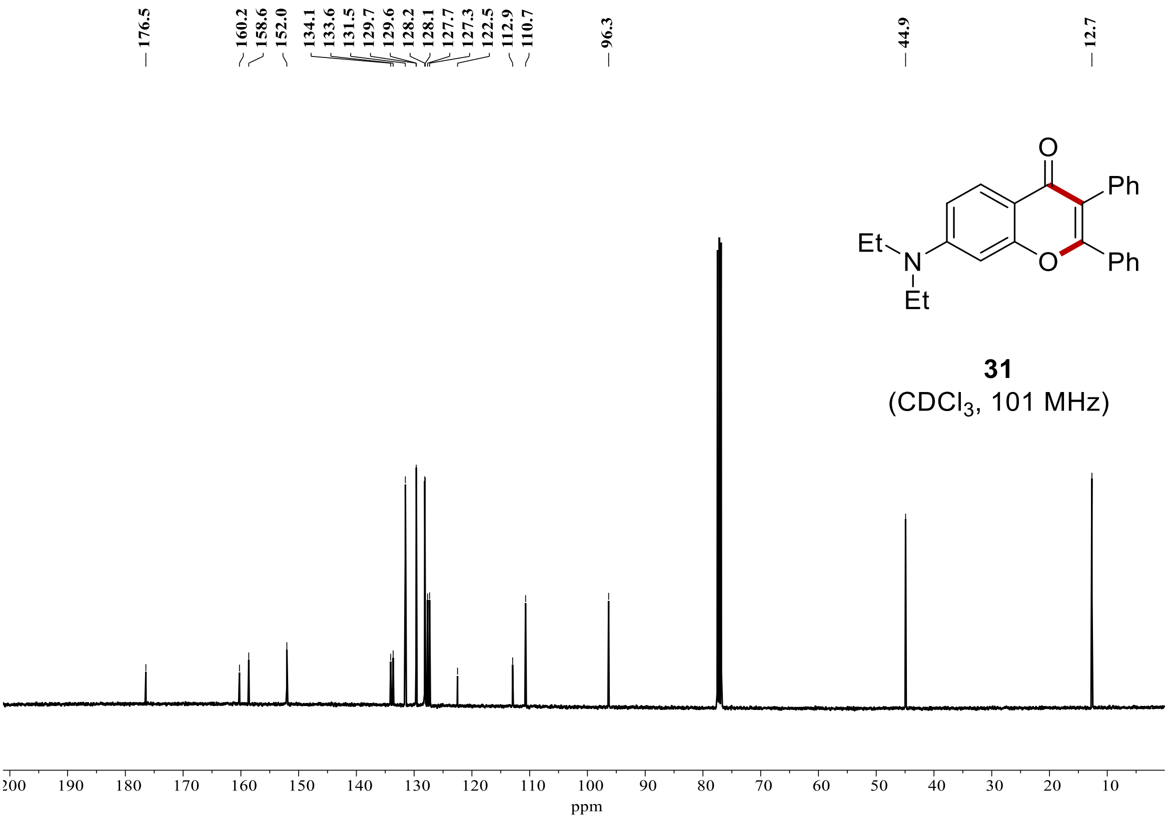

Supplementary Figure 83. ^{13}C-NMR spectrum of **31**.

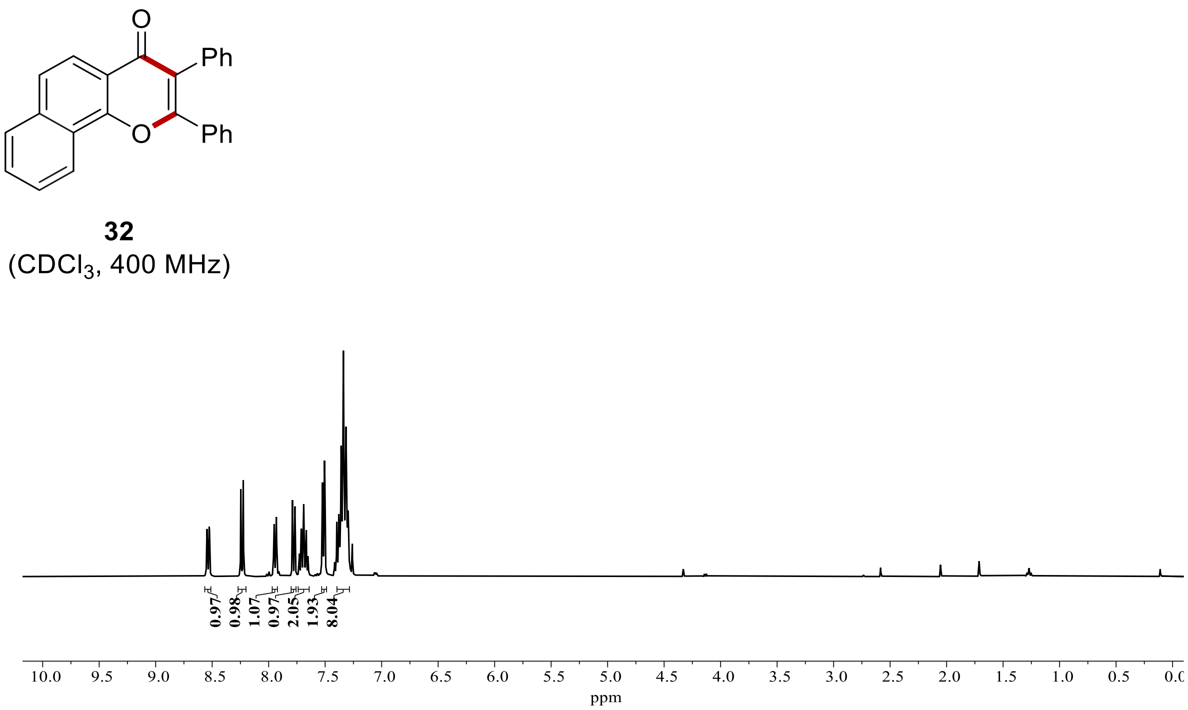

Supplementary Figure 84. ^{1}H-NMR spectrum of **32**.

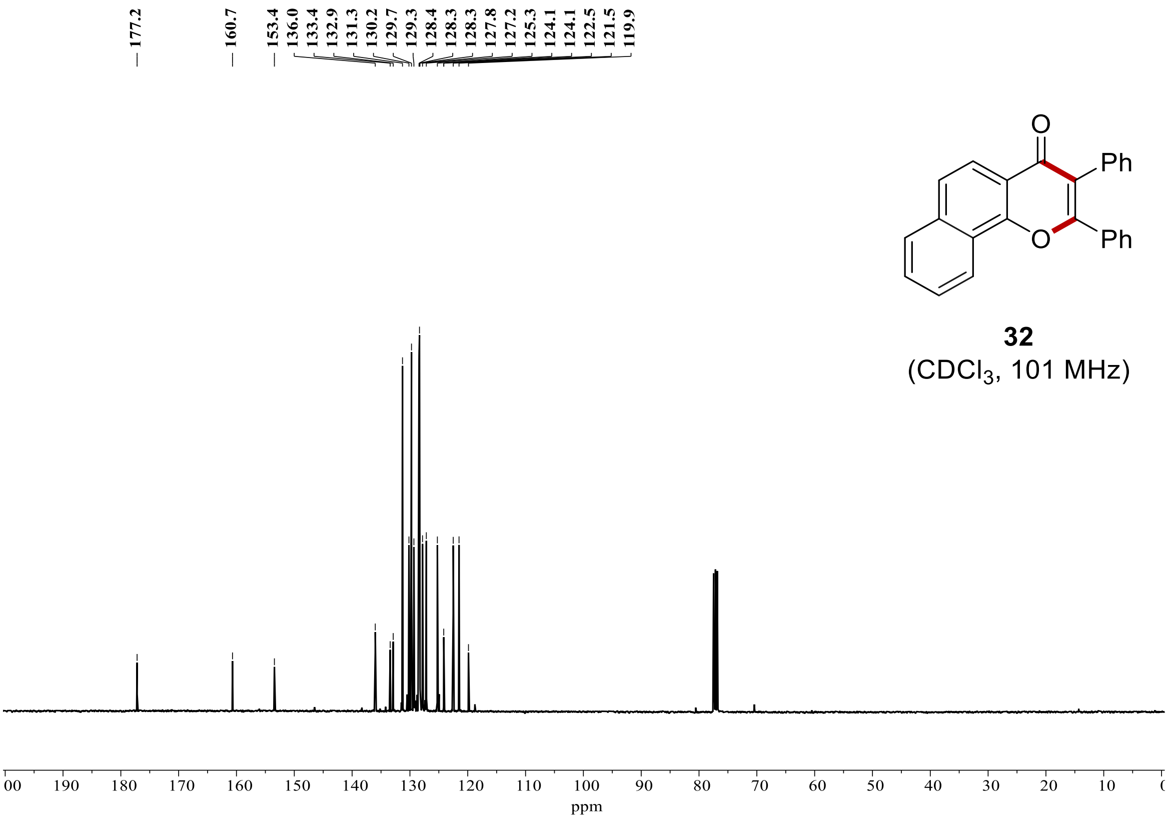

Supplementary Figure 85. ^{13}C-NMR spectrum of **32**.

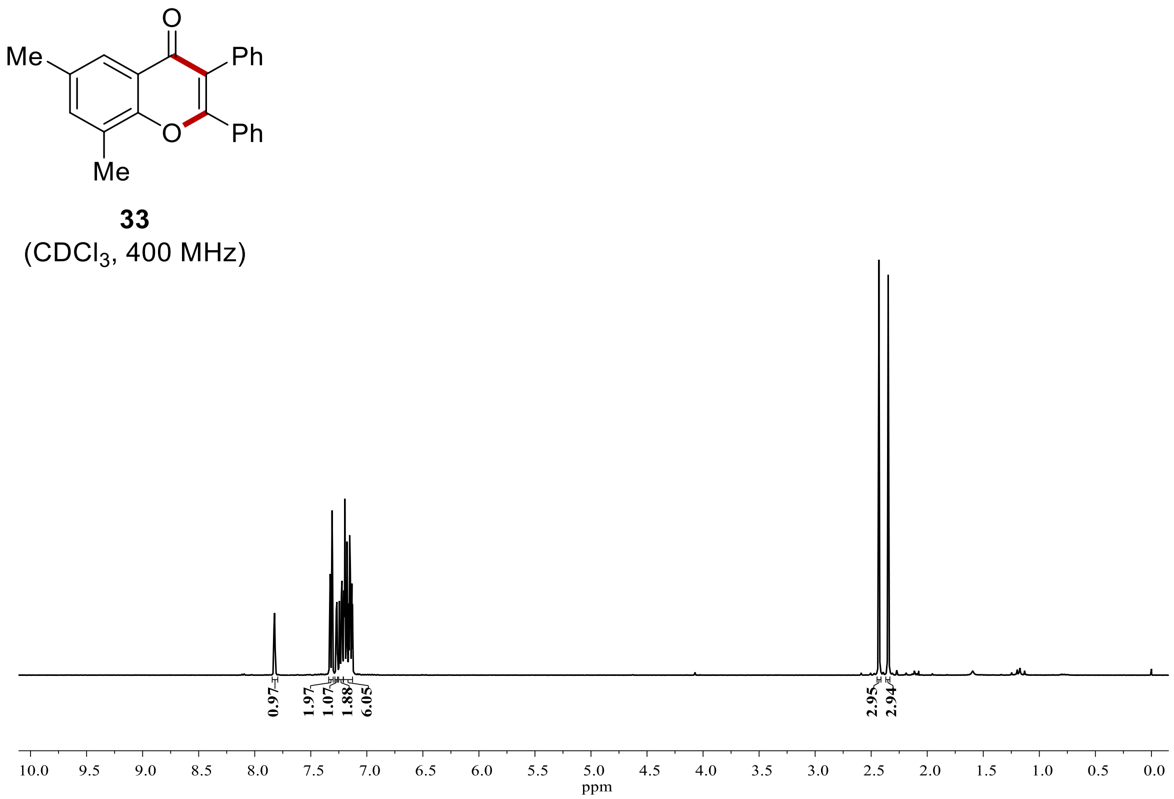

Supplementary Figure 86. ^{1}H-NMR spectrum of **33**.

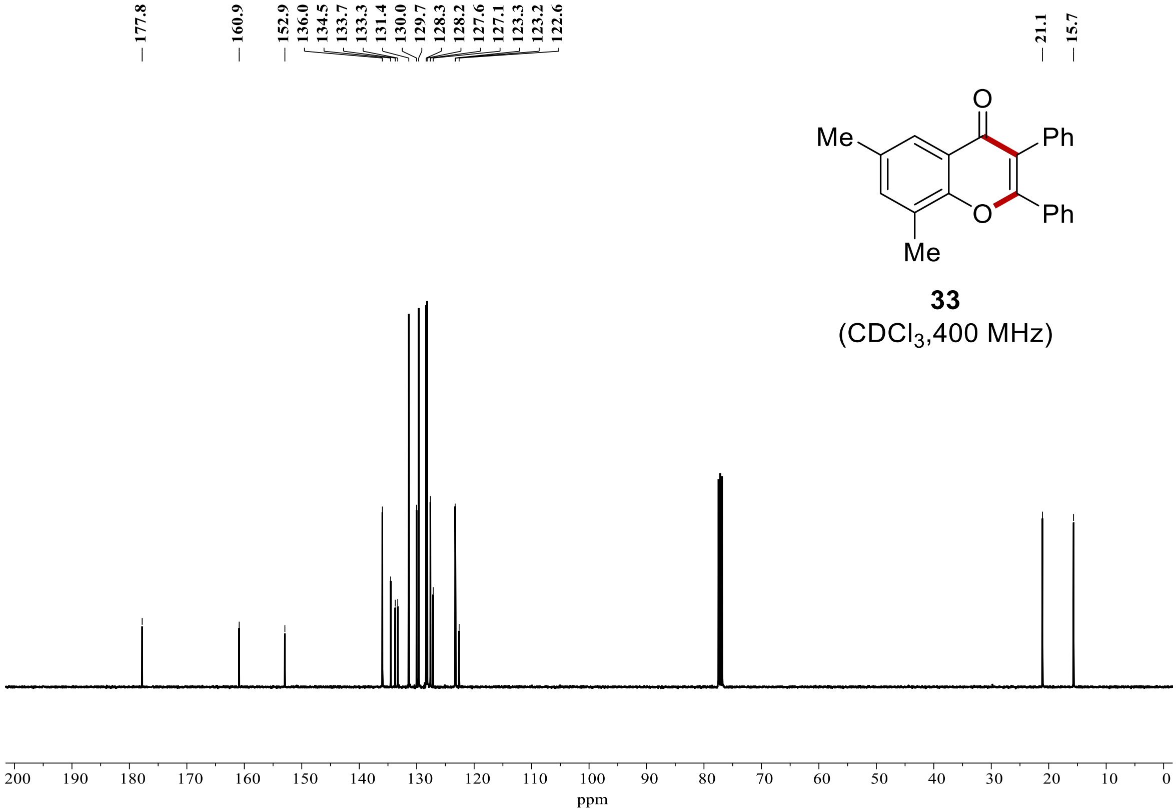

Supplementary Figure 87. ^{13}C-NMR spectrum of **33**.

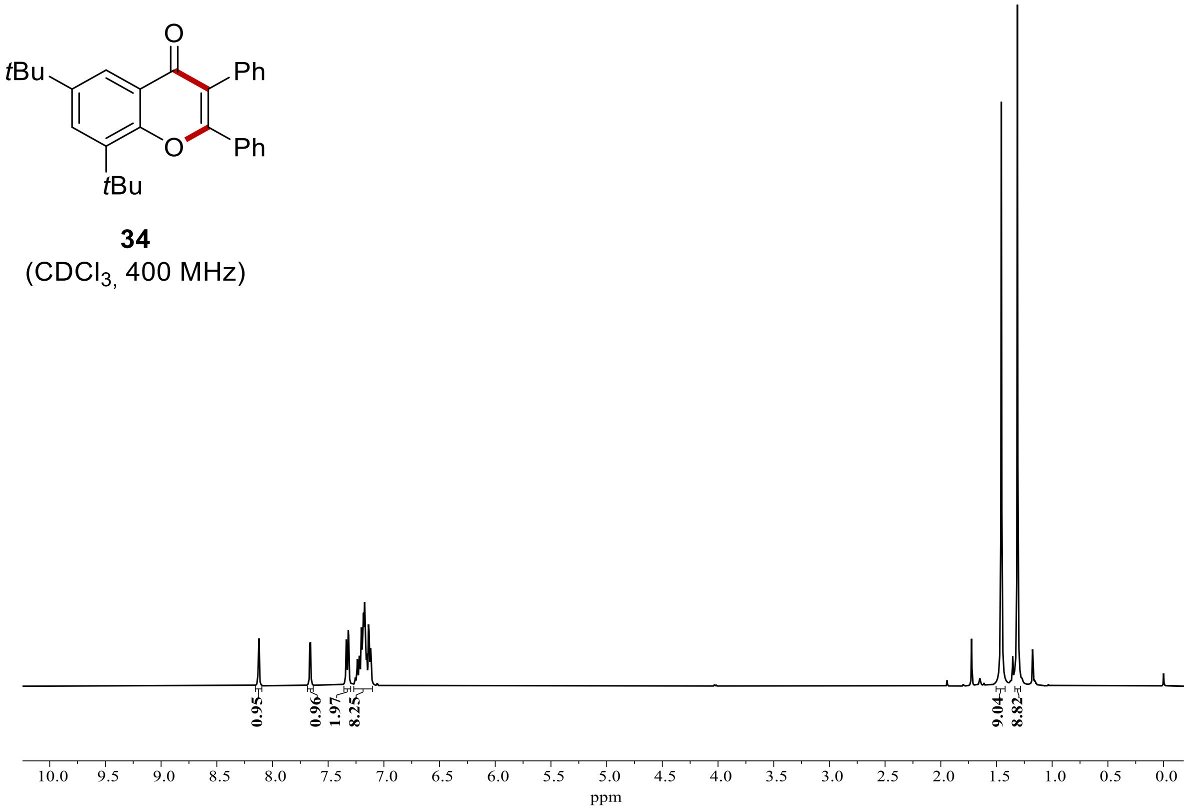

Supplementary Figure 88. ^{1}H-NMR spectrum of **34**.

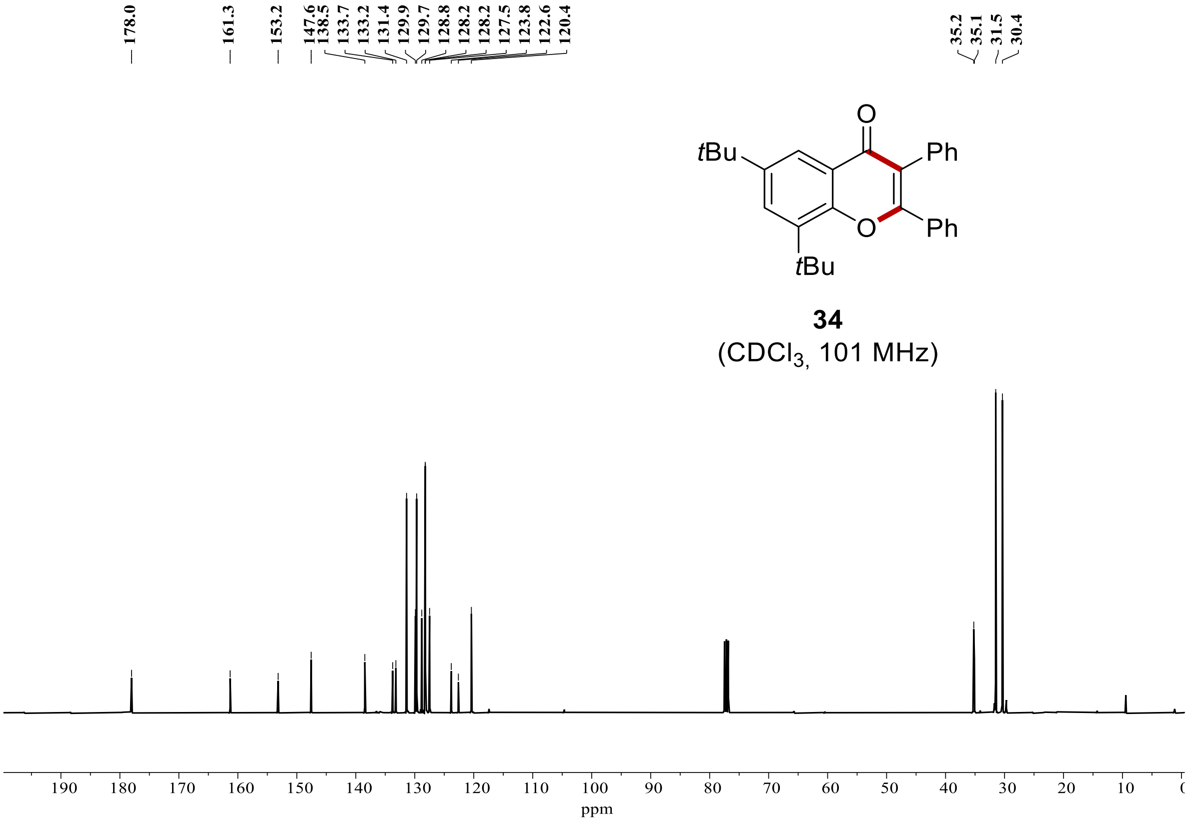

Supplementary Figure 89. ^{13}C-NMR spectrum of **34**.

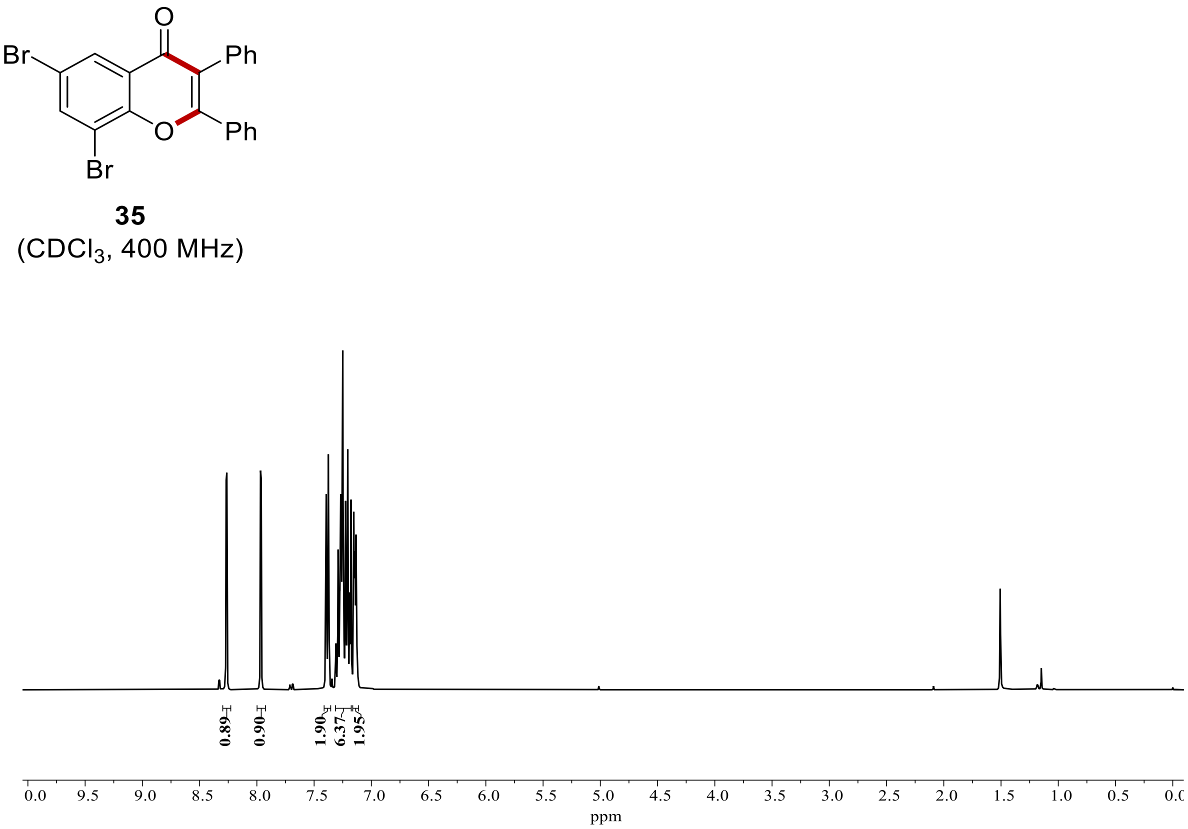

Supplementary Figure 90. ^{1}H-NMR spectrum of **35**.

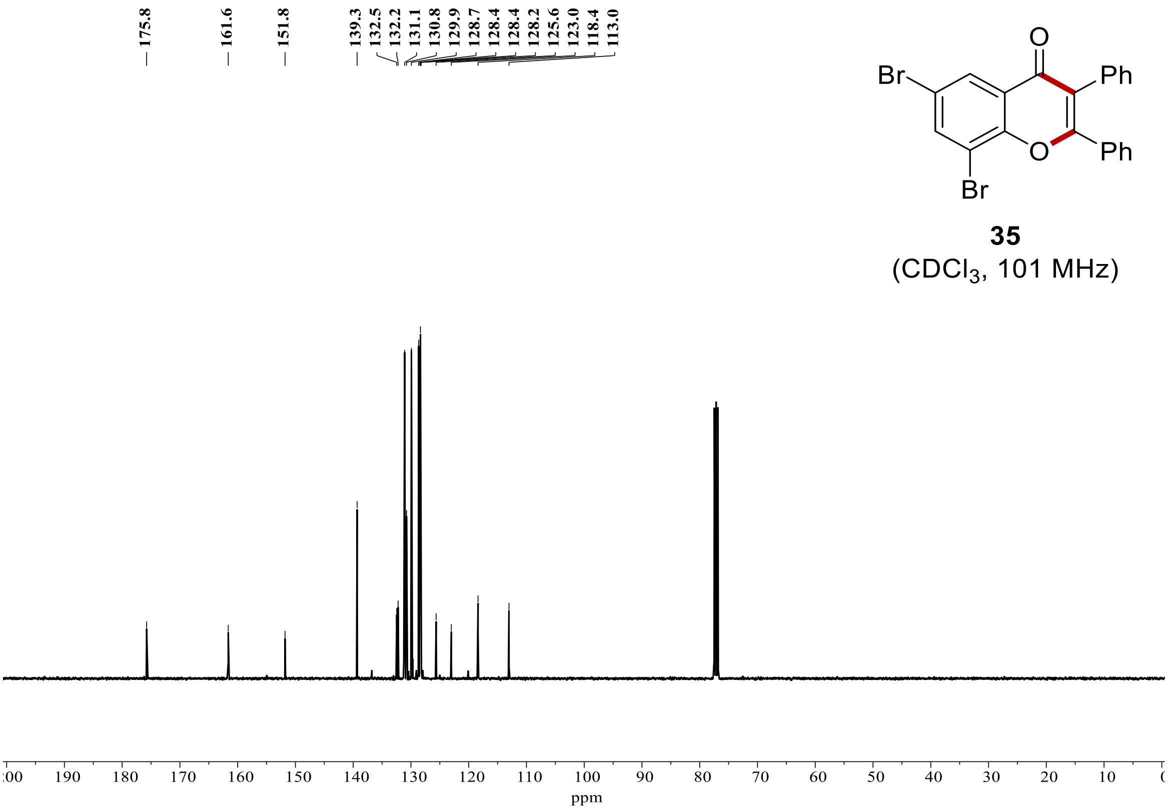

Supplementary Figure 91. ^{13}C-NMR spectrum of **35**.

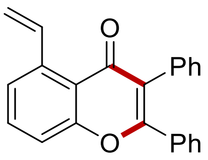

36
($CDCl_3$, 400 MHz)

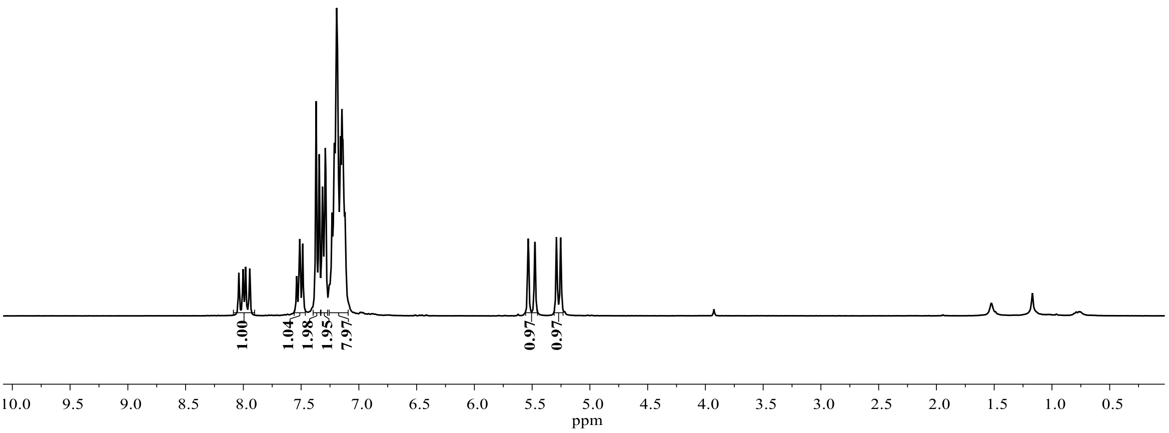

Supplementary Figure 92. ^{1}H-NMR spectrum of **36**.

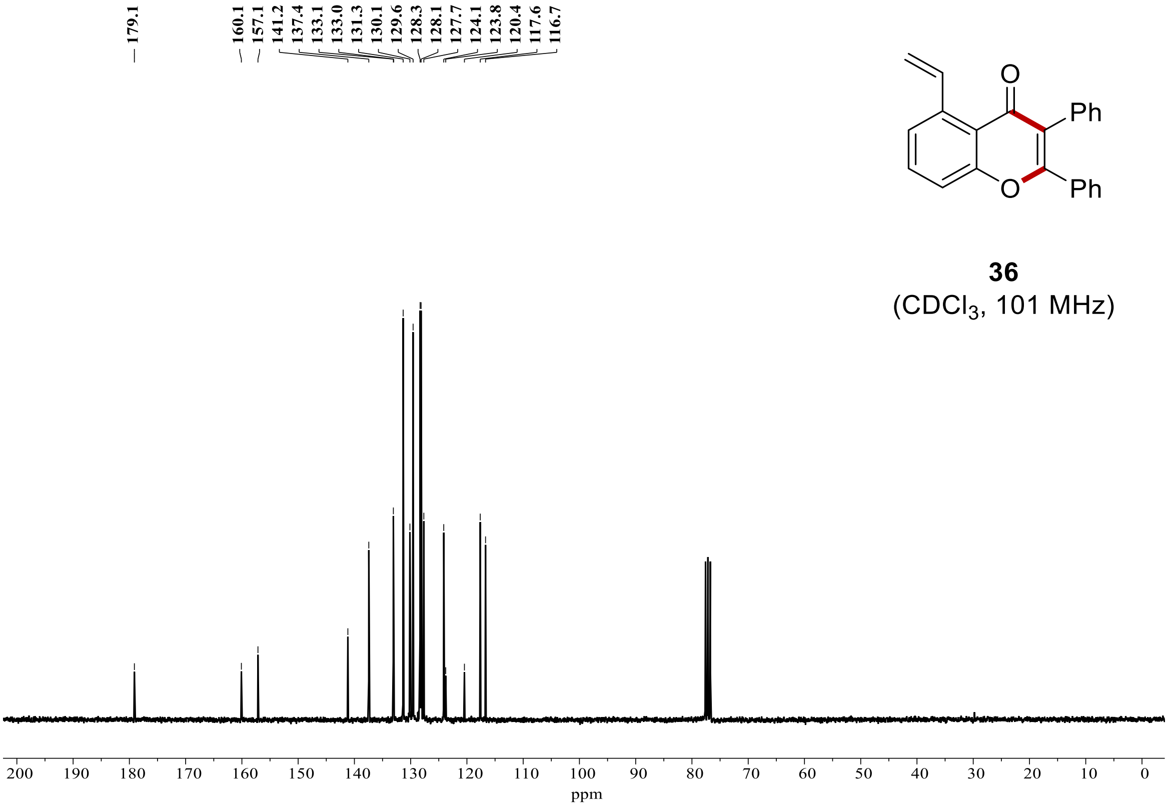

36
($CDCl_3$, 101 MHz)

Supplementary Figure 93. ^{13}C-NMR spectrum of **36**.

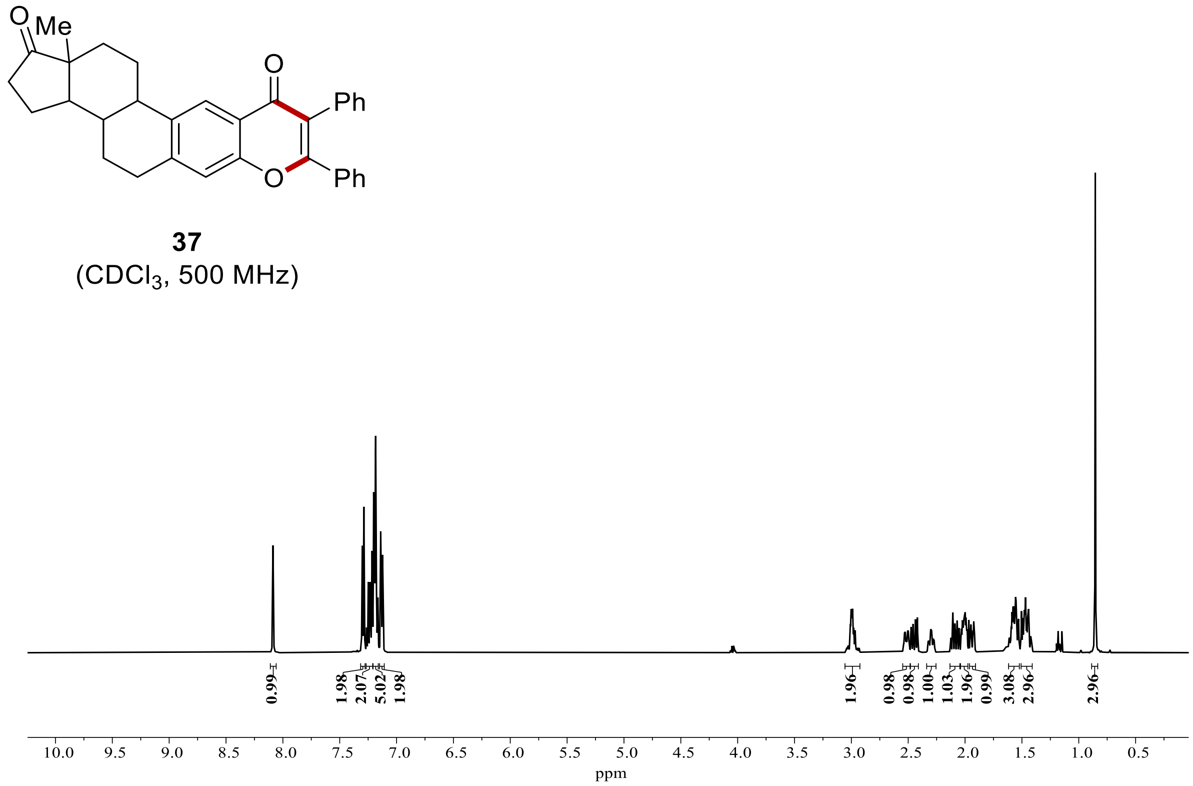

Supplementary Figure 94. ^{1}H-NMR spectrum of **37**.

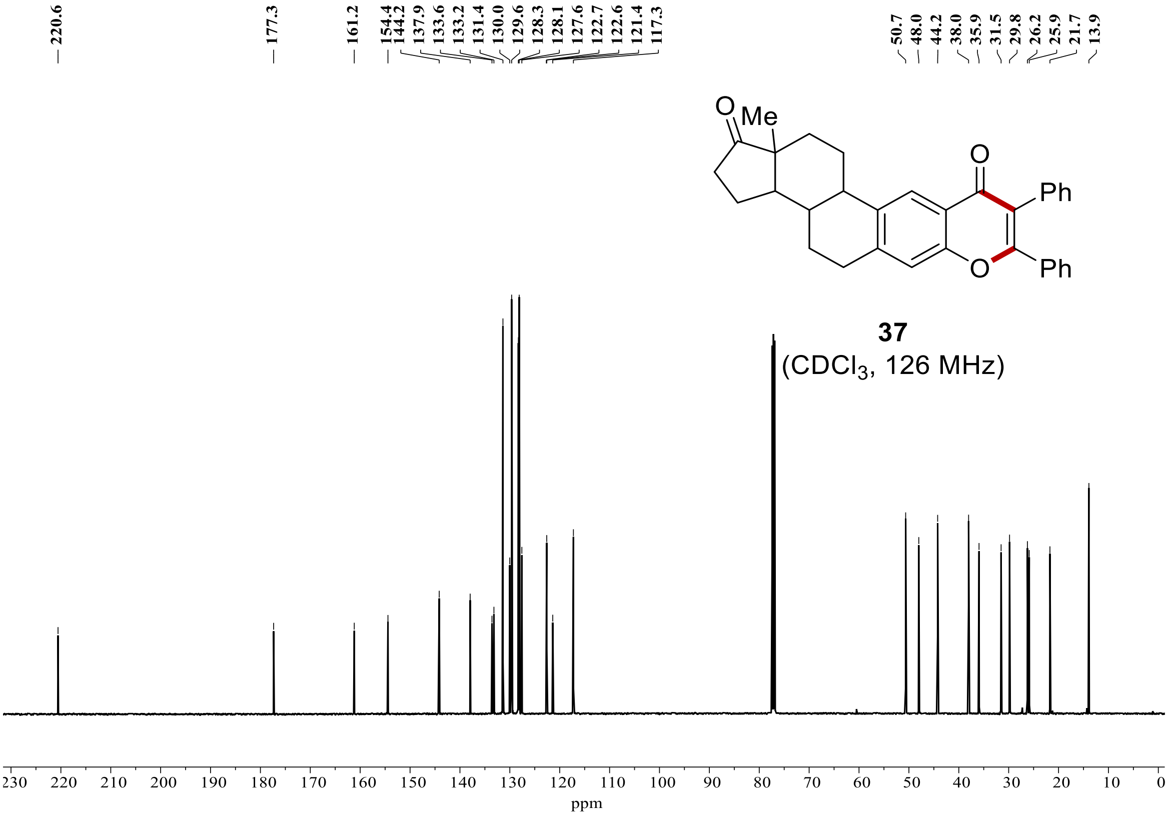

Supplementary Figure 95. ^{13}C-NMR spectrum of **37**.

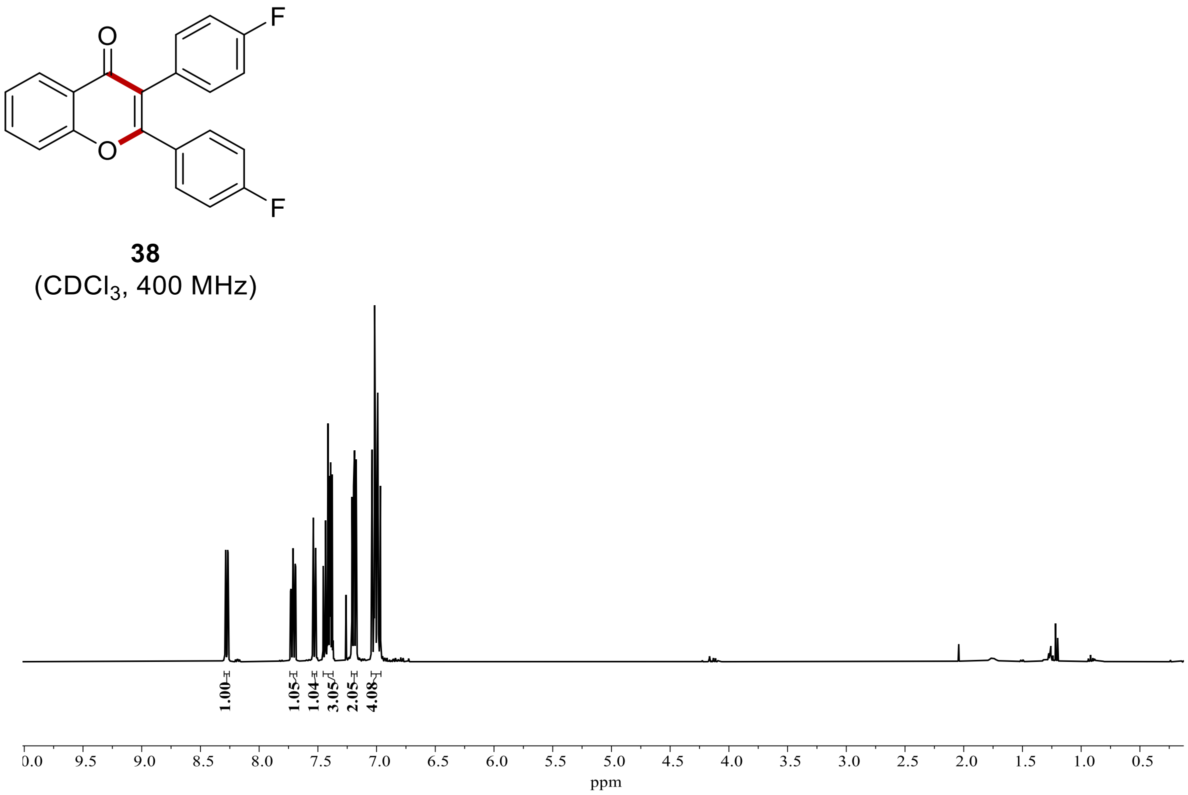

Supplementary Figure 96. ^{1}H-NMR spectrum of **38**.

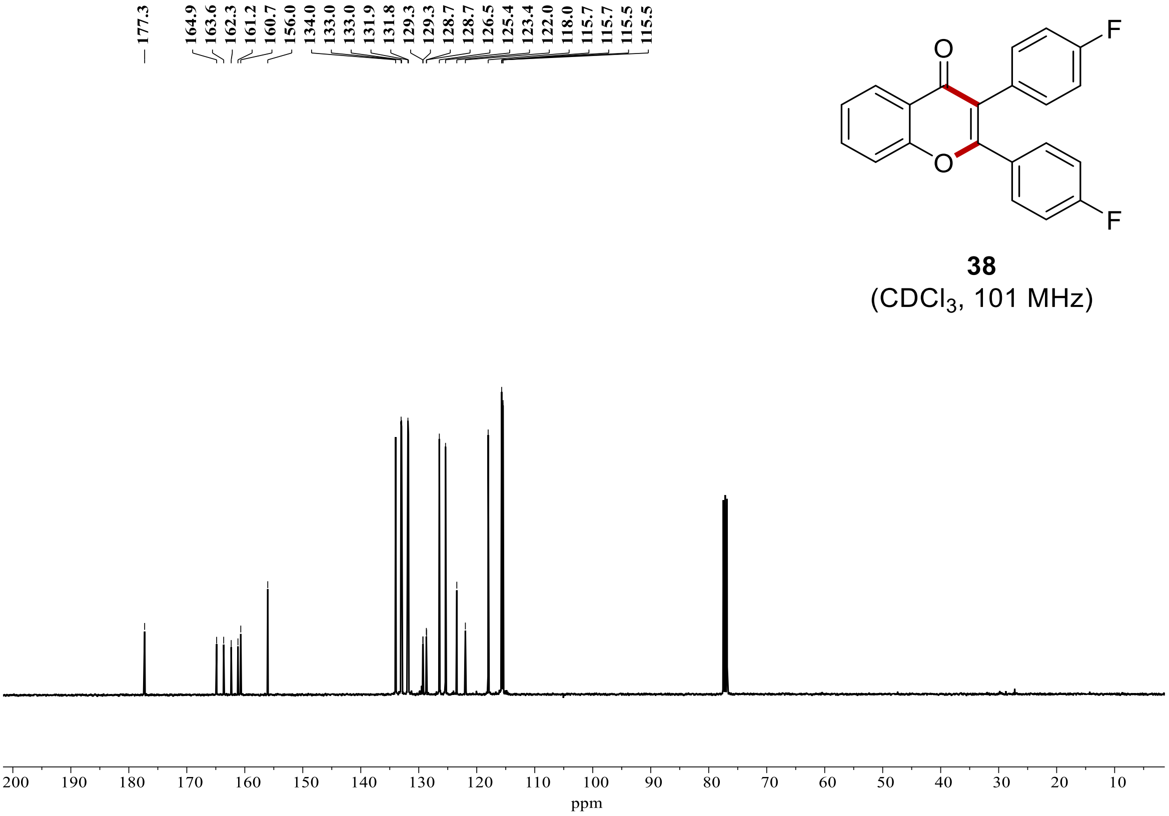

Supplementary Figure 97. ^{13}C-NMR spectrum of **38**.

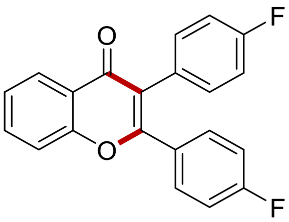

38
($CDCl_3$, 376 MHz)

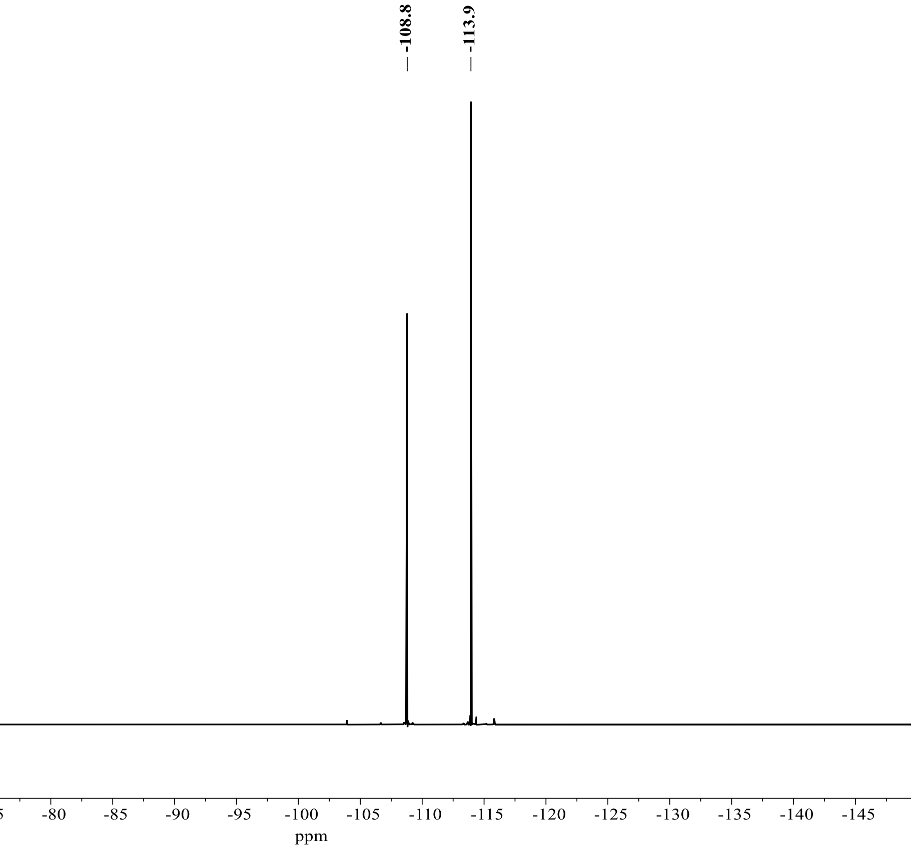

Supplementary Figure 98. ^{19}F-NMR spectrum of **38**.

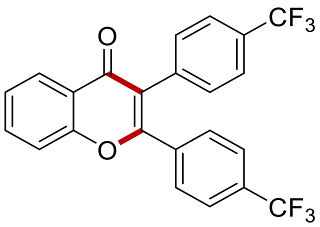

39
($CDCl_3$, 400 MHz)

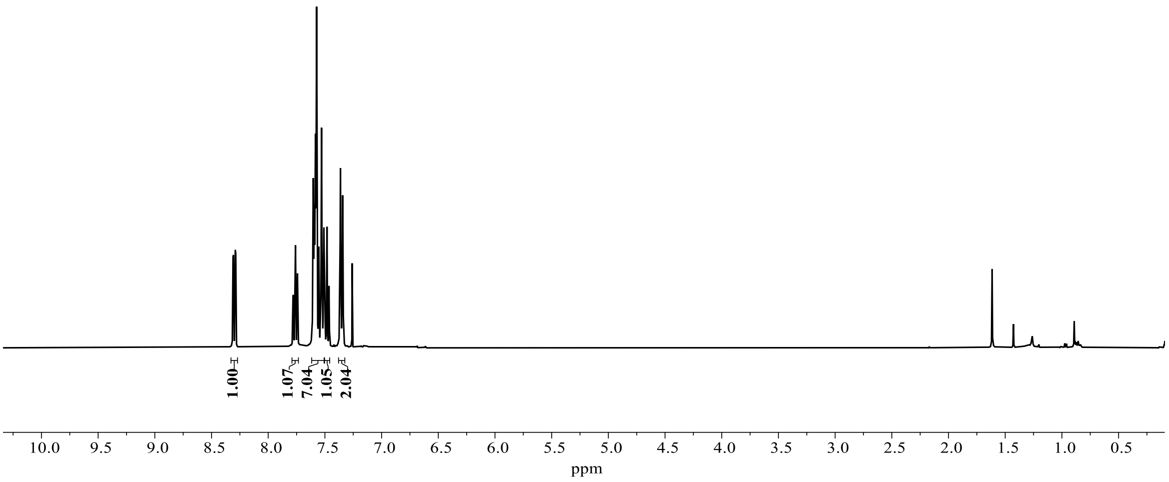

Supplementary Figure 99. ^{1}H-NMR spectrum of **39**.

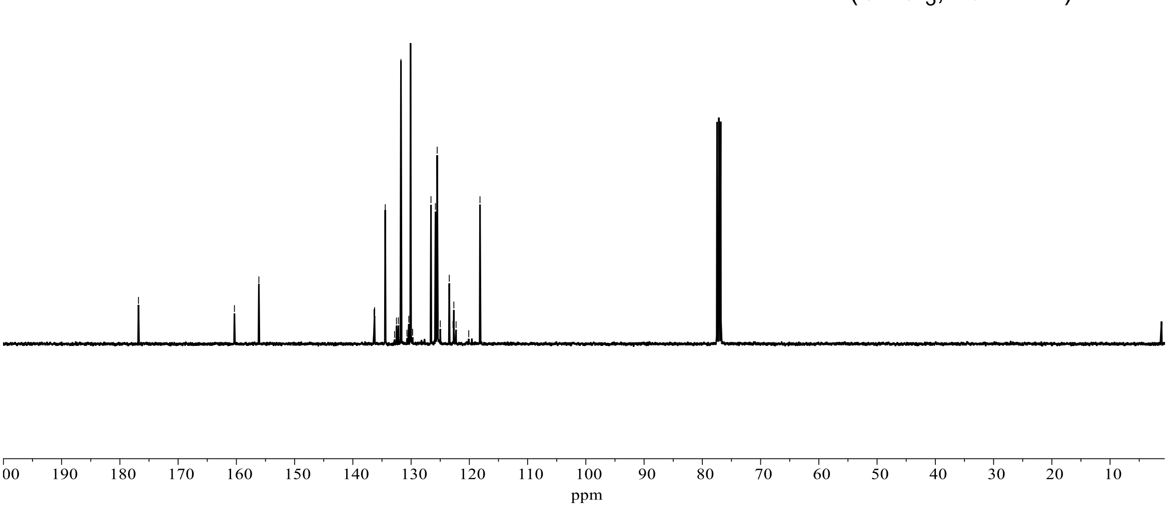

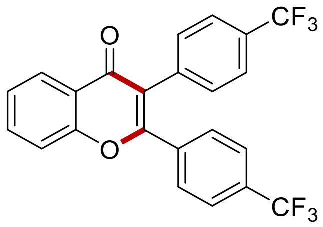

39
($CDCl_3$, 101 MHz)

Supplementary Figure 100. ^{13}C-NMR spectrum of **39**.

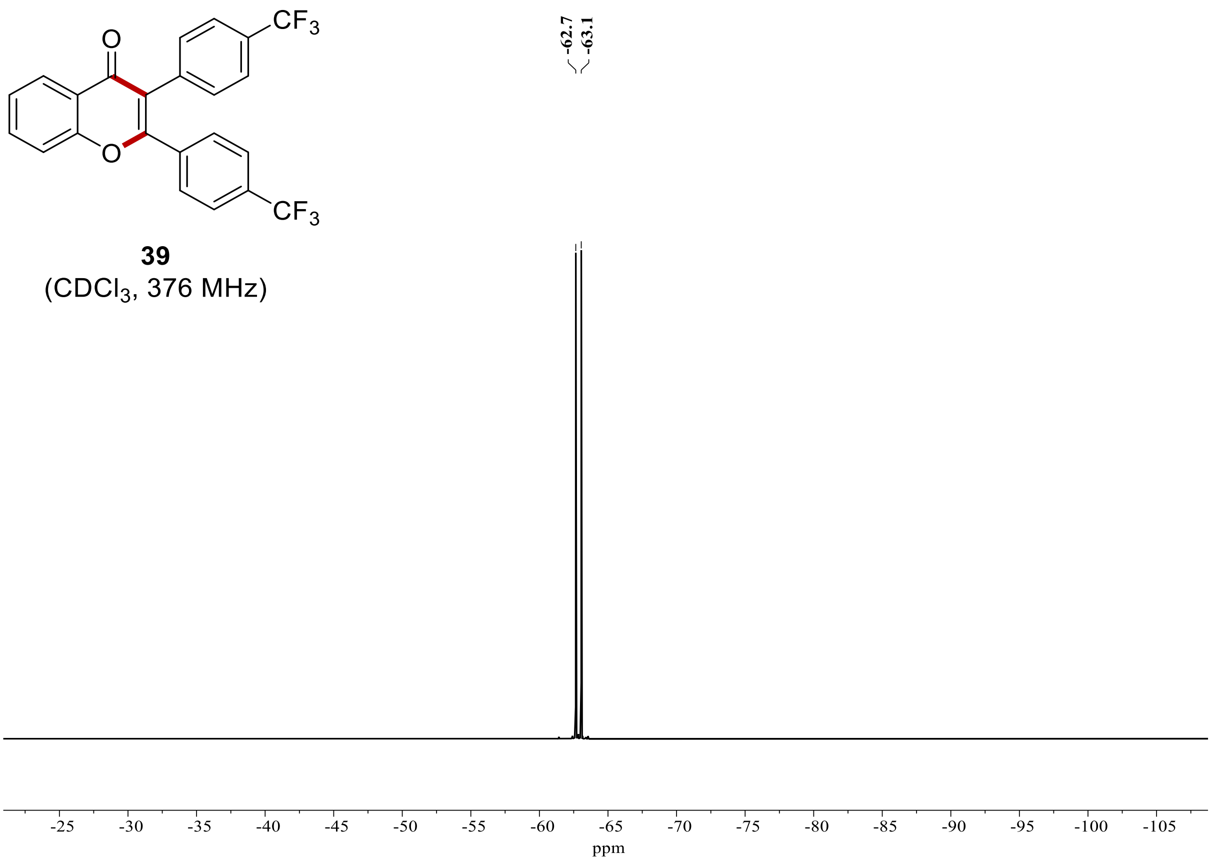

Supplementary Figure 101. ^{19}F-NMR spectrum of **39**.

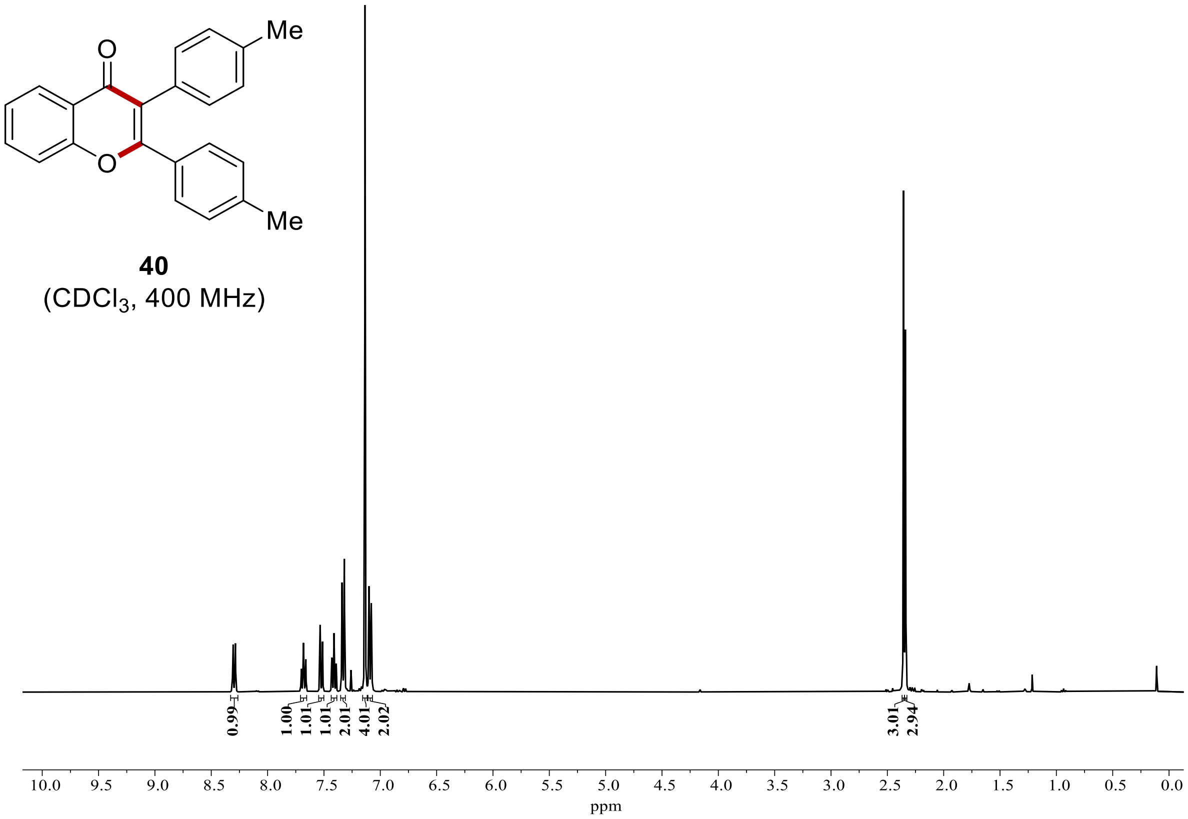

Supplementary Figure 102. ^{1}H-NMR spectrum of **40**.

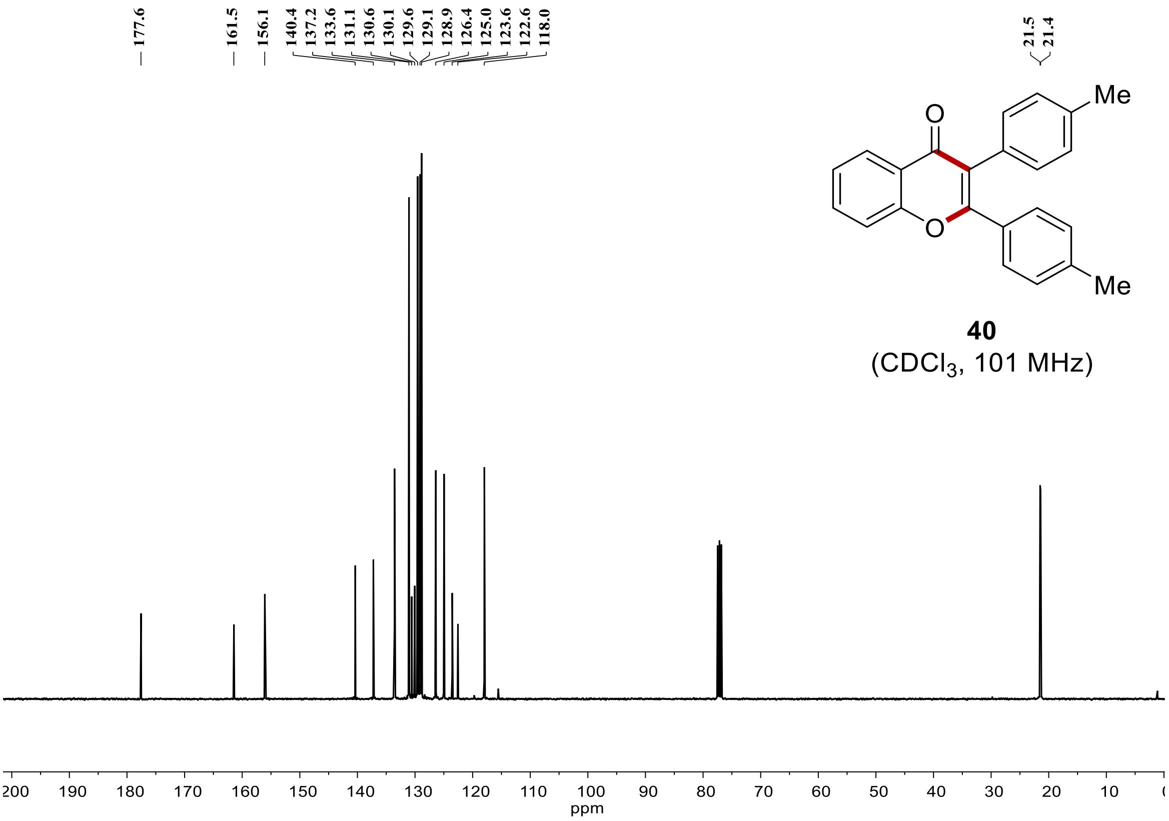

Supplementary Figure 103. ^{13}C-NMR spectrum of **40**.

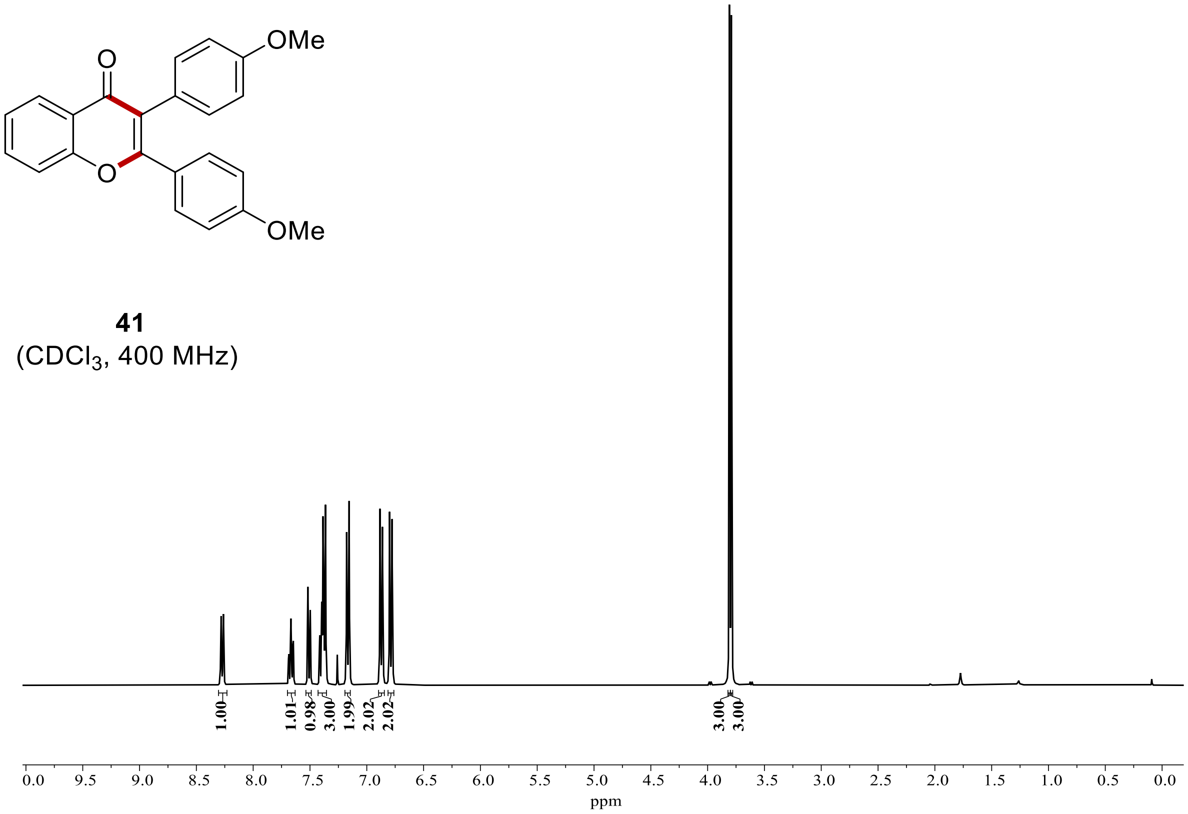

Supplementary Figure 104. ^{1}H-NMR spectrum of **41**.

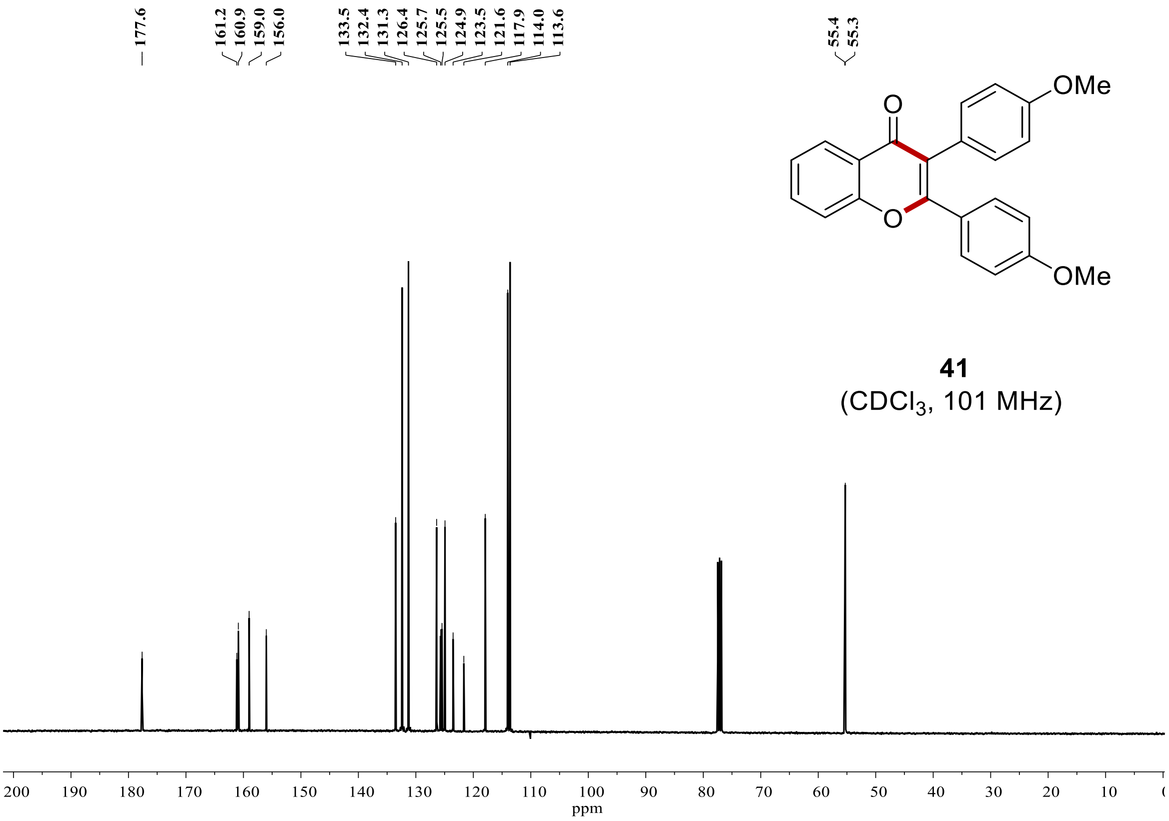

Supplementary Figure 105. ^{13}C-NMR spectrum of **41**.

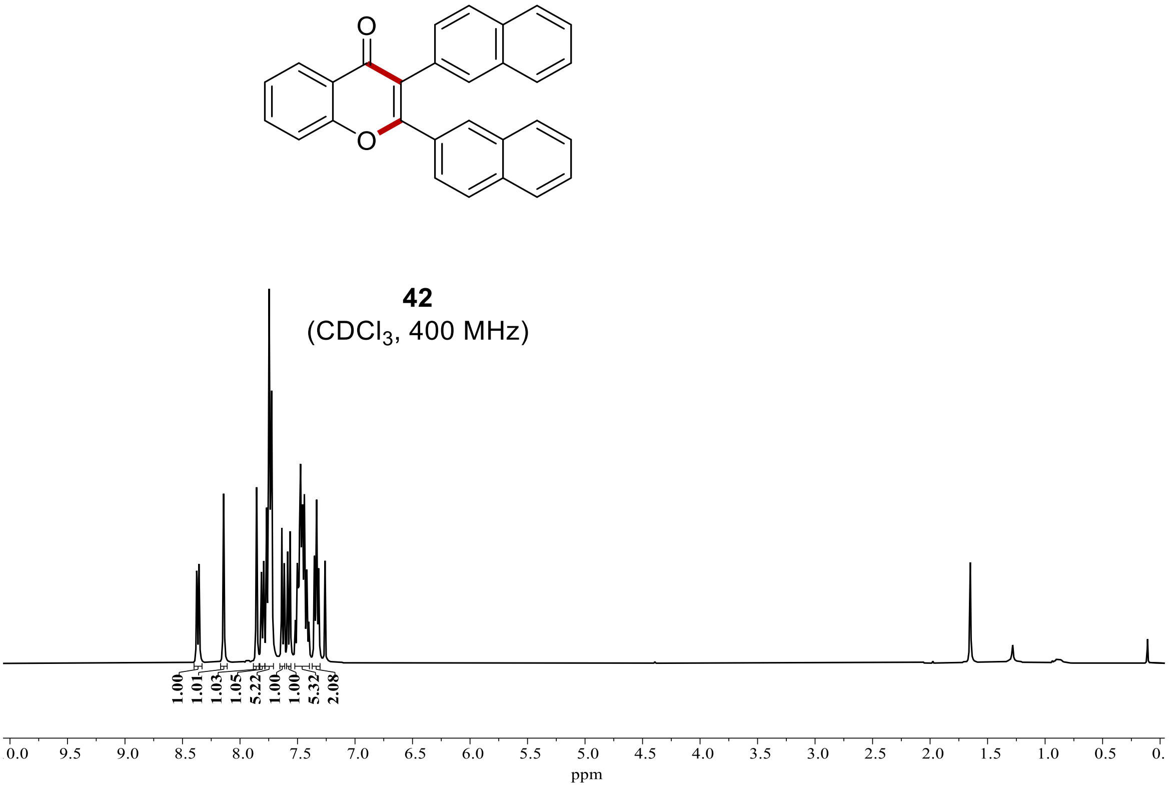

Supplementary Figure 106. ^{1}H-NMR spectrum of **42**.

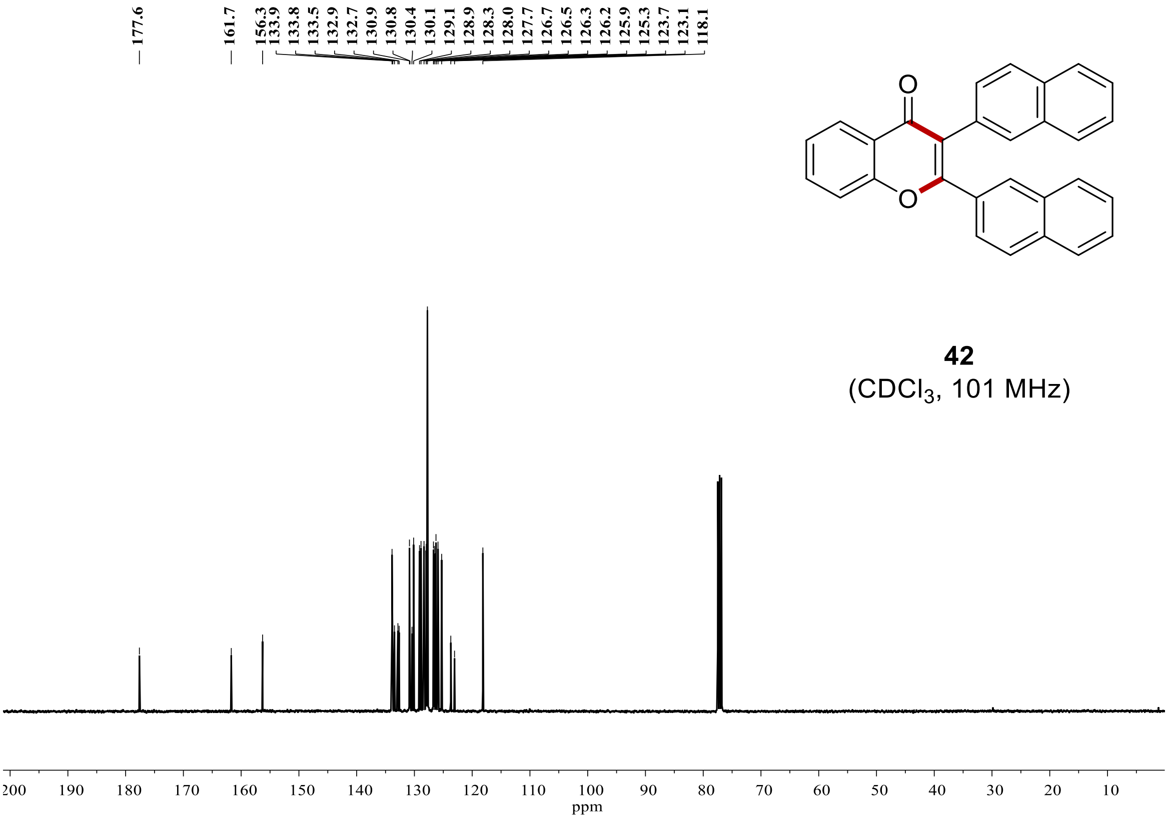

Supplementary Figure 107. ^{13}C-NMR spectrum of **42**.

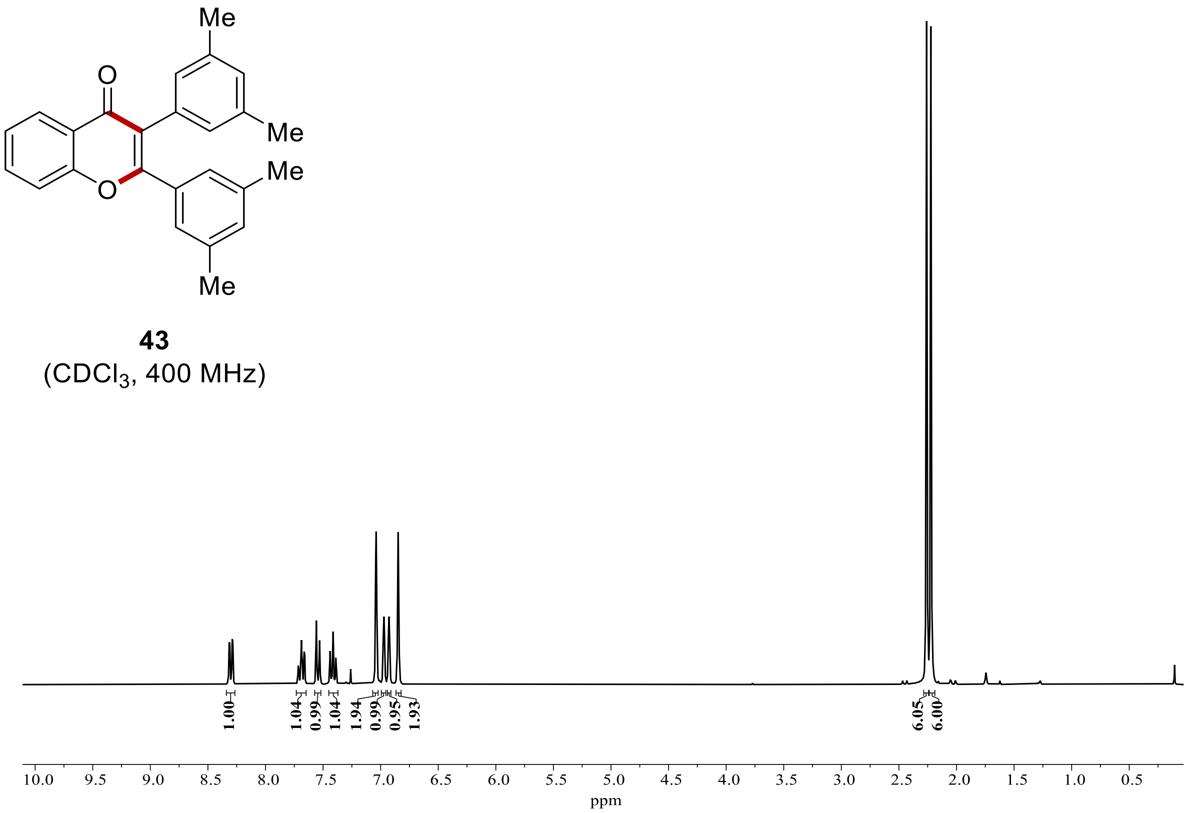

Supplementary Figure 108. ^{1}H-NMR spectrum of **43**.

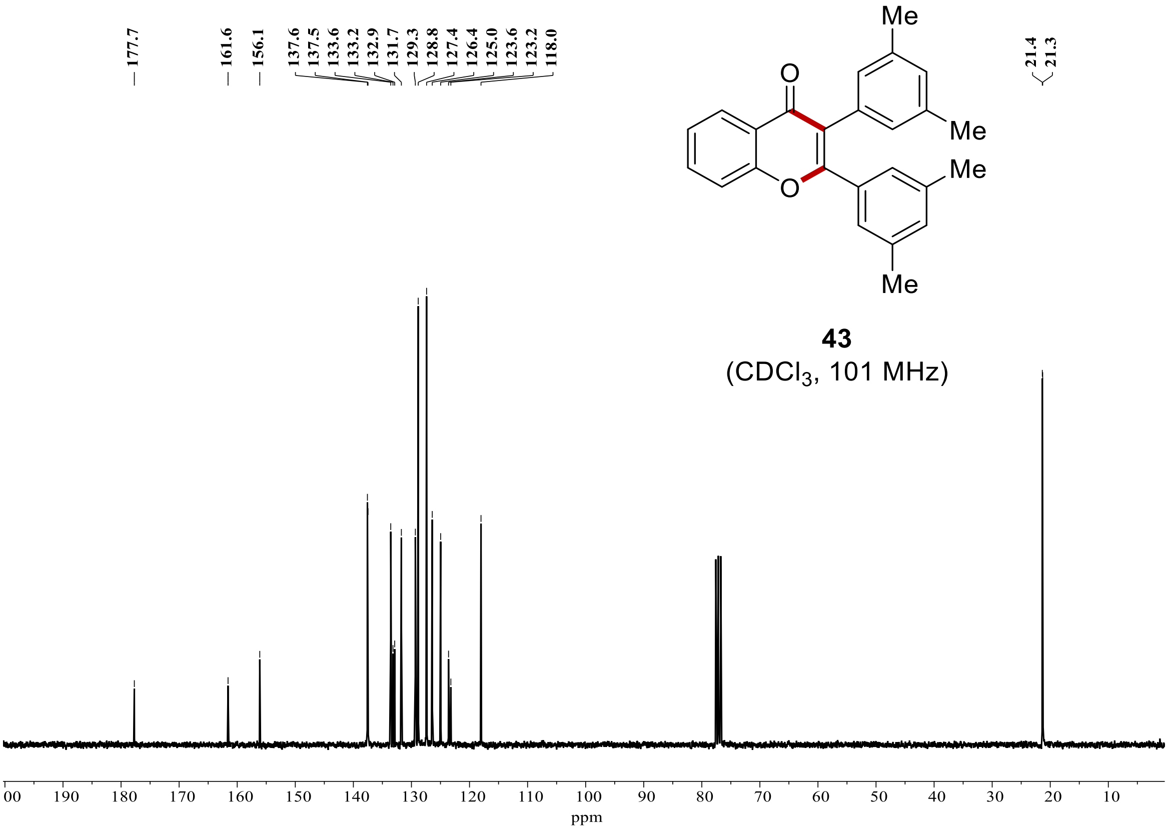

Supplementary Figure 109. ^{13}C-NMR spectrum of **43**.

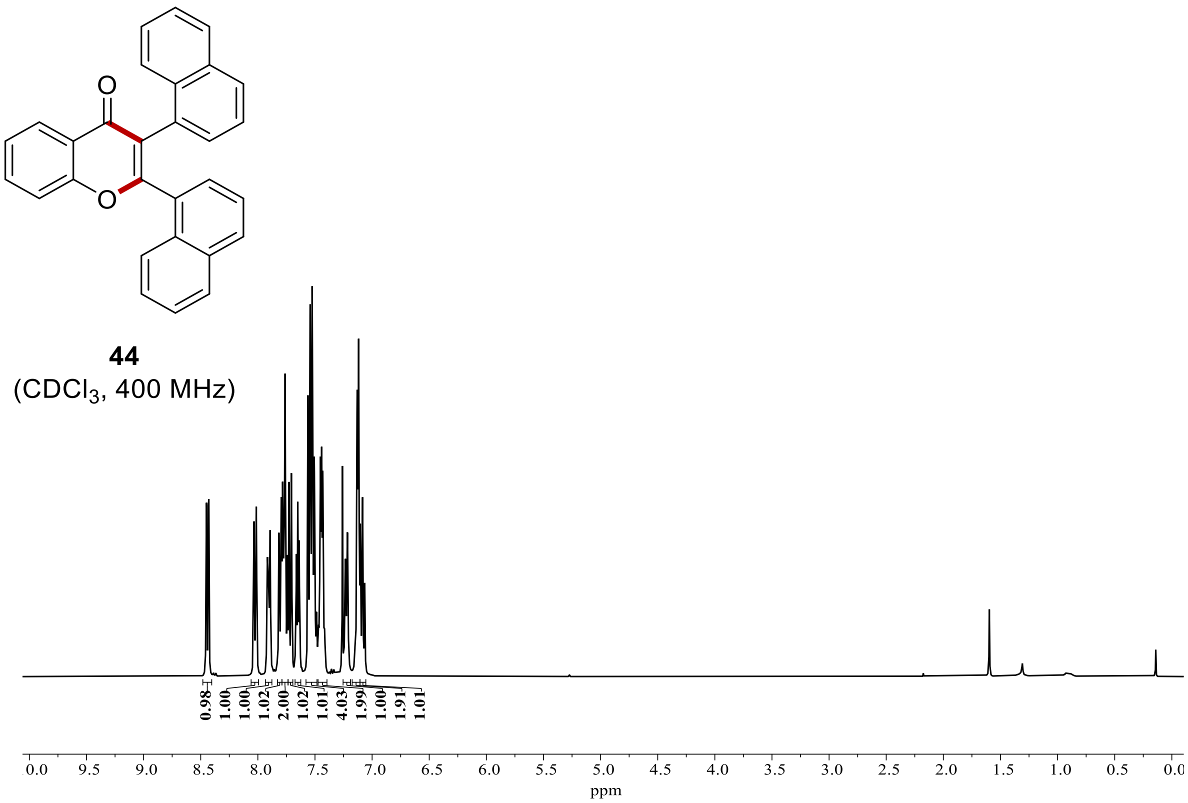

Supplementary Figure 110. ^{1}H-NMR spectrum of **44**.

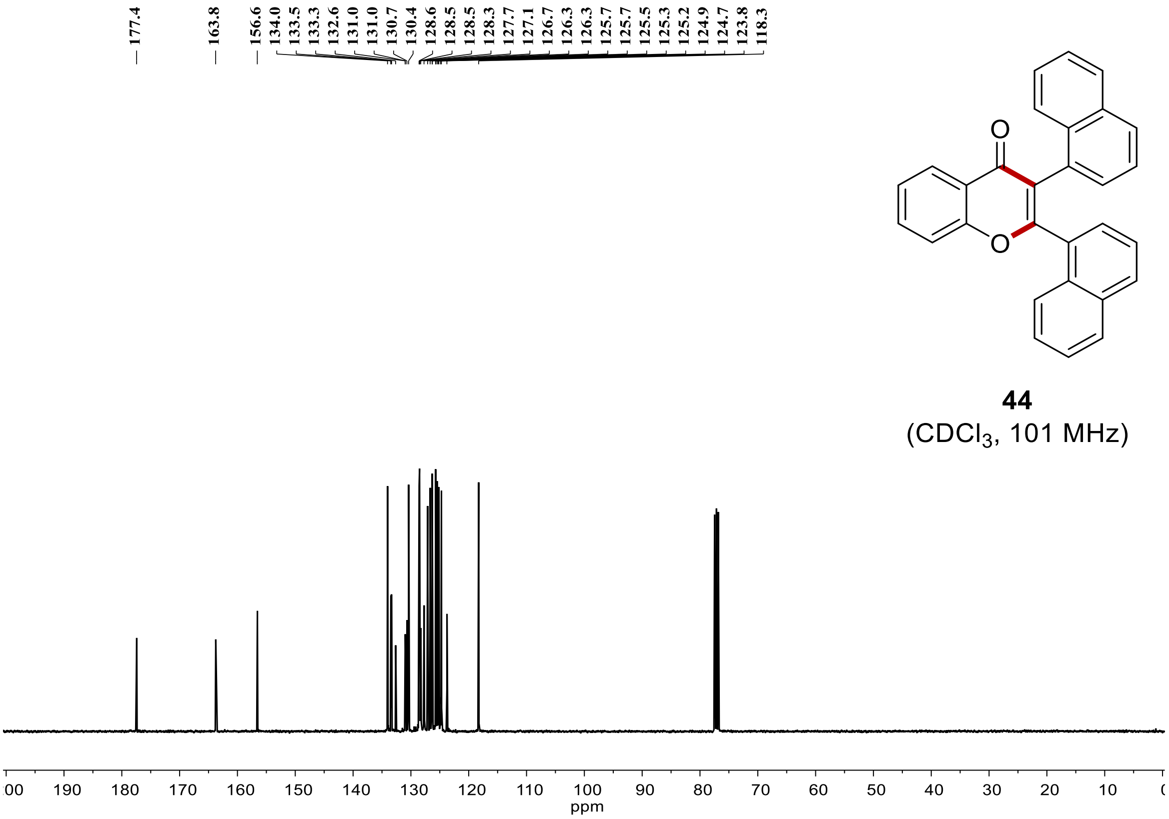

Supplementary Figure 111. ^{13}C-NMR spectrum of **44**.

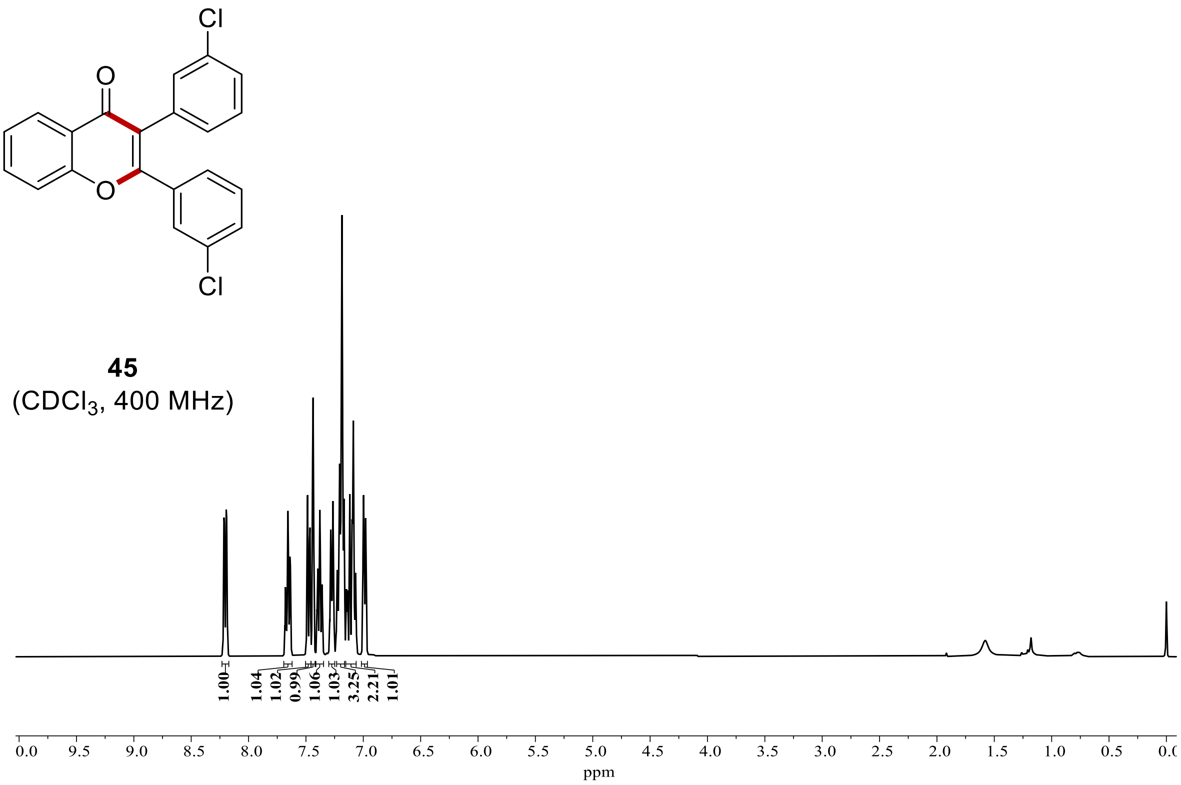

Supplementary Figure112. ^{1}H-NMR spectrum of **45**.

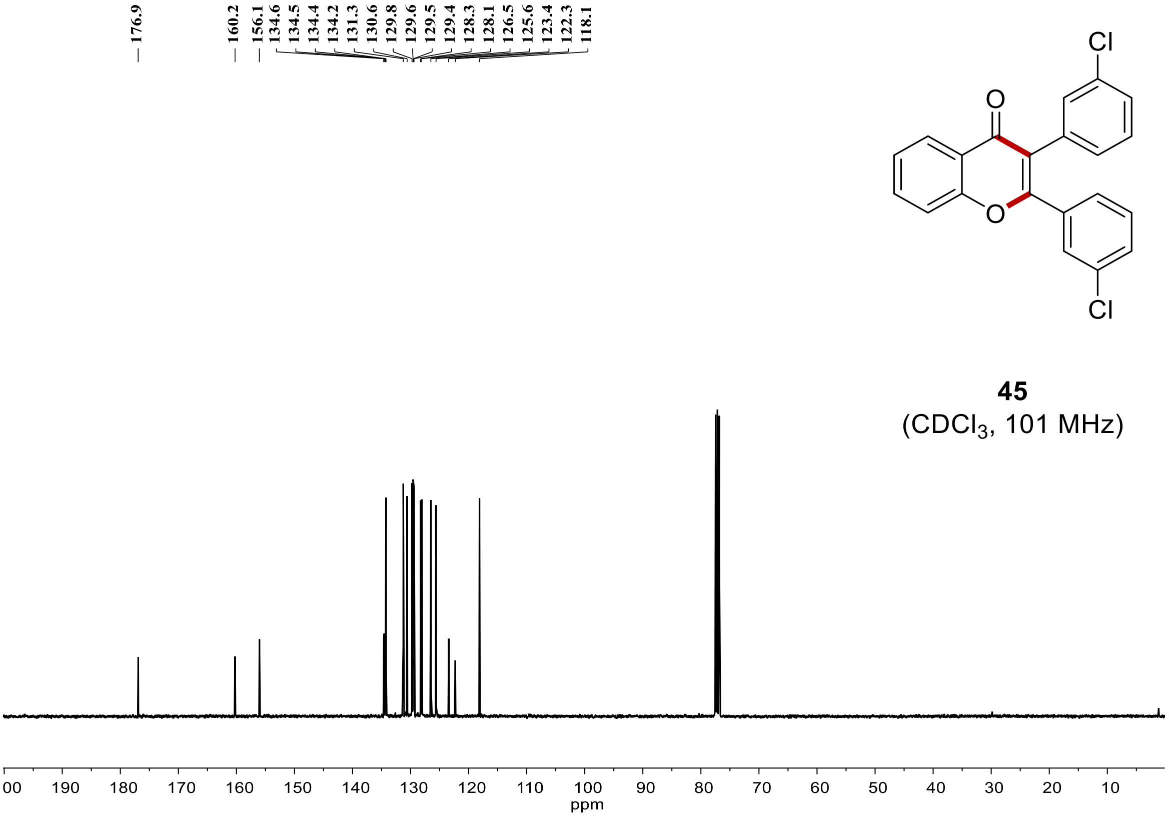

Supplementary Figure 113. ^{13}C-NMR spectrum of **45**.

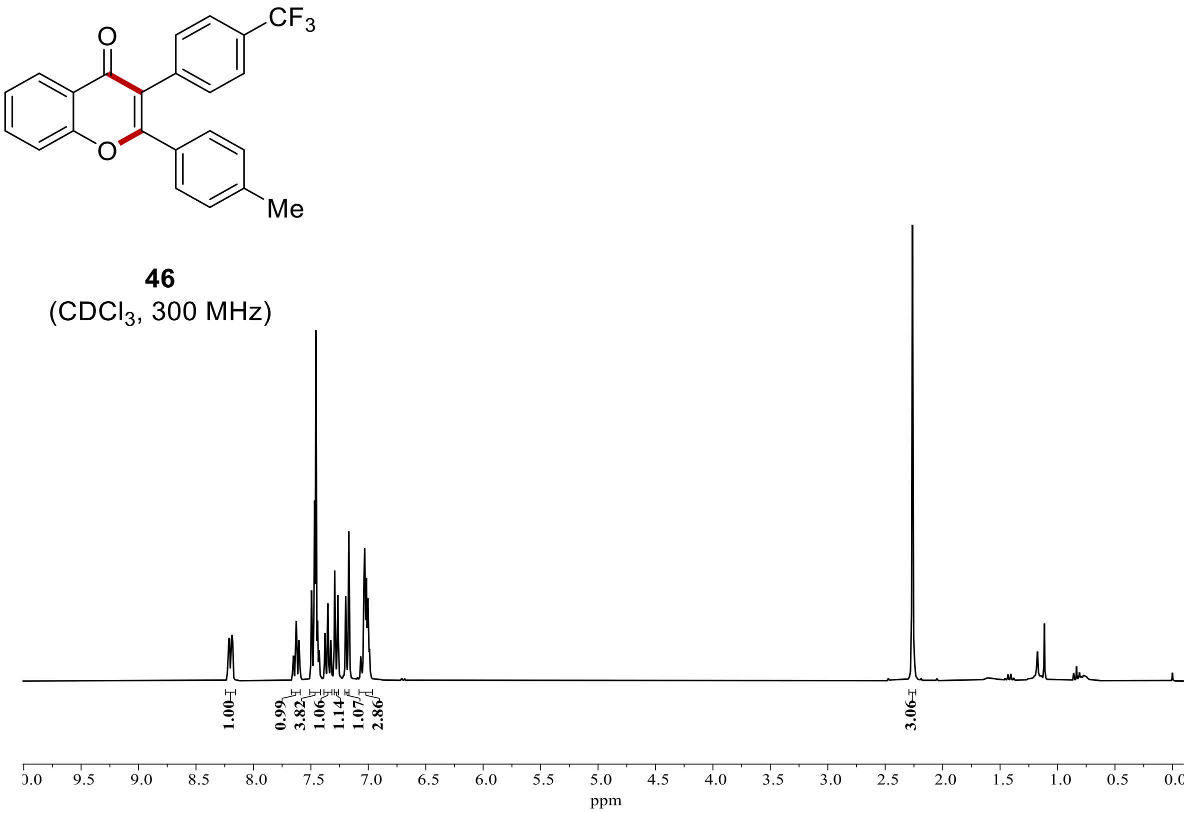

Supplementary Figure 114. ^{1}H-NMR spectrum of **46**.

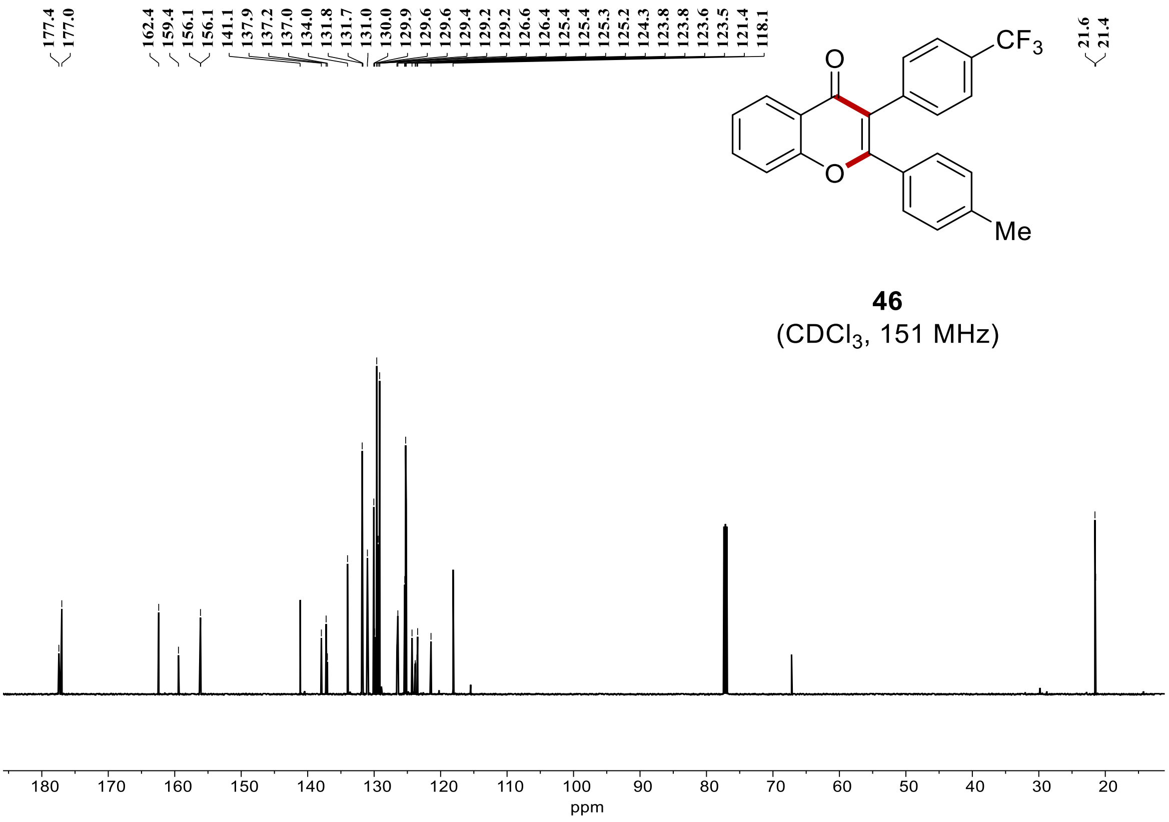

Supplementary Figure 115. ^{13}C-NMR spectrum of **46**.

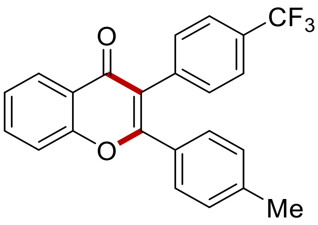

46
($CDCl_3$, 282 MHz)

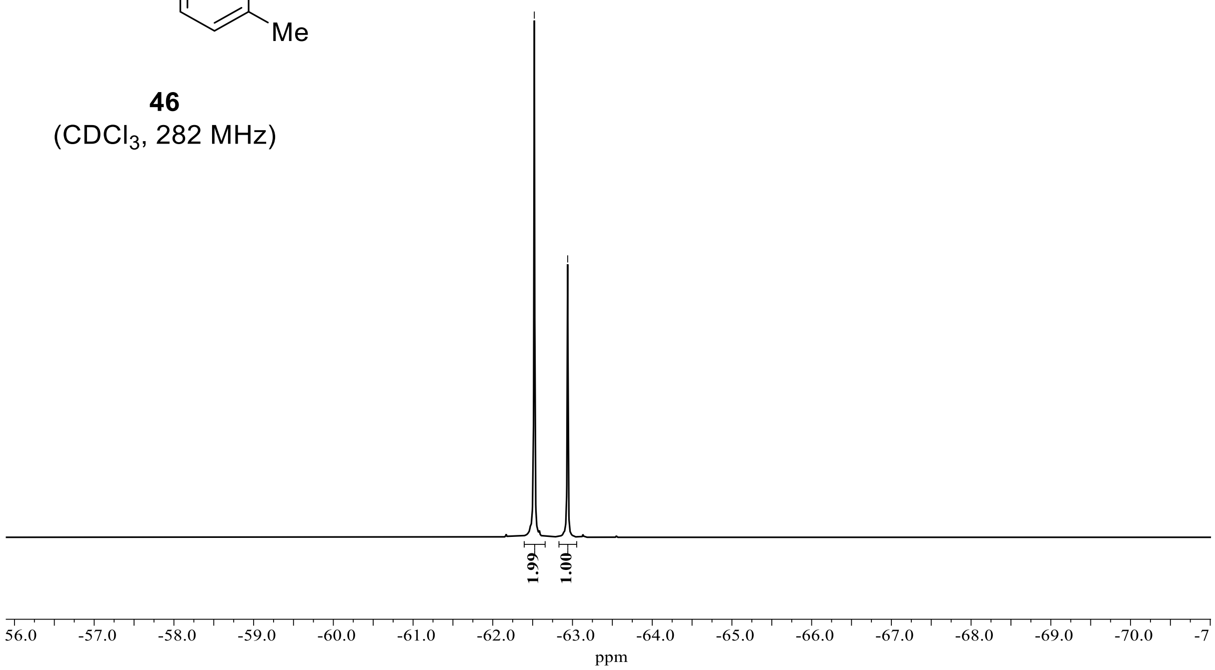

Supplementary Figure 116. ^{19}F-NMR spectrum of **46**.

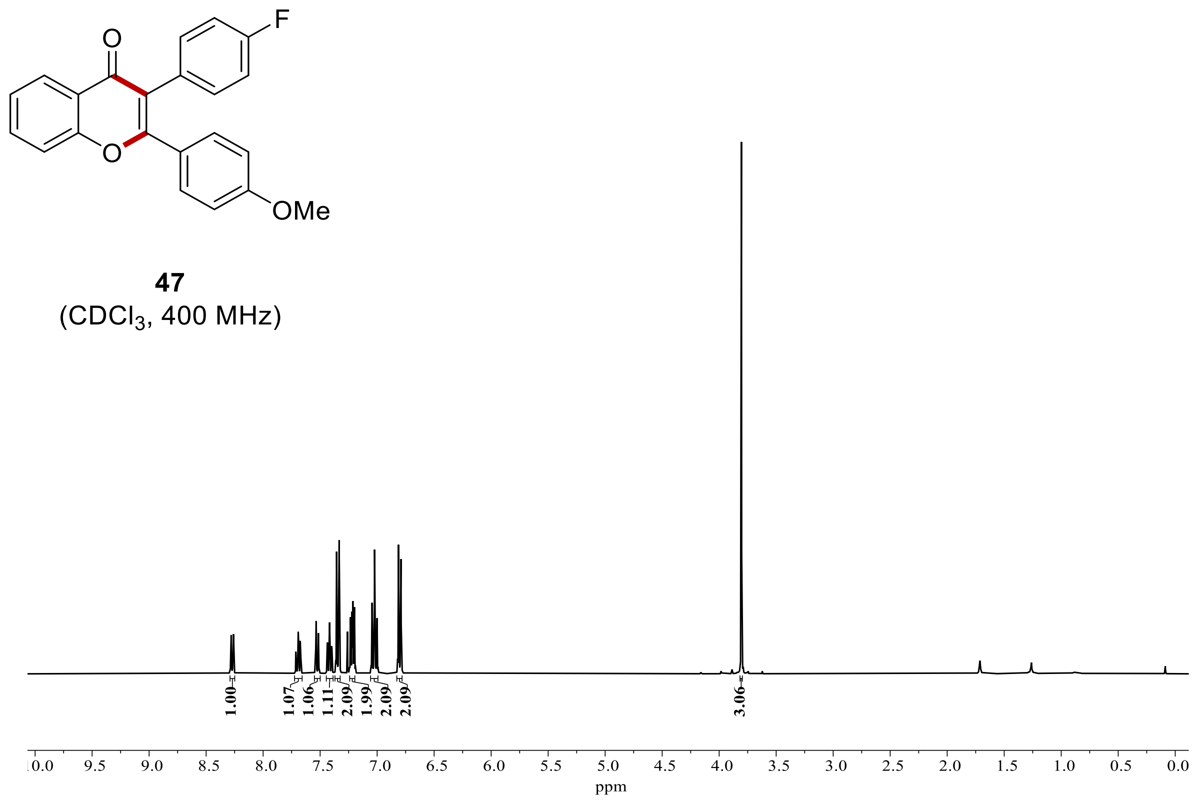

Supplementary Figure 117. ^{1}H-NMR spectrum of **47**.

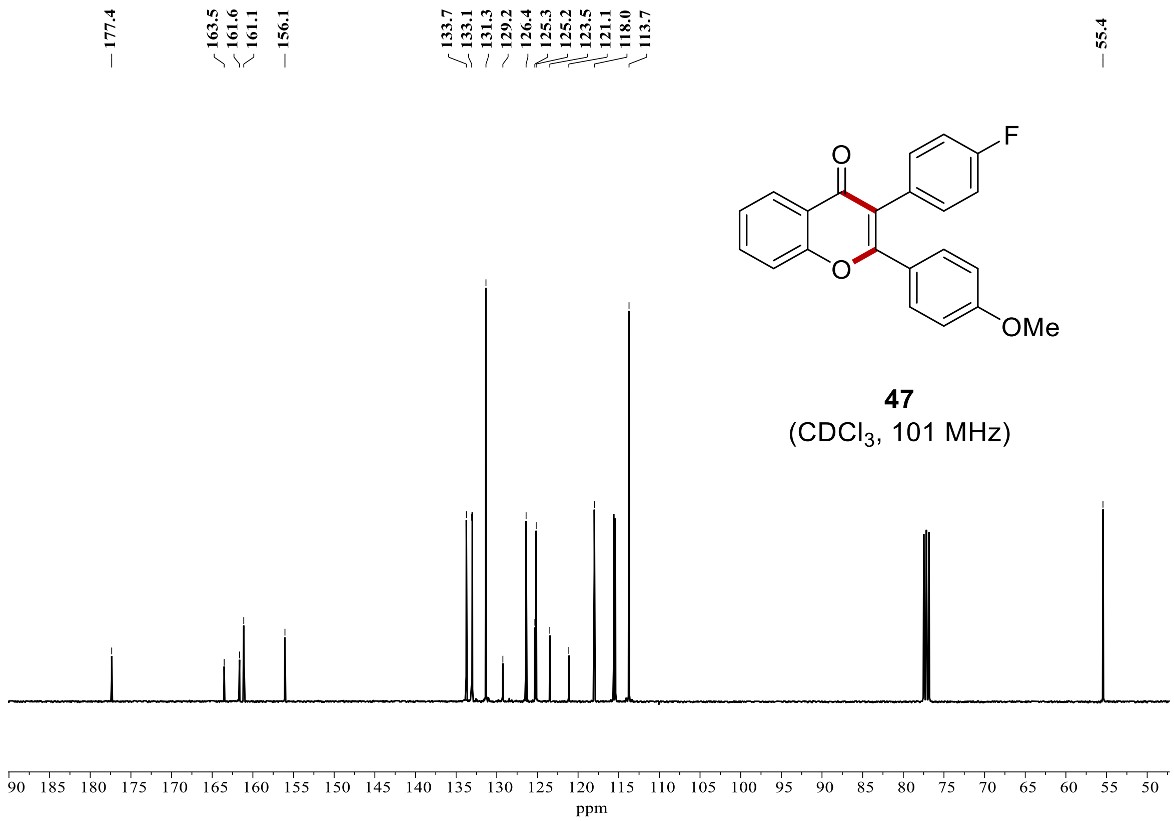

Supplementary Figure 118. ^{13}C-NMR spectrum of **47**.

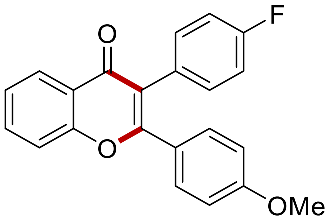

47
($CDCl_3$, 377 MHz)

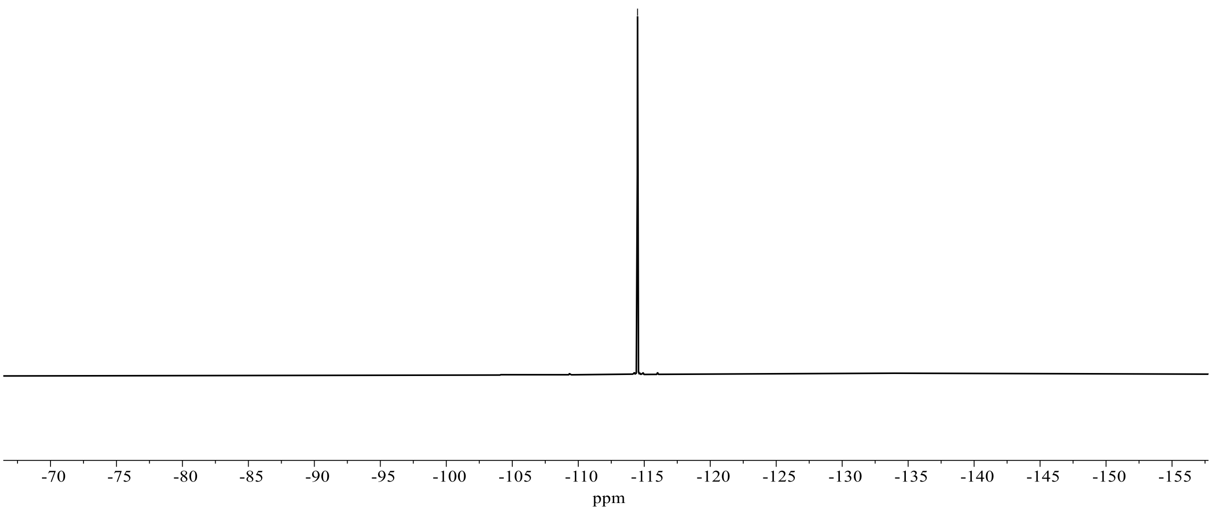

Supplementary Figure 119. ^{19}F-NMR spectrum of **47**.

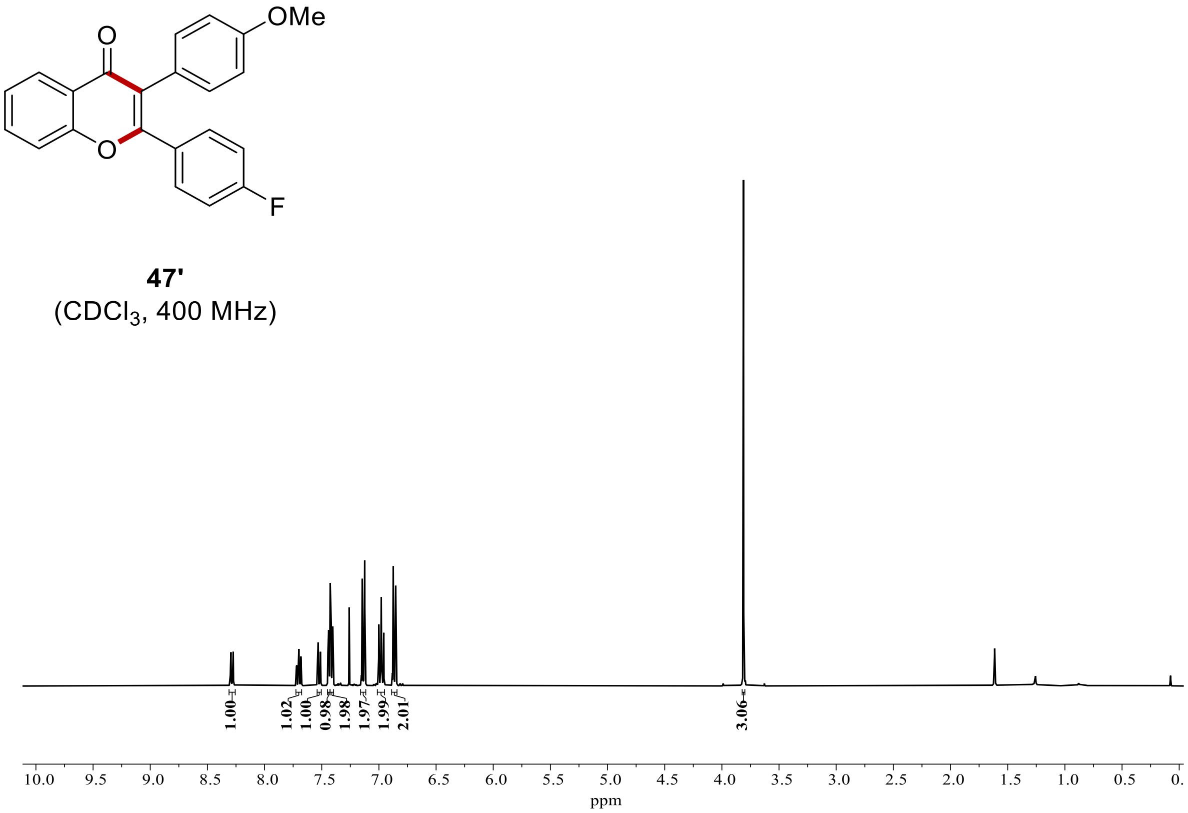

Supplementary Figure 120. ^{1}H-NMR spectrum of **47'**.

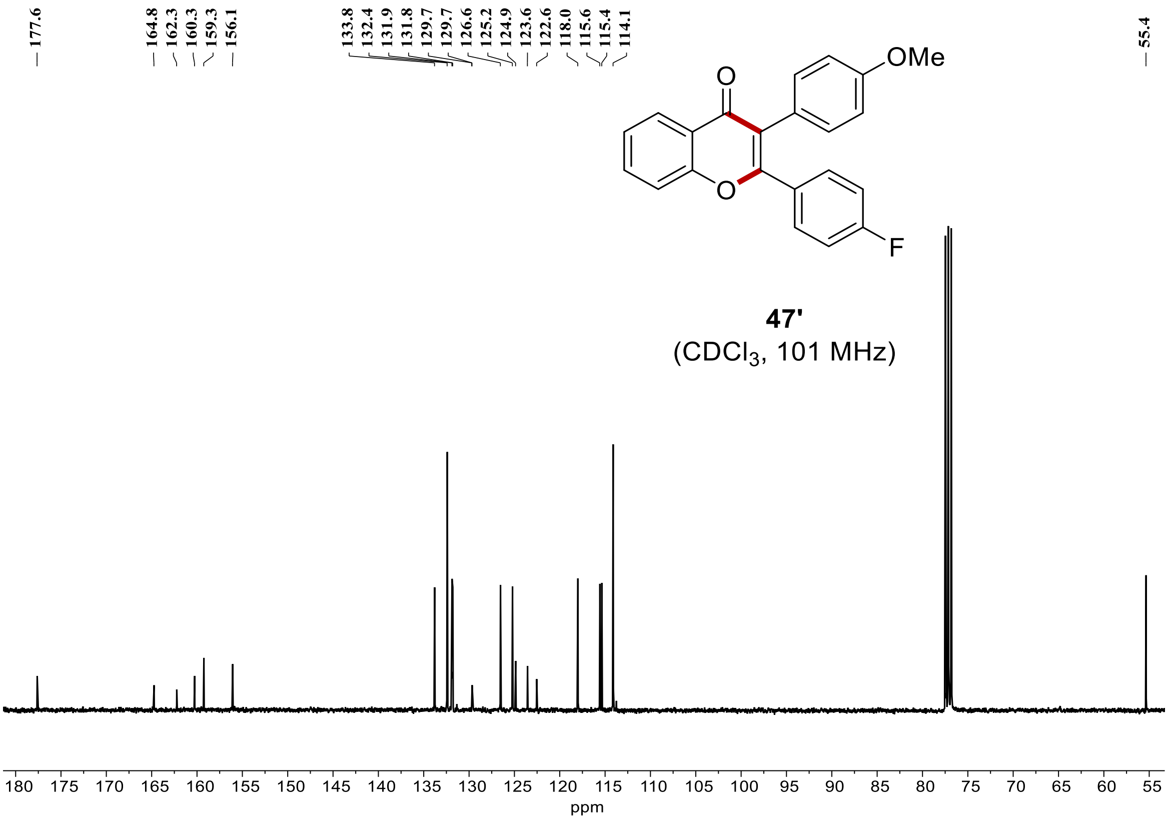

Supplementary Figure 121. ^{13}C-NMR spectrum of **47'**.

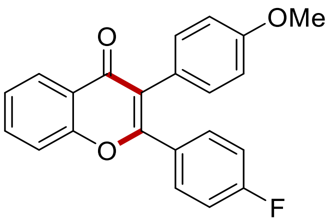

47'
($CDCl_3$, 377 MHz)

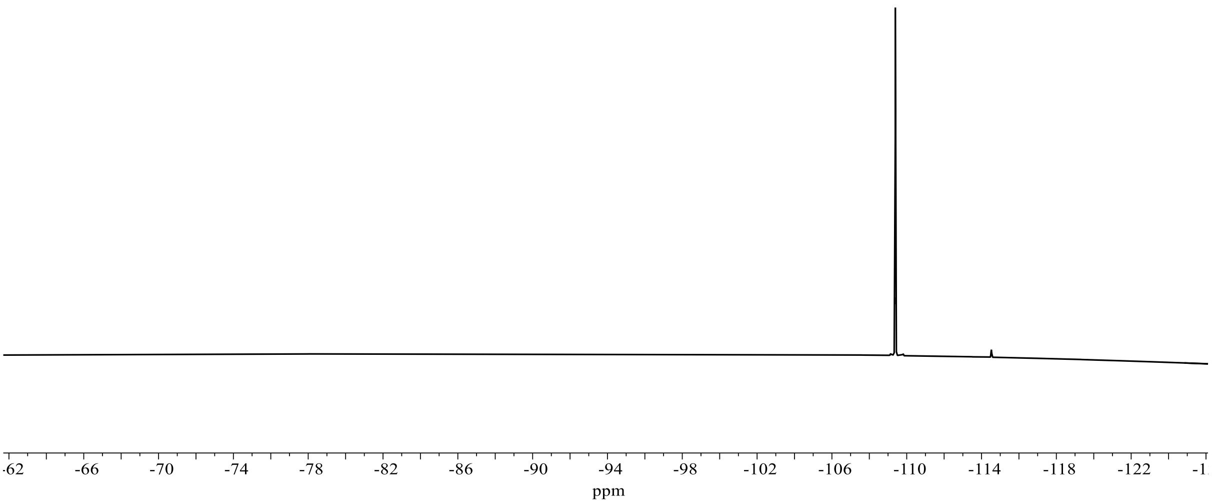

Supplementary Figure 122. ^{19}F-NMR spectrum of **47’**.

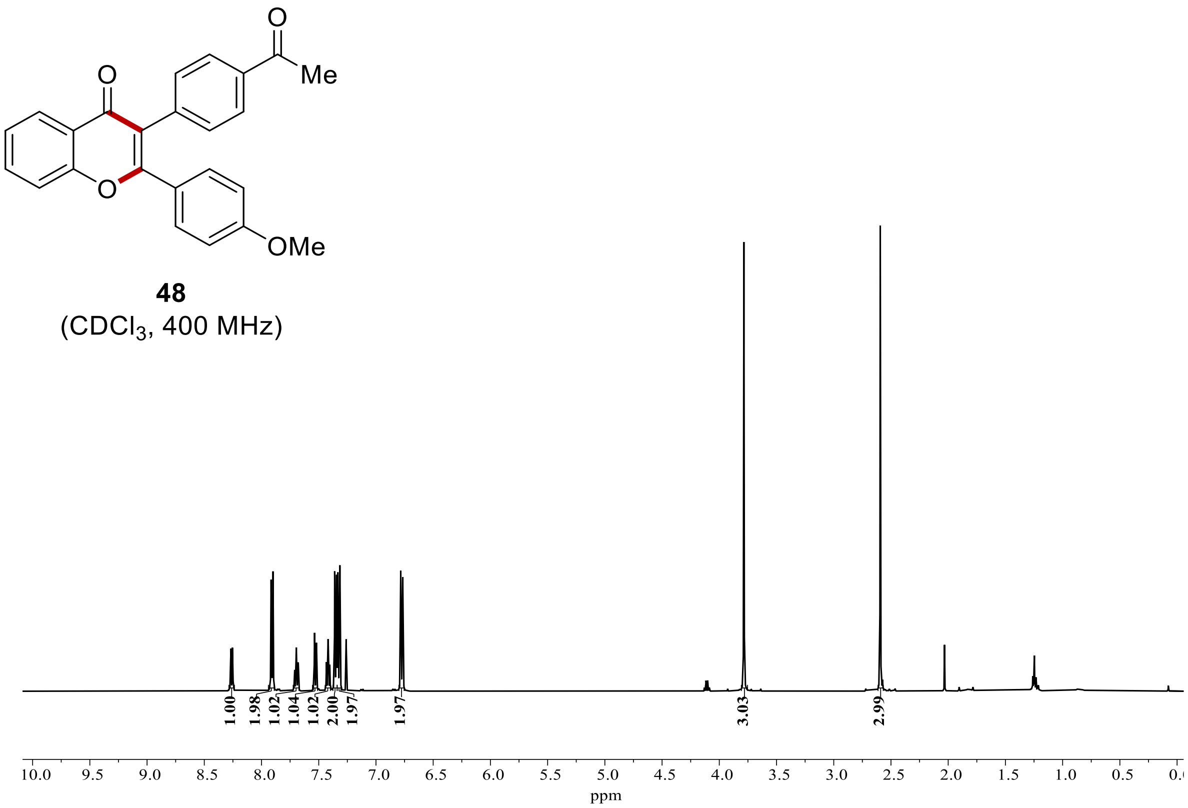

Supplementary Figure 123. ^{1}H-NMR spectrum of **48**.

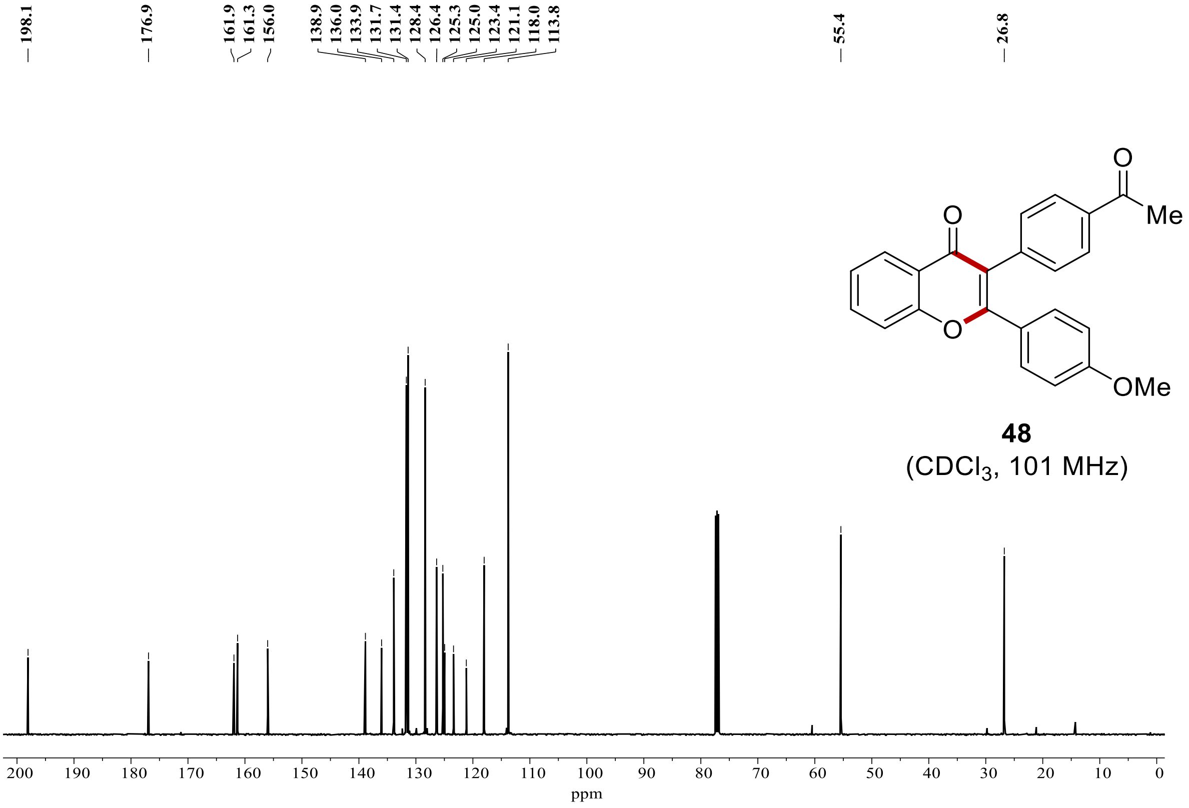

Supplementary Figure 124. ^{13}C-NMR spectrum of **48**.

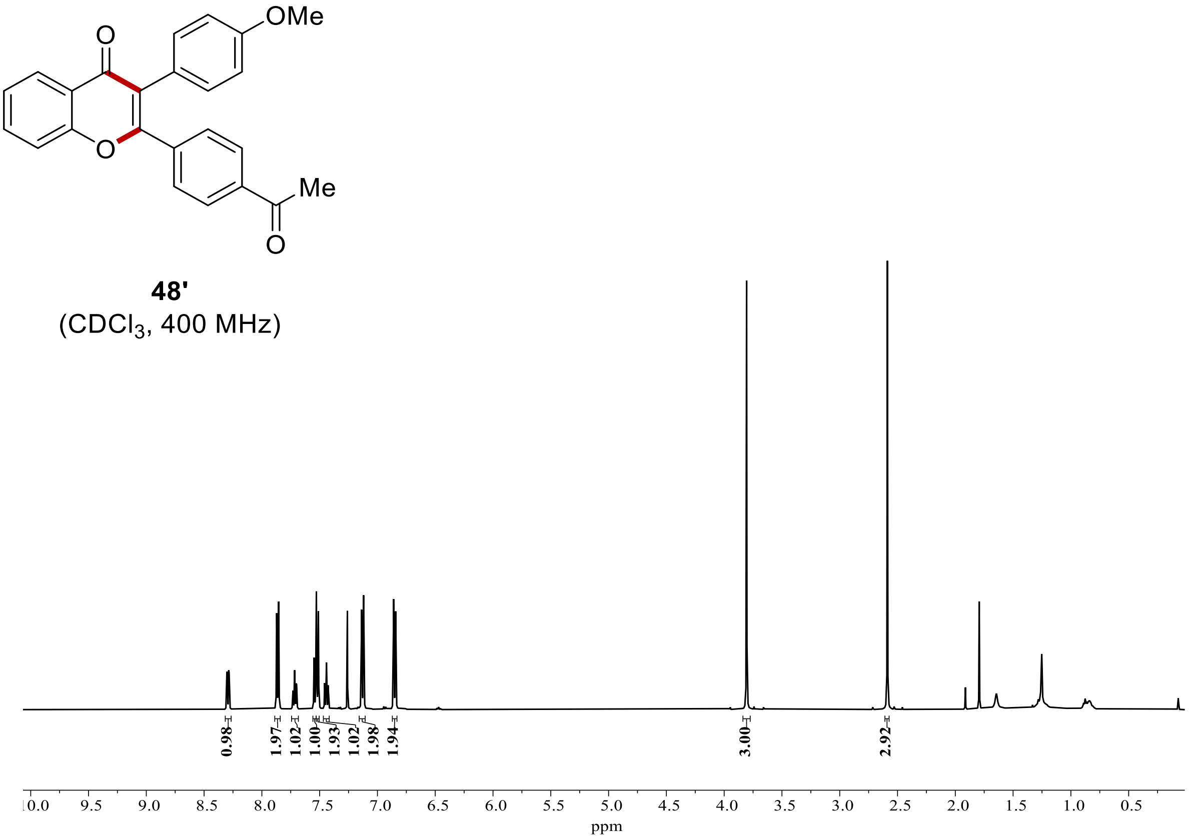

Supplementary Figure 125. ^{1}H-NMR spectrum of **48’**.

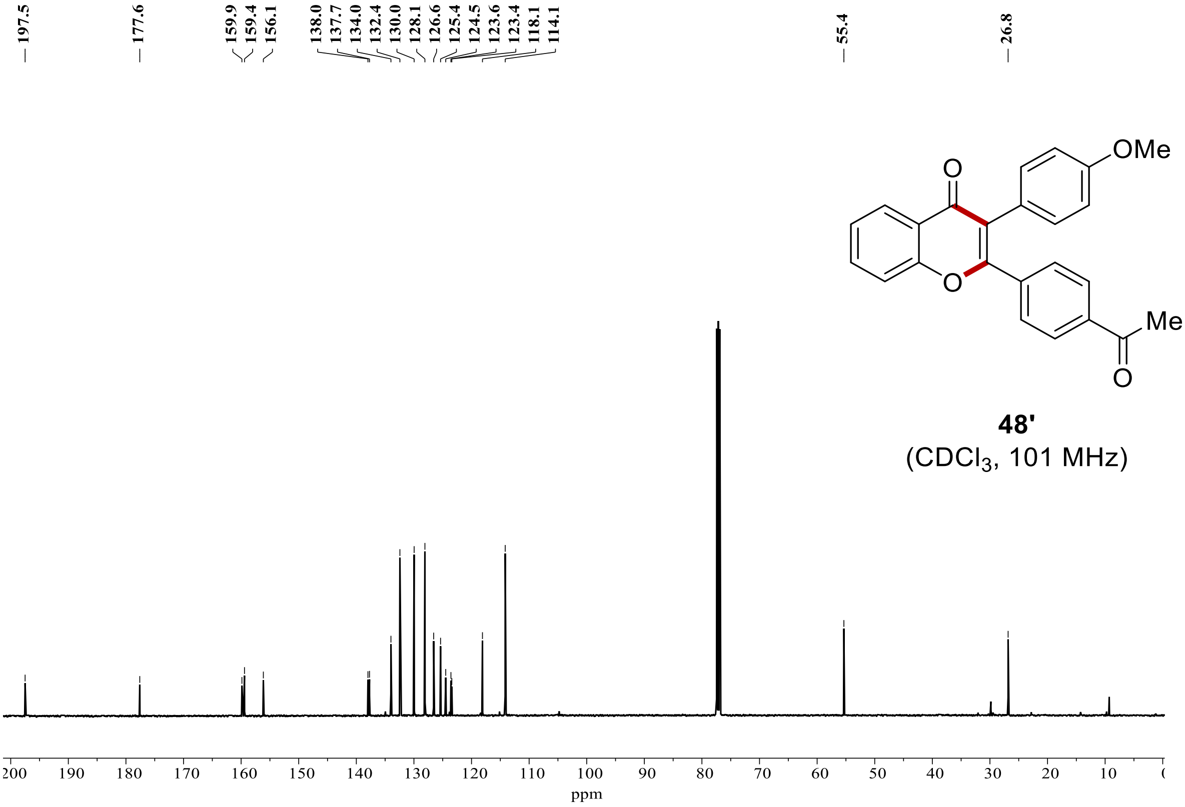

Supplementary Figure 126. ^{13}C-NMR spectrum of **48’**.

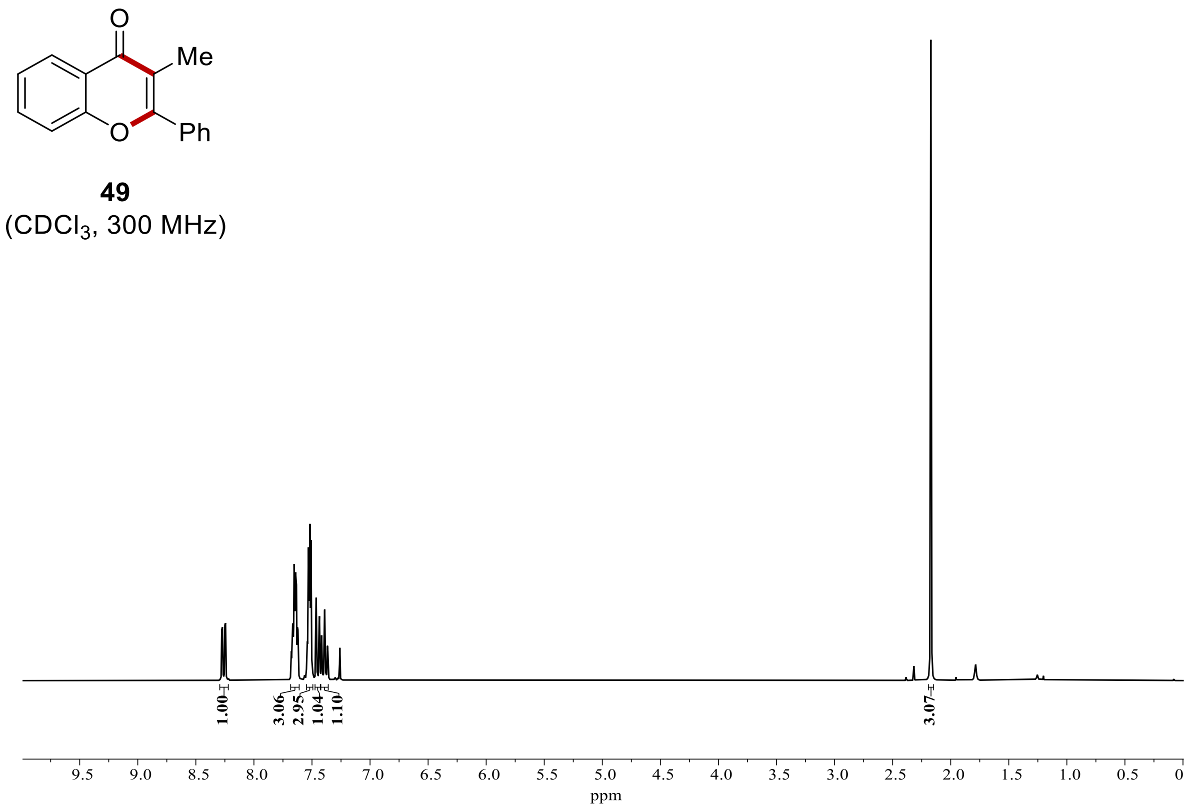

Supplementary Figure 127. ^{1}H-NMR spectrum of **49**.

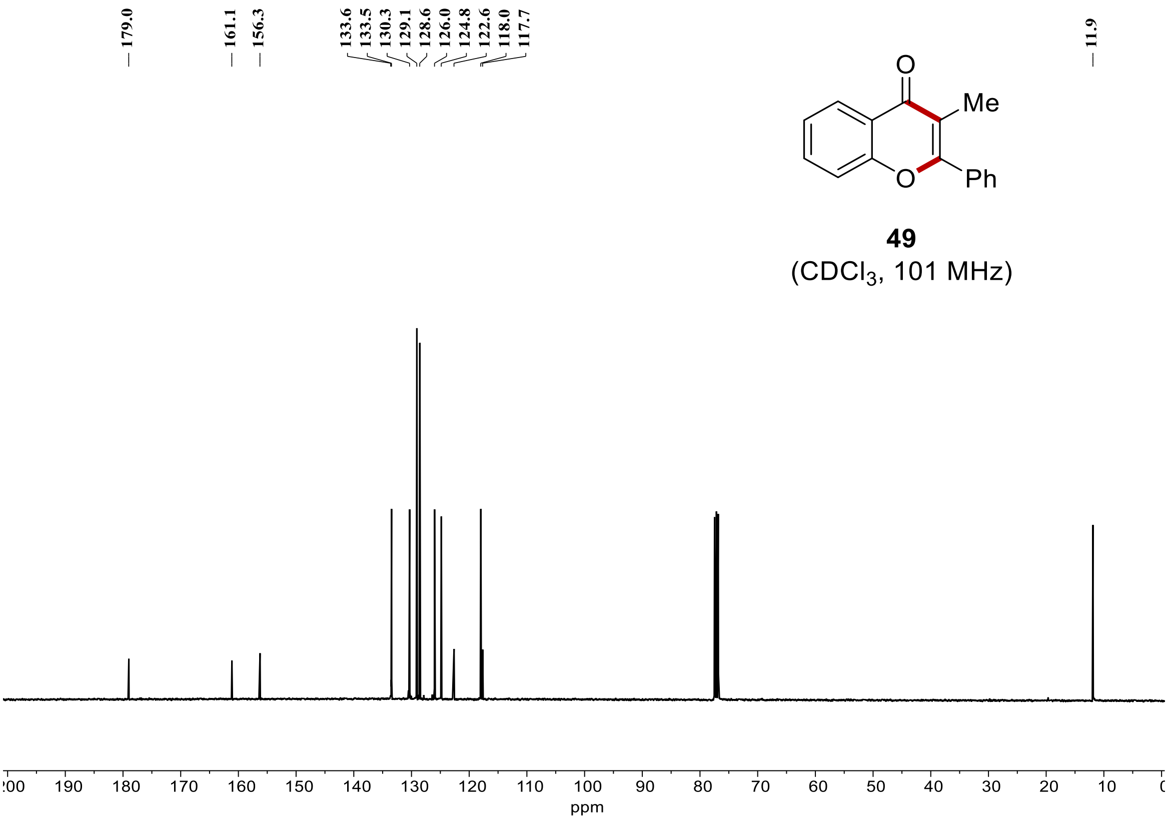

Supplementary Figure 128. ^{13}C-NMR spectrum of **49**.

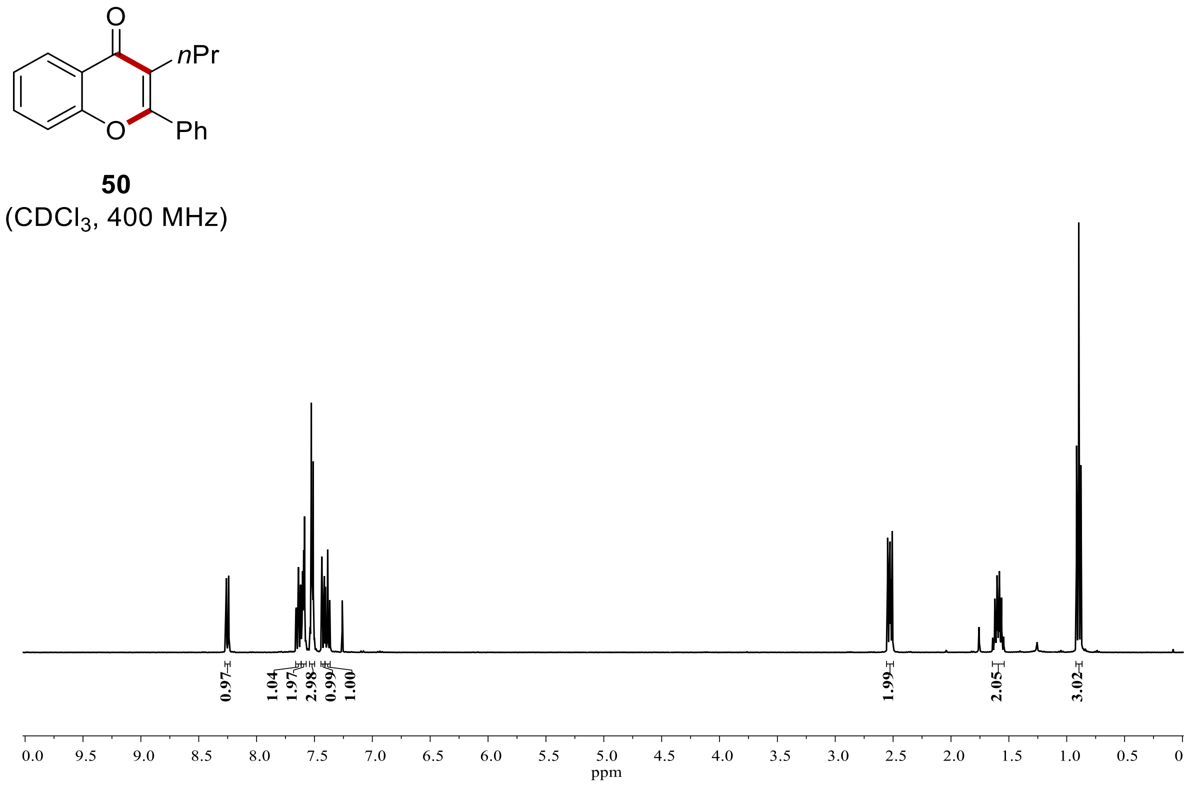

Supplementary Figure 129. ^{1}H-NMR spectrum of **50**.

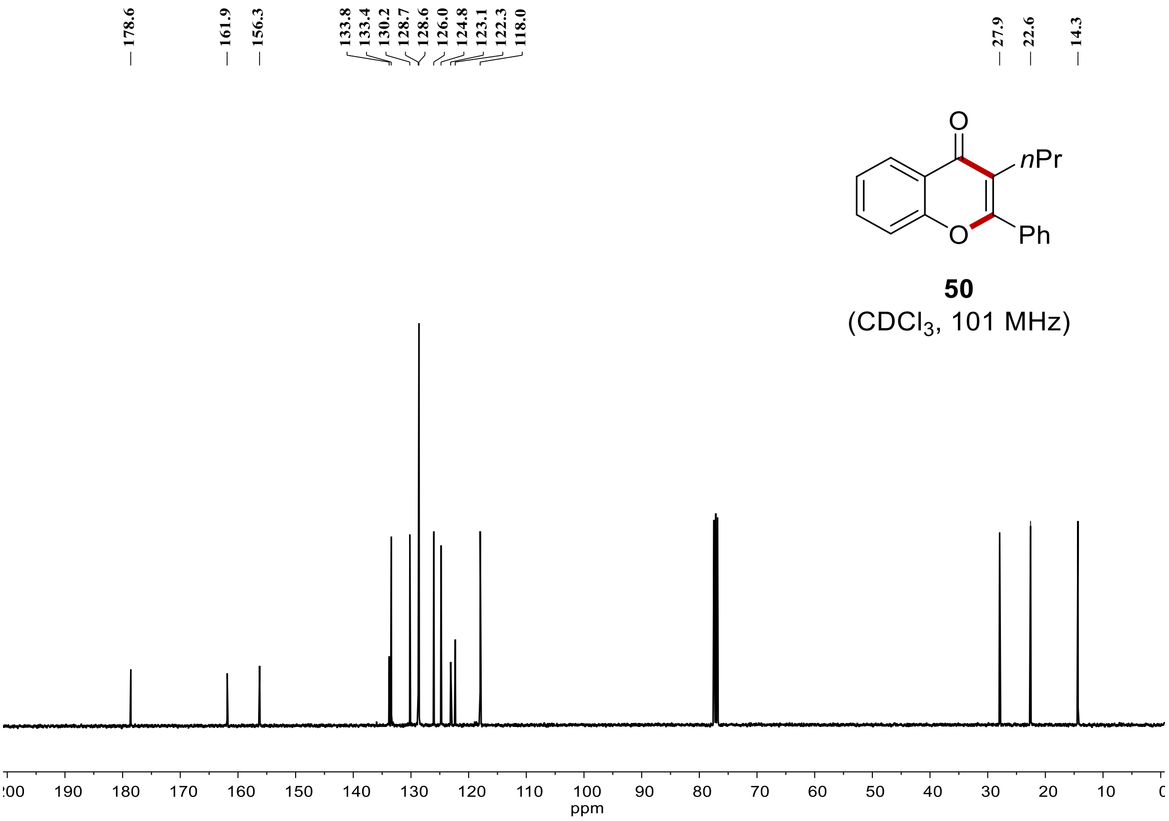

Supplementary Figure 130. ^{13}C-NMR spectrum of **50**.

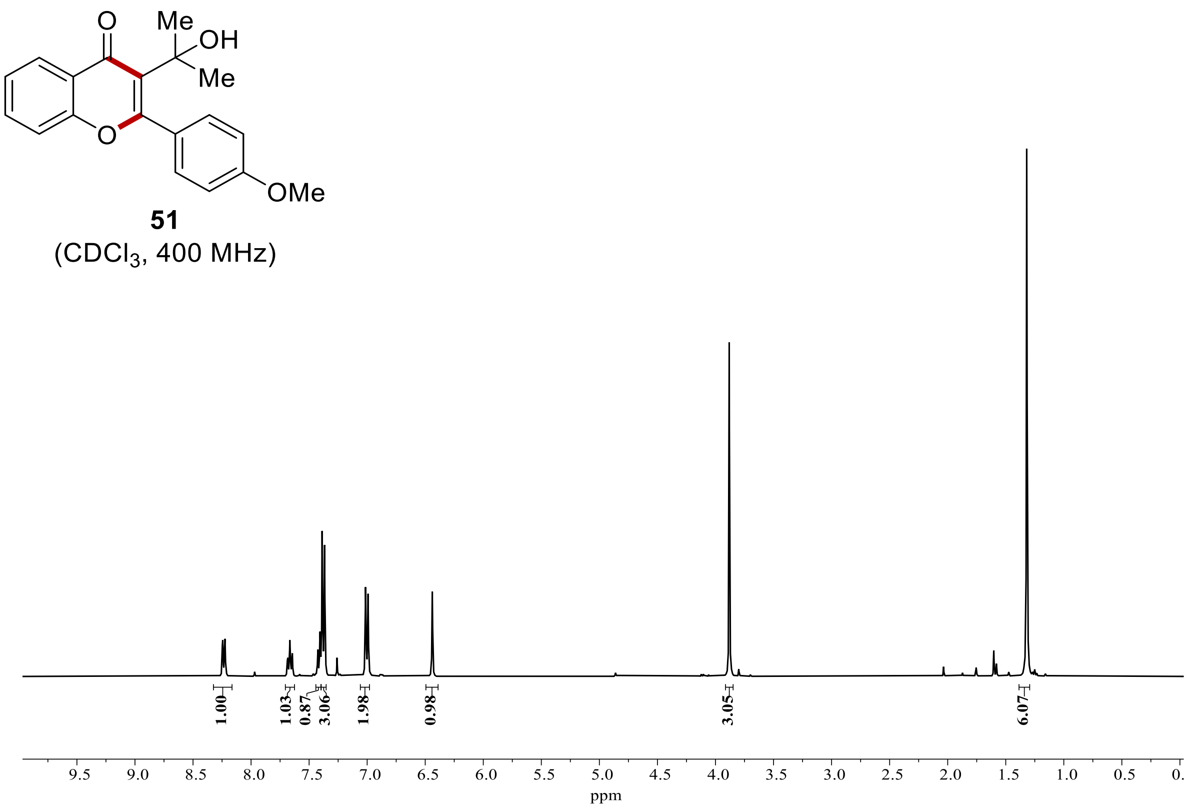

Supplementary Figure 131. ^{1}H-NMR spectrum of **51**.

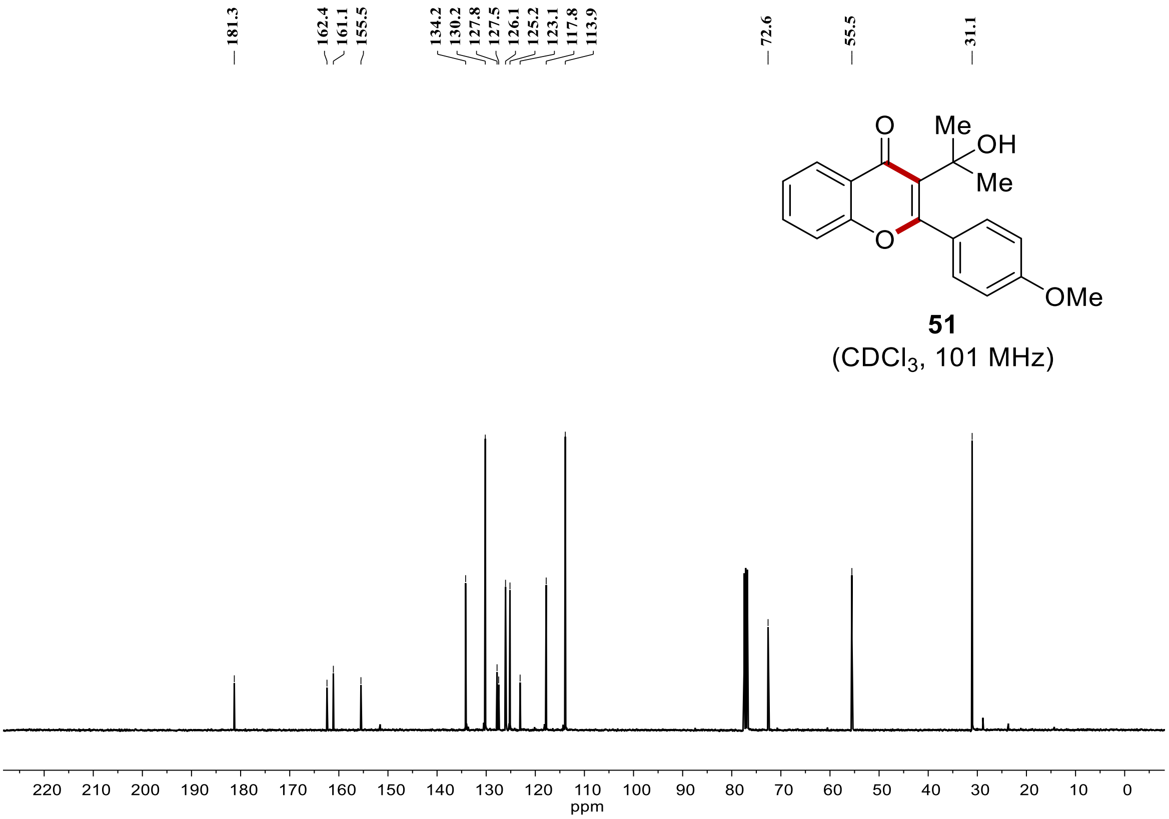

Supplementary Figure 132. ^{13}C-NMR spectrum of **51**.

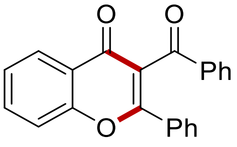

52
($CDCl_3$, 400 MHz)

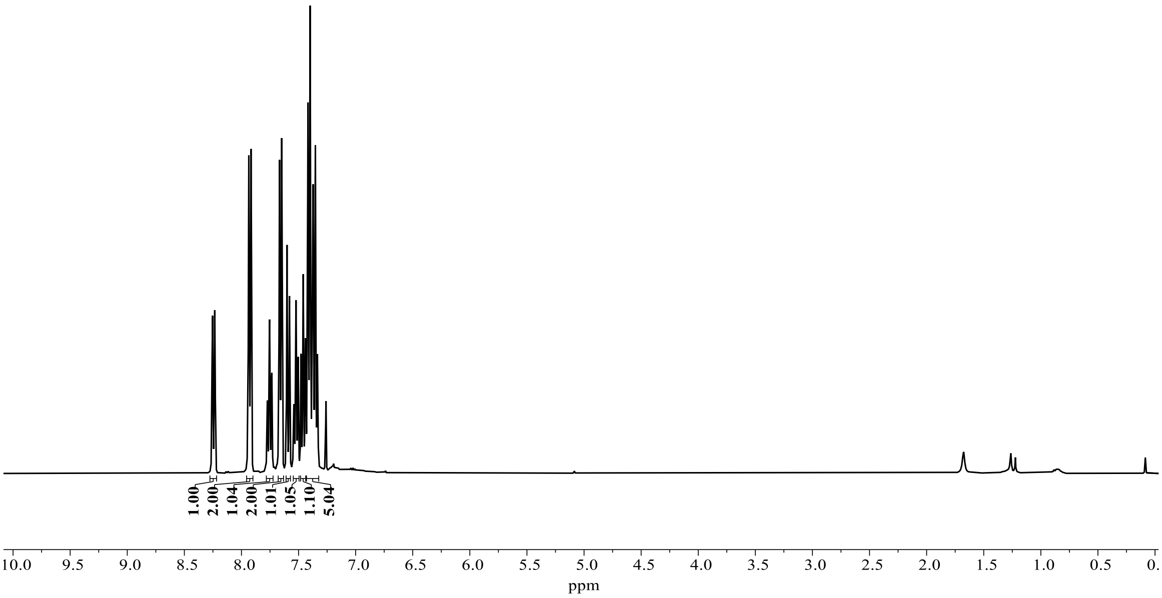

Supplementary Figure 133. ^{1}H-NMR spectrum of **52**.

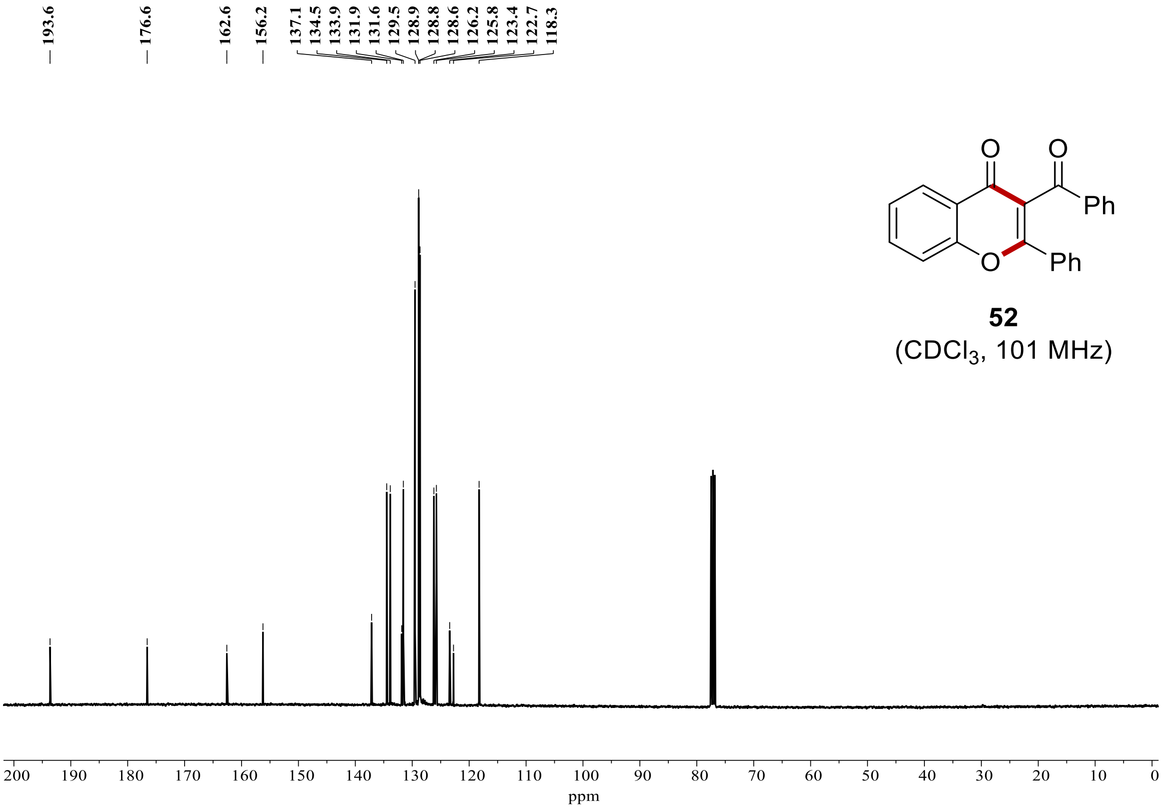

52
($CDCl_3$, 101 MHz)

Supplementary Figure 134. ^{13}C-NMR spectrum of **52.**

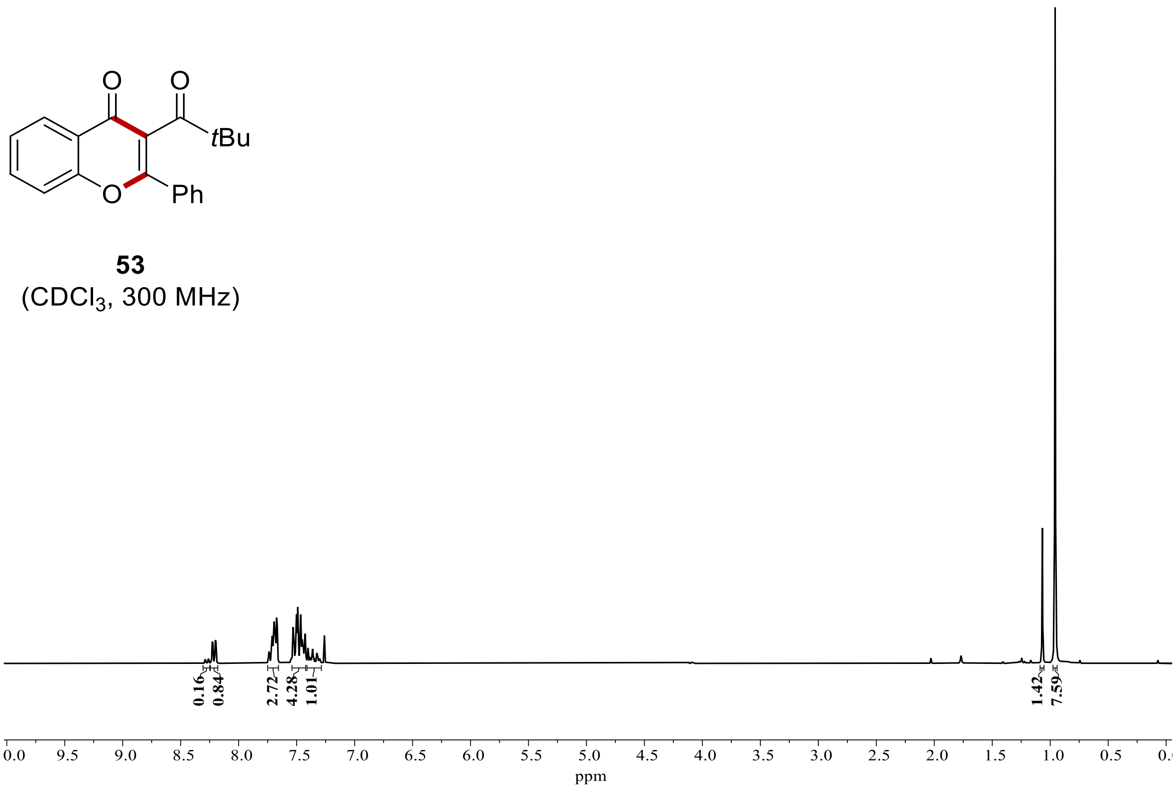

Supplementary Figure 135. ^{1}H-NMR spectrum of **53**.

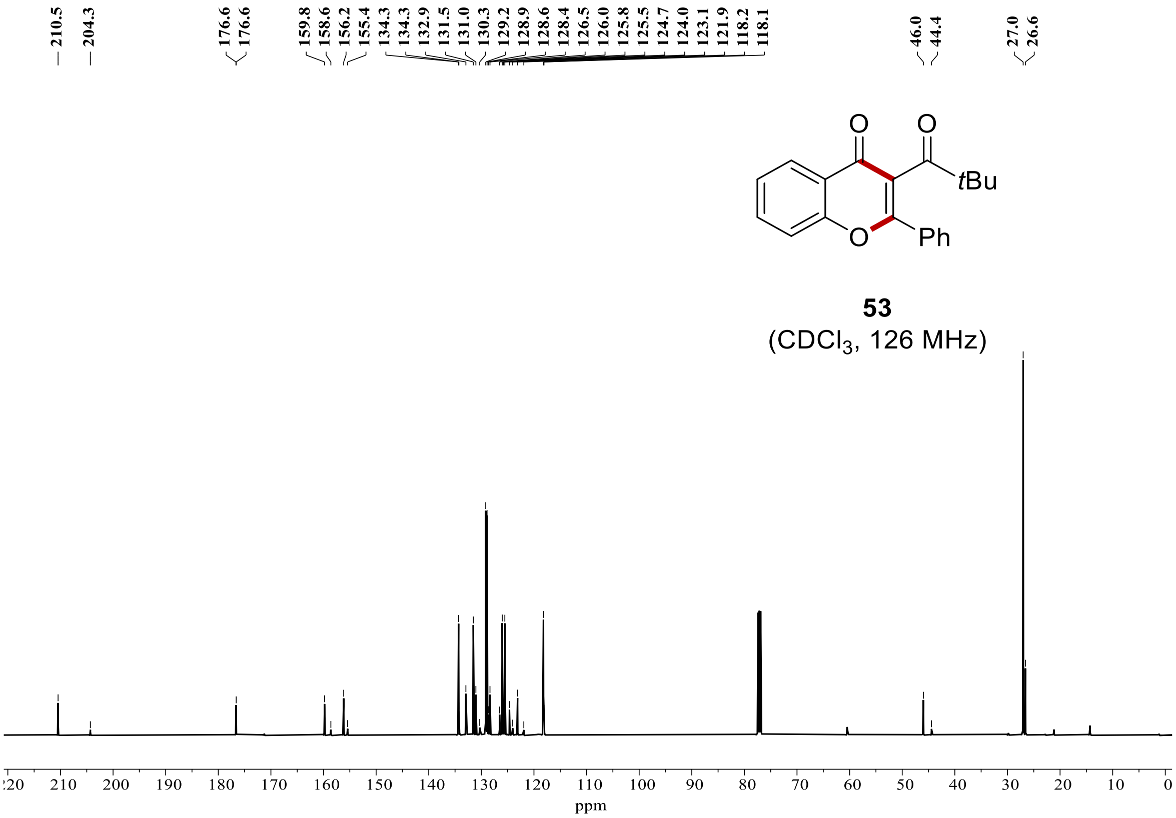

Supplementary Figure 136. ^{13}C-NMR spectrum of **53.**

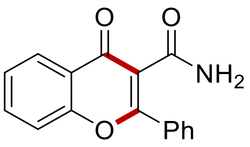

54
($CDCl_3$, 400 MHz)

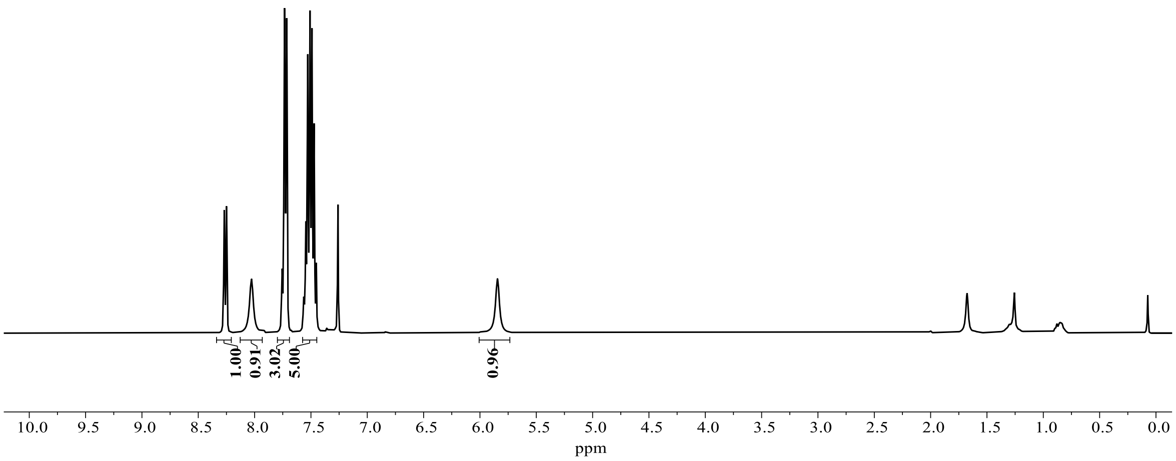

Supplementary Figure 137. ^{1}H-NMR spectrum of **54**.

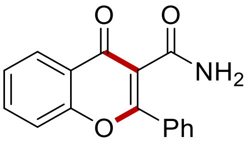

54
($CDCl_3$, 101 MHz)

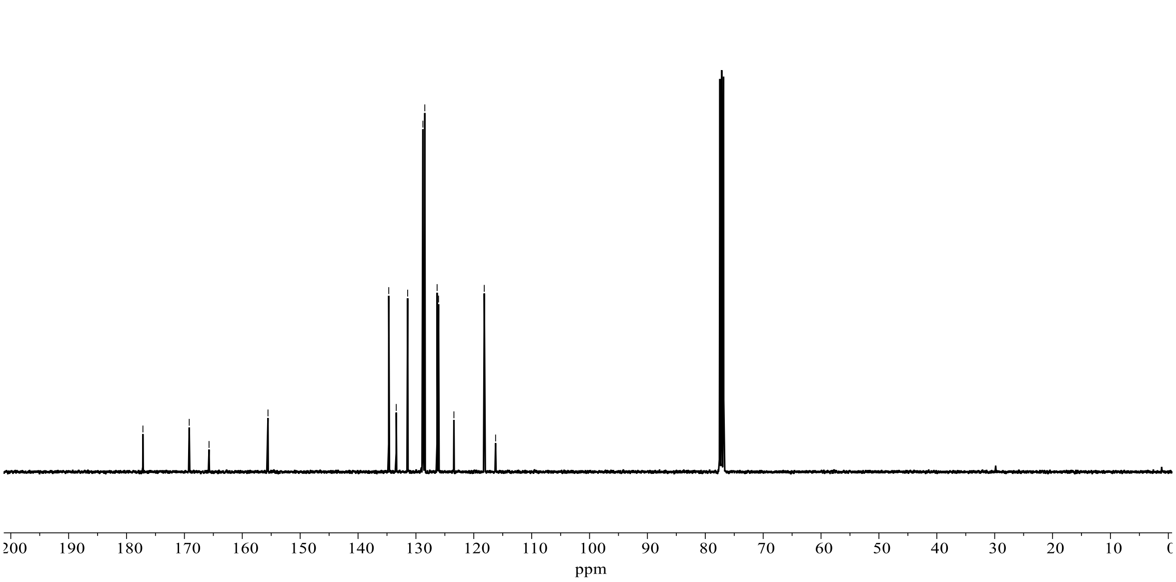

Supplementary Figure 138. ^{13}C-NMR spectrum of **54.**

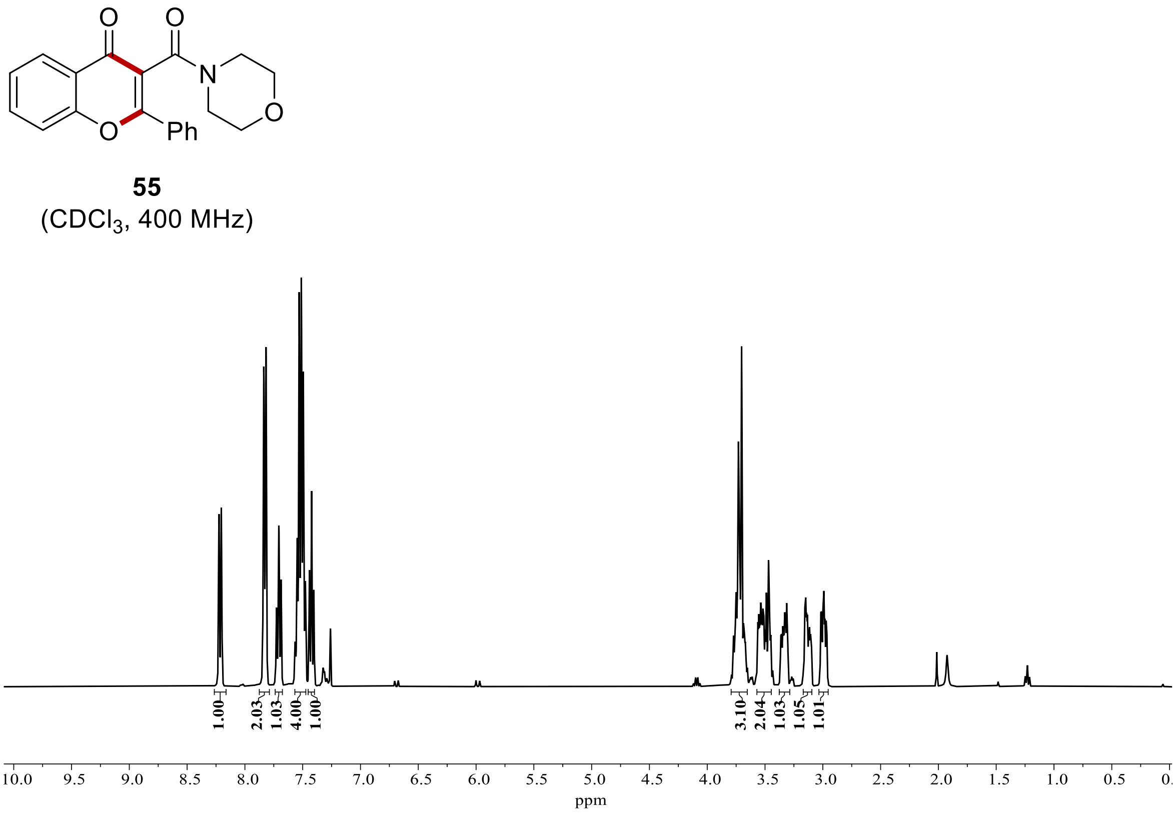

Supplementary Figure 139. ^{1}H-NMR spectrum of **55**.

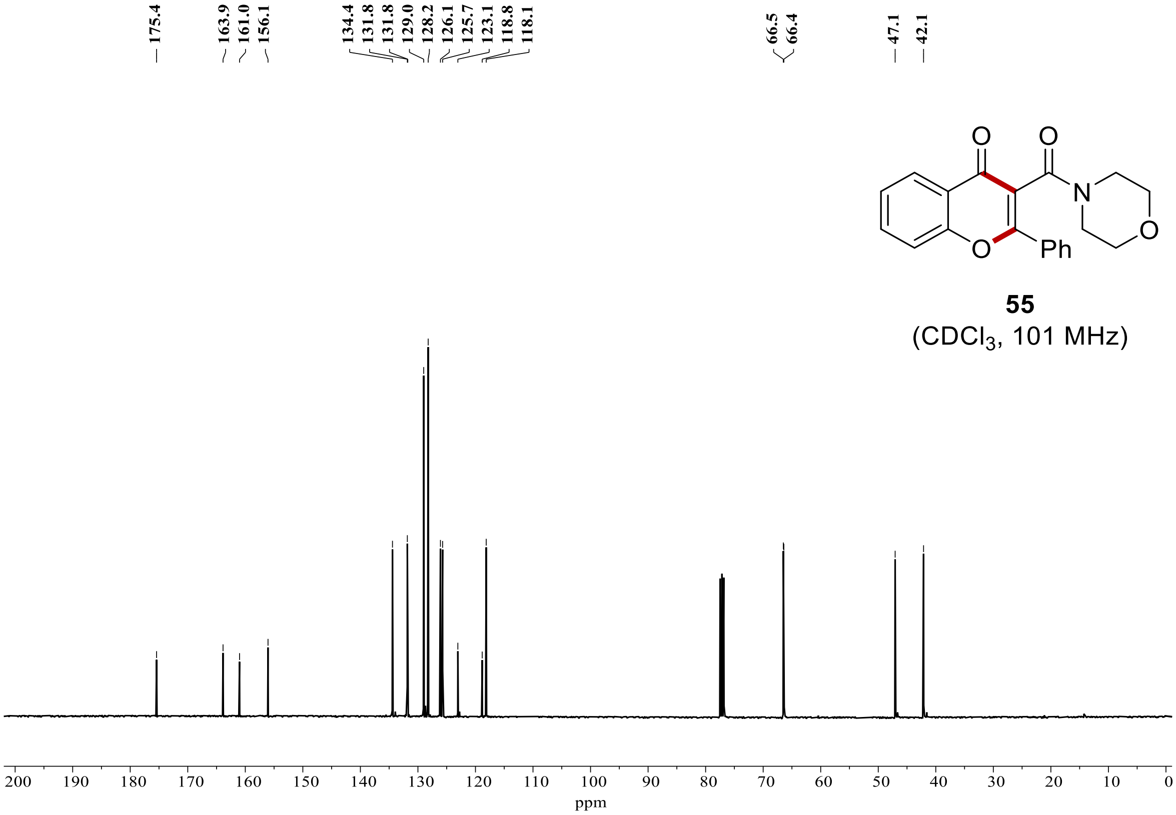

Supplementary Figure 140. ^{13}C-NMR spectrum of **55.**

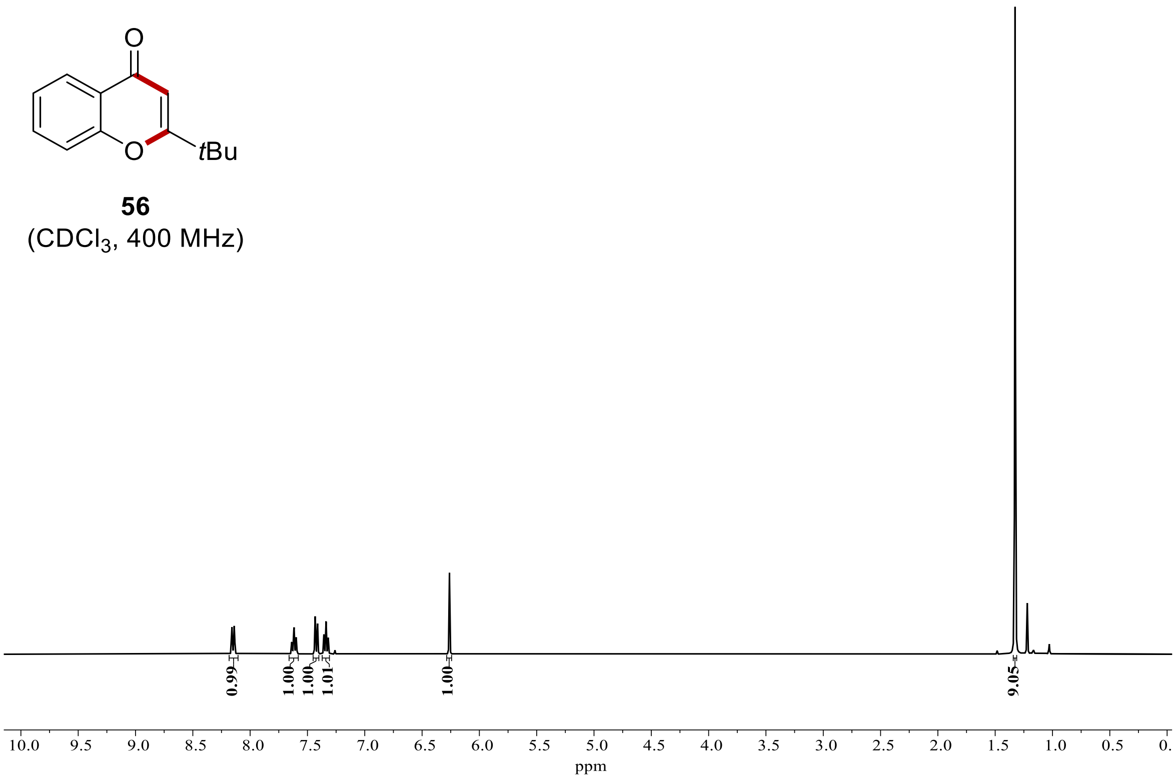

Supplementary Figure 141. ^{1}H-NMR spectrum of **56**.

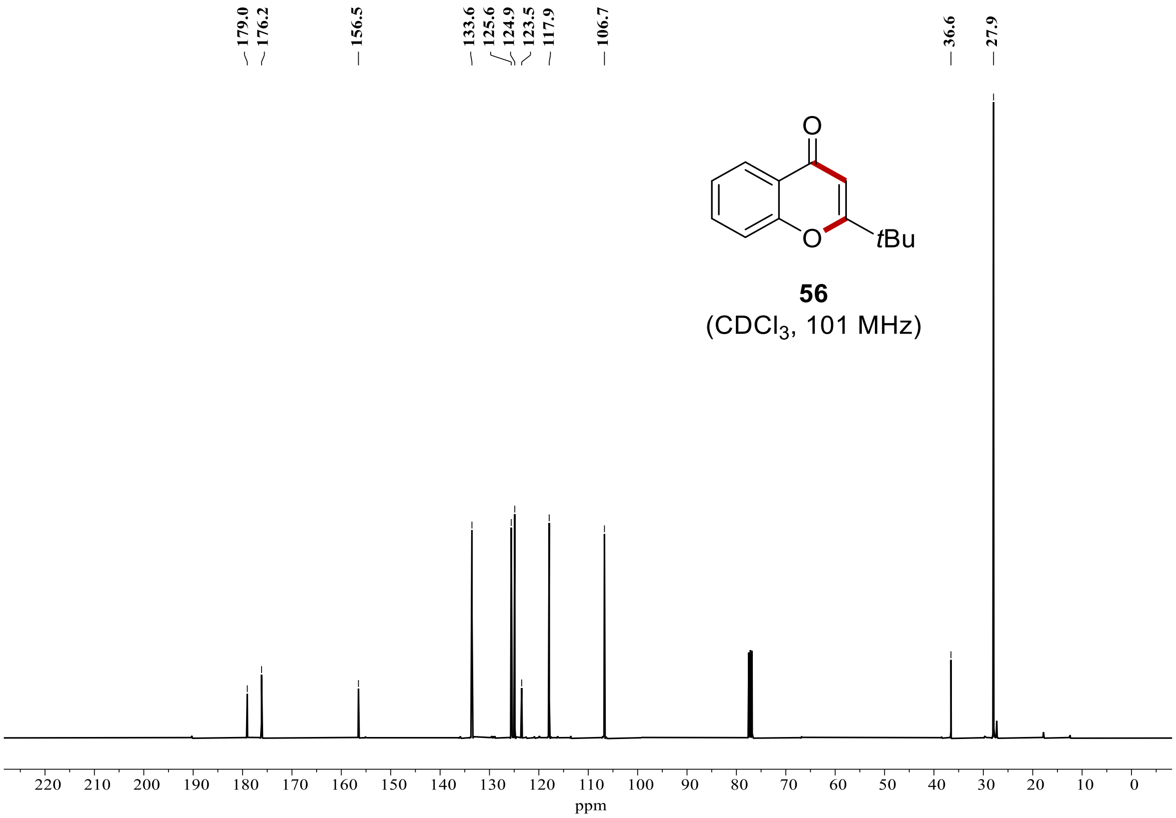

Supplementary Figure 142. ^{13}C-NMR spectrum of **56**.

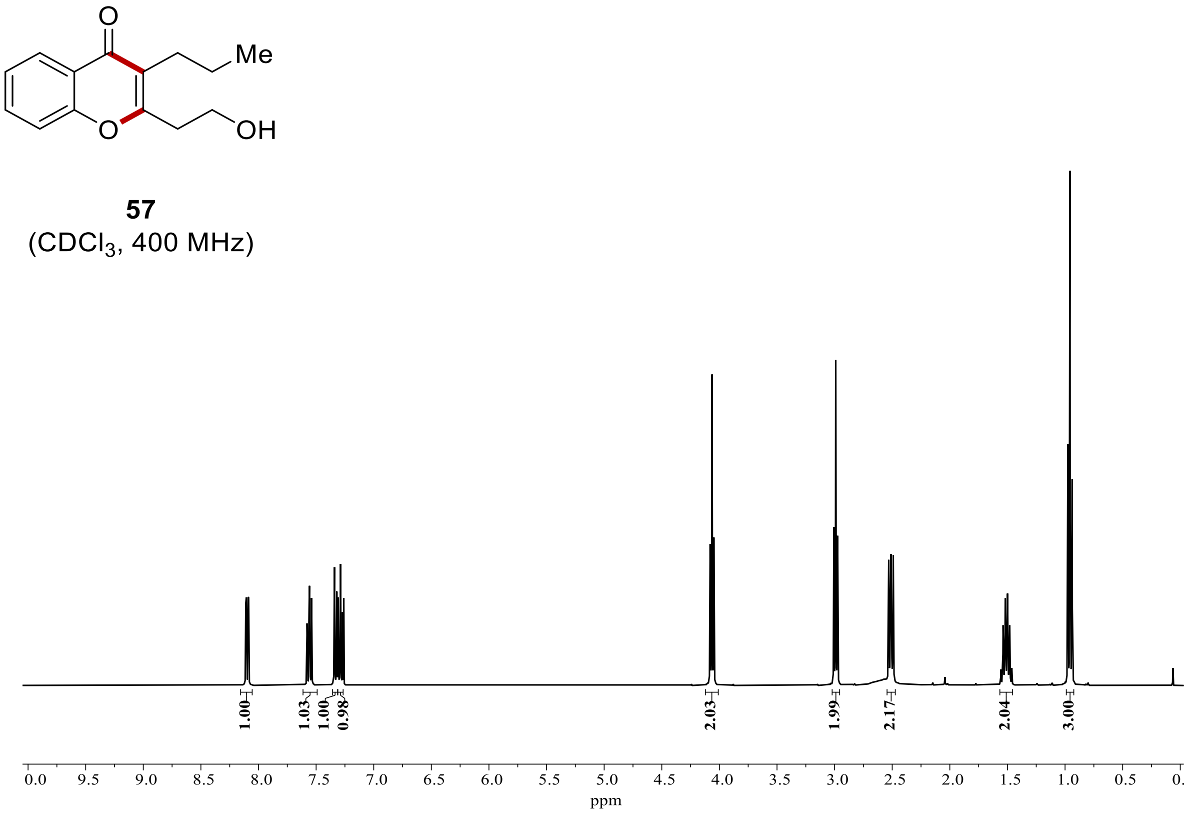

Supplementary Figure 143. ^{1}H-NMR spectrum of **57**.

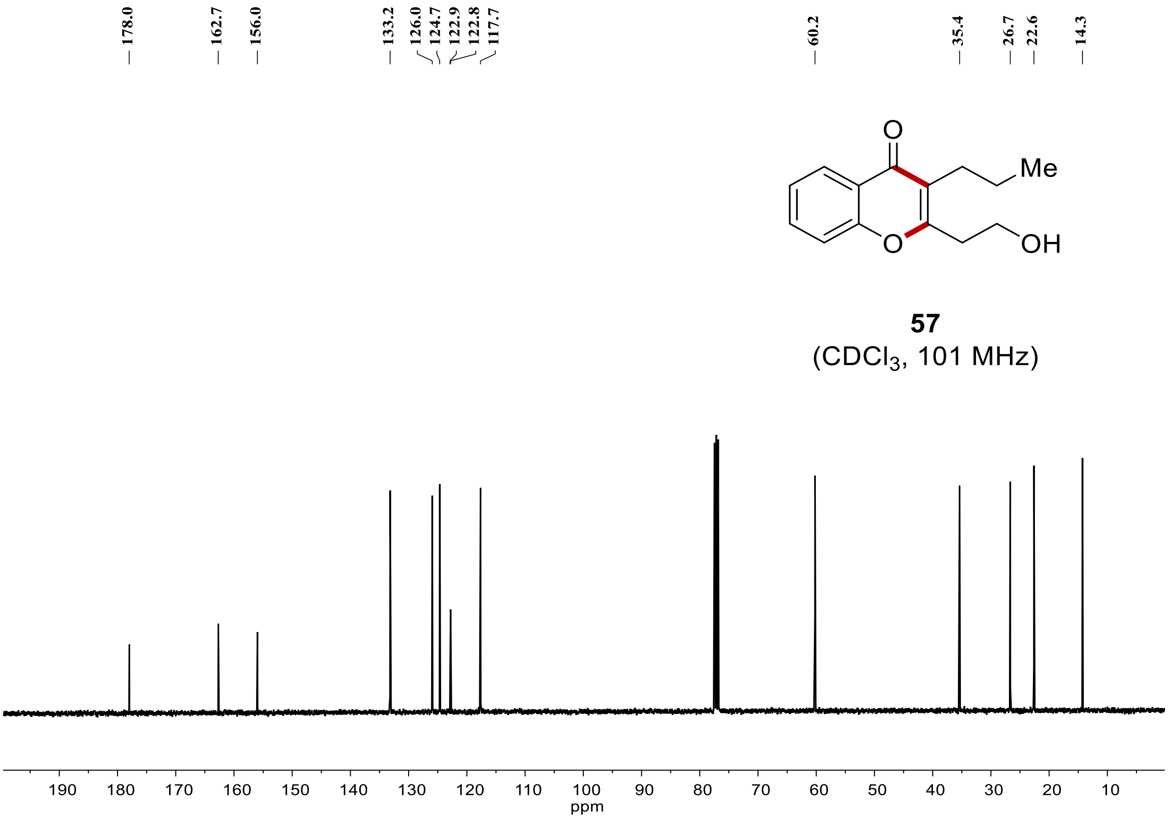

Supplementary Figure 144. ^{13}C-NMR spectrum of **57**.

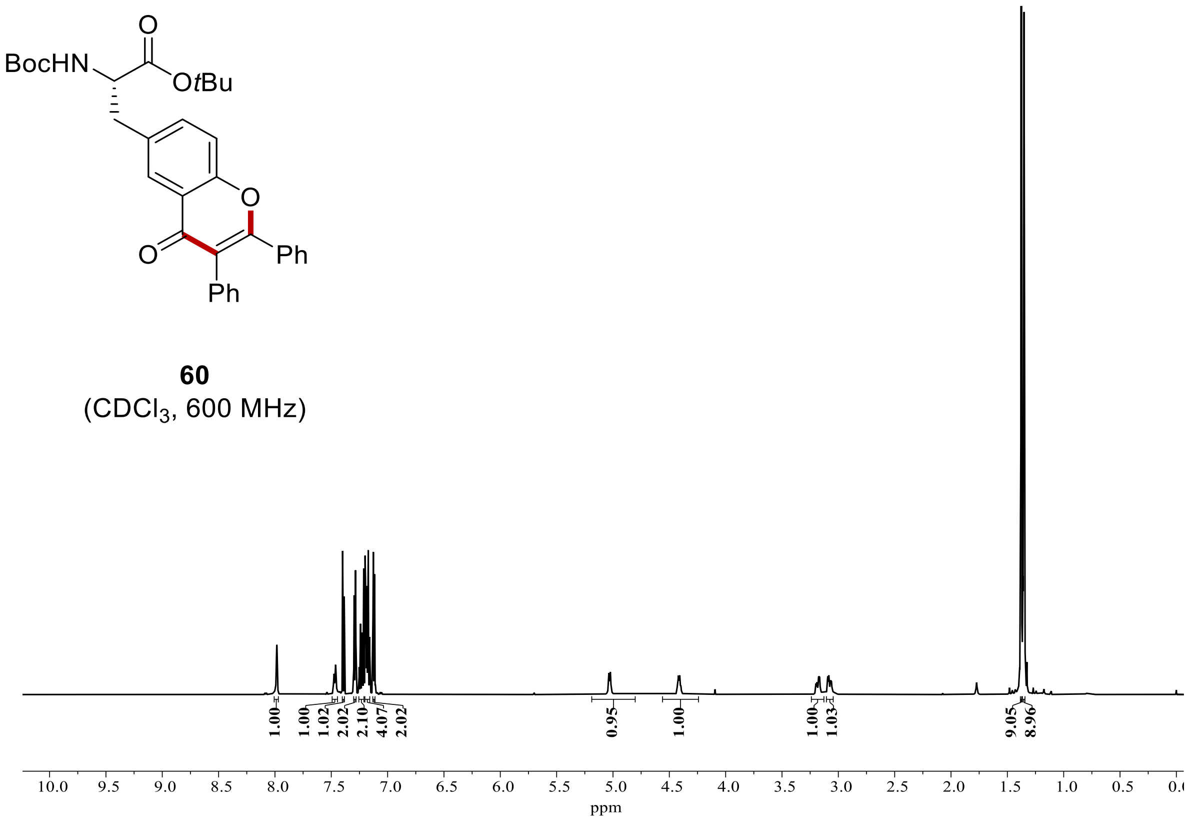

Supplementary Figure 145. ^{1}H-NMR spectrum of **60**.

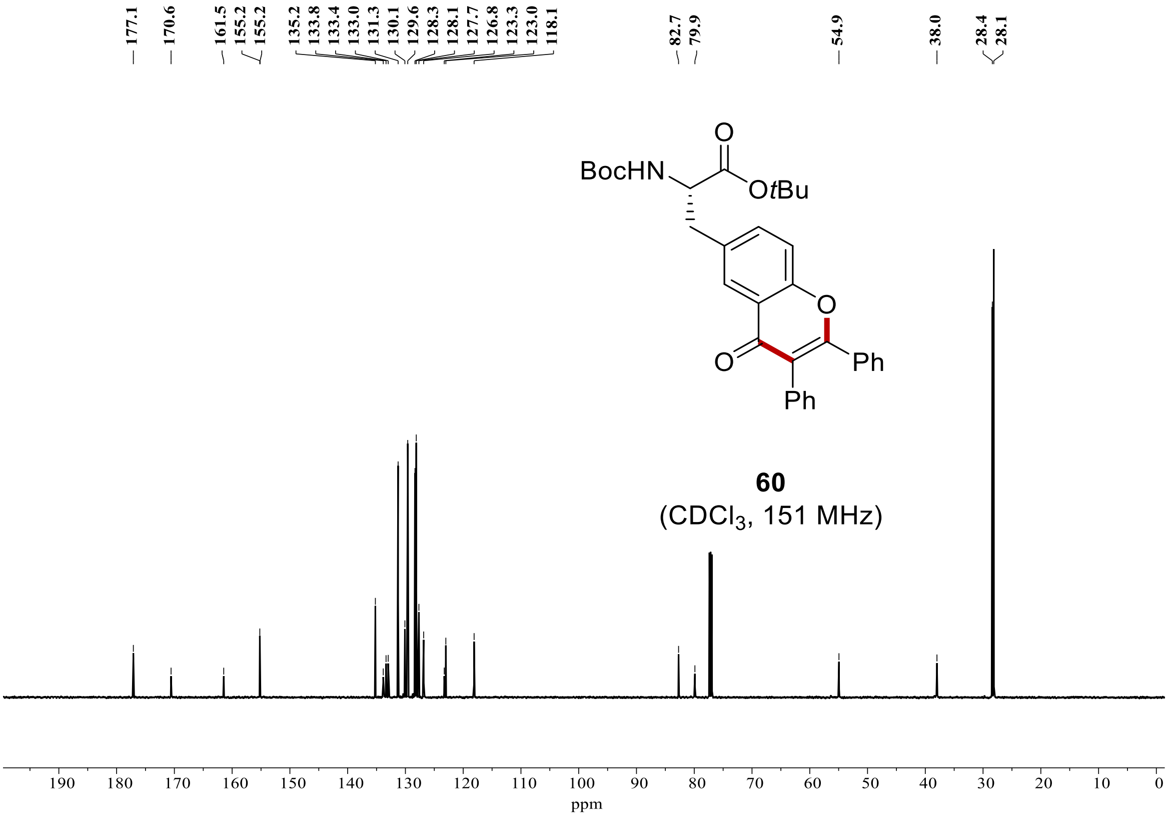

Supplementary Figure 146. ^{13}C-NMR spectrum of **60**.

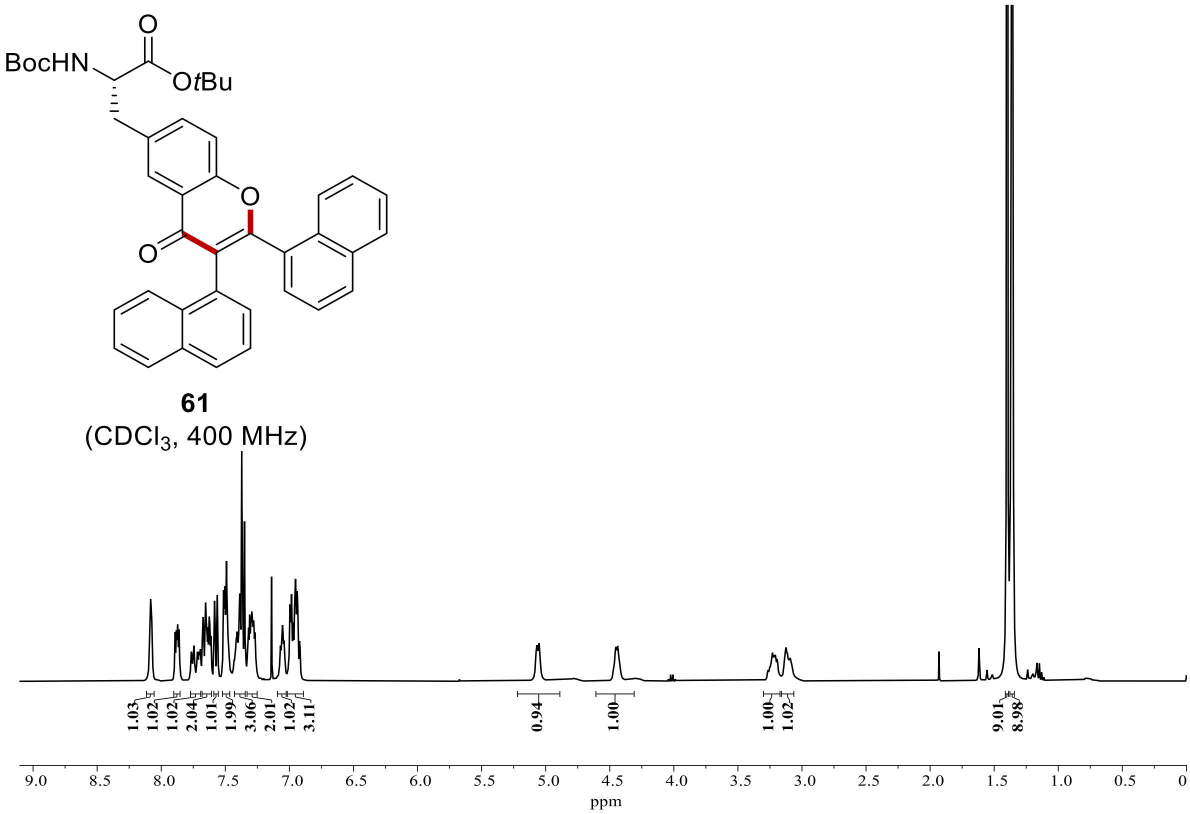

Supplementary Figure 147. ^{1}H-NMR spectrum of **61**.

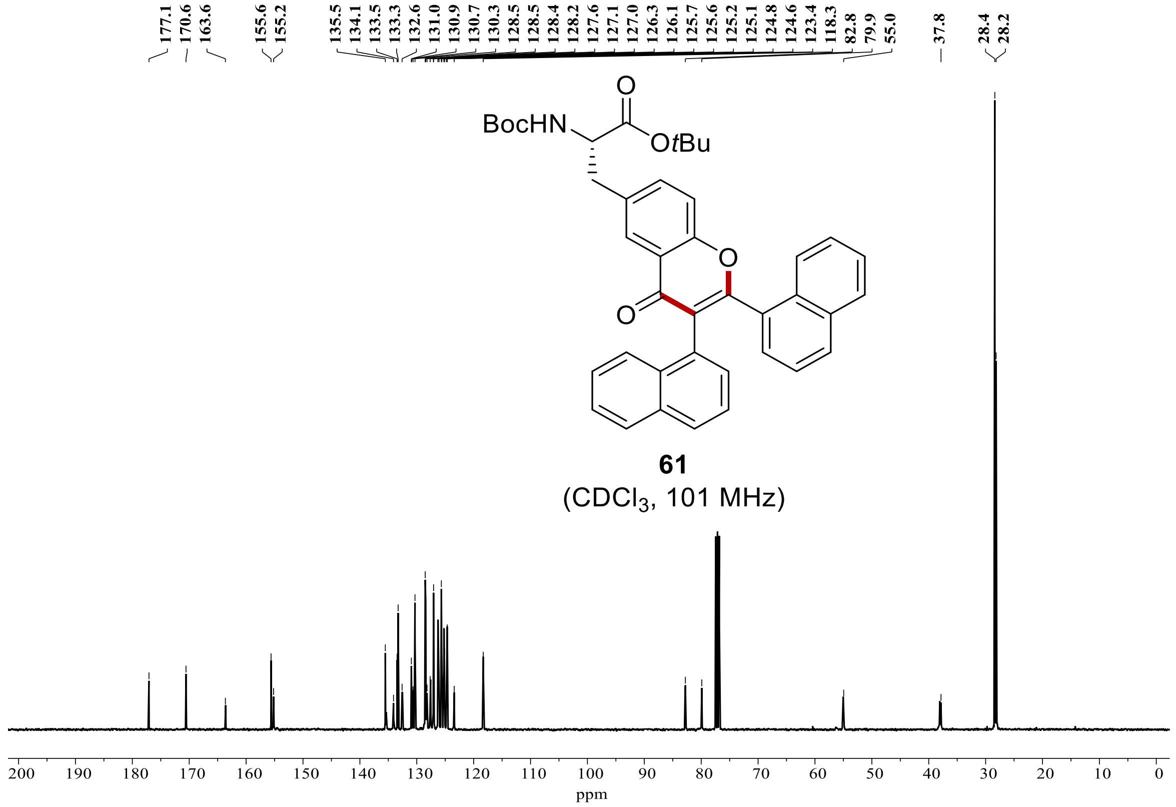

Supplementary Figure 148. ^{13}C-NMR spectrum of **61**.

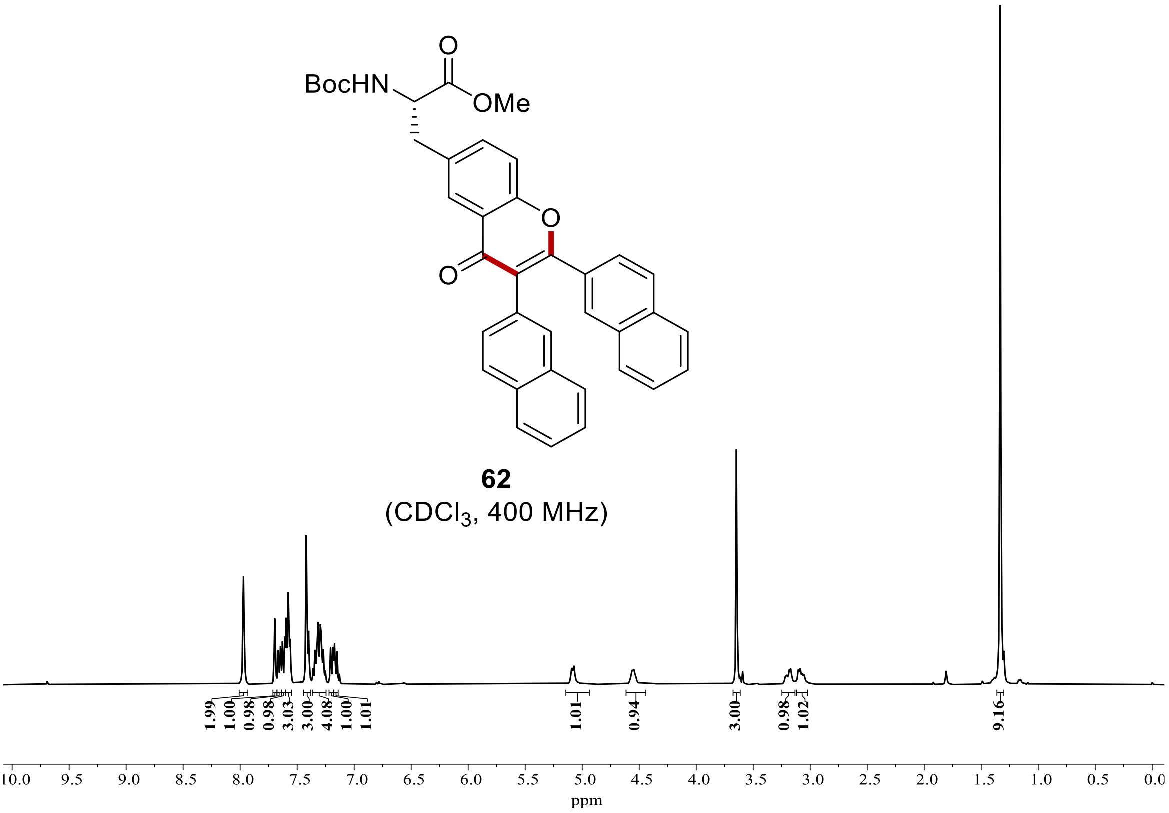

Supplementary Figure 149. ^{1}H-NMR spectrum of **62**.

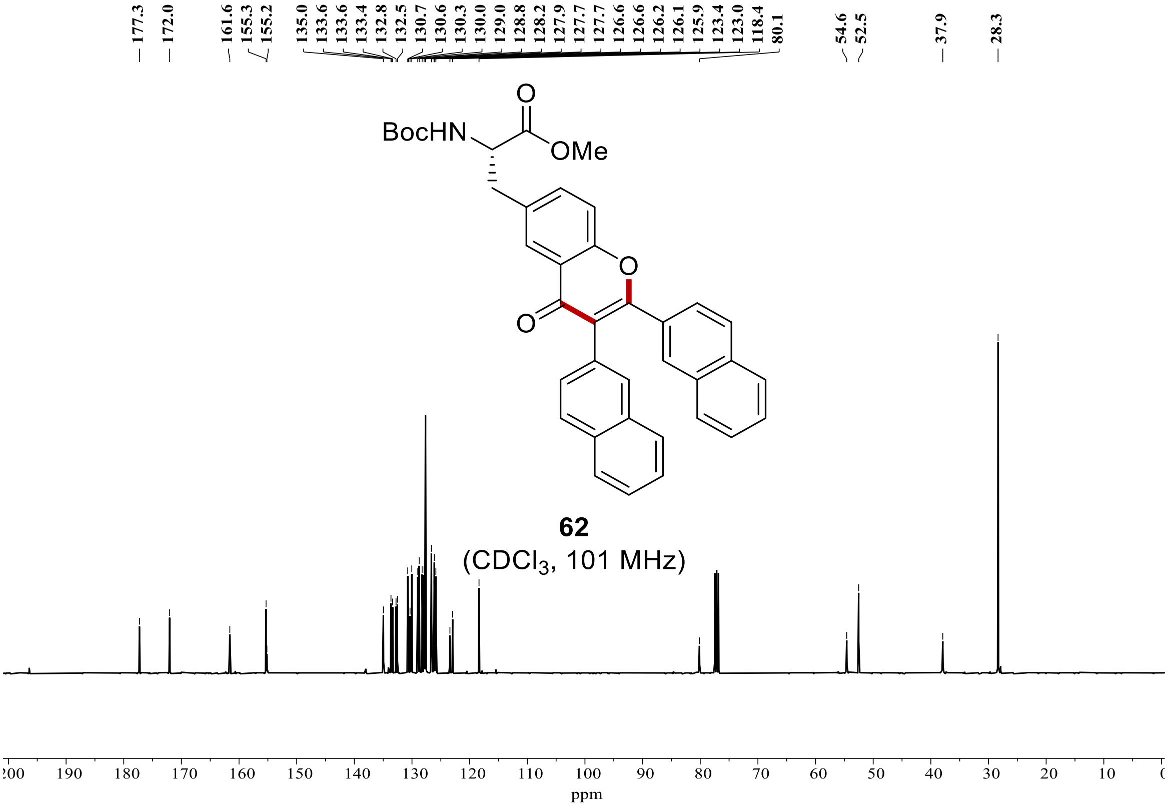

Supplementary Figure 150. ^{13}C-NMR spectrum of **62**.

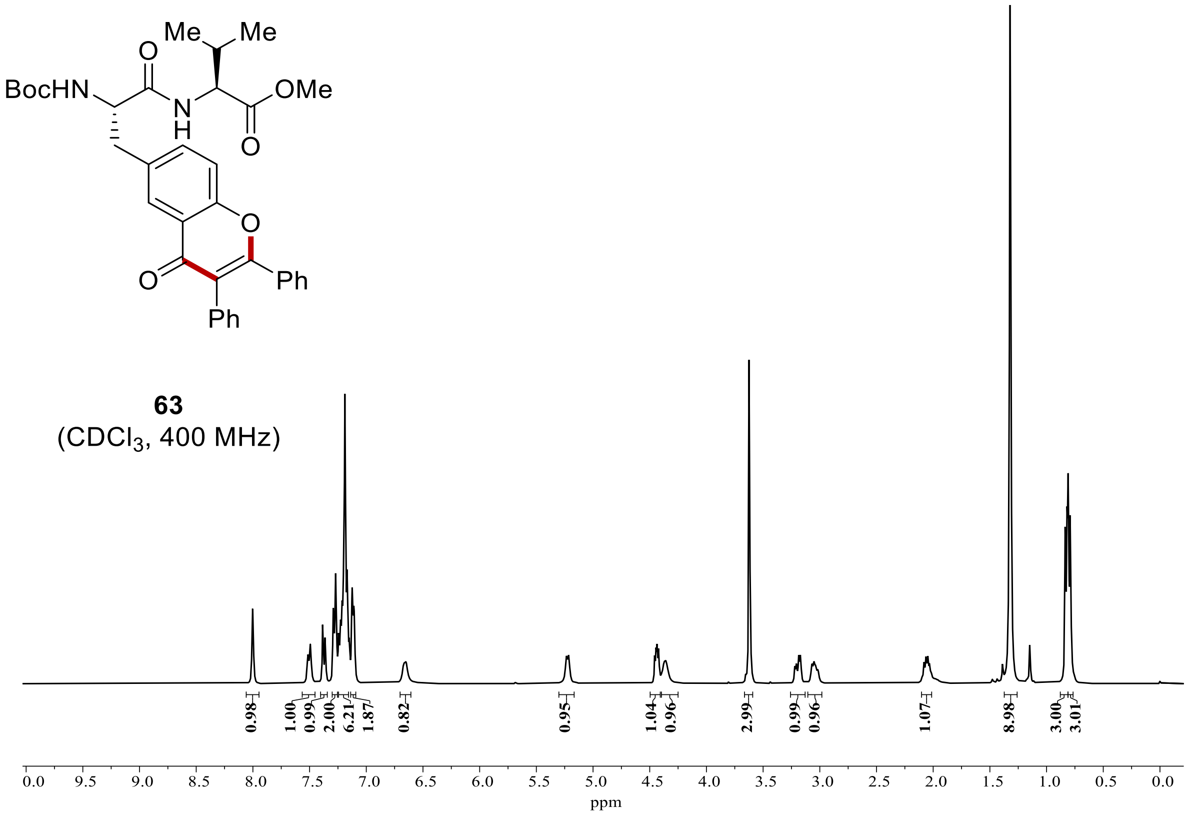

Supplementary Figure 151. ^{1}H-NMR spectrum of **63**.

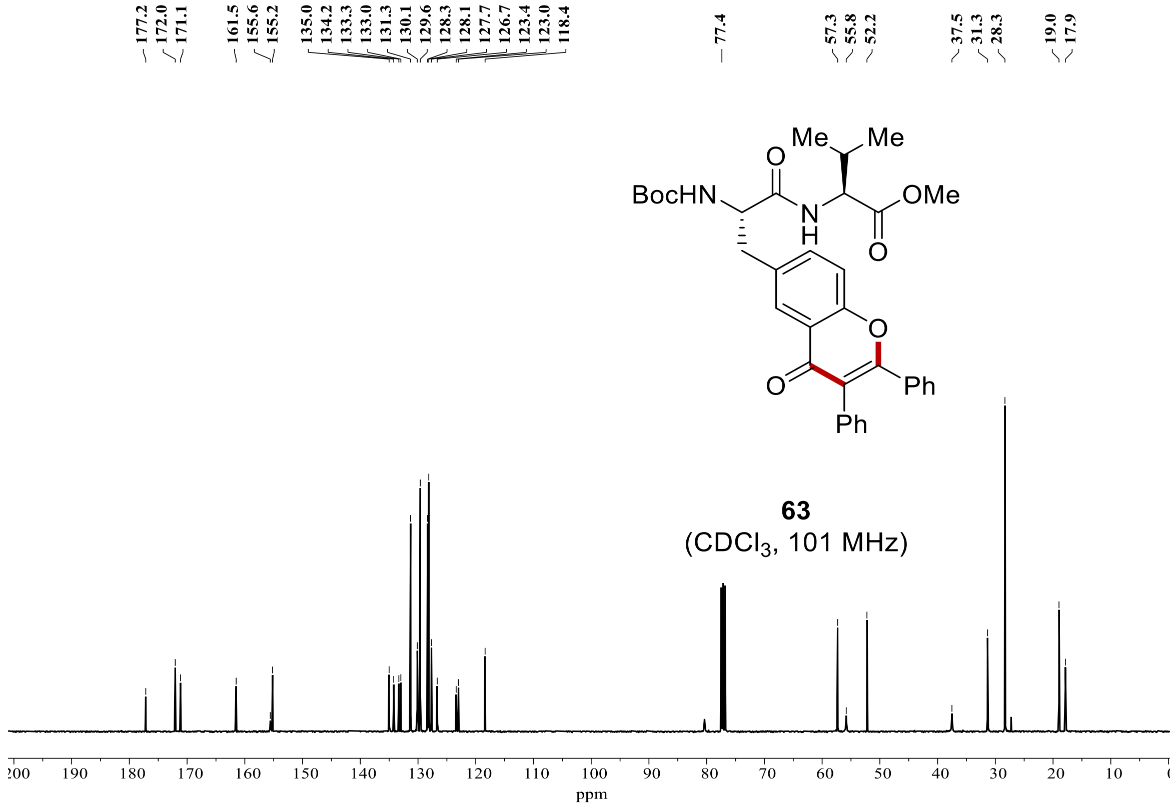

Supplementary Figure 152. ^{13}C-NMR spectrum of **63**.

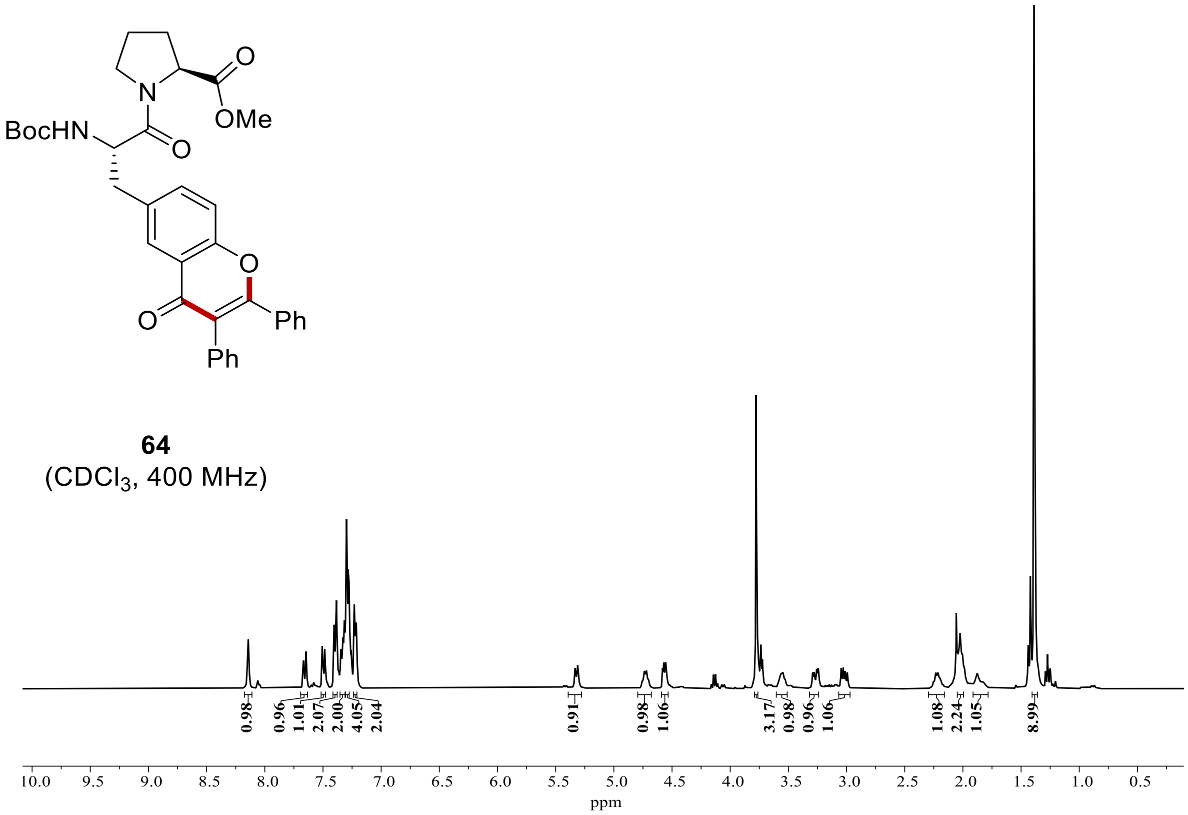

Supplementary Figure 153. ^{1}H-NMR spectrum of **64**.

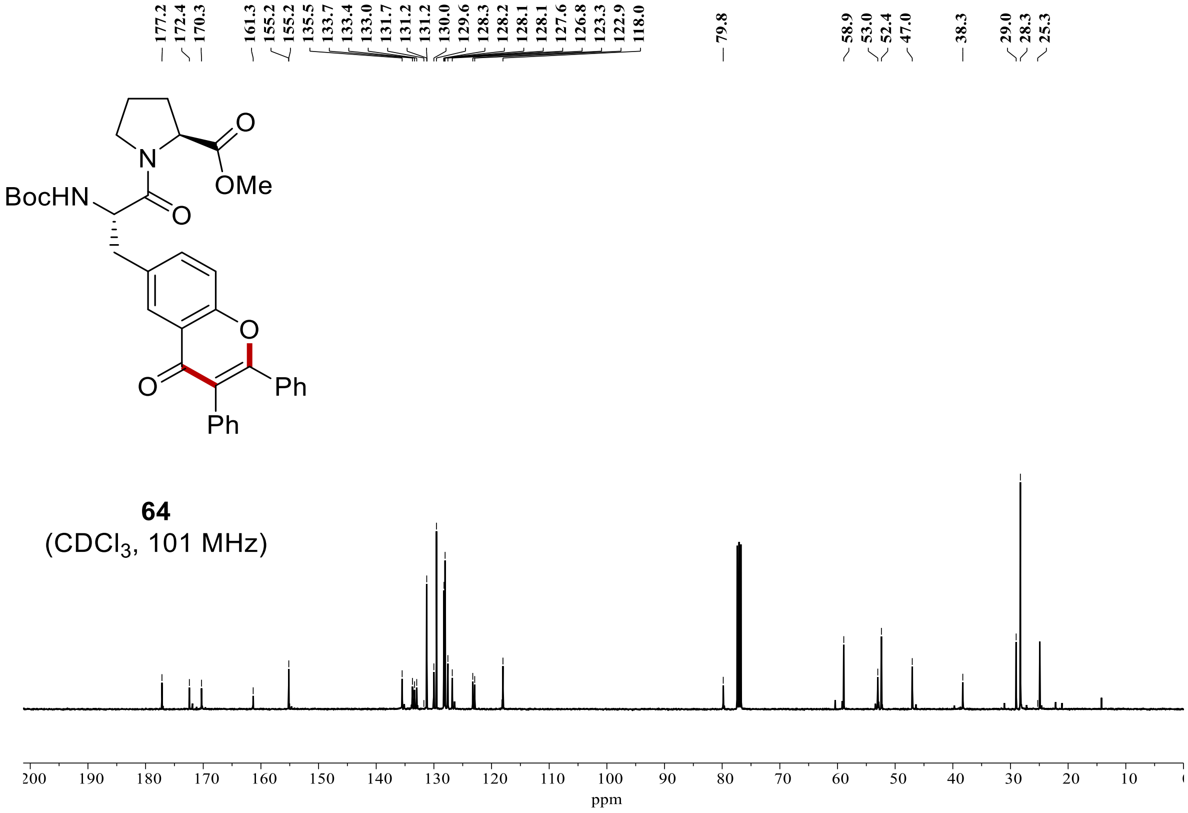

Supplementary Figure 154. ^{13}C-NMR spectrum of **64**.

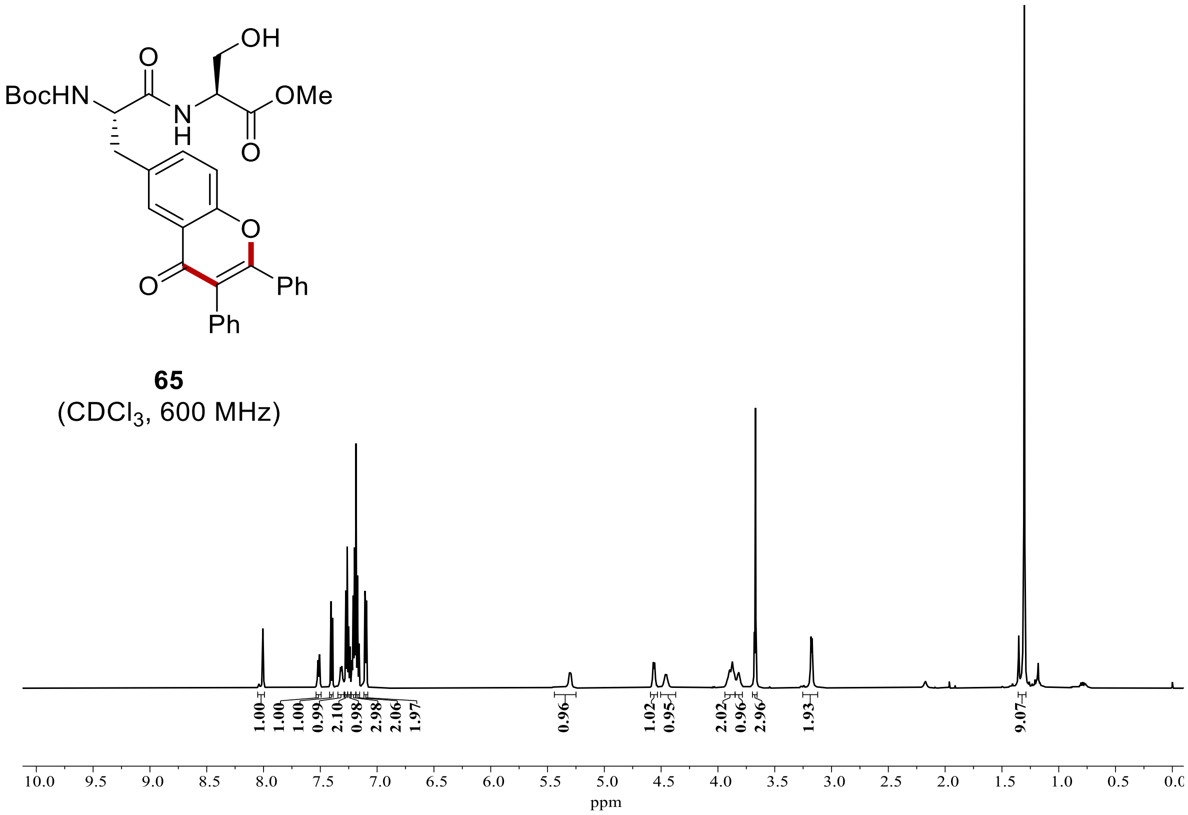

Supplementary Figure 155. ^{1}H-NMR spectrum of **65**.

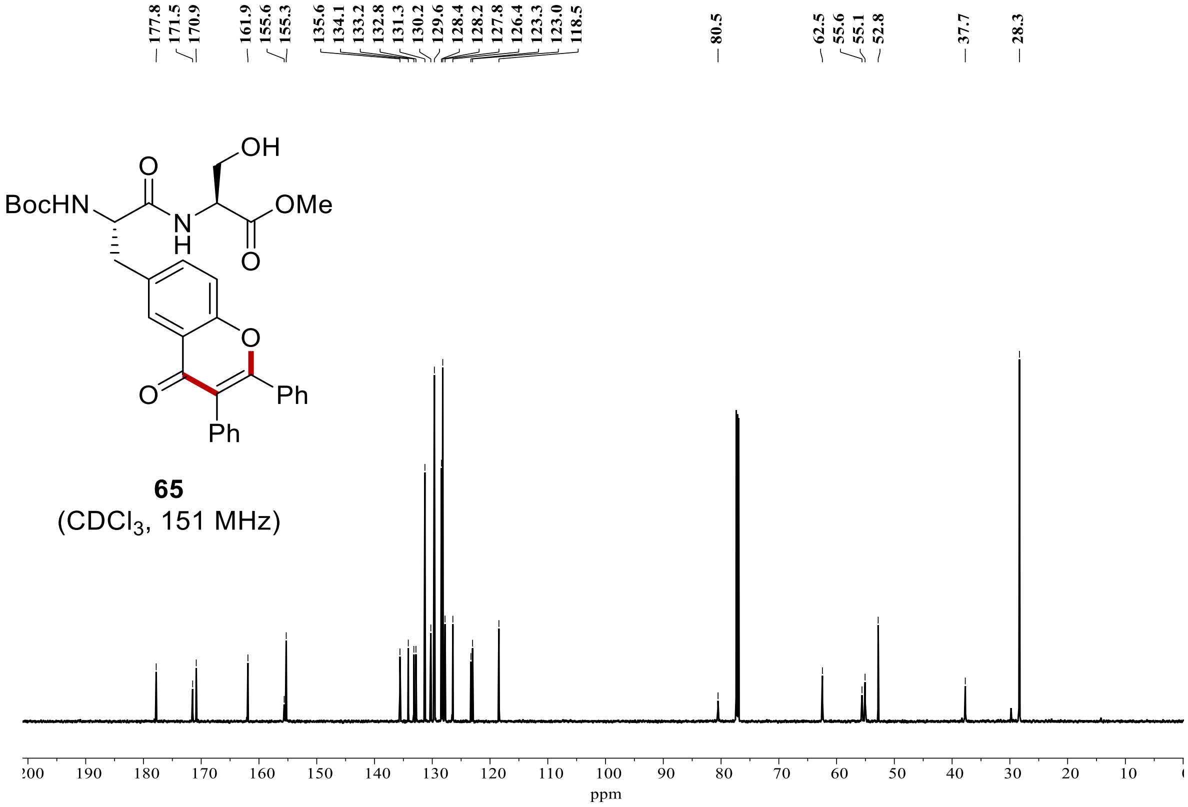

Supplementary Figure 156. ^{13}C-NMR spectrum of **65**.

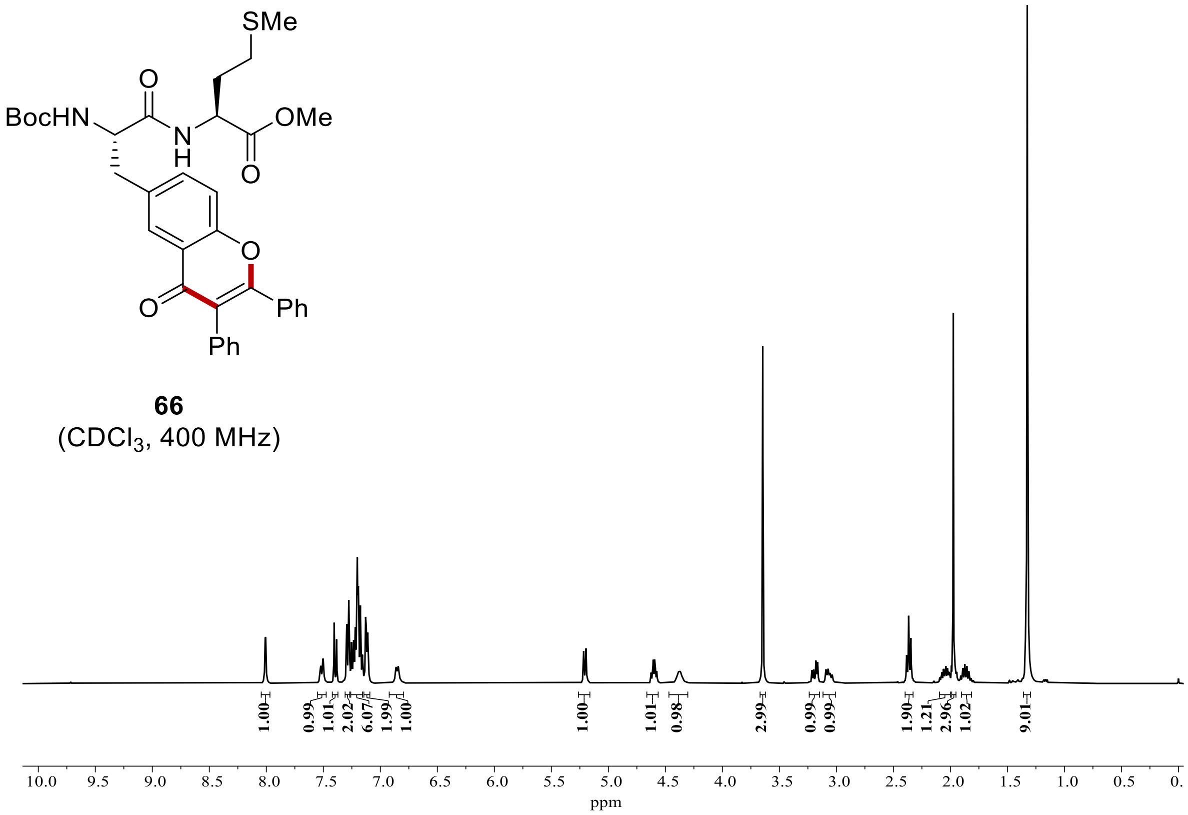

Supplementary Figure 157. ^{1}H-NMR spectrum of **66**.

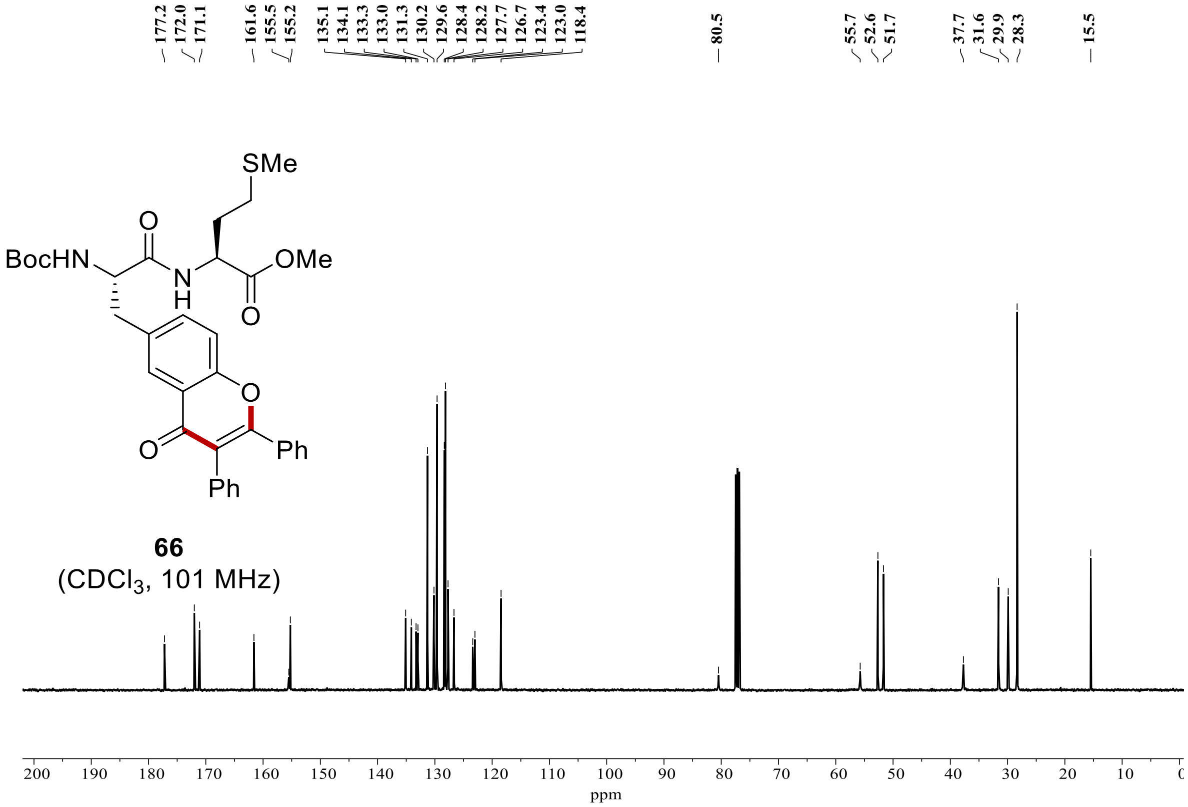

Supplementary Figure 158. ^{13}C-NMR spectrum of **66**.

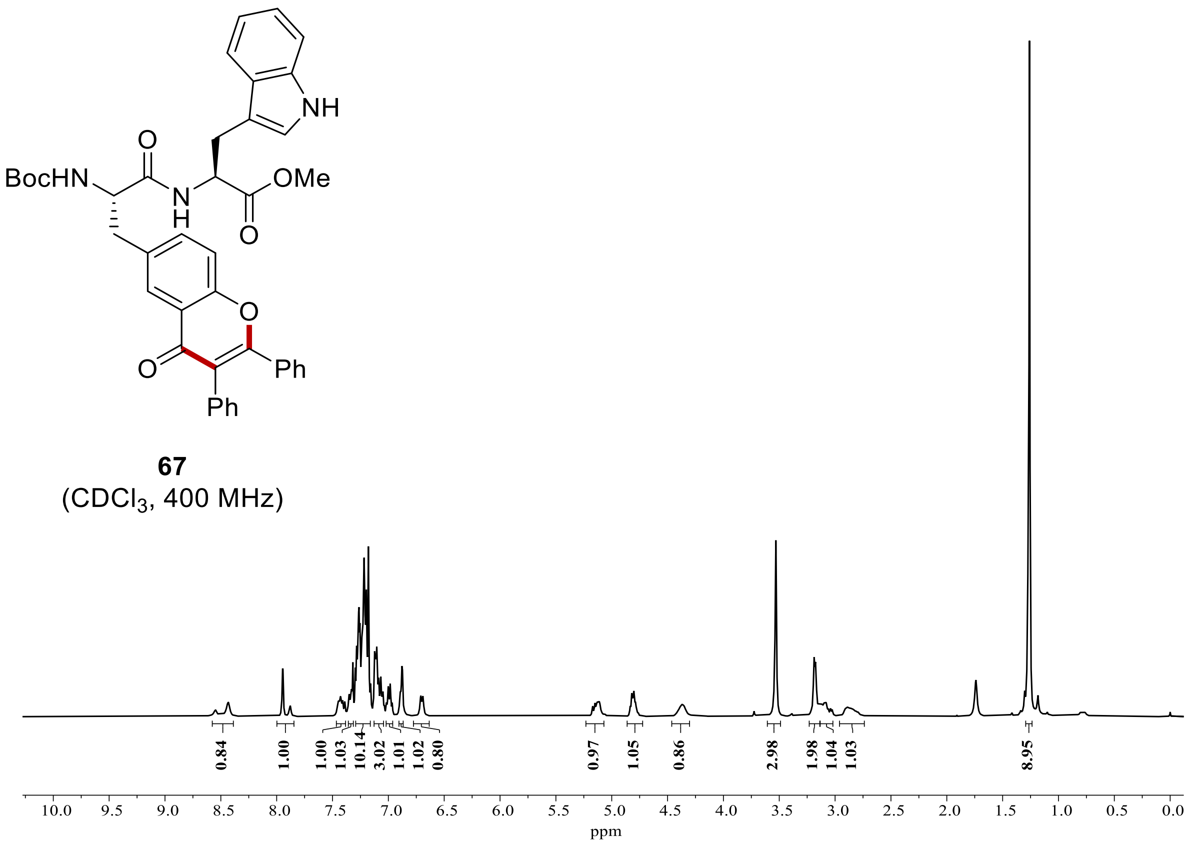

Supplementary Figure 159. ^{1}H-NMR spectrum of **67**.

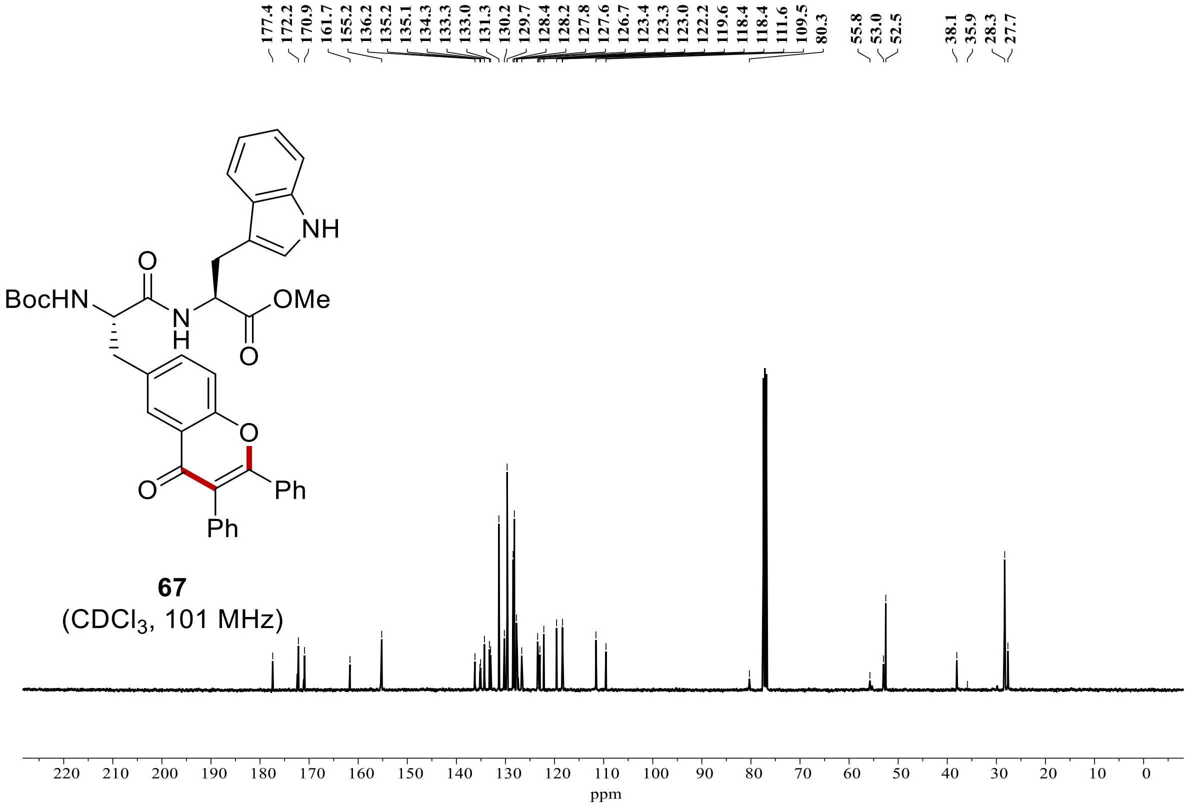

Supplementary Figure 160. ^{13}C-NMR spectrum of **67**.

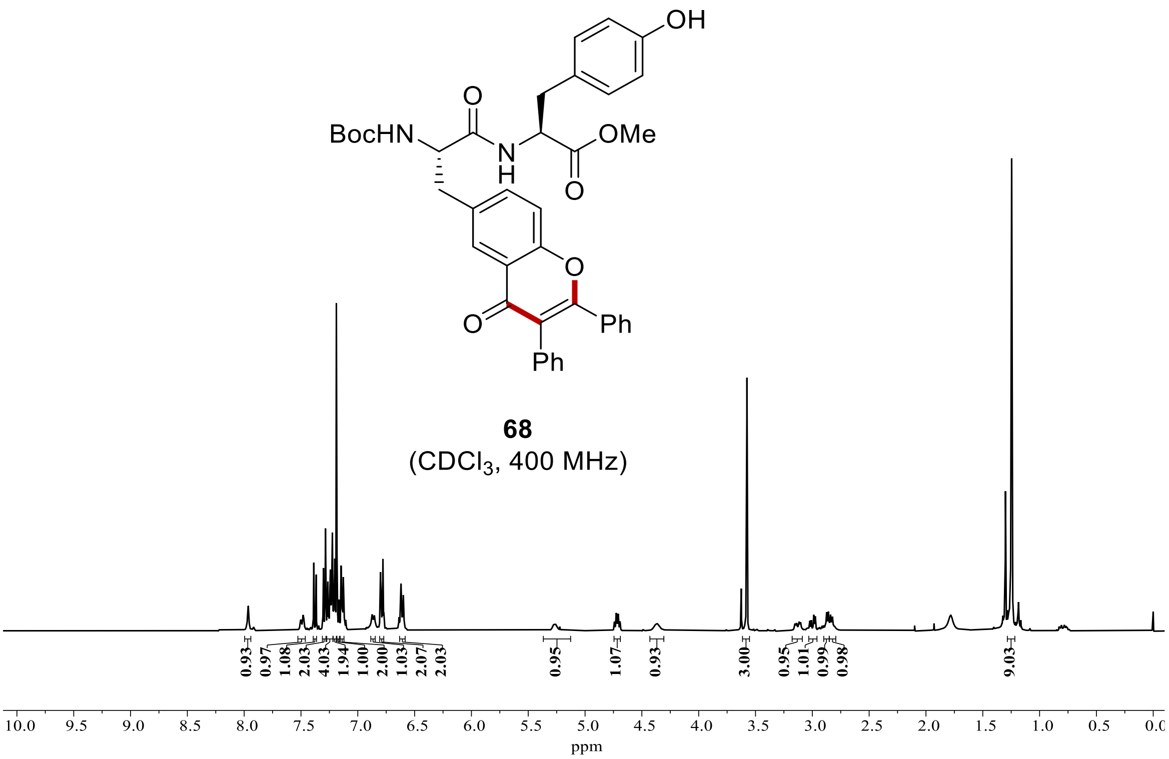

Supplementary Figure 161. ^{1}H-NMR spectrum of **68**.

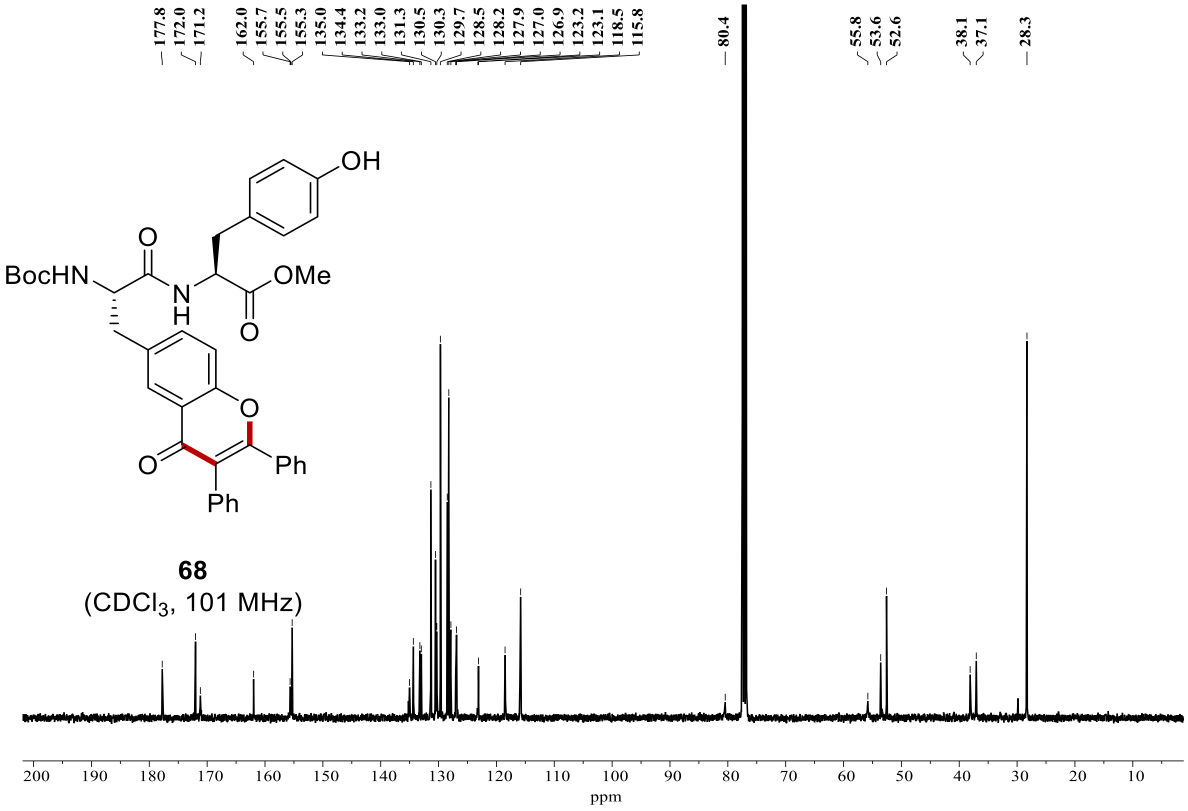

Supplementary Figure 162. ^{13}C-NMR spectrum of **68**.

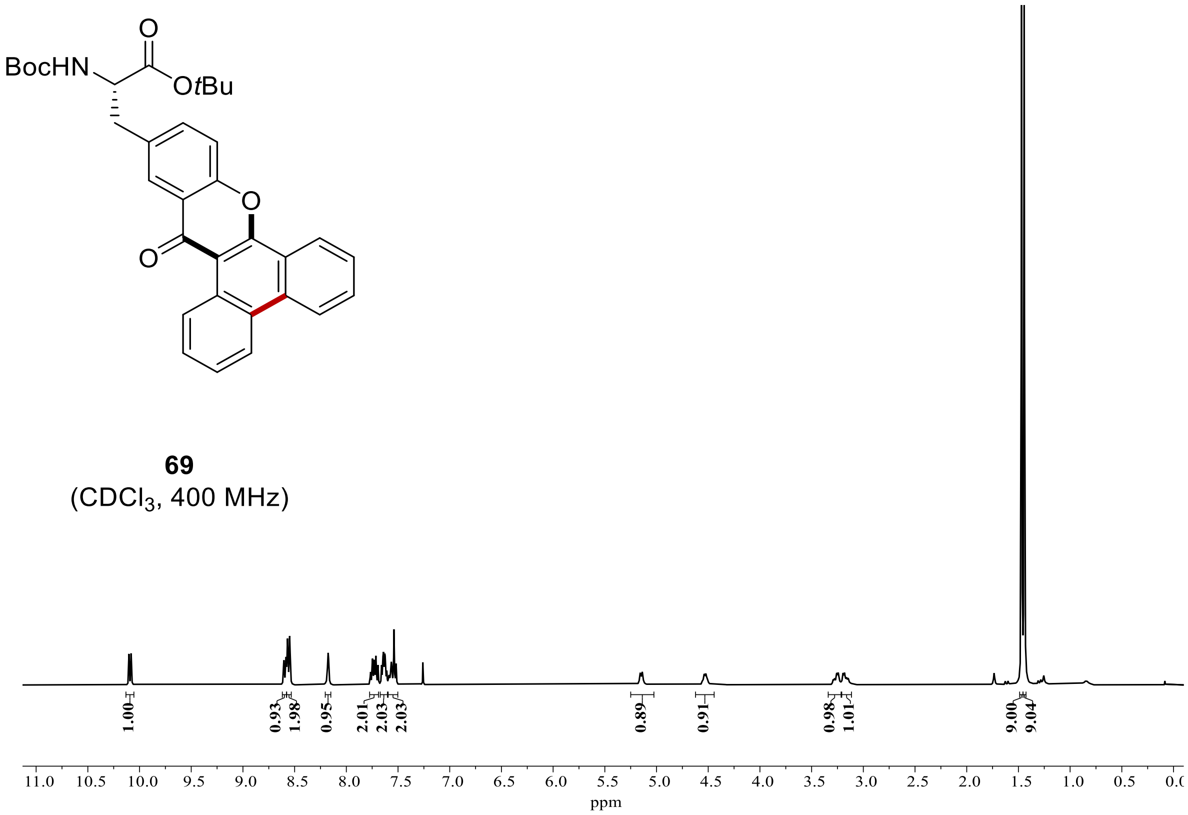

Supplementary Figure 163. ^{1}H-NMR spectrum of **69**.

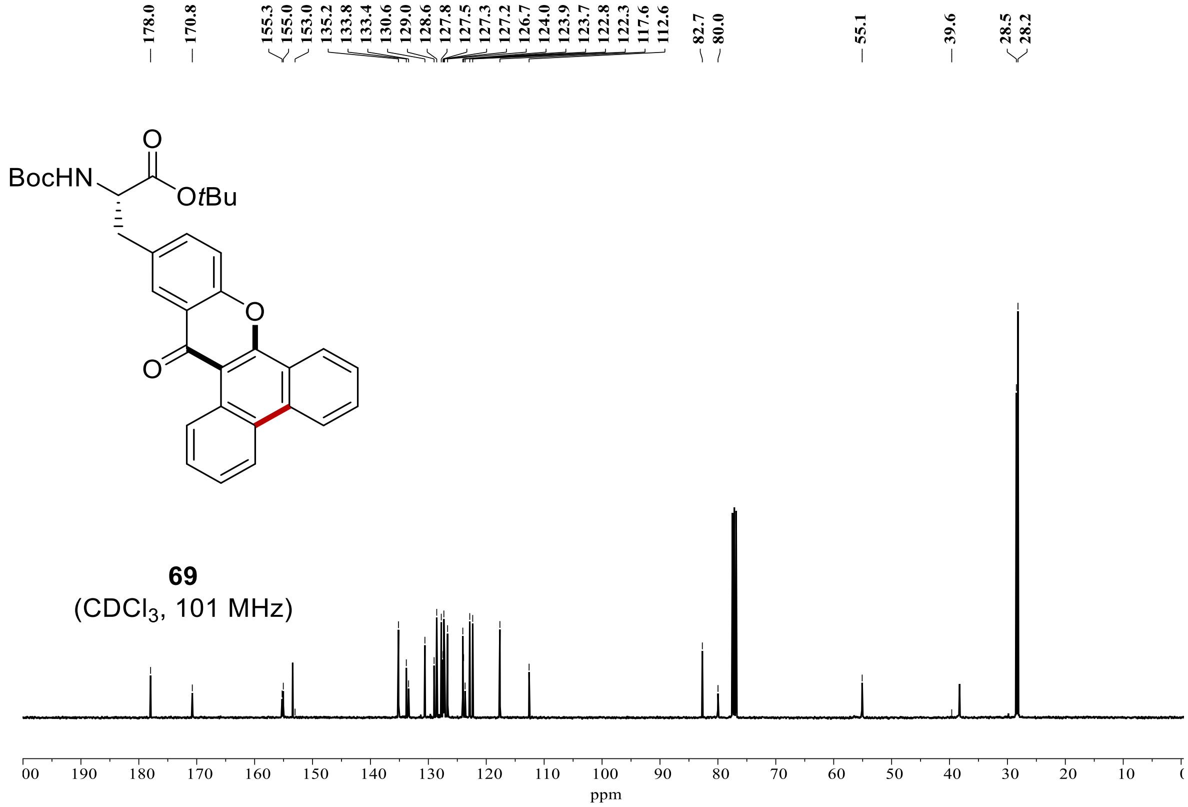

Supplementary Figure 164. ^{13}C-NMR spectrum of **69**.

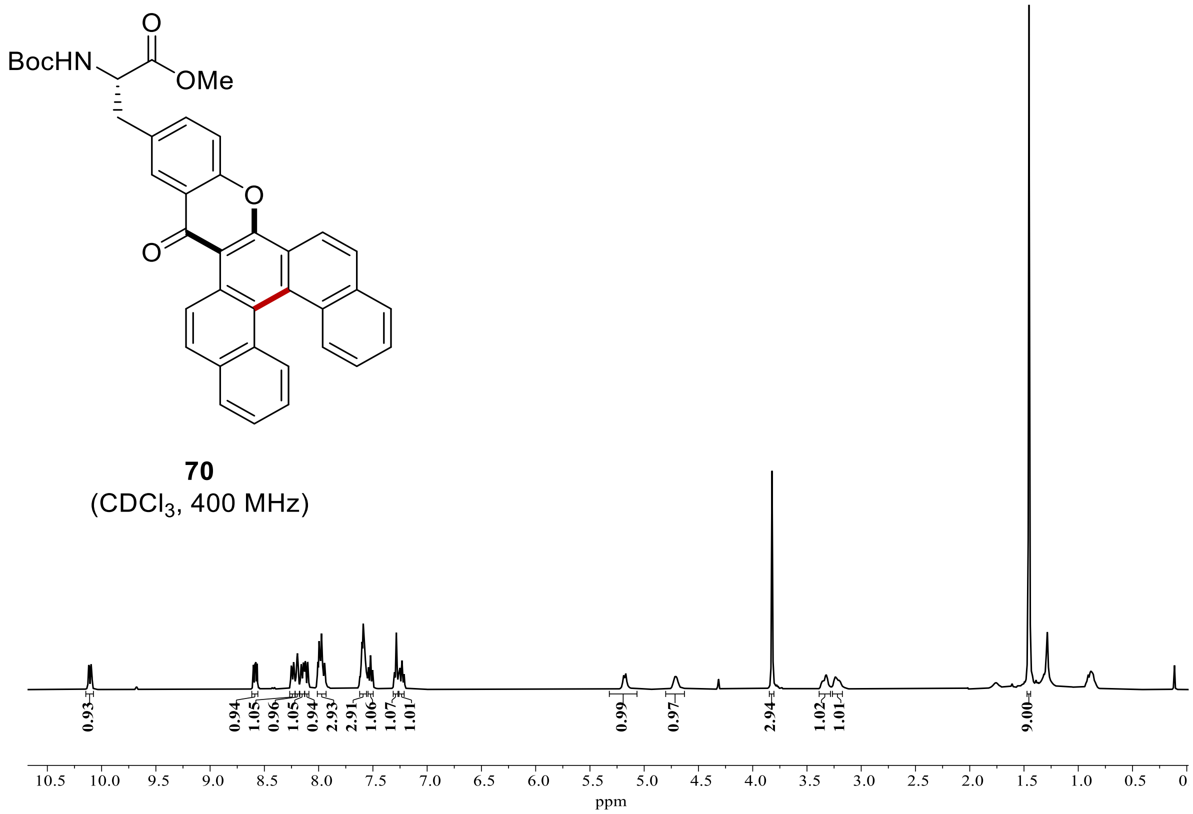

Supplementary Figure 165. ^{1}H-NMR spectrum of **70**.

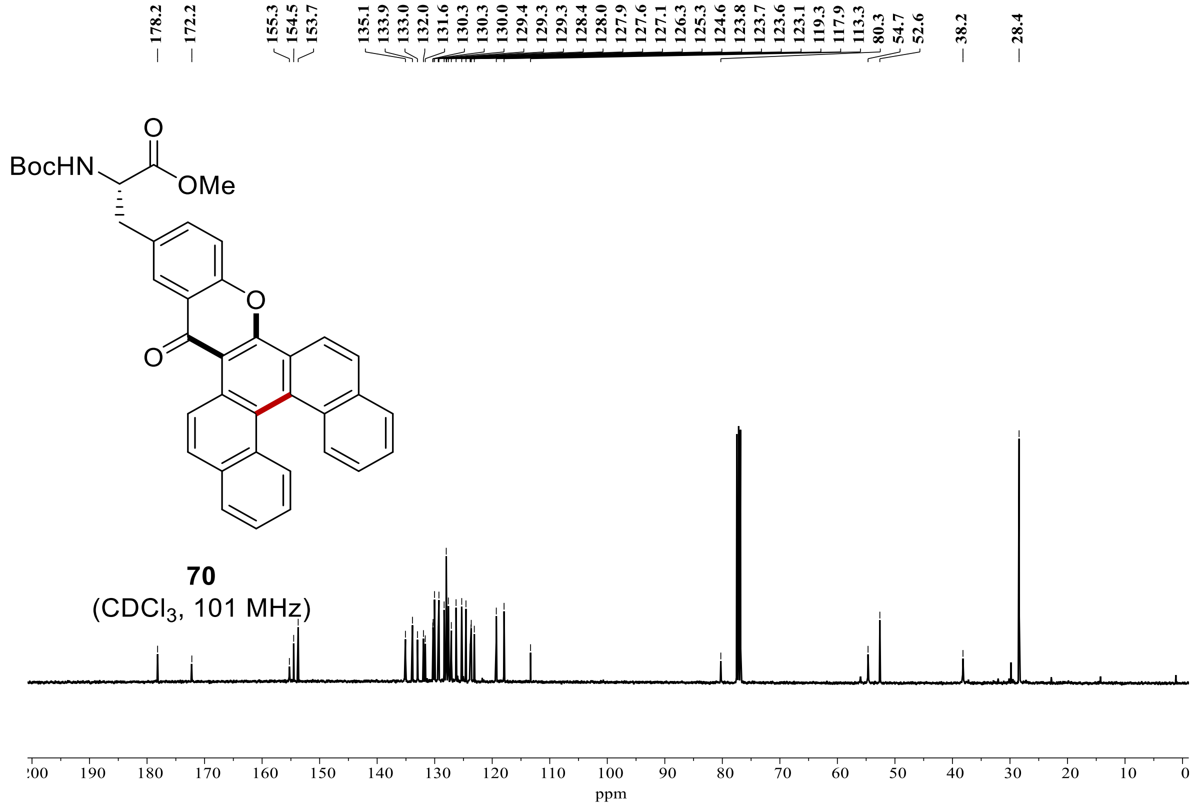

Supplementary Figure 166. ^{13}C-NMR spectrum of **70**.

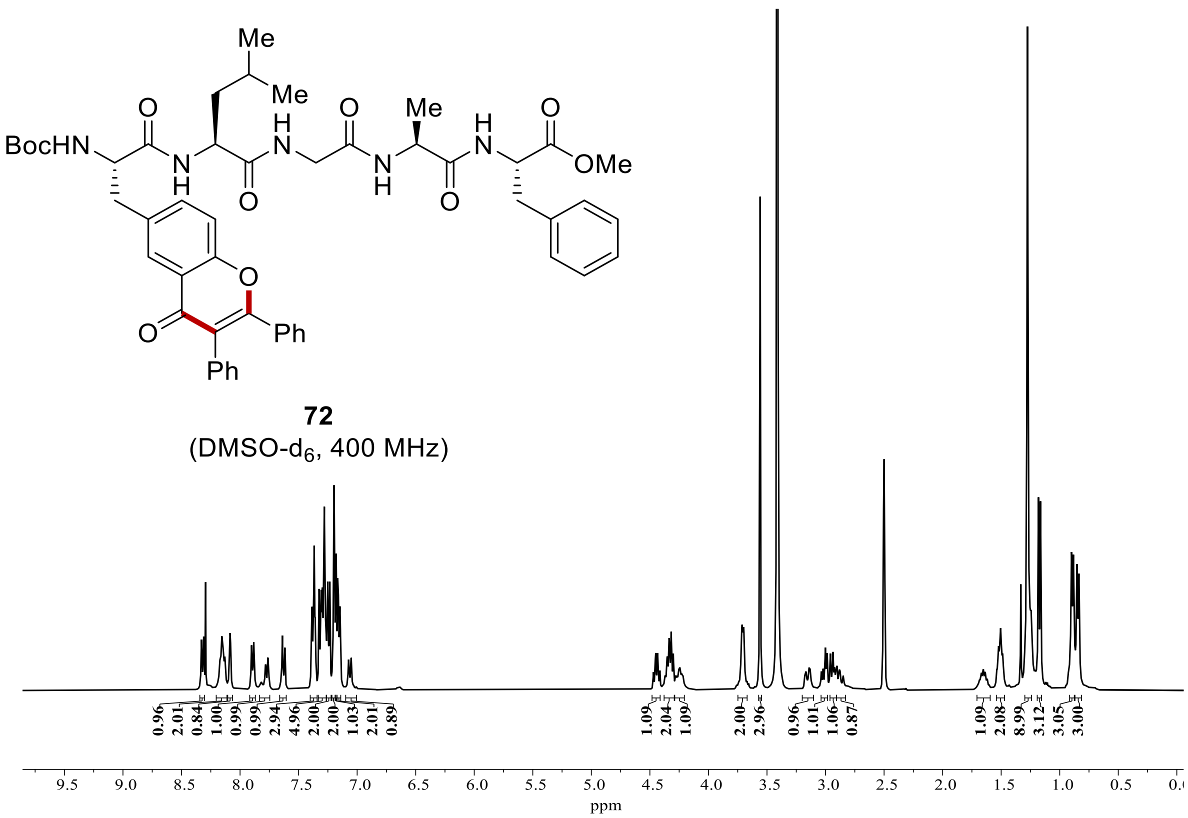

Supplementary Figure 167. ^{1}H-NMR spectrum of **72**.

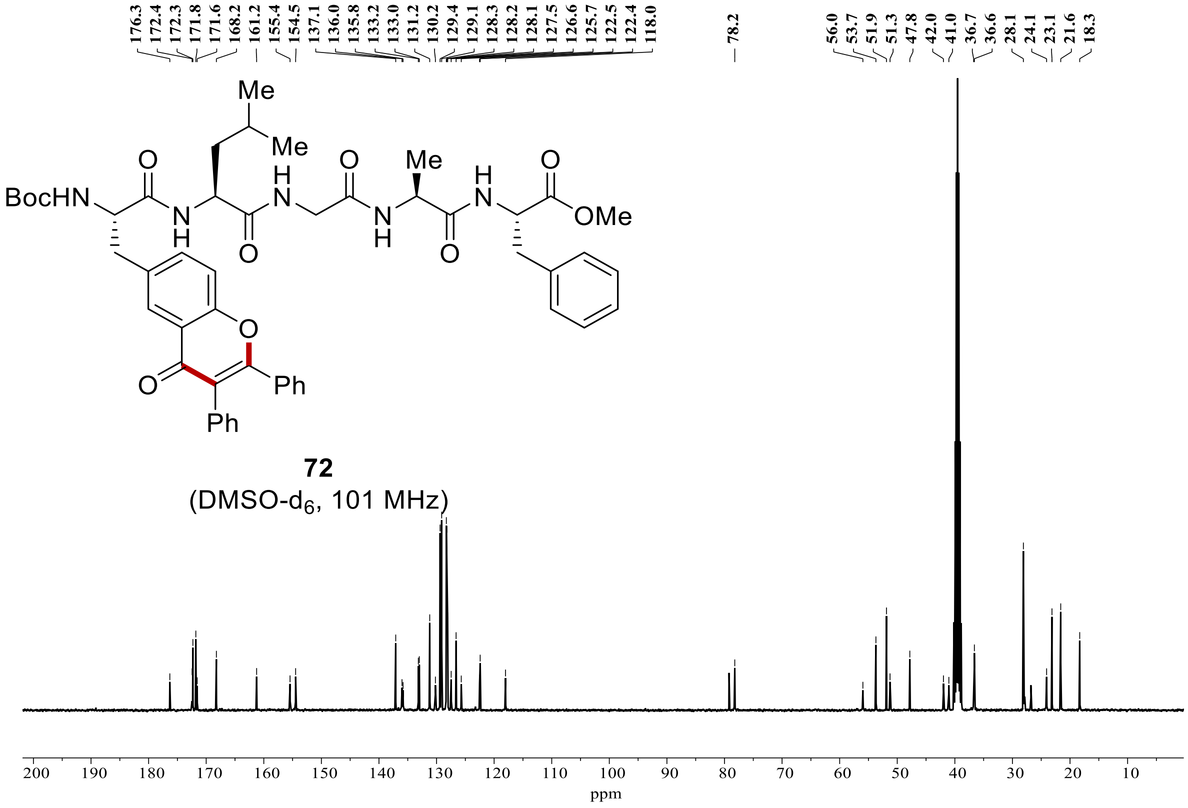

Supplementary Figure 168. ^{13}C-NMR spectrum of **72**.

17. Supplementary References

1 Mio, M. J., Kopel, L. C., Braun, J. B., Gadzikwa, T. L., Hull, K. L., Brisbois, R. G., Markworth, C. J. & Grieco, P. A. One-Pot Synthesis of Symmetrical and Unsymmetrical Bisarylethynes by a Modification of the Sonogashira Coupling Reaction. *Org. Lett.* **4**, 3199–3202, (2002).

2 Van den Hoven, B. G., Ali, B. E. & Alper, H. Chemo- and Regioselective Cyclohydrocarbonylation of α-Keto Alkynes Catalyzed by a Zwitterionic Rhodium Complex and Triphenyl Phosphite. *J. Org. Chem.* **65**, 4131-4137, (2000).

3 Strübing, D., Neumann, H., Klaus, S., Hübner, S. & Beller, M. A facile and efficient synthesis of enyne-reaction precursors by multicomponent reactions. *Tetrahedron* **61**, 11333-11344, (2005).

4 Pierce, B. M., Simpson, B. F., Ferguson, K. H. & Whittaker, R. E. Phosphine-mediated partial reduction of alkynes to form both (E)- and (Z)-alkenes. *Org. Biomol. Chem.* **16**, 6659-6662, (2018).

5 Baruah, S., Kaishap, P. P. & Gogoi, S. Ru(II)-Catalyzed C–H activation and annulation of salicylaldehydes with monosubstituted and disubstituted alkynes. *Chem. Comm.* **52**, 13004–13007, (2016).

6 Hirano, K., Biju, A. T., Piel, I. & Glorius, F. N-Heterocyclic Carbene-Catalyzed Hydroacylation of Unactivated Double Bonds. *J. Am. Chem. Soc.* **131**, 14190–14191, (2009).

7 Raju, B. C., Tiwari, A. K., Kumar, J. A., Ali, A. Z., Agawane, S. B., Saidachary, G. & Madhusudana, K. α-Glucosidase inhibitory antihyperglycemic activity of substituted chromenone derivatives. *Bioorg. Med. Chem.* **18**, 358–365, (2010).

8 Djukic, B., Poddutoori, P. K., Dube, P. A., Seda, T., Jenkins, H. A. & Lemaire, M. T. Bimetallic Iron(3+) Spin-Crossover Complexes Containing a 2,2′-Bithienyl Bridging bis-QsalH Ligand. *Inorg. Chem.* **48**, 6109–6116, (2009).

9 Hofsløkken, N. U. S., Lars. Convenient Method for the ortho-Formylation of Phenols. *Acta Chem. Scand.* **53**, 258–262, (1999).

10 Moon, D. J., Al-Amin, M., Lewis, R. S., Arnold, K. M., Yap, G. P. A., Sims-Mourtada, J. & Chain, W. J. A Strategy toward Icetexane Natural Products. *Eur. J. Org. Chem.* **2018**, 3348–3351, (2018).

11 Shen, K., Qi, L. & Ravula, M. Facile Incorporation of a Phosphatase Activity-Dependent Quinone Methide Generating Motif into Phosphotyrosine. *Synthesis* **2009**, 3765–3768, (2009).

12 Kalesh, K. A., Tan, L. P., Lu, K., Gao, L., Wang, J. & Yao, S. Q. Peptide-based activity-based probes (ABPs) for target-specific profiling of protein tyrosine phosphatases (PTPs). *Chem. Comm.* **46**, 589–591, (2010).

13 Kang, Y., Wang, T., Liang, Y., Zhang, Y., Wang, R. & Zhang, Z. Annulation of 2,3-diphenyl-4H-chromen-4-ones via photo-induced hydrogen evolution. *RSC Adv.* **7**, 44333–44339, (2017).

14 Wu, Z.-J., Su, F., Lin, W., Song, J., Wen, T.-B., Zhang, H.-J. & Xu, H.-C. Scalable Rhodium(III)-Catalyzed Aryl C−H Phosphorylation Enabled by Anodic Oxidation Induced Reductive Elimination. *Angew. Chem. Int. Ed.* **58**, 16770–16774, (2019).

15 Kong, W.-J., Finger, L. H., Messinis, A. M., Kuniyil, R., Oliveira, J. C. A. & Ackermann, L. Flow Rhodaelectro-Catalyzed Alkyne Annulations by Versatile C–H Activation: Mechanistic Support for Rhodium(III/IV). *J. Am. Chem. Soc.* **141**, 17198–17206, (2019).

16 Bruker. SAINT+ Integration Engine and Data Reduction Software v. v8.38A (Bruker AXS Inc., Madison, Wisconsin, USA, 2015).

17 Bruker. SADABS Bruker AXS Area Detector Scaling and Absorption Correction v. 2016/2 (Bruker AXS Inc., Madison, Wisconsin, USA, 2016).
18 Sheldrick, G. SHELXT - Integrated space-group and crystal-structure determination. *Acta Crystallogr. A* **71**, 3-8, (2015).
19 Sheldrick, G. Crystal structure refinement with SHELXL. *Acta Crystallogr. C* **71**, 3-8, (2015).
20 Dolomanov, O. V., Bourhis, L. J., Gildea, R. J., Howard, J. A. K. & Puschmann, H. OLEX2: a complete structure solution, refinement and analysis program. *J. Appl. Crystallogr.* **42**, 339-341, (2009).
21 Zhu, F., Wang, Z., Li, Y. & Wu, X.-F. Iridium-Catalyzed and Ligand-Controlled Carbonylative Synthesis of Flavones from Simple Phenols and Internal Alkynes. *Chem. Eur. J.* **23**, 3276–3279, (2017).
22 Bam, R. & Chalifoux, W. A. One-Pot Domino Friedel–Crafts Acylation/Annulation between Alkynes and 2-Methoxybenzoyl Chlorides: Synthesis of 2,3-Disubstituted Chromen-4-one Derivatives. *J. Org. Chem.* **83**, 9929–9938, (2018).
23 Shimizu, M., Tsurugi, H., Satoh, T. & Miura, M. Rhodium-catalyzed oxidative coupling between salicylaldehydes and internal alkynes with C–H bond cleavage to produce 2,3-disubstituted chromones. *Chem. Asian J.* **3**, 881–886, (2008).
24 Yoshida, M., Saito, K., Fujino, Y. & Doi, T. A concise synthesis of 3-aroylflavones via Lewis base 9-azajulolidine-catalyzed tandem acyl transfer–cyclization. *Chem. Comm.* **48**, 11796-11798, (2012).
25 Yoshida, M., Fujino, Y. & Doi, T. Synthesis of γ-Benzopyranone by TfOH-Promoted Regioselective Cyclization of o-Alkynoylphenols. *Org. Lett.* **13**, 4526-4529, (2011).
26 Frisch, M. J., Trucks, G. W., Schlegel, H. B., Scuseria, G. E., Robb, M. A., Cheeseman, J. R., Scalmani, G., Barone, V., Petersson, G. A., Nakatsuji, H., Li, X., Caricato, M., Marenich, A. V., Bloino, J., Janesko, B. G., Gomperts, R., Mennucci, B., Hratchian, H. P., Ortiz, J. V., Izmaylov, A. F., Sonnenberg, J. L., Williams, Ding, F., Lipparini, F., Egidi, F., Goings, J., Peng, B., Petrone, A., Henderson, T., Ranasinghe, D., Zakrzewski, V. G., Gao, J., Rega, N., Zheng, G., Liang, W., Hada, M., Ehara, M., Toyota, K., Fukuda, R., Hasegawa, J., Ishida, M., Nakajima, T., Honda, Y., Kitao, O., Nakai, H., Vreven, T., Throssell, K., Montgomery Jr, J. A., Peralta, J. E., Ogliaro, F., Bearpark, M. J., Heyd, J. J., Brothers, E. N., Kudin, K. N., Staroverov, V. N., Keith, T. A., Kobayashi, R., Normand, J., Raghavachari, K., Rendell, A. P., Burant, J. C., Iyengar, S. S., Tomasi, J., Cossi, M., Millam, J. M., Klene, M., Adamo, C., Cammi, R., Ochterski, J. W., Martin, R. L., Morokuma, K., Farkas, O., Foresman, J. B. & Fox, D. J. Gaussian 16 Rev. A.03 (Wallingford, CT, 2016).
27 Adamo, C. & Barone, V. Toward reliable density functional methods without adjustable parameters: The PBE0 model. *J. Chem. Phys.* **110**, 6158–6170, (1999).
28 Ernzerhof, M. & Scuseria, G. E. Assessment of the Perdew–Burke–Ernzerhof exchange-correlation functional. *J. Chem. Phys.* **110**, 5029–5036, (1999).
29 Grimme, S., Ehrlich, S. & Goerigk, L. Effect of the damping function in dispersion corrected density functional theory. *J. Comput. Chem.* **32**, 1456–1465, (2011).
30 Grimme, S., Antony, J., Ehrlich, S. & Krieg, H. A consistent and accurate ab initio parametrization of density functional dispersion correction (DFT-D) for the 94 elements H-Pu. *J. Chem. Phys.* **132**, 154104, (2010).
31 Weigend, F. Accurate Coulomb-fitting basis sets for H to Rn. *Phys. Chem. Chem. Phys.* **8**, 1057–1065, (2006).
32 Weigend, F. & Ahlrichs, R. Balanced basis sets of split valence, triple zeta valence and quadruple zeta valence quality for H to Rn: Design and assessment of accuracy. *Phys. Chem. Chem. Phys.* **7**, 3297–3305, (2005).

33 Schäfer, A., Horn, H. & Ahlrichs, R. Fully optimized contracted Gaussian basis sets for atoms Li to Kr. *J. Chem. Phys.* **97**, 2571–2577, (1992).

34 Schäfer, A., Huber, C. & Ahlrichs, R. Fully optimized contracted Gaussian basis sets of triple zeta valence quality for atoms Li to Kr. *J. Chem. Phys.* **100**, 5829–5835, (1994).

35 Martin, J. M. L. & Sundermann, A. Correlation consistent valence basis sets for use with the Stuttgart–Dresden–Bonn relativistic effective core potentials: The atoms Ga–Kr and In–Xe. *J. Chem. Phys.* **114**, 3408–3420, (2001).

36 Dolg, M., Wedig, U., Stoll, H. & Preuss, H. Energy-adjusted ab initio pseudopotentials for the first row transition elements. *J. Chem. Phys.* **86**, 866–872, (1987).

37 Zhao, Y. & Truhlar, D. G. Design of Density Functionals That Are Broadly Accurate for Thermochemistry, Thermochemical Kinetics, and Nonbonded Interactions. *J. Phys. Chem. A* **109**, 5656–5667, (2005).

38 Marenich, A. V., Cramer, C. J. & Truhlar, D. G. Universal Solvation Model Based on Solute Electron Density and on a Continuum Model of the Solvent Defined by the Bulk Dielectric Constant and Atomic Surface Tensions. *J. Phys. Chem. B* **113**, 6378–6396, (2009).

39 Contreras-García, J., Johnson, E. R., Keinan, S., Chaudret, R., Piquemal, J.-P., Beratan, D. N. & Yang, W. NCIPLOT: A Program for Plotting Noncovalent Interaction Regions. *J. Chem. Theory Comput.* **7**, 625–632, (2011).

40 Johnson, E. R., Keinan, S., Mori-Sánchez, P., Contreras-García, J., Cohen, A. J. & Yang, W. Revealing Noncovalent Interactions. *J. Am. Chem. Soc.* **132**, 6498–6506, (2010).

41 Roth, H. G., Romero, N. A. & Nicewicz, D. A. Experimental and Calculated Electrochemical Potentials of Common Organic Molecules for Applications to Single-Electron Redox Chemistry. *Synlett* **27**, 714–723, (2016).

42 Bao, D., Millare, B., Xia, W., Steyer, B. G., Gerasimenko, A. A., Ferreira, A., Contreras, A. & Vullev, V. I. Electrochemical Oxidation of Ferrocene: A Strong Dependence on the Concentration of the Supporting Electrolyte for Nonpolar Solvents. *J. Phys. Chem. A* **113**, 1259–1267, (2009).
